# Co^+^(C_2_H_2_)_*n*_ Complexes Studied with Selected-Ion Infrared Spectroscopy and Theory

**DOI:** 10.1021/acs.jpca.4c05304

**Published:** 2024-10-07

**Authors:** Anna G. Batchelor, Joshua H. Marks, Timothy B. Ward, Michael A. Duncan

**Affiliations:** Department of Chemistry, University of Georgia, Athens, Georgia 30602, United States

## Abstract

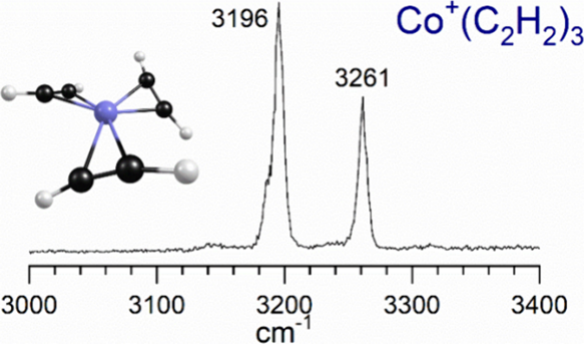

Co^+^(C_2_H_2_)_*n*_ (*n* = 1–6) complexes produced with laser vaporization in a supersonic molecular beam are studied with infrared photodissociation spectroscopy and computational chemistry. Infrared spectra are measured in the C–H stretching region using the method of tagging with argon atoms to enhance the photodissociation yields. C–H stretch vibrations for all clusters studied are shifted to lower frequencies than those of the well-known acetylene vibrations from ligand → metal charge transfer interactions. The magnitude of the red shifts decreases in the larger clusters as the interaction is distributed over more ligands. Computational studies identify various unreacted complexes with individual acetylene ligands in cation-π bonding configurations as well as reacted isomers in which ligand coupling reactions have taken place. Infrared spectra are consistent only with unreacted structures, even though the formation of reacted structures such as the metal ion-benzene complex is highly exothermic. Large activation barriers are predicted by theory along the reaction coordinates for the *n* = 2 and 3 complexes, which inhibit reactions in these smaller clusters, and this situation is presumed to persist in larger clusters.

## Introduction

Although not the main industrial process for benzene production, cycloaddition reactions that form benzene from acetylene are well-known and are recognized to involve significant activation barriers requiring carefully chosen catalysts and reaction conditions.^1−4^ The individual molecular reaction steps relevant for this chemistry have been investigated with gas phase ion chemistry and mass spectrometry measurements on metal cation-acetylene complexes, complemented by many computational studies.^5−25^ Our laboratory has extended this work using mass spectrometry combined with UV–visible or infrared spectroscopy and computational chemistry.^26−42^ In complexes containing multiple acetylene molecules, infrared spectroscopy makes it possible to identify the formation of “unreacted” cation-π complexes versus the products of ligand-coupling reactions. In the present work, we investigate the possible chemistry exhibited by cobalt cation-acetylene complexes. Cobalt is a well-known catalytic metal with a rich history in organometallic chemistry.^43^ In mass spectrometry experiments, its cations have been implicated in the catalytic cycloaddition reaction of acetylene to form benzene.^15,22^

Metal ion-acetylene complexes have been studied for many years in mass spectrometry. Early studies used collision-induced dissociation measurements^17^ or computational chemistry^6,10−12^ to investigate bond energies. Electronic spectroscopy explored excited states and structures for alkaline-earth metal complexes.^26−28^ More recently, scanned photodissociation thresholds and photofragment imaging were used to determine bond energies for Fe^+^(C_2_H_2_).^40^ Infrared photodissociation spectroscopy measurements by our group investigated the formation of cation-π complexes versus reactions in multiple-acetylene complexes for several transition metal ions.^29−39,41,42^ Vibrational bands in the C–H stretching region were found to shift to frequencies lower than those of acetylene because of the ligand → metal charge-transfer interaction. This charge transfer removes electron density from the acetylene ligands, weakening the acetylene bonding and reducing the vibrational frequencies. Computational studies predicted vibrational patterns for various metal complex structures and spin states. Band patterns for unreacted complexes were found to be clearly different from those for the products of ligand-coupling reactions. In the case of certain metal ions (Ni^+^, Cu^+^, Ag^+^, Au^+^, Fe^+^, and Pt^+^),^30,32,33,37,39,41^ reactions were inhibited by barriers, and cation-π complexes formed with individual acetylene ligands coordinating around the central metal ion. Ligands beyond the initial coordination formed solvation networks. In other systems (V, Zn, and Ti), ligand coupling reactions occur, and the products were detected via distinctive patterns in the infrared spectra.^35,36,41^ In the case of vanadium, ligand coupling reactions formed metallacycle intermediates and eventually the metal ion-benzene complex.^35^ Zinc ions produced a different chemistry, with end-to-end ligand coupling to form polyacetylene structures.^36^

Cobalt ion–molecule complexes have been studied with mass spectrometry and with photodissociation spectroscopy, both in the UV–visible and in the infrared.^44−60^ Brucat and co-workers reported vibrationally resolved electronic spectra for several Co^+^(L) complexes, where L = Ar, Kr, CO_2_, and N_2_.^44−49^ Our research group measured a similar spectrum for Co^+^Ne.^51^ Metz and co-workers obtained electronic spectra for Co^+^(CH_4_) and Co^+^(H_2_O), and employed IR-optical double resonance experiments on Co^+^(H_2_O).^57,58^ In the infrared, Co^+^(benzene) complexes were studied in the fingerprint region using the FELIX free electron laser.^55^ Our lab measured infrared spectra for Co^+^(CO)_*n*_ ions (*n* = 1–9)^56^ and for RG-Co^+^(H_2_O), where RG = Ar, Ne, and He.^60^ There have been no spectroscopy studies to our knowledge on other cation-π complexes of Co^+^, although Bowen and co-workers reported photoelectron spectroscopy of cobalt-benzene anions.^53^ Lau and co-workers investigated the ferromagnetism in Co_3_^+^(benzene)_0–3_ cluster ions using X-ray magnetic circular dichroism spectroscopy.^59^ In the case of cobalt-acetylene, Gan and co-workers explored the reactions of a distribution of Co_*n*_^+^ cations in a flow-tube apparatus at near-thermal conditions.^22^ On the basis of a large peak at the mass of Co^+^(benzene), they concluded that cycloaddition reactions had occurred. Photodissociation experiments indicated the loss of either benzene or three acetylenes, but there was no further spectroscopic confirmation of the identity of this product ion. The present infrared spectroscopic study of cobalt-acetylene cations therefore provides a valuable comparison to the other metal-acetylene and cobalt-ion systems studied previously.

## Methods

Cation-molecular complexes of the form Co^+^(C_2_H_2_)_*n*_ and Co^+^(C_2_H_2_)_*n*_Ar_*m*_ were produced by laser vaporization^61^ of a rotating metal rod in a pulsed supersonic expansion containing about 1% acetylene in argon. The ions were analyzed and selected for study with a reflectron time-of-flight mass spectrometer designed for photodissociation experiments.^62,63^ Mass selection was accomplished with pulsed deflection plates by using the transit time through the first flight tube section of the instrument. Photodissociation takes place at the turning point in the reflectron field, and fragment mass analysis is accomplished by using the flight time through the second flight tube section. Tunable infrared radiation for these experiments was provided by an Nd:YAG-pumped optical parametric oscillator/amplifier (OPO/OPA) laser system (LaserVision). Because the binding energies of acetylene ligands to cobalt cations are generally greater than the infrared photon energy, ion–molecule complexes were “tagged” with argon atoms to enhance photodissociation yields. IR excitation of acetylene vibrations leads to the elimination of argon from these complexes. Larger complexes with multiple ligands fragment by losing acetylene. In each case, the yield of the fragment ion mass was recorded versus the IR photon energy to obtain the infrared spectrum. Computational studies of complexes with or without argon were used to investigate the effects of argon attachment on the vibrational spectra.

Computational studies on the cobalt cation-acetylene complexes were carried out with the Gaussian16 program package,^64^ using density functional theory (DFT), the B3LYP functional, and the def2-TZVP basis set.^65^ Isomers of larger complexes were identified using manually selected structures based on our experience with several previous metal-acetylene systems and known organometallic chemistry. We have confidence that we have identified the lowest energy structures for the ligands coordinated directly to the acetylene, but for those complexes with multiple external ligands, there are many configurations lying close in energy, and finding the lowest of these is problematic. Infrared frequencies from theory were scaled by a factor of 0.96 for comparison to experimentally measured spectra. This factor was determined by comparing the computed frequencies for acetylene to its known experimental values and rounding them to two digits beyond the decimal. Energetics were corrected for the harmonic, nonscaled zero point energies. To identify transition states, we used the synchronous-transition-guided quasi-Newton (STQN) method in Gaussian16 with the QST3 command. Transition states were confirmed with subsequent TS and IRC calculations.

## Results and Discussion

**Figure 1:**
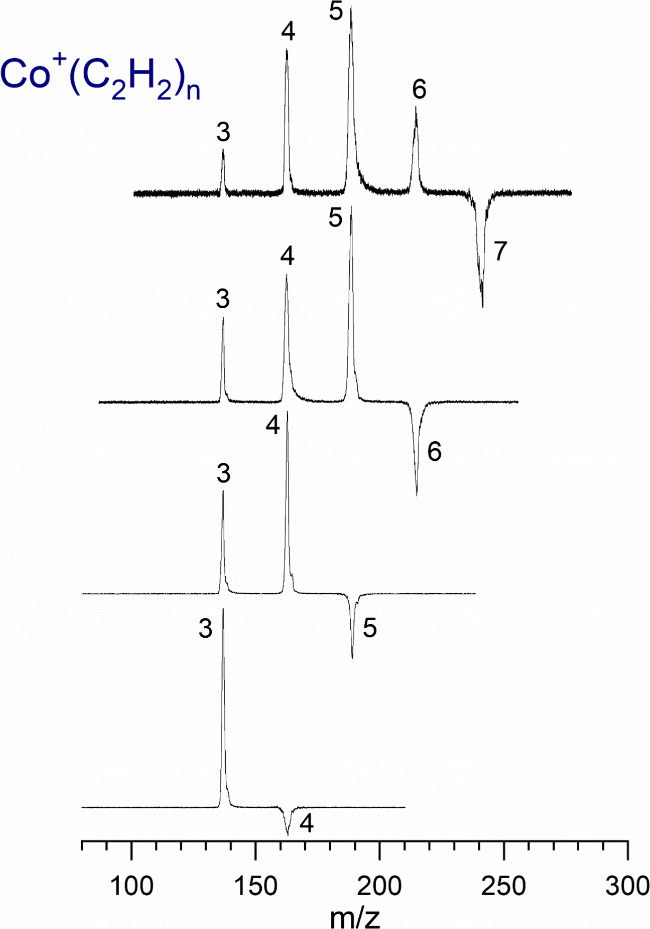
Fragmentation mass spectrum for the Co^+^(C_2_H_2_)_4–7_ complexes, showing the termination of fragmentation at the *n* = 3 complex ion.

Laser vaporization produces a distribution of Co^+^(C_2_H_2_)_*n*_ complexes similar to those previously produced for other metals. A representative example is presented as Figure S1 in the Supporting Information. By selecting different Co^+^(C_2_H_2_)_*n*_ complexes and tuning the infrared laser through the C–H stretching region, we find no efficient photodissociation for the smaller complexes (*n* ≤ 3), but photodissociation is detected for the larger complexes (*n* ≥ 4). Presumably, the small complexes do not dissociate because their bond energies are greater than the photon energy in the C–H stretching region. The larger clusters photodissociate by losing one or more intact acetylene molecules because these complexes have weakly bonded acetylene molecules in the second coordination sphere. The fragmentation mass spectra for these larger complexes are shown in Figure 1. This data was collected in a difference mode of operation in which the mass spectrum of a selected ion without the dissociation laser is subtracted from that with it on. The negative peak indicates the depletion of the selected parent ion, and the positive peaks indicate the photofragments produced. Each complex loses one or more intact acetylene molecules until it reaches the *n* = 3 complex, and then, no further fragmentation occurs. This data suggests that the coordination of acetylenes around Co^+^ is filled with three ligands. However, this may be misleading if other isomers are formed that have only strongly bonded ligands. The combination of these fragmentation patterns with infrared spectra (*vide infra*) makes it possible to confirm the coordination number. Coordination numbers for other metal ions with acetylene have been determined in this same way, with Cu^+^, Au^+^, Zn^+^, and Pt^+^ having coordination numbers of three^32,33,36,41^ and Ni^+^, Ag^+^, and Fe^+^ having coordination numbers of four.^30,37,39^

**Figure 2:**
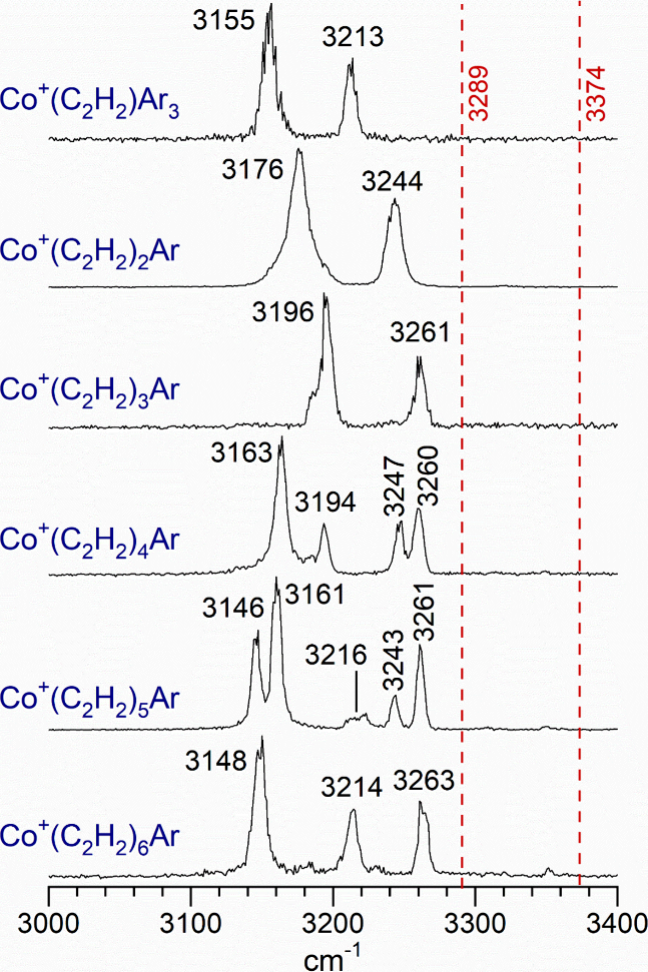
Infrared photodissociation spectra of the Co^+^(C_2_H_2_)_*n*_ complexes for *n* = 1–6. The *n* = 1 complex was tagged with three argon atoms, whereas the large clusters were tagged with a single argon.

The infrared photodissociation spectra of these complexes were recorded by measuring the yield of the respective fragment ions versus the infrared photon energy. Figure 2 shows the spectra obtained for the Co^+^(C_2_H_2_)_*n*_ complexes for *n* = 1–6. The *n* = 1 complexes were studied by tagging with three argons and measuring the mass channel corresponding to the elimination of one argon, whereas the other spectra were measured via tagging with and elimination of a single argon. Vibrational resonances in the C–H stretching region were obtained for each of these complexes, with bands near those of the isolated acetylene molecule.

The vibrational bands for these various Co^+^(C_2_H_2_)_*n*_ complexes appear at frequencies lower than those of the isolated acetylene molecule (antisymmetric and symmetric stretches at 3289 and 3374 cm^–1^, respectively).^66^ This is consistent with the behavior observed previously for other metal ion-acetylene complexes,^29−39,41,42^ and also with the expectations of the Dewar-Chatt-Duncanson model of cation-π interactions.^67−70^ The charged metal polarizes acetylene, drawing electron density toward the metal and out of the bonding molecular orbitals. This charge transfer interaction removes the bonding electron density along the C–H bonds, weakening the bonds and lowering the vibrational frequencies. An additional back-donation occurs in which an occupied *d* orbital donates electron density into the acetylene π* orbital. These interactions vary with different metal ions and complexes with different numbers of acetylene ligands. The effect is reduced for larger complexes that have the charge-polarization effect distributed over multiple ligands.

**Figure 3:**
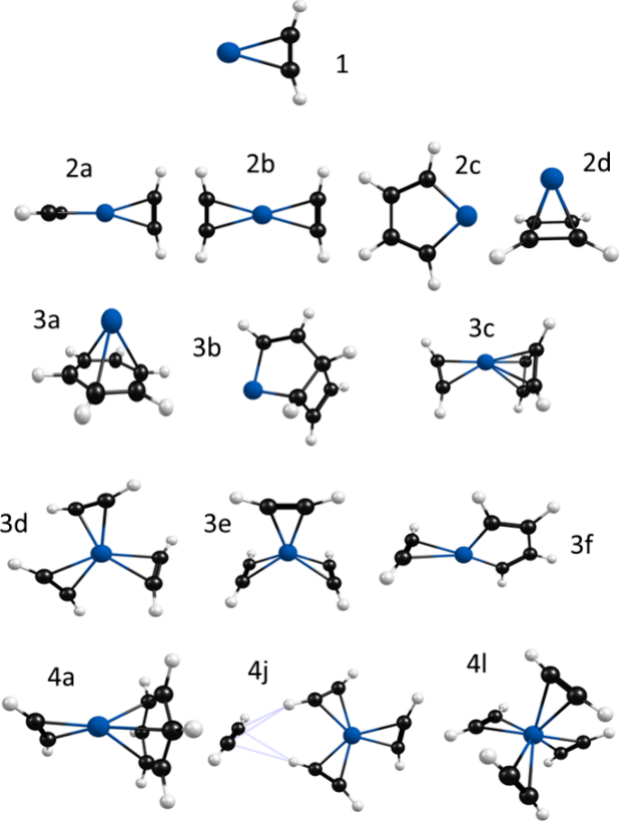
Structures for Co^+^(C_2_H_2_)_*n*_ complexes for *n* = 1–4 obtained from density functional theory computations. The isomers are labeled in the order of their energies. Selected isomers are shown for *n* = 4.

To compare specific vibrational patterns with different cluster structures, we performed computational studies on these cobalt ion-acetylene complexes. The important low-energy structures are shown in Figure 3; a complete description of all the structural isomers is presented in the Supporting Information. Because of the known atomic states of Co^+^,^71^ we investigated singlet, triplet, and quintet spin states, finding that the triplet spin state lies lowest in energy for each complex except the *n* = 5, for which some isomers are more stable as singlets. The *n* = 1 complex has the expected cation-π configuration, with the metal ion located in a symmetric position (*C*_2v_ symmetry) above the triple bond of acetylene. This same binding motif occurs throughout many of the isomers of the larger clusters. In the *n* = 1 structure, the C–H bonds bend away from the metal ion, with an H–C–C angle of 164°; smaller distortions like this occur in the larger clusters. The *n* = 2 structures include those with two acetylenes in cation-π positions opposite each other (isomers 2a and 2b, with *D*_2d_ and *D*_2h_ symmetry) and reacted structures in which these ligands are coupled into a metallacycle (2c) or cyclobutadiene (2d) structure. The most stable *n* = 3 isomer (3a) is that in which three acetylenes have coupled to form benzene. Isomers 3b and 3c have cyclobutadienyl moieties on one side, whereas isomers 3d and 3e have three unreacted ligands in cation-π configurations. Isomer 3f has a metallacycle structure with one cation-π acetylene. There are too many *n* = 4 isomers to show here, but we select the most stable isomer 4a which has benzene opposite a cation-π acetylene and two unreacted isomers. 4j has a 3 + 1 coordination, and 4l has a four-coordinate structure that is nearly tetrahedral. As shown below, these unreacted isomers are more relevant for infrared spectroscopy. Infrared spectra were predicted by theory for each of the *n* = 1–6 isomers for comparison to the measured infrared patterns. The relative energies of these different isomers are presented in Table 1, with further details in the Supporting Information. The scaled (0.96 factor) harmonic frequencies are compared to the experimental spectra in the following figures. As shown in the Table, the bond dissociation energies for the *n* = 1–3 complexes are much higher than the infrared photon energy in the C–H stretching region (9–10 kcal/mol), whereas the binding energies are much lower for the *n* = 4 complexes. This is consistent with the fragmentation mass spectra in Figure 1.

**Table 1:** Energetics of Co^+^(C_2_H_2_)_*n*_ Complexes Resulting from Computations^a^

*n*	2S + 1	isomer	*E* (Hartree)	relative energy (kcal/mol)	BDE (kcal/mol)
0	1		–1382.456398	+16.8	
0	3		–1382.483220	+0.0	
0	5		–1382.456083	+17.0	
1	1	1a	–1459.867434	+16.5	46.0
1	3	1a	–1459.893768	+0.0	45.7
1	5	1a	–1459.831937	+38.8	24.0
2	3	2a	–1537.292467	+0.0	38.3
2	3	2b	–1537.289272	+2.0	36.3
2	3	2c	–1537.268488	+15.0	23.2
2	3	2d	–1537.261270	+19.6	18.7
3	3	3a	–1614.813851	+0.0	115.3
					58.6^b^
3	3	3b	–1614.667715	+91.7	23.6
3	3	3c	–1614.659935	+96.6	18.7
3	3	3d	–1614.659485	+96.9	18.4
3	3	3e	–1614.651585	+101.8	13.5
3	3	3f	–1614.647219	+104.6	10.7
4	3	4a	–1692.202361	+0.0	31.9
					44.8^b^
4	3	4j	–1692.002605	+125.3	0.1
4	3	4l	–1691.998233	+128.1	3.4
4	3	4n	–1691.995089	+130.1	–1.3

a Additional isomers and larger clusters are presented in the Supporting Information. Bond dissociation energies (BDE) are for the elimination of acetylene except as noted.

b Elimination of benzene.

**Figure 4:**
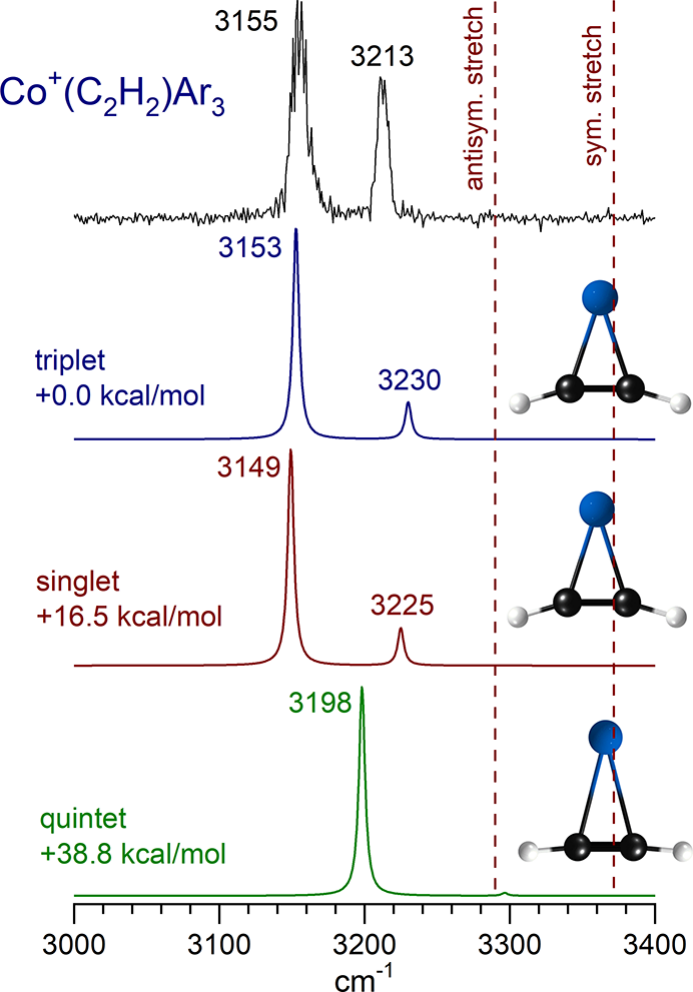
Infrared photodissociation spectrum measured for Co^+^(C_2_H_2_) tagged with three argon atoms compared to the spectra predicted by theory for different spin states of this ion. The theory shown is for the tag-free ion; computations show that the argons induce only very small shifts on the spectra (see Supporting Information).

Figure 4 shows the spectrum measured for the Co^+^(C_2_H_2_) ion tagged with three argon atoms compared with the spectra predicted by theory for the different spin states of this ion. Tagging with fewer argons than this did not produce a sufficient photodissociation yield. The experiment has two bands at 3155 and 3213 cm^–1^. The red dashed lines in the figure show the positions of the antisymmetric and symmetric stretches of acetylene at 3289 and 3374 cm^–1^, respectively. From this, it can be seen that the two experimental bands are likely from these same vibrations on acetylene, which are shifted to lower frequencies by the interaction with the metal ion. The symmetric stretch of acetylene is not IR-active, but as seen in previous studies, this vibration becomes active in the metal ion complex. The red shift is from the charge-transfer interaction between the metal ion and the ligand, as discussed above. Theory reproduces the red shift in the vibrations quite nicely, but both the triplet and singlet states have essentially the same IR pattern. Because the triplet is predicted to be much lower in energy, we assume that this explains the experiment. It is clear from the IR patterns that the quintet state is inconsistent with the experiment, and this state is also predicted to be even less stable. The spectra from the theory shown here and in subsequent cluster sizes are those for the tag-free ions; computations show that the argon induces only small shifts in these frequencies (see the Supporting Information). Likewise, the triplet states are lower in energy for each of the larger clusters except for some isomers of *n* = 5, which are more stable as singlets. Triplet states are able to explain the experimental spectra, as shown below. Spectra for other spin states are provided in the Supporting Information.

**Figure 5:**
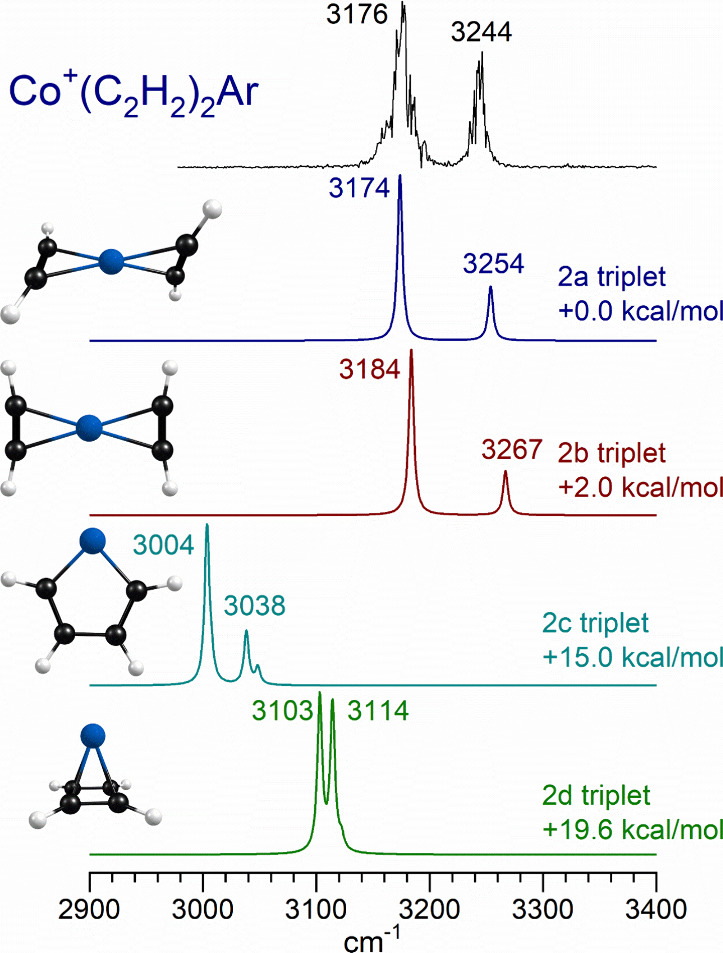
Infrared photodissociation spectrum measured for Co^+^(C_2_H_2_)_2_ tagged with one argon atom compared to the spectra predicted by theory for different isomers of this ion.

**Figure 6:**
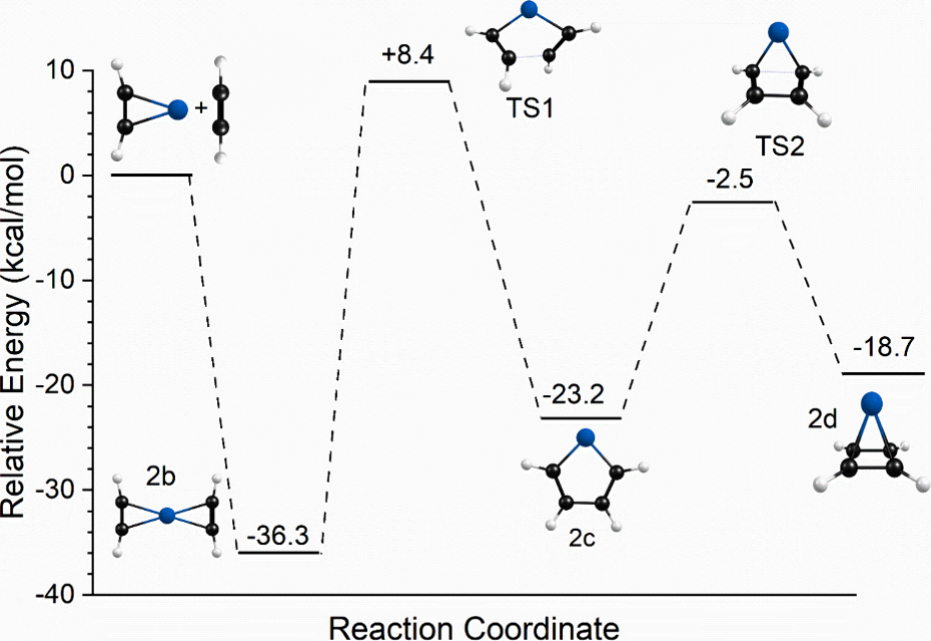
Reaction coordinate for the Co^+^(C_2_H_2_)_2_ ion resulting from DFT computations.

Figure 5 shows the infrared photodissociation spectra measured for the Co^+^(C_2_H_2_)_2_ ion tagged with argon compared to the spectra predicted by theory for the different possible isomers of this ion. Two experimental bands are found at 3176 and 3244 cm^–1^. As indicated, there are two “unreacted” isomers at low energy with the two acetylenes in cation-π binding positions opposite each other (isomers 2a and 2b). These isomers differ only in the orientation of the ligands. The *D*_2d_ structure lies slightly lower in energy than the *D*_2h_ structure. Higher energy reacted structures correspond to a metallacycle (isomer 2c) and a metal-cyclobutadienyl structure (isomer 2d). A metallacycle structure like this has been detected in our previous study on V^+^ and Ti^+^ clusters with acetylene,^35,42^ and also in the work of Bakker and co-workers on Ta^+^ complexes with ethylene.^72^ As shown in the figure, the spectra for either isomer 2a or 2b are consistent with the experimental spectrum. However, the spectra for reacted isomers 2c and 2d are inconsistent with the experiment. We can therefore safely conclude that this complex forms only unreacted cation-π complexes, although we cannot determine the orientation of the ligands. To further investigate the reactivity of this cluster, we performed additional computations to document its reaction coordinate. As shown in Figure 6, not only is the reaction endothermic, but there is an additional activation energy for any ligand coupling reactions. It is therefore understandable that we detect only the unreacted complexes.

**Figure 7:**
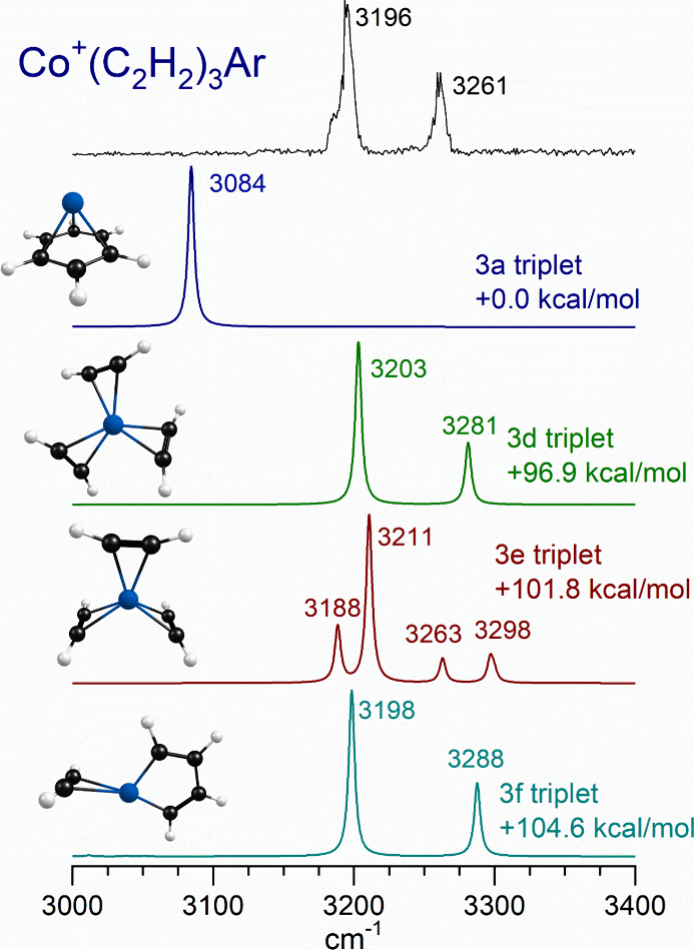
Infrared photodissociation spectrum measured for Co^+^(C_2_H_2_)_3_ tagged with argon, compared to the spectra predicted by theory for different isomers and of this ion.

Figure 7 shows the spectra measured for the Co^+^(C_2_H_2_)_3_ ion tagged with argon compared to the spectra predicted by theory for the different possible isomers of this ion. This cluster size is one that could conceivably form a metal-benzene complex via a cyclo-addition reaction, and the resulting structure (isomer 3a) is the most stable. The energetics in the figure are, therefore, relative to this. Other structures include two different unreacted cation-π complexes (isomers 3d and 3e) with three individual acetylene ligands as well as two reacted structures with cyclobutadienyl rings and one with a metallacycle opposite a single cation-π ligand (isomer 3f). Isomers similar to these have been identified for the transition metal ion-acetylene complexes studied previously.^32−39,41,42^ In the case of the V^+^(C_2_H_2_)_3_ complex, the metallacycle intermediate and the V^+^(benzene) product ion were both detected via their IR spectra.^35^

As shown in Figure 2, the spectrum for the *n* = 3 complex looks almost identical to those for the *n* = 1 and 2 complexes, with two bands at 3196 and 3261 cm^–1^. The pattern for the bands for the *n* = 1–3 is very similar, but the red shift is progressively smaller for the larger clusters. This is to be expected as the charge-transfer interaction is distributed over more ligands. The metallacycle structure (isomer 3f) has a spectrum similar to those of the unreacted isomers, but this structure is predicted to be much less stable. It is clear that the metal-benzene structure does not form under these conditions since its single band would be at much lower frequency. There is nothing in the experimental spectrum near the band predicted for this structure at 3084 cm^–1^. It should be noted that the IR intensity for the single Co^+^(benzene) band (19.5 km/mol) is much smaller than those for the unreacted structures (e.g., 302.6 and 150.6 km/mol for isomer 3d), making it harder to detect. We therefore cannot rule out the formation of this isomer completely but can conclude that its concentration, if any, is significantly smaller than that of the unreacted isomer.

**Figure 8:**
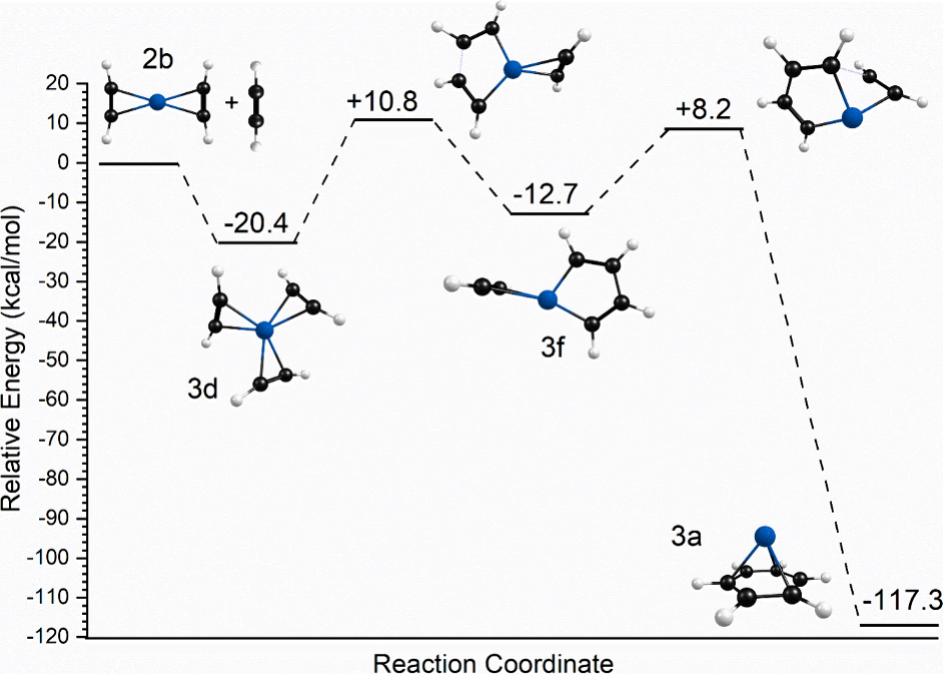
Reaction coordinate for the Co^+^(C_2_H_2_)_3_ ion resulting from DFT computations.

To further investigate the *n* = 3 complex and its potential reactions, we attempted to compute its reaction coordinate, which is presented in Figure 8. The energetics here are relative to those of the Co^+^(C_2_H_2_)_2_ (structure 2b) + C_2_H_2_ reactants. However, despite extensive computations, we were unable to locate a well-behaved transition state connecting the unreacted cation-π complex (3d) and an initial ligand coupling product (structure 3f) with the metallacycle structure. Instead, we located a second-order saddle point structure between these, with two imaginary frequencies. This structure, which is shown in Figure 8, is much higher in energy than the starting structure, suggesting that there is indeed a significant barrier to this coupling reaction, but we cannot confirm its exact value. A barrier here in the reaction path is consistent with our observation of the unreacted species in the infrared experiment. The same kind of reaction barrier was found previously in our studies of Fe^+^ and Pt^+^ complexes with acetylene, where reactions were also not detected.^39,41^ A smaller barrier was found in the case of V^+^(C_2_H_2_)_*n*_ complexes, where reactions were detected but only warmer ions reacted.^35^ As an additional consideration here, isomer 3f lies at higher energy than isomer 3d, and so the formation of this intermediate would be endothermic. Likewise, there is another significant barrier to form the transition state (well-behaved) between structures 3f and 3a. Therefore, it is understandable that we do not form a benzene complex.

**Figure 9:**
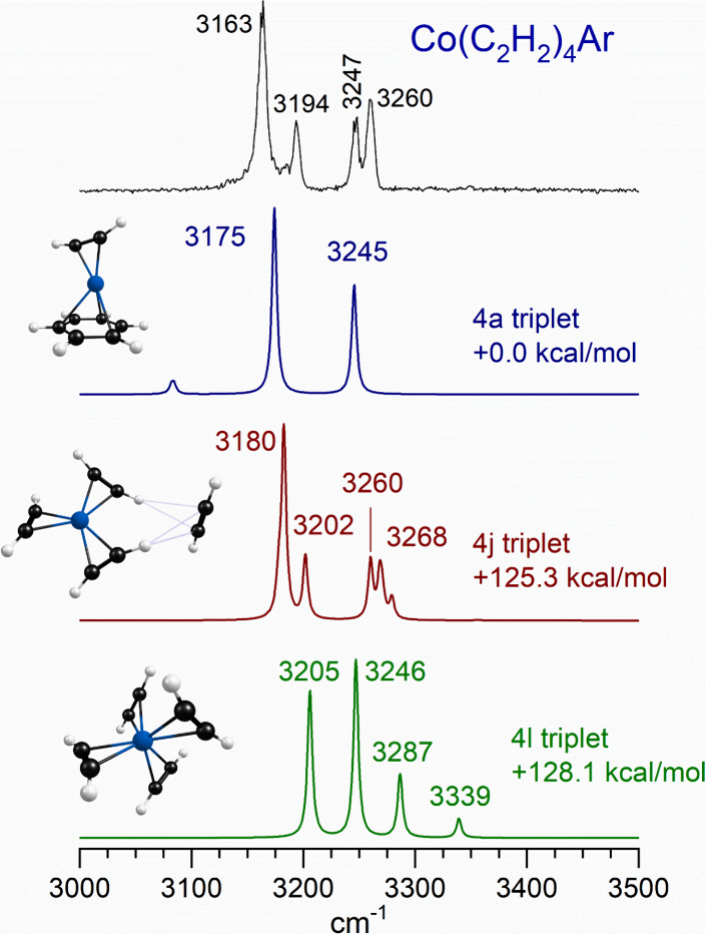
Infrared photodissociation spectrum measured for Co^+^(C_2_H_2_)_4_ tagged with argon, compared to the spectra predicted by theory for different isomers and of this ion.

Figure 9 presents the infrared photodissociation spectrum for the *n* = 4 complex compared to the spectra predicted by theory for selected isomers. As shown here and in Figure 2, a significant change occurred in the spectrum compared to those of smaller complexes. The main features in the spectrum are two doublets at 3163/3194 and 3247/3260 cm^–1^. Significantly, the higher frequency bands in these two doublets are located almost exactly at the positions of the bands in the *n* = 3 spectrum, suggesting that they have the same origin, i.e., the antisymmetric (3194 cm^–1^) and symmetric (3260 cm^–1^) stretches of acetylene molecules coordinated to the metal ion. The new bands must arise from some other structural configuration. This suggests that the *n* = 4 spectrum is that of a solvated *n* = 3 species, i.e., a ″3 + 1″ complex, with one external molecule not coordinated to the metal. We have shown in previous work that such an external molecule binds in a CH−π configuration with the coordinated ligands, and then the C–H stretches of the internal “donor” molecule become more intense and have a greater frequency shift to the red.^32,33,37,39,41^ This explains the lower frequency bands in the two doublets. The 3163 and 3247 cm^–1^ bands are therefore the antisymmetric and symmetric stretches of the donor ligands involved in the CH−π bond. Although the agreement is not perfect, isomer 4j has the structural pattern and predicted spectrum for this kind of 3 + 1 complex and is therefore the best assignment for the structure of this ion. A similar pattern of doublet bands was observed previously for Cu^+^(C_2_H_2_)_4_, which formed unreactive three-coordinate complexes in such a 3 + 1 configuration.^32^

**Figure 10:**
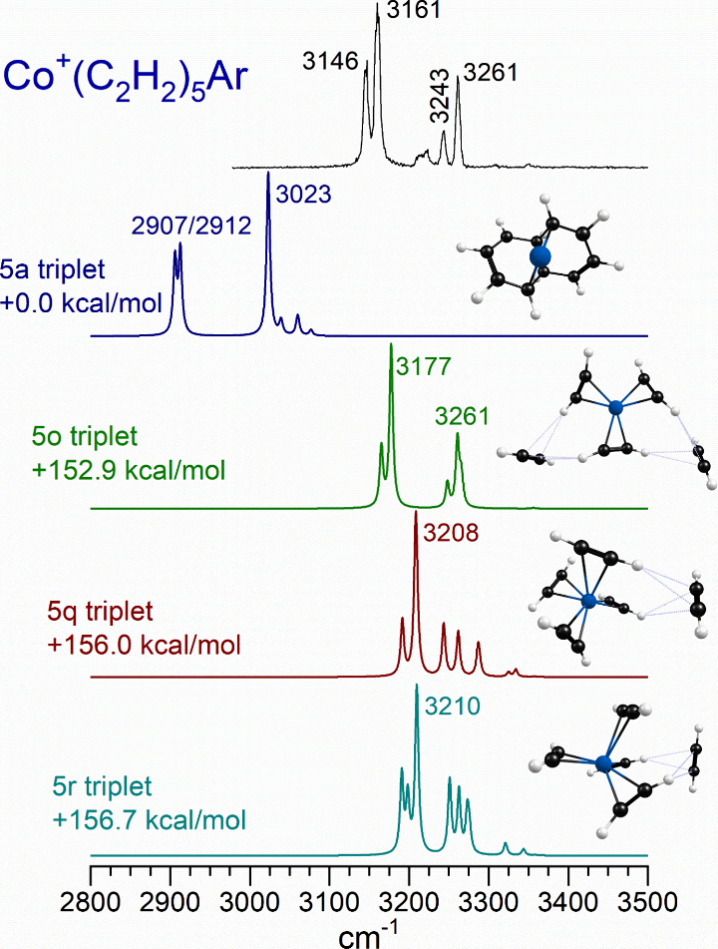
Infrared photodissociation spectrum measured for Co^+^(C_2_H_2_)_5_ tagged with argon, compared to the spectra predicted by theory for different isomers and of this ion.

The spectrum for the *n* = 5 complex has features very similar to those for the *n* = 4 complex, i.e., a pair of doublet bands in the C–H stretching region. Its spectrum is compared with the predictions of theory for different isomers in Figure 10. The most stable isomer predicted by theory for this ion is the metal-naphthalene complex, and the energies presented are relative to this. This structure can be ruled out immediately because its strongly red-shifted bands are not detected. Instead, the bands in the spectrum fall in the region of unreacted complexes with one or two external solvating acetylene ligands. The pattern of bands with a doublet at 3146/3161 cm^–1^ and another at 3243/3261 cm^–1^ looks similar to that for the *n* = 4 complex. 3 + 2 structures (i.e., three ligands coordinated to metal and two external to this) are computed to lie close in energy to 4 + 1 structures. Building on the structures already determined for the *n* = 3 and 4 complexes, the most likely structure is the 3 + 2 configuration, with a three-coordinate core and two solvating molecules. Isomer 5o has such a structure, and its predicted infrared spectrum matches the experiment reasonably well. It has the two main doublets seen but not the additional weak band at 3216 cm^–1^, which could come from a small concentration of another isomer. Therefore, this 5o structure is the most likely one for this ion. As an additional note, some of the *n* = 5 isomers are more stable in the singlet spin state (see Supporting Information). Isomer 5o, which matches the experimental spectrum, is more stable in the triplet state.

**Figure 11:**
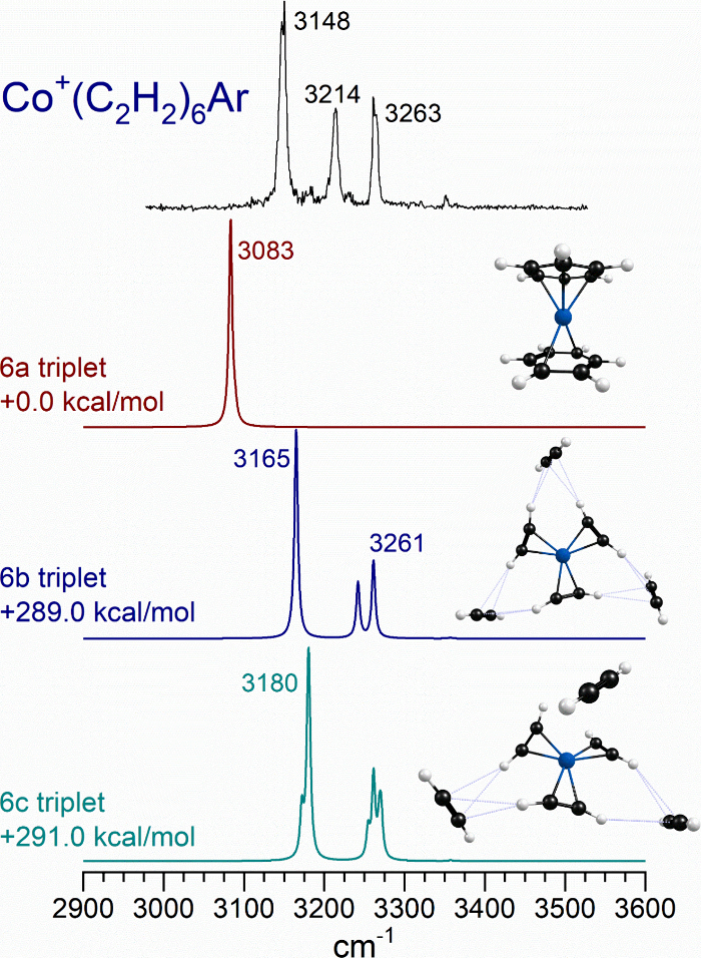
Infrared photodissociation spectrum measured for Co^+^(C_2_H_2_)_6_ tagged with argon, compared to the spectra predicted by theory for different isomers and of this ion.

Figure 11 shows the spectrum measured for the *n* = 6 complex compared to the predictions of theory for three of the lower energy isomers. The measured bands fall in the same frequency range as those detected for the *n* = 4 and 5 complexes, but the pattern has changed to that of three roughly equally spaced bands. The most stable structure for this ion is the metal dibenzene sandwich, whose single band at lower frequency does not match the experiment. Again, if we assume that we have correctly identified the *n* = 5 structure, the most likely *n* = 6 structure is one adding a single external acetylene to this, producing a 3 + 3 structure like isomer 6b. This has three core acetylenes coordinated directly to the metal ion and three external ones bound via CH−π bonds to the core ligands. The intense IR bands are those for the core ligands involved as donors for the CH−π bonds. Although the spacings predicted for this isomer do not exactly match those in the spectrum, the three bands predicted and measured seem to indicate that this is the isomer produced.

To summarize, the fragmentation patterns complemented by theory for these Co^+^(C_2_H_2_)_*n*_ complexes are consistent with strong bonding for the first three ligands and weaker bonding for subsequent ligands. The infrared patterns are all consistent with unreacted structures having intact acetylene ligands coordinated to the metal and clustering around it. The coordination number of three ligands is similar to that seen for copper, silver, platinum, and gold cations, as is the second-sphere clustering via additional ligands bound by cation-π interactions. Unreacted structures lie lower in energy than reacted ones for the *n* = 2 complex, but in larger clusters, the formation of benzene or naphthalene structures is exothermic. However, activation barriers apparently inhibit the occurrence of such reactions under the cold conditions of our supersonic molecular beams.

Takayanagi and co-workers have performed computational studies to investigate the activation barriers to metal-catalyzed cycloaddition reactions of acetylene for several transition metal ions.^23−25^ They found that the activation barriers are lower for early transition metals or for later transition metals with an electron-withdrawing ligand attached. Further experimental studies of such systems are therefore warranted.

## Conclusions

Co^+^(C_2_H_2_)_*n*_ complexes produced by laser ablation in a supersonic beam source were studied with infrared photodissociation spectroscopy and computational chemistry by using density functional theory. Fragmentation channels and infrared spectroscopy are consistent in indicating that the coordination around Co^+^ is filled with three acetylene ligands bound in cation-π configurations. Additional ligands cluster around this three-coordinate core via CH−π bonding interactions. Although theory indicates that cyclization chemistry is exothermic to form benzene or naphthalene structures in larger clusters, these reaction products are not detected. Studies of the reaction coordinates indicate that activation barriers inhibit such reactions. This result is somewhat surprising, since other studies of the reactions of cobalt cations with acetylene suggested that cyclization chemistry did occur.^15,22^ However, the cold supersonic beam conditions may produce different chemical outcomes for metal ion-acetylene interactions than higher temperature environments, where such reactions have been studied previously. Additionally, previous studies suggesting the occurrence of such chemistry did not have spectroscopic evidence of the products. Future studies will investigate this chemistry for early transition metals, where activation barriers are predicted to be lower.

## Supporting Information Available

Full citations for refs (59,64) and the details of the DFT computations done, including the structures and energetics for each of the ions considered (PDF)
