# Supplementary material for: Co+(C2H2)n Complexes Studied with Selected-Ion Infrared Spectroscopy and Theory
Source: J Phys Chem A. 2024 Oct 7;128(41):8954–63. doi: 10.1021/acs.jpca.4c05304 (PMC11492291; doi:10.1021/acs.jpca.4c05304)
Supplement: Supplementary file 1 — jp4c05304_si_001.pdf [file jp4c05304_si_001.pdf]

Supporting Information

$Co^+(C_2H_2)_n$ Complexes Studied with Selected-Ion Infrared Spectroscopy and Theory

Anna G. Batchelor,[1] Joshua H. Marks,[1] Timothy B. Ward,[1] and Michael A. Duncan[1]*

[1]Department of Chemistry, University of Georgia, Athens, GA 30602

*Email: maduncan@uga.edu

Table of Contents

Full citations for refs. 59 and 64. S3

Figure S1: Mass spectrum of $Co^+(C_2H_2)_n$ S4

(C_2H_2)
Table S1: energy and Figure S2: structure S5 – S6

Co^+
Table S2: relative energies S7

$Co^+(C_2H_2)$ & $Co^+(C_2H_2)Ar_2$
Tables S3 – S4: relative energies S8 – S9
Figures S3 – S11: cartesian coordinates & unscaled vibrational frequencies S10 – S18
Figures S12 – S14: simulated spectra S19 – S21

$Co^+(C_2H_2)_2$
Table S5: relative energies S22
Figures S15 – S28: cartesian coordinates & unscaled frequencies S23 – S36
Figures S29 – S31: simulated spectra S37 – S39

$Co^+(C_2H_2)_3$
Table S6: relative energies S40
Figure S32: diagram of isomers S41
Tables S7 – S25: cartesian coordinates & unscaled vibrational frequencies S42 – S60
Figures S33 – S34: cartesian coordinates & unscaled vibrational frequencie S61 – S62
Figures S35 – S40: simulated spectra S63 – S68

$Co^+(C_2H_2)_4$
Table S26: relative energies S69
Tables S27 – S67: cartesian coordinates & unscaled vibrational frequencies S70 – S110
Figures S41 – S48: simulated spectra S111 – S118

$Co^+(C_2H_2)_5$
Table S68: relative energies S119 – S120
Tables S69 – S188: cartesian coordinates & unscaled vibrational frequencies S121 – S240
Figures S49 – S59 : simulated spectra S241 – S251

$Co^+(C_2H_2)_6$
Table S189: relative energies S252
Tables S190 – S199: cartesian coordinates & unscaled vibrational frequencies S253 – S262
Figures S60 – S61: simulated spectra S263 – S264

Full citation for reference 59:

Akin, S. T.; Zamudio-Bayer, V.; Duanmu, K.; Leistner, G.; Hirsch, K.; Bülow, C.; Ławicki, A.; Terasaki, A.; von Issendorff, B.; Truhlar, D. G.; Lau, J. T.; Duncan, M. A. Size-Dependent Ligand Quenching of Ferromagnetism in $Co_3(benzene)_n^+$ Clusters Studied with X-ray Magnetic Circular Dichroism. *J. Phys. Chem. Lett.* **2016**, *7*, 4568–4575.

Full citation for reference 64:

Frisch, M. J.; Trucks, G. W.; Schlegel, H. B.; Scuseria, G. E.; Robb, M. A.; Cheeseman, J. R.; Scalmani, G.; Barone, V.; Petersson, G. A.; Nakatsuji, H.; Li, X.; Caricato, M.; Marenich, A. V.; Bloino, J.; Janesko, B. G.; Gomperts, R.; Mennucci, B.; Hratchian, H. P.; Ortiz, J. V.; Izmaylov, A. F.; Sonnenberg, J. L.; Williams-Young, D.; Ding, F.; Lipparini, F.; Egidi, F.; Goings, J.; Peng, B.; Petrone, A.; Henderson, T.; Ranasinghe, D.; Zakrzewski, V. G.; Gao, J.; Rega, N.; Zheng, G.; Liang, W.; Hada, M.; Ehara, M.; Toyota, K.; Fukuda, R.; Hasegawa, J.; Ishida, M.; Nakajima, T.; Honda, Y.; Kitao, O.; Nakai, H.; Vreven, T.; Throssell, K.; Montgomery, J. A., Jr.; Peralta, J. E.; Ogliaro, F.; Bearpark, M. J.; Heyd, J. J.; Brothers, E. N.; Kudin, K. N.; Staroverov, V. N.; Keith, T. A.; Kobayashi, R.; Normand, J.; Raghavachari, K.; Rendell, A. P.; Burant, J. C.; Iyengar, S. S.; Tomasi, J.; Cossi, M.; Millam, J. M.; Klene, M.; Adamo, C.; Cammi, R.; Ochterski, J. W.; Martin, R. L.; Morokuma, K.; Farkas, O.; Foresman, J. B.; Fox, D. J. Gaussian 16 (Revision C.01), Gaussian, Inc., Wallingford CT, 2009.

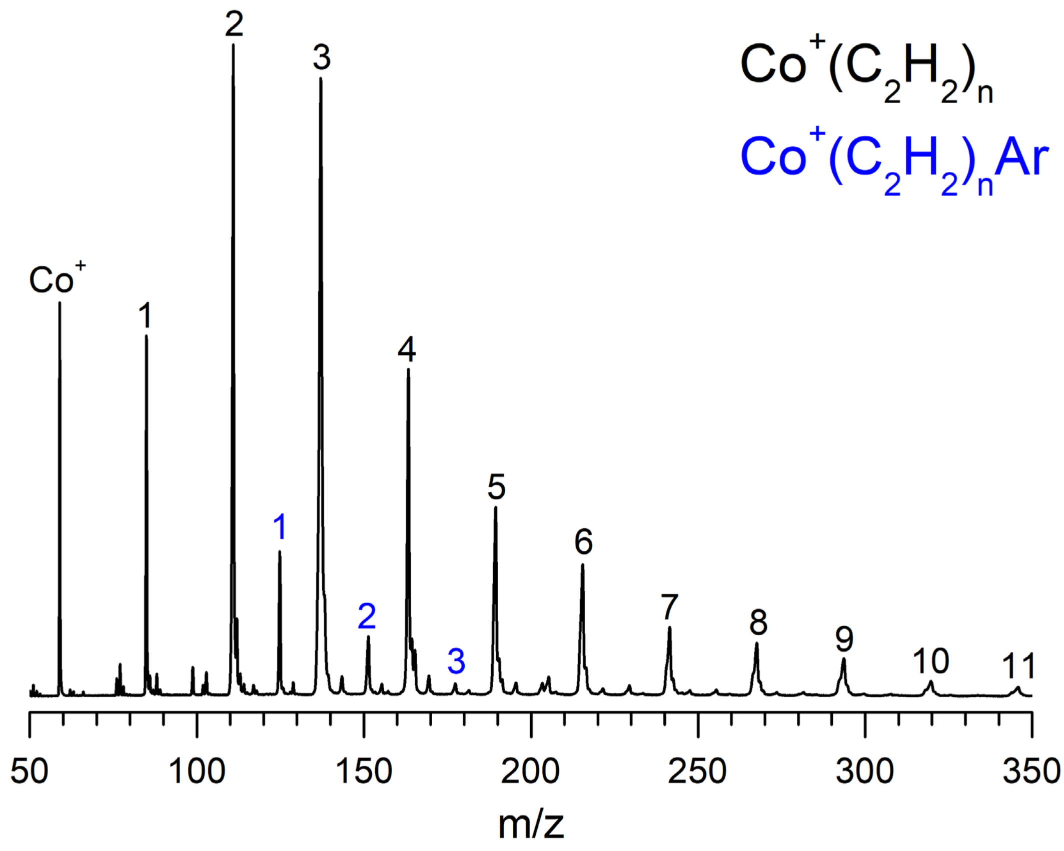

Figure S1. Mass spectrum of $Co^+(C_2H_2)_n$ and $Co^+(C_2H_2)_nAr$ ions produced via laser vaporization of a cobalt rod in a supersonic expansion of argon seeded with acetylene.

Table S1. C_2H_2 calculated at the B3LYP/Def2TZVP level of theory using Gaussian16.

2s + 1	E (hartree)	Relative E (kcal/mol)
1	-77.337670	+0.0

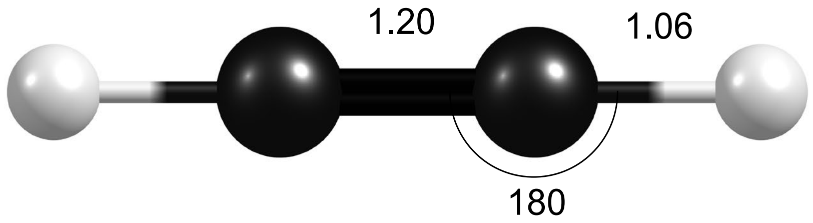

Figure S2. The optimized geometry of C_2H_2 followed by its predicted frequencies (cm^{-1}) and IR intensities (km/mol).

Frequency (cm^{-1})	Intensity (km/mol)
620.1749	0
620.1749	0
764.564	108.0076
764.564	108.0076
2072.3708	0
3417.374	90.6378
3515.5372	0

Table S2. Co^+ electronic energy calculated at the B3LYP/def2TZVP level.

2s + 1	E (hartree)	Relative E (kcal/mol)
1	-1382.456398	+16.8
3	-1382.483220	+0.0
5	-1382.456083	+17.0

Table S3. $Co^+(C_2H_2)$ electronic energy calculated at the B3LYP/Def2TZVP level.

2s + 1	E (hartree)	Relative E (kcal/mol)	C_2H_2 BE (kcal/mol)
1	-1459.867434	+16.5	46.0
3	-1459.893768	+0.0	45.7
5	-1459.831937	+38.8	24.0

Table S4. $Co^+(C_2H_2)Ar_3$ electronic energy calculated at the B3LYP/Def2TZVP level.

Isomer	2s + 1	E (hartree)	Relative E (kcal/mol)	Ar BE (kcal/mol)
1a	1	-3042.543038	+0.0	2.5
1b	1	-3042.539911	+2.0	0.5
1c	1	-3042.535764	+4.6	-2.1
1a	3	-3042.568805	+0.0	2.2
1b	3	-3042.566143	+1.7	0.5
1c	3	-3042.562183	+4.2	-2.0

Figure S3. The optimized geometry of singlet $Co^+(C_2H_2)$ followed by its predicted frequencies (cm^{-1}) and IR intensities (km/mol).

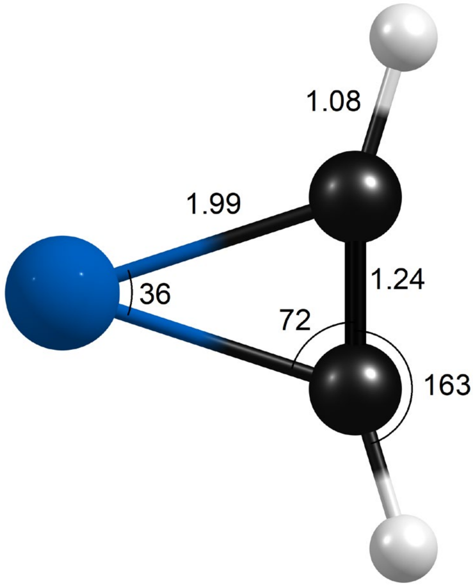

Frequency (cm^{-1})	Intensity (km/mol)
350.2654	0.386
412.8363	0.511
662.9494	0
745.4544	132.9705
746.382	71.4967
777.2597	14.6749
1856.0109	0.5357
3280.3958	204.3216
3359.242	35.4205

Figure S4. The optimized geometry of triplet $Co^+(C_2H_2)$ followed by its predicted frequencies (cm^{-1}) and IR intensities (km/mol).

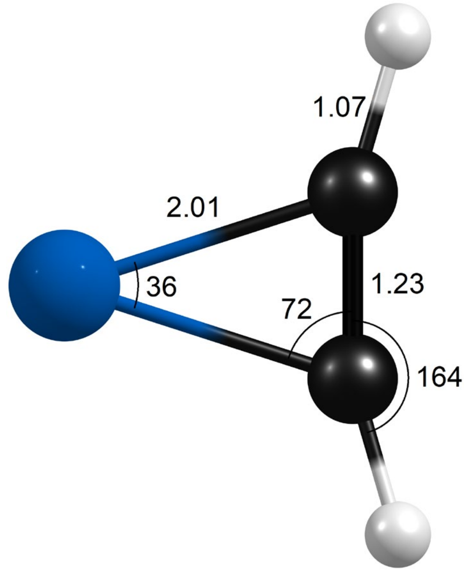

Frequency (cm^{-1})	Intensity (km/mol)
359.6565	0.8207
395.1802	0.9083
654.5931	0
739.201	69.1065
752.5205	124.5949
778.6347	12.4431
1870.2945	0.1069
3284.103	206.0014
3364.5999	36.1285

Figure S5. The optimized geometry of quintet $Co^+(C_2H_2)$ followed by its predicted frequencies (cm^{-1}) and IR intensities (km/mol).

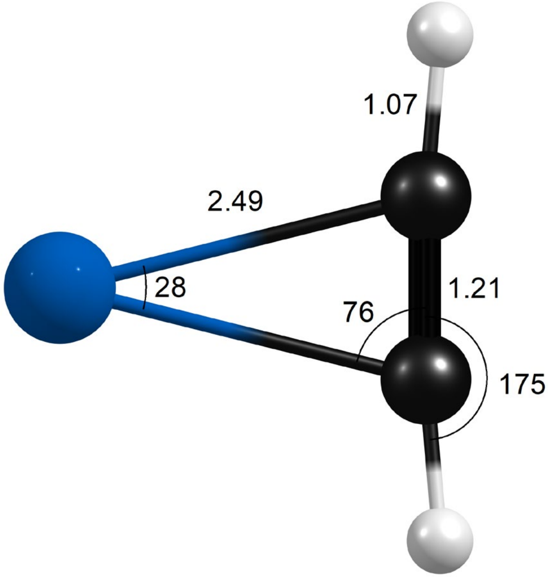

Frequency (cm^{-1})	Intensity (km/mol)
83.9917	7.5685
186.7299	27.1807
625.4695	0.0203
690.0153	4.7822
783.301	90.1802
857.7126	128.1977
2016.6671	57.2292
3331.4102	241.9843
3433.7149	3.4281

Figure S6. The optimized geometry of 1a-singlet $Co^{+}(C_2H_2)Ar_3$ followed by its predicted frequencies (cm^{-1}) and IR intensities (km/mol).

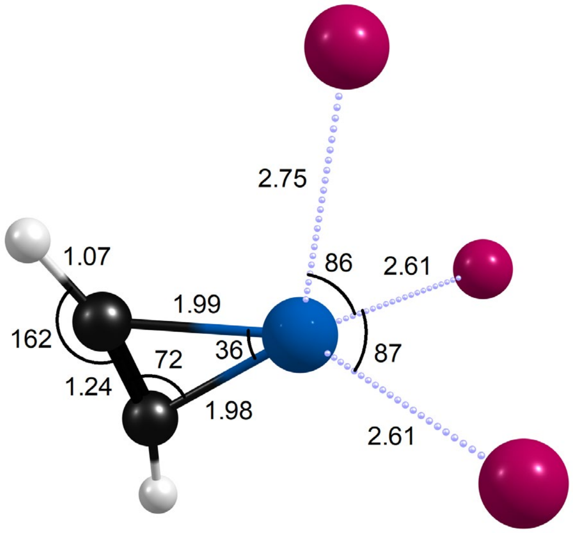

Frequency (cm^{-1})	Intensity (km/mol)
42.68	0.0967
48.2393	0.1358
50.2549	0.3051
65.0339	2.6493
69.7312	3.5317
78.9615	0.4033
84.9146	2.411
97.9785	8.363
126.7155	8.8658
349.1768	1.3756
432.3987	0.051
679.0001	0.0555
747.3978	60.0034
759.0856	89.5897
768.4251	7.2121
1855.1543	3.0501
3294.1347	144.8538
3369.9293	46.1983

Figure S7. The optimized geometry of 1b-singlet $Co^{+}(C_2H_2)Ar_3$ followed by its predicted frequencies (cm^{-1}) and IR intensities (km/mol).

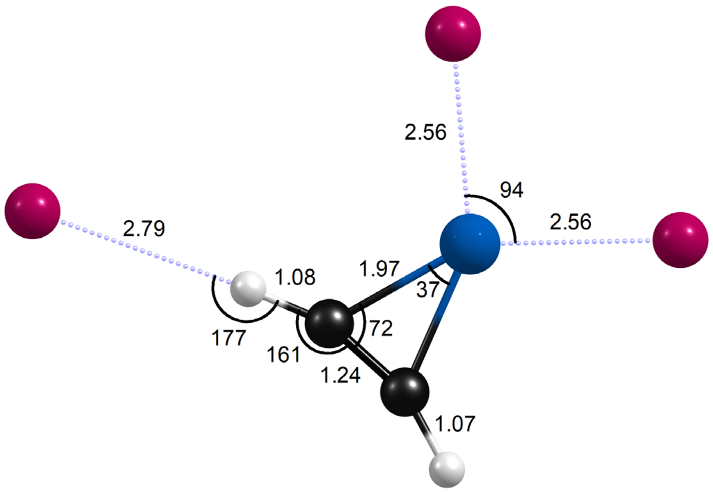

Frequency (cm^{-1})	Intensity (km/mol)
10.2382	0.7666
10.6605	0.5914
32.0967	2.8472
46.9115	0.7865
52.0157	0.3322
61.0162	0.0378
72.4375	0.1562
120.4371	12.6405
134.3422	9.9608
379.8585	0.2584
457.7633	0.0118
690.8156	4.4924
758.1639	87.2676
766.3197	63.0107
782.0085	11.5658
1839.6884	1.1319
3279.4077	229.5116
3355.8461	59.5414

Figure S8. The optimized geometry of singlet-1c $Co^{+}(C_2H_2)Ar_3$ followed by its predicted frequencies (cm^{-1}) and IR intensities (km/mol).

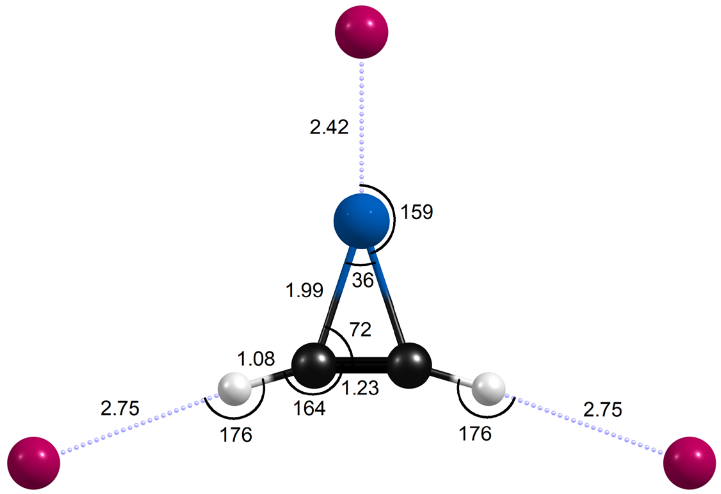

Frequency (cm^{-1})	Intensity (km/mol)
9.0793	1.6215
13.0047	1.7619
13.3335	0.0309
34.4939	1.0182
44.5448	5.3872
49.1535	0.4143
62.3221	0.8367
75.7462	0.3016
171.2473	8.9855
355.8343	0.8596
420.1405	2.5486
684.0398	0.0879
753.5491	55.9069
769.2711	82.363
790.4271	3.3015
1866.0456	0.1082
3267.808	375.2882
3350.0886	78.5079

Figure S9. The optimized geometry of triplet-1a $Co^{+}(C_2H_2)Ar_3$ followed by its predicted frequencies (cm^{-1}) and IR intensities (km/mol).

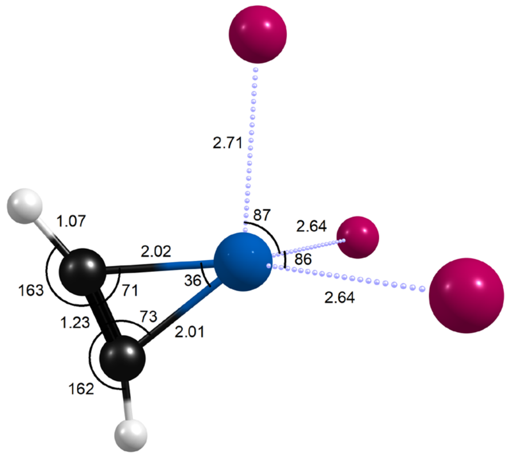

Frequency (cm^{-1})	Intensity (km/mol)
41.37	0.0871
46.9844	0.6107
49.3842	0.3164
63.6436	1.059
74.6351	2.7653
83.8618	0.499
87.7735	5.0589
92.1542	7.7486
124.6248	8.7421
353.4676	2.4178
411.1892	0.315
667.3768	0.1469
736.6532	58.6244
755.9515	93.3221
768.0024	4.6011
1873.4451	5.5358
3298.9817	148.8295
3377.0746	47.67

Figure S10. The optimized geometry of triplet-1b $Co^+(C_2H_2)Ar_3$ followed by its predicted frequencies (cm^{-1}) and IR intensities (km/mol).

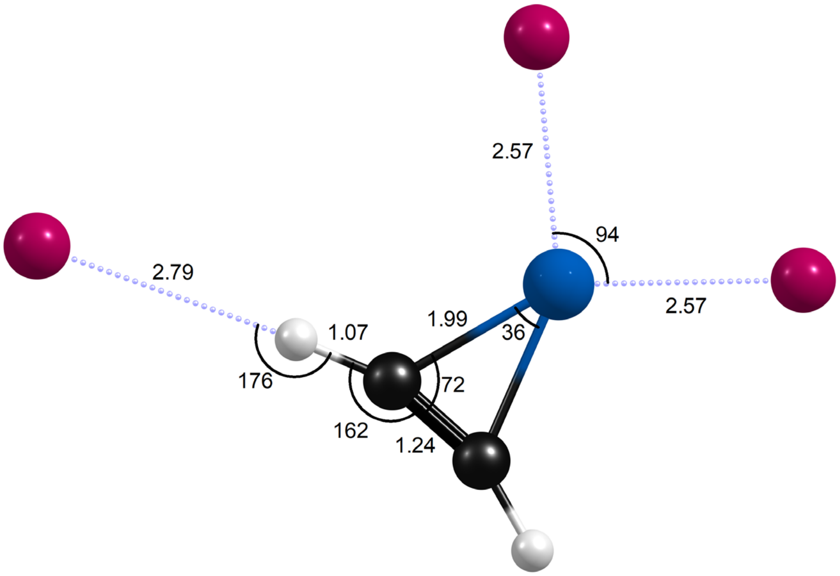

Frequency (cm^{-1})	Intensity (km/mol)
10.1953	0.6721
11.3753	0.721
26.6276	2.7049
44.2474	0.4629
46.9445	0.8903
70.9523	0.0048
75.9284	0.238
120.2451	12.8091
133.8678	10.3634
390.6017	0.8185
438.3794	0.0354
682.2698	3.5765
757.1074	67.1752
762.4208	84.2479
781.5046	4.6763
1856.3371	2.6012
3283.4128	232.3742
3361.6912	61.5232

Figure S11. The optimized geometry of triplet-1c $Co^{+}(C_2H_2)Ar_3$ followed by its predicted frequencies (cm^{-1}) and IR intensities (km/mol).

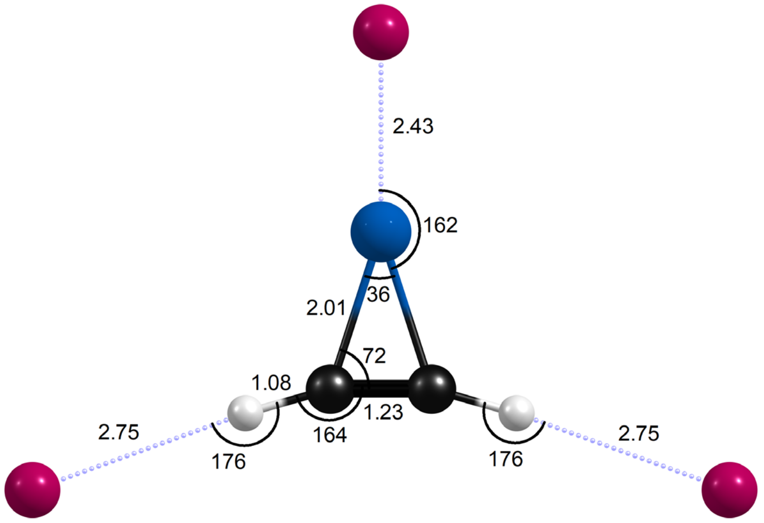

Frequency (cm^{-1})	Intensity (km/mol)
9.0084	1.6617
10.2251	2.2769
12.8821	0.0351
16.4723	0.3929
34.3914	1.0061
45.4018	5.8051
60.4798	0
69.8638	0.6843
169.7109	8.787
374.4599	1.1975
406.0799	2.9703
677.1297	0
748.2232	54.3959
770.8929	86.8387
791.802	0.7593
1877.982	0.0104
3270.5033	378.3404
3354.2082	79.5971

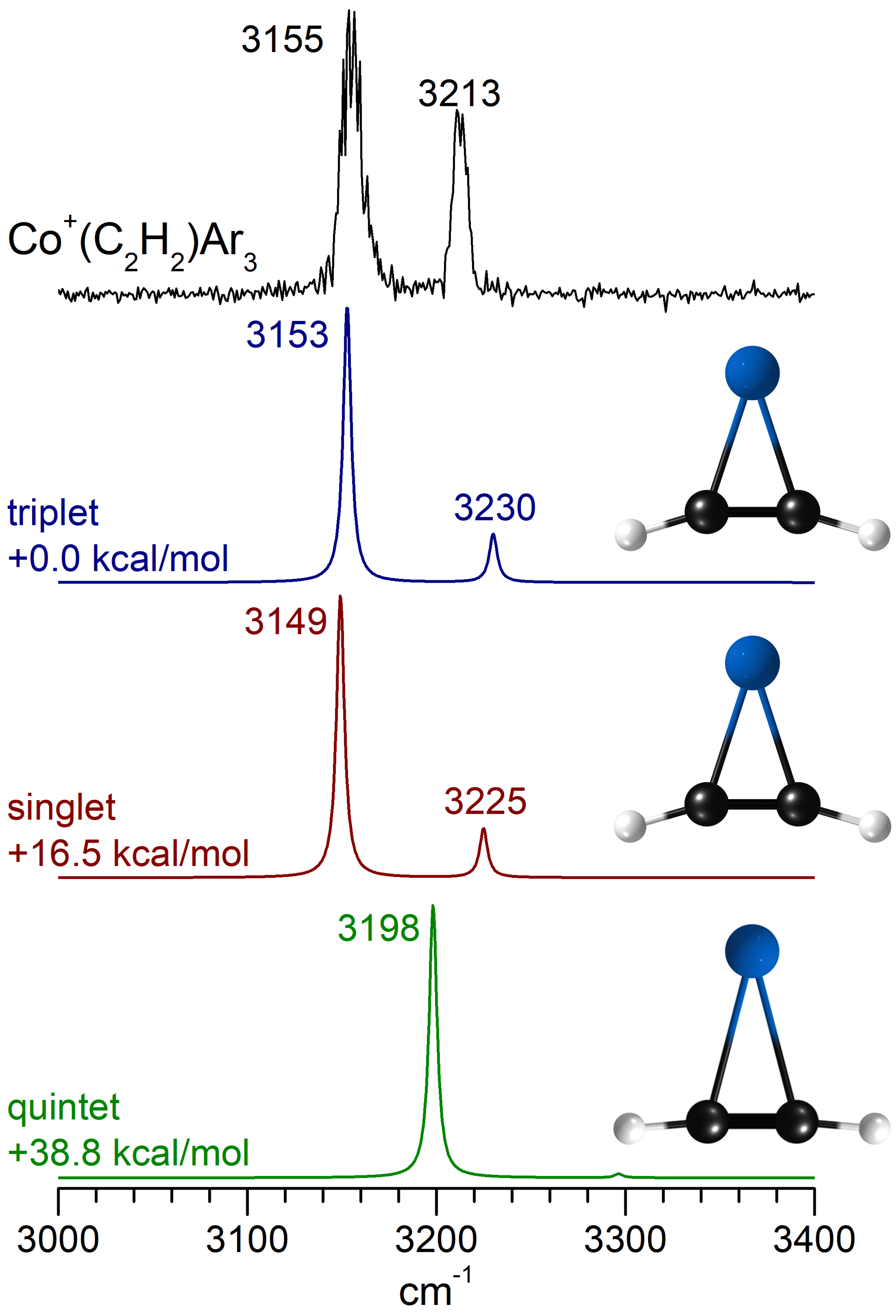

Figure S12. The experimental spectrum for $Co^{+}(C_2H_2)Ar_3$ with simulated spectra for $Co^{+}(C_2H_2)$ as a singlet, triplet, and quintet.

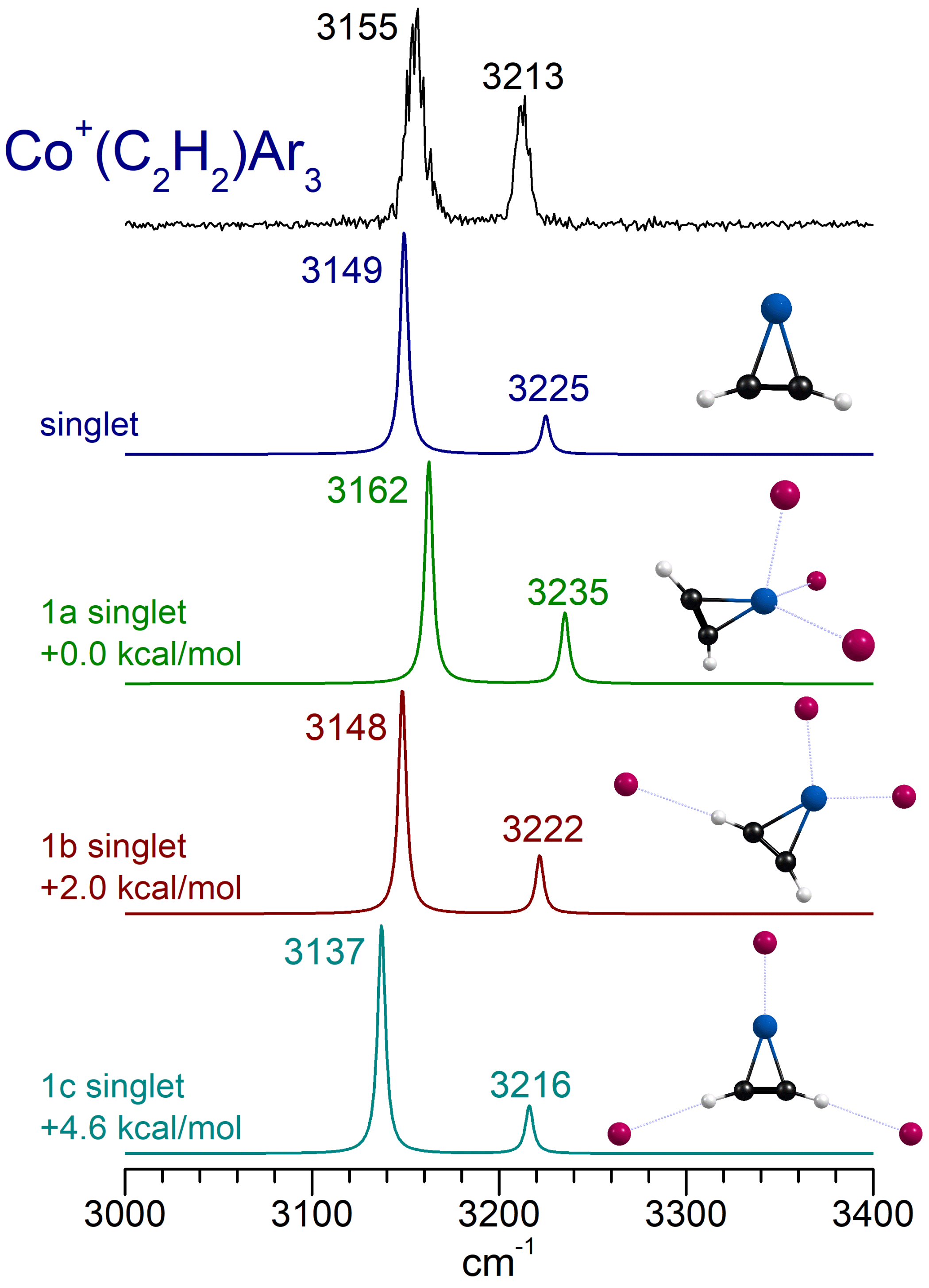

Figure S13. The experimental spectrum for $Co^{+}(C_2H_2)Ar_3$ with simulated spectra for $Co^{+}(C_2H_2)$ as a singlet and all predicted isomers of singlet-$Co^{+}(C_2H_2)Ar_3$.

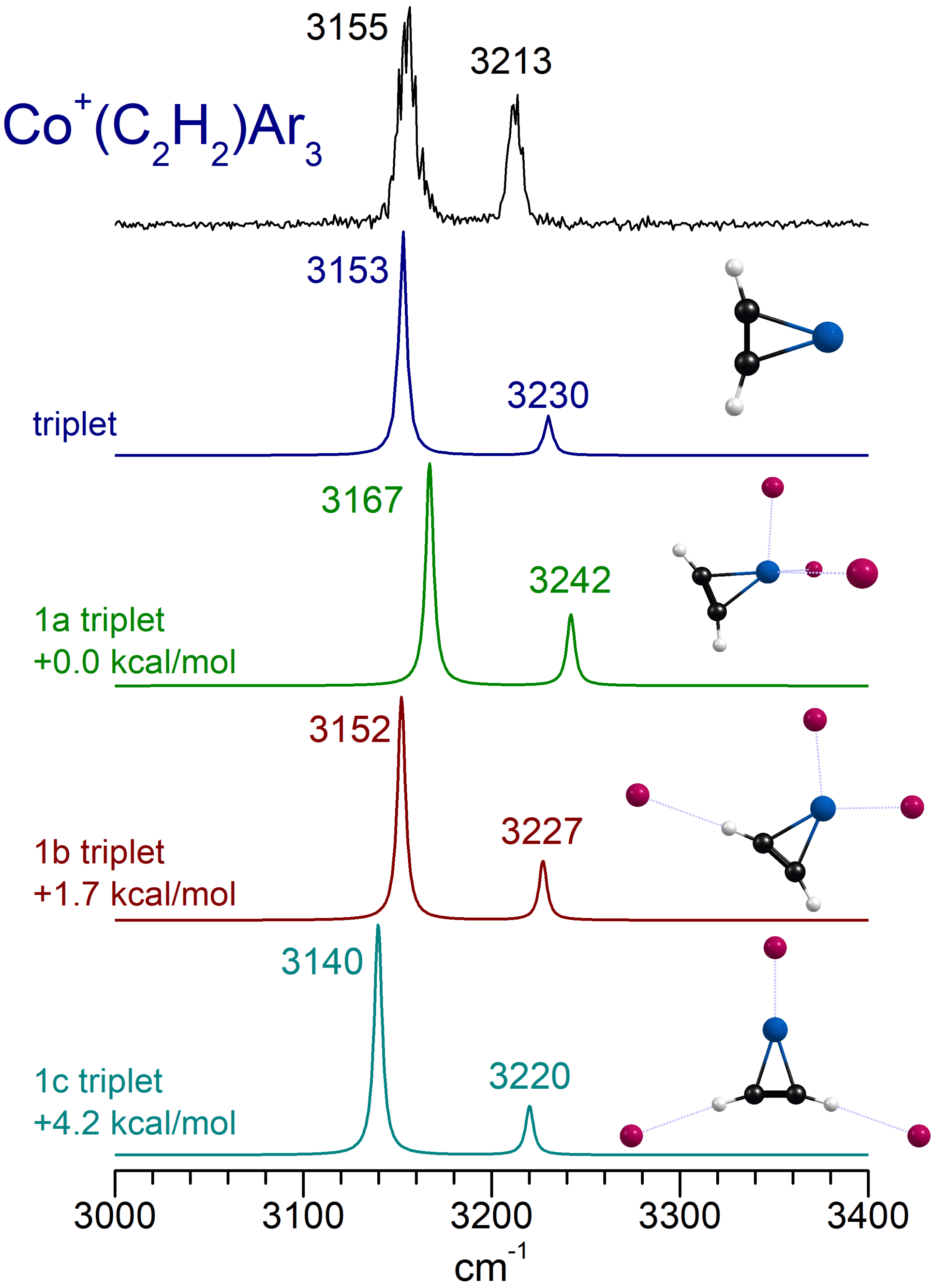

Figure S14. The experimental spectrum for $Co^+(C_2H_2)Ar_3$ with simulated spectra for $Co^+(C_2H_2)$ as a triplet and all predicted isomers of triplet-$Co^+(C_2H_2)Ar_3$.

Table S5. $Co^+(C_2H_2)_2$ calculated at the B3LYP/Def2TZVP level of theory using Gaussian16. Sorted by multiplicity, then isomer.

Isomer	2s + 1	E (hartree)	Relative E (kcal/mol)
2a	1	-1537.266479	+16.3
2b	1	-1537.263705	+18.0
2c	1	-1537.261660	+19.3
2d	1	-1537.263316	+18.3
2a	3	-1537.292467	+0.0
2b	3	-1537.289272	+2.0
2c	3	-1537.268488	+15.0
2d	3	-1537.261270	+19.6
2a	5	-1537.200515	+57.7
2b	5	-1537.211968	+50.5
2c	5	-1537.250293	+26.5
2d	5	-1537.237274	+34.6

Figure S15. The optimized geometry of singlet-2a $Co^{+}(C_2H_2)_2$ followed by its predicted frequencies (cm^{-1}) and IR intensities (km/mol).

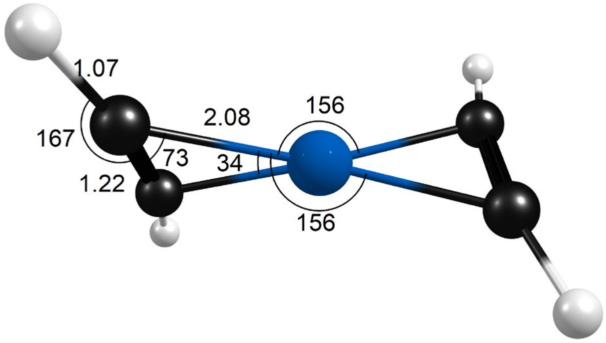

Frequency (cm^{-1})	Intensity (km/mol)
67.8911	0.0021
67.8937	0.0021
159.095	0
296.1812	0
358.7172	0.3625
358.721	0.3626
374.392	19.3706
654.6815	0
659.2677	0
725.5001	29.6714
725.5044	29.6726
752.4775	113.4503
752.4797	113.453
779.1148	2.6059
801.2676	0
1925.0102	0.0002
1926.6561	12.6964
3308.2259	192.2811
3308.2297	192.2873
3392.4897	122.6147
3395.5401	0.0001

Figure S16. The optimized geometry of singlet-2b $Co^+(C_2H_2)_2$ followed by its predicted frequencies (cm^{-1}) and IR intensities (km/mol).

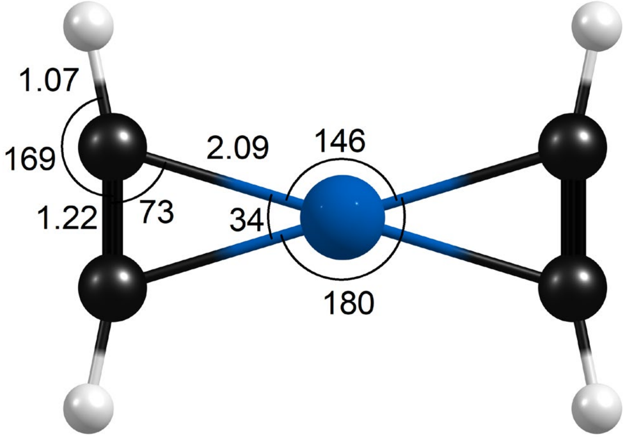

Frequency (cm^{-1})	Intensity (km/mol)
68.4354	1.2143
84.3324	0.3072
122.2365	0
251.8228	22.5231
294.053	0
319.0999	4.8384
331.8472	0
639.7051	0
640.6011	0
692.0413	0
715.8515	59.0913
757.9332	0
760.7581	13.0447
773.7046	180.8403
812.1274	0
1938.2414	3.4163
1946.6922	0
3313.3212	0
3314.148	414.1698
3400.0812	108.8418
3405.4555	0

Figure S17. The optimized geometry of singlet-2c $Co^{+}(C_2H_2)_2$ followed by its predicted frequencies (cm^{-1}) and IR intensities (km/mol).

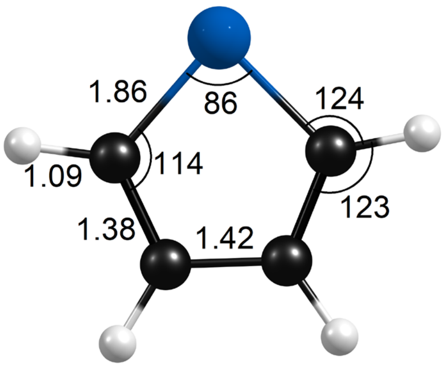

Frequency (cm^{-1})	Intensity (km/mol)
143.548	0.6984
318.3729	0
396.431	0.5219
493.8241	2.1846
591.2621	8.6929
640.2109	115.3482
759.599	11.7282
767.3784	0
944.0872	0.9515
1000.5523	0
1035.5801	0.4208
1089.0326	16.5678
1116.1937	2.0433
1248.1646	23.8009
1357.2861	5.8265
1473.3862	49.7116
1497.7967	3.7913
3120.1878	7.666
3124.2521	0.0612
3169.9072	2.5715
3181.6766	3.5109

Figure S18. The optimized geometry of singlet-2d $Co^+(C_2H_2)_2$ followed by its predicted frequencies (cm^{-1}) and IR intensities (km/mol).

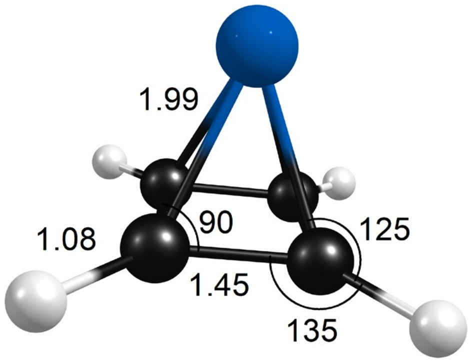

Frequency (cm^{-1})	Intensity (km/mol)
368.1117	3.4283
368.1123	3.4283
383.3739	18.411
404.7009	0
777.3912	28.2596
777.392	28.2594
811.2896	72.9316
870.2093	0
930.5773	8.0782
930.5779	8.0781
947.1243	0
952.9362	0
1208.0654	0
1212.7692	0
1233.718	0.3351
1331.2651	12.4063
1331.2652	12.4063
3221.5682	0
3233.4032	29.1532
3233.4039	29.1532
3249.8121	0.9702

Figure S19. The optimized geometry of triplet-2a $Co^+(C_2H_2)_2$ followed by its predicted frequencies (cm^{-1}) and IR intensities (km/mol).

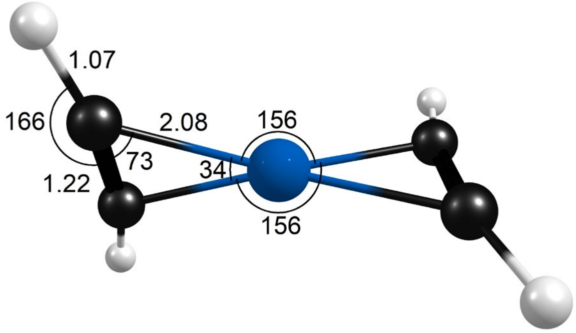

Frequency (cm^{-1})	Intensity (km/mol)
62.1401	0.0128
63.3009	0.0108
167.934	0
299.2142	0
358.6991	0.2712
359.3939	0.2931
380.9209	18.5543
655.9061	0
660.7331	0.0001
725.9771	27.0176
726.103	26.7715
751.9691	117.5456
751.9776	117.6458
775.2934	1.8321
798.6475	0.0002
1918.4585	0
1919.9899	14.5386
3306.0408	188.6065
3306.0723	188.4309
3389.3493	123.2122
3392.3179	0.0008

Figure S20. The optimized geometry of triplet-2b $Co^{+}(C_2H_2)_2$ followed by its predicted frequencies (cm^{-1}) and IR intensities (km/mol).

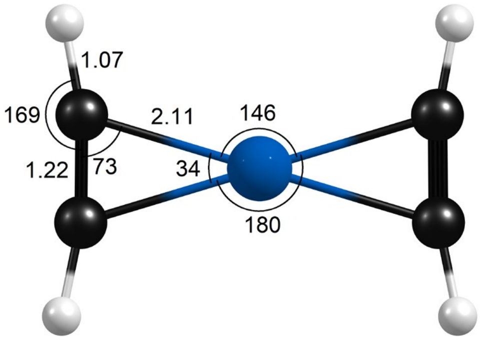

Frequency (cm^{-1})	Intensity (km/mol)
80.3877	1.1406
99.1873	0.218
114.5017	0
259.0418	24.1785
297.0564	0
314.753	5.6513
319.9969	0.0001
633.4539	0
634.1628	0
691.1492	0
715.8054	56.4862
759.3845	0
763.2666	12.457
769.9337	183.3908
815.7703	0
1945.0944	4.9833
1955.2123	0
3315.3041	0.0061
3316.2542	411.6199
3402.8147	109.7746
3408.3464	0.0001

Figure S21. The optimized geometry of triplet-2c $Co^+(C_2H_2)_2$ followed by its predicted frequencies (cm^{-1}) and IR intensities (km/mol).

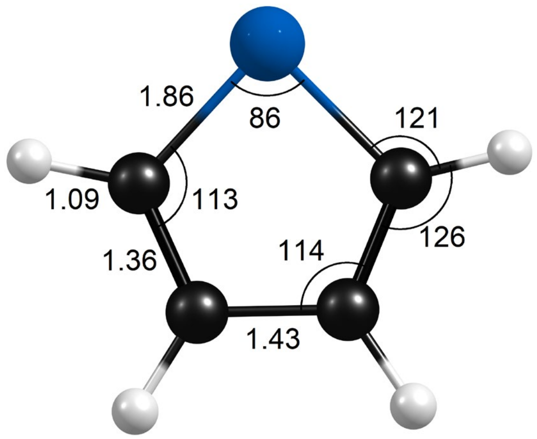

Frequency (cm^{-1})	Intensity (km/mol)
87.6835	0.0351
396.7272	7.8589
397.8443	0
480.6337	2.6275
582.7182	143.3527
584.3921	1.1068
720.234	0
768.0898	23.4976
868.6076	158.2729
903.999	6.0248
994.8421	0
1071.3073	24.8805
1089.9462	12.9293
1179.0043	91.5024
1233.8691	62.0904
1469.4606	5.082
1519.3374	12.4141
3128.7516	22.6359
3132.6585	2.213
3164.9115	7.6948
3175.3801	2.4303

Figure S22. The optimized geometry of triplet-2d $Co^+(C_2H_2)_2$ followed by its predicted frequencies (cm^{-1}) and IR intensities (km/mol).

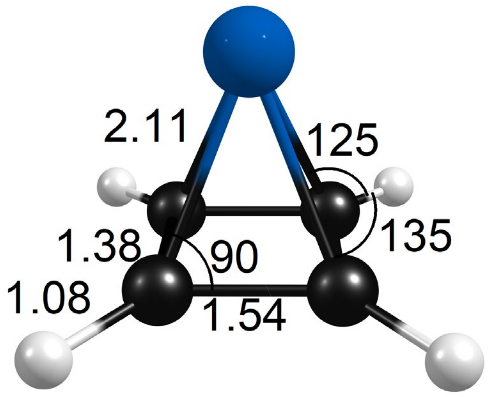

Frequency (cm^{-1})	Intensity (km/mol)
171.9297	7.9038
327.5036	1.9619
347.3491	1.2465
552.4685	0
723.8309	8.2428
761.2188	105.5879
766.2214	16.6085
831.9569	0
889.5923	6.6658
897.7341	7.3299
915.1927	0
1032.3466	10.9674
1113.2032	1.6773
1195.2123	0
1272.1086	41.1445
1406.0577	0.8183
1473.1483	0.4474
3216.7663	0
3232.2385	23.1845
3243.9388	21.9934
3252.2016	1.8726

Figure S23. The optimized geometry of quintet-2a $Co^+(C_2H_2)_2$ followed by its predicted frequencies (cm^{-1}) and IR intensities (km/mol).

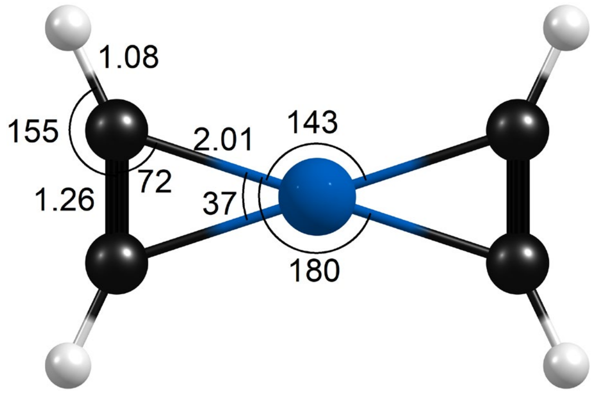

Frequency (cm^{-1})	Intensity (km/mol)
24.5538	0.0088
162.3328	5.5818
234.0888	0
248.1576	144.5548
391.0963	0
463.7737	0
482.1193	29.3625
575.3713	94.7054
589.1216	0
686.7264	0
687.6667	199.9265
715.7254	0
756.5202	0
774.2969	121.3657
831.9654	0
1650.4409	145.1215
1788.481	0
3224.5661	0
3226.2145	185.289
3281.7816	203.7931
3288.1536	0

Figure S24. The optimized geometry of quintet-2b $Co^+(C_2H_2)_2$ followed by its predicted frequencies (cm^{-1}) and IR intensities (km/mol).

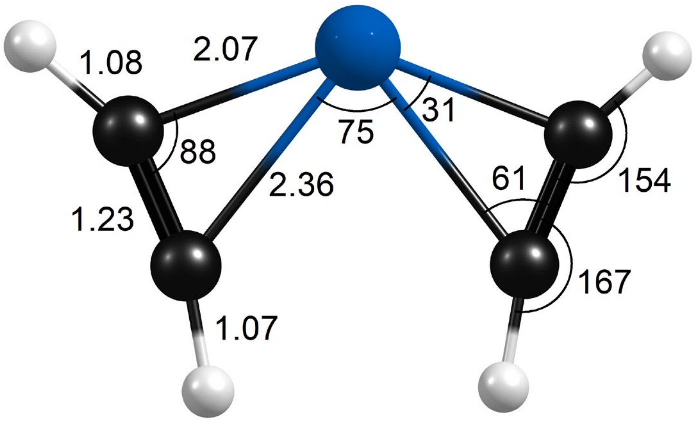

Frequency (cm^{-1})	Intensity (km/mol)
95.6246	0.5362
110.6214	0
126.8248	0.1178
146.3966	3.7112
270.713	1.4757
360.8539	65.0644
367.0566	0.29
605.7408	6.991
650.8395	0
660.4156	42.2426
696.8181	14.9464
713.3008	92.1024
721.8858	0
723.3741	14.7564
729.8075	158.1708
1820.4893	163.3248
1849.6221	31.0857
3270.4695	164.067
3272.3541	141.3871
3392.3308	117.4078
3403.9103	151.9561

Figure S25. The optimized geometry of quintet-2c $Co^+(C_2H_2)_2$ followed by its predicted frequencies (cm^{-1}) and IR intensities (km/mol).

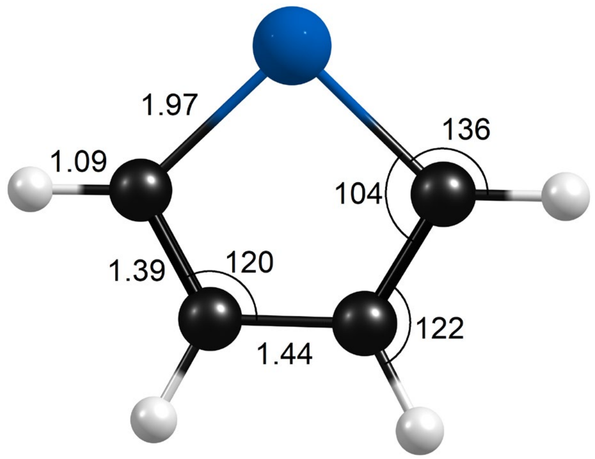

Frequency (cm^{-1})	Intensity (km/mol)
200.5324	6.8745
244.9318	0
276.6235	5.0918
461.8913	0.0238
614.8113	0.1511
653.4782	89.8439
739.2689	0
743.3223	10.7218
972.8607	0.2415
976.9189	0.1259
1019.4194	0
1104.8705	25.6871
1130.6278	7.4165
1260.1798	4.419
1335.1313	2.1072
1481.8352	73.3944
1490.6175	0.6409
3138.1012	0.1446
3150.4789	1.3417
3173.4183	8.747
3174.8597	0.0131

Figure S26. The optimized geometry of quintet-2d $Co^+(C_2H_2)_2$ followed by its predicted frequencies (cm^{-1}) and IR intensities (km/mol).

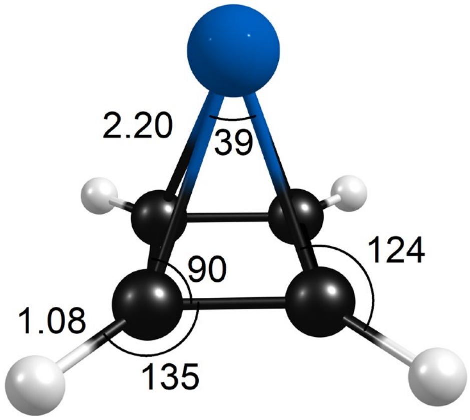

Frequency (cm^{-1})	Intensity (km/mol)
78.3598	14.4172
78.3604	14.4172
315.7031	0.0394
460.135	0
676.1071	26.2801
676.1071	26.2801
715.2471	115.2514
771.2631	0
904.0769	18.5927
904.0769	18.5927
962.6613	0
977.5299	0
1199.7741	0
1226.9798	0
1234.7868	4.9096
1311.3316	4.7524
1311.3316	4.7524
3230.572	0
3241.5921	27.0928
3241.5921	27.0927
3258.0754	1.474

Figure S27. The optimized geometry of TS1 triplet-$Co^+(C_2H_2)_2$ followed by its predicted frequencies (cm^{-1}) and IR intensities (km/mol).

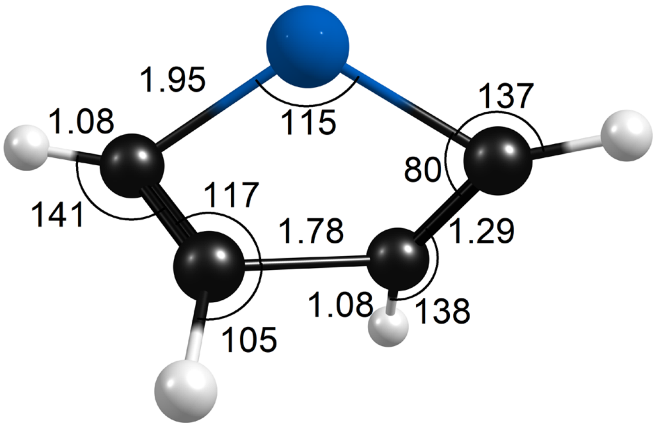

Frequency (cm^{-1})	Intensity (km/mol)
-647.978	13.4097
202.2352	24.5392
281.8379	0.0822
360.346	2.1554
381.6889	4.5361
523.2482	2.066
523.5083	19.0609
638.3077	384.5435
707.1853	51.1222
762.3378	63.7261
804.9053	94.5687
821.9799	2.1926
844.6424	19.4433
1012.7948	13.9231
1102.3755	33.7465
1516.1231	4.2249
1692.7398	38.8794
3193.3979	37.5727
3196.4014	39.5868
3255.1658	199.6959
3258.4439	15.7723

Figure S28. The optimized geometry of TS2 triplet-$Co^+(C_2H_2)_2$ followed by its predicted frequencies (cm^{-1}) and IR intensities (km/mol).

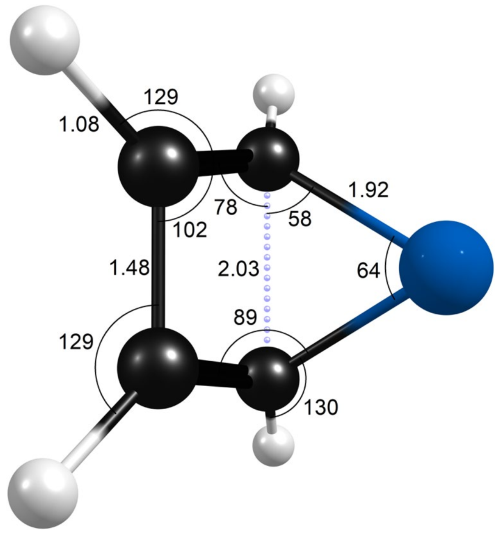

Frequency (cm^{-1})	Intensity (km/mol)
-394.5544	2.8479
189.0727	0.9025
257.1286	0.837
480.5427	16.1123
550.7033	5.8619
675.0499	105.113
734.1908	3.7389
801.922	16.4354
883.5323	26.0992
906.0189	11.6786
996.9414	9.7768
1014.305	2.7873
1015.8597	16.9852
1159.7629	47.6902
1288.719	36.5886
1456.6113	1.388
1509.1205	0.074
3182.1196	0.2901
3193.4215	20.4743
3212.4711	38.1866
3214.3701	7.7914

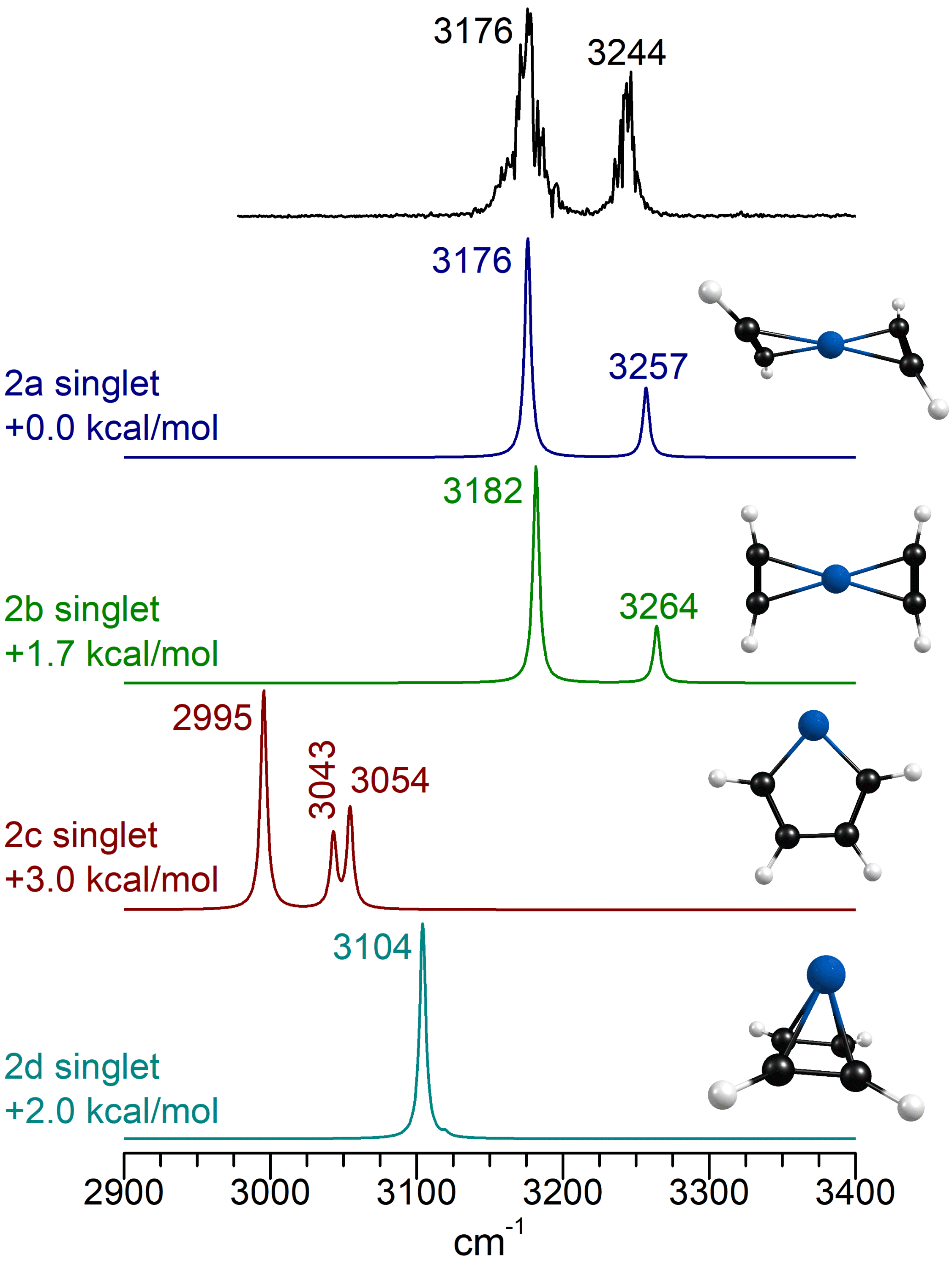

Figure S29. The experimental spectrum for $Co^+(C_2H_2)_2Ar$ with simulated spectra for $Co^+(C_2H_2)_2$ as a singlet and all predicted isomers of singlet-$Co^+(C_2H_2)_2$.

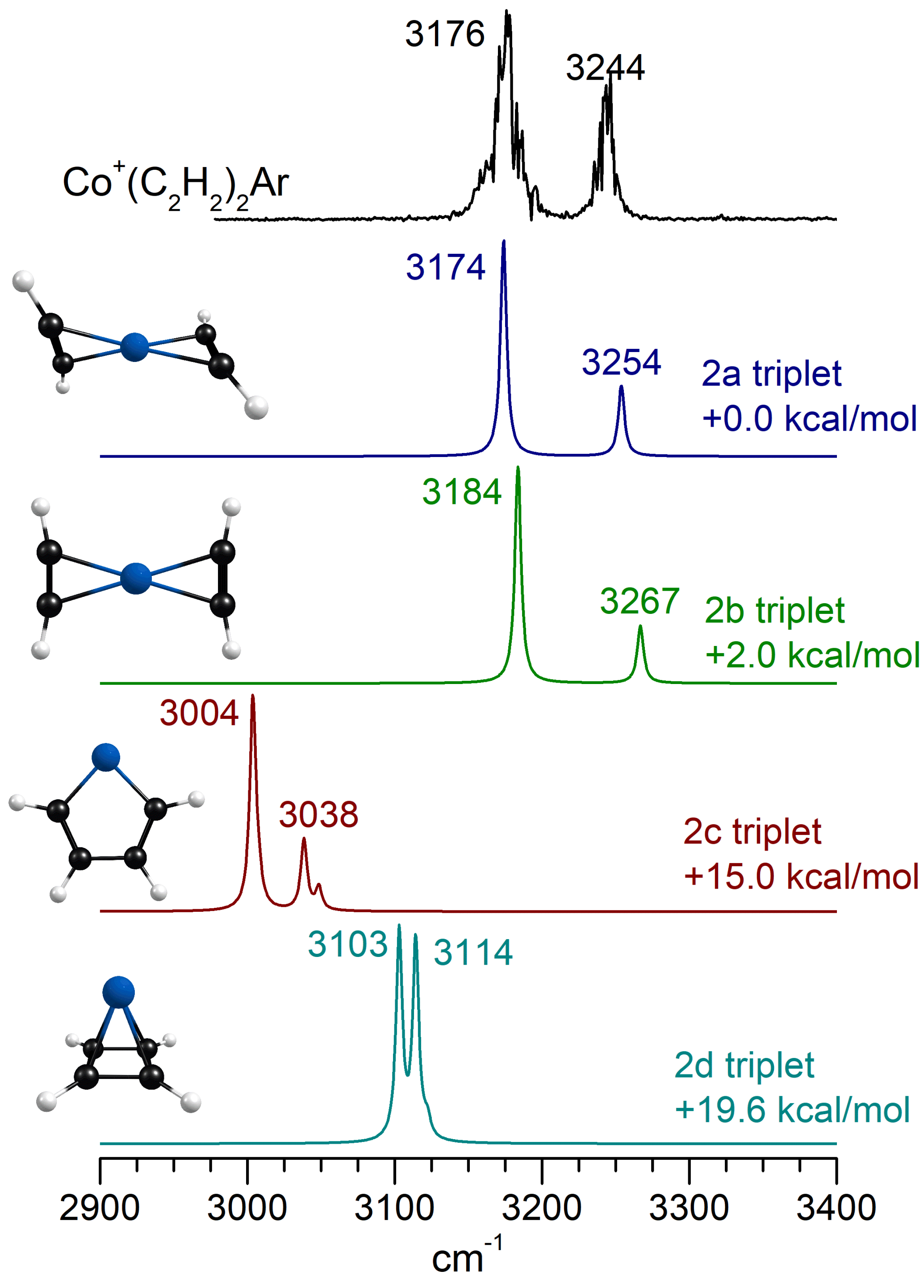

Figure S30. The experimental spectrum for $Co^{+}(C_2H_2)_2Ar$ with simulated spectra for $Co^{+}(C_2H_2)_2$ as a triplet and all predicted isomers of triplet-$Co^{+}(C_2H_2)_2$.

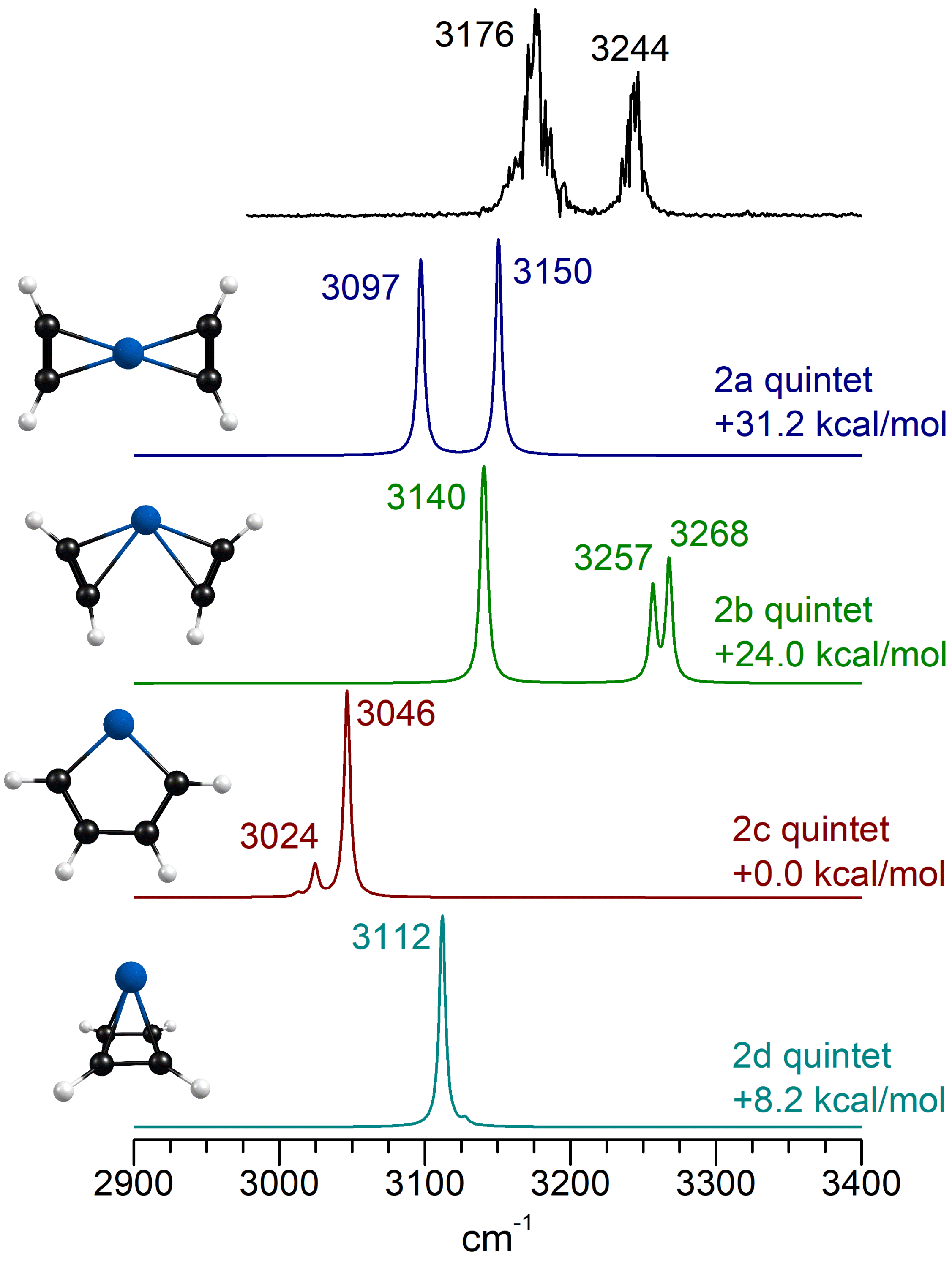

Figure S31. The experimental spectrum for $Co^+(C_2H_2)_2Ar$ with simulated spectra for $Co^+(C_2H_2)_2$ as a quintet and all predicted isomers of quintet-$Co^+(C_2H_2)_2$.

Table S6. $Co^{+}(C_2H_2)_3$ calculated at the B3LYP/Def2TZVP level of theory using Gaussian16. Sorted by isomer, then multiplicity.

Isomer	2s + 1	E (hartree)	Relative E (kcal/mol)
3a	1	-1614.792014	+107.6
3a	3	-1614.813851	+0.0
3a	5	-1614.760465	+114.4
3b	1	-1614.646145	+105.2
3b	3	-1614.667715	+91.7
3b	5	-1614.640577	+108.7
3c	1	-1614.659351	+97.0
3c	3	-1614.659935	+96.6
3c	5	-1614.634404	+112.6
3d	1	-1614.633122	+113.4
3d	3	-1614.659485	+96.9
3d	5	did not converge	
3e	1	-1614.627985	+116.6
3e	3	-1614.651585	+101.8
3e	5	-1614.592163	+139.1
3f	1	-1614.642332	+107.6
3f	3	-1614.647219	+104.6
3f	5	-1614.631628	+114.3
3g	1	-1614.686444	+79.9
3g	5	-1614.671401	+89.4

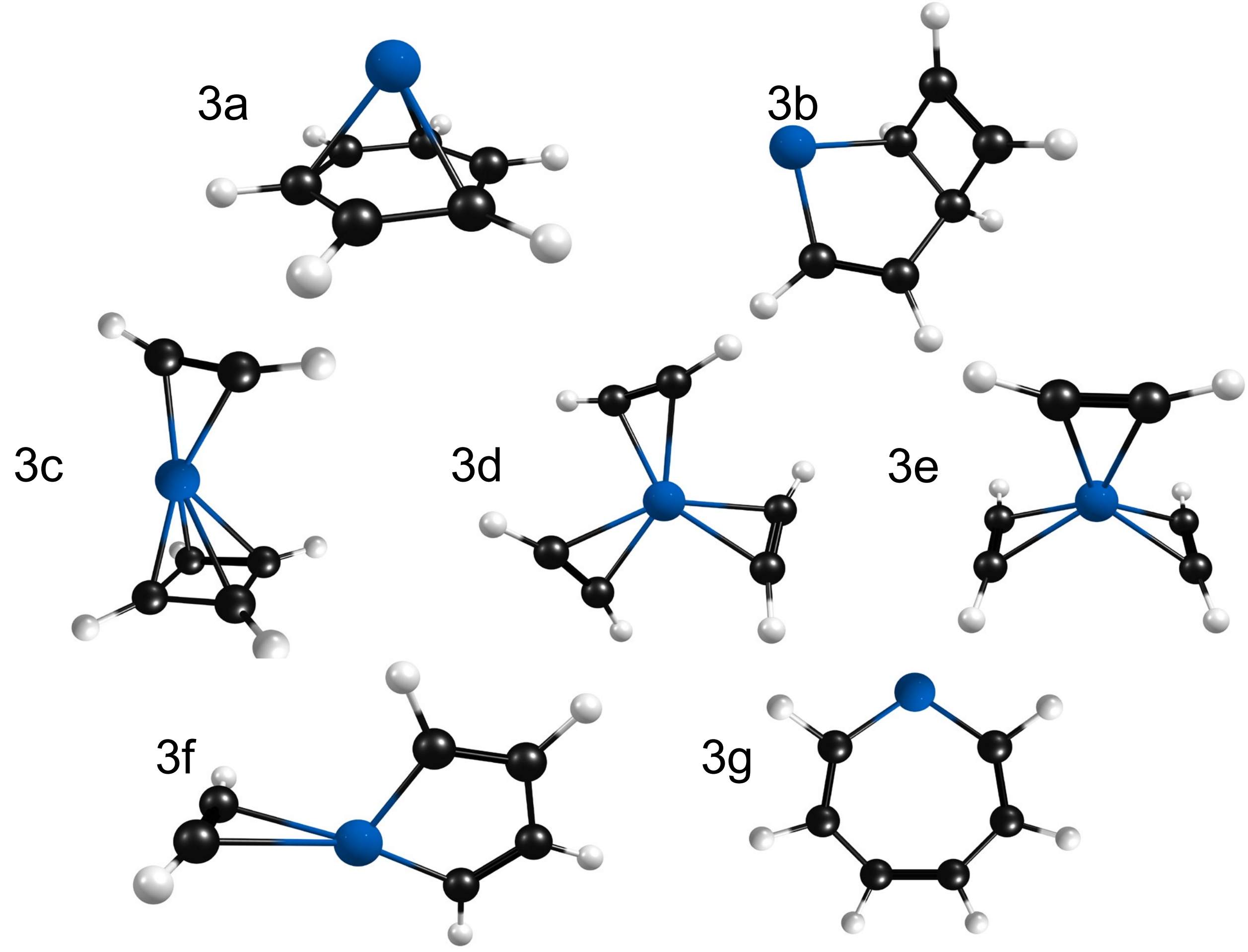

Figure S32. Predicted isomers for $Co^{+}(C_2H_2)_3$ complexes.

Table S7. Cartesian coordinates for the optimized geometry of isomer 3a-singlet of $Co^+(C_2H_2)_3$ followed by its predicted frequencies (cm^{-1}) and IR intensities (km/mol).

Z	x	y	z
6	1.221939000	0.705524000	-0.655125000
6	0.000000000	1.410811000	-0.655361000
6	1.221939000	-0.705524000	-0.655125000
1	0.000000000	2.492082000	-0.634279000
1	2.158277000	-1.246251000	-0.634178000
6	-1.221939000	0.705524000	-0.655125000
6	0.000000000	-1.410811000	-0.655361000
1	-2.158277000	1.246251000	-0.634178000
1	0.000000000	-2.492082000	-0.634279000
6	-1.221939000	-0.705524000	-0.655125000
1	-2.158277000	-1.246251000	-0.634178000
1	2.158277000	1.246251000	-0.634178000
27	0.000000000	0.000000000	1.014541000

Frequency (cm^{-1})	Intensity (km/mol)	Frequency (cm^{-1})	Intensity (km/mol)
239.1325	1.1878	1039.631	0
240.7873	1.216	1178.1086	0.0008
277.4594	0.0317	1180.725	0
408.4223	0	1183.2208	0.0001
412.2251	0	1316.4856	0.004
614.6321	0	1377.9384	0
620.9476	0	1487.115	15.1689
679.8073	0	1487.1388	15.2117
800.049	99.9527	1554.4433	0
918.8129	1.2944	1561.1303	0.0004
919.096	1.291	3197.3803	0.0009
989.4886	0.5419	3202.9924	0
994.1732	0	3203.0054	0
996.4824	0.0005	3212.1757	10.1264
1002.6806	0.0005	3212.3102	10.1979
1034.9805	4.0439	3218.1755	0.0232
1035.2041	3.9688		

Table S8. Cartesian coordinates for the optimized geometry of isomer 3b-singlet of $Co^{+}(C_2H_2)_3$ followed by its predicted frequencies (cm^{-1}) and IR intensities (km/mol).

Z	x	y	z
6	0.539733000	-0.494067000	-0.964364000
6	1.338376000	0.693012000	-0.347213000
6	1.718674000	-0.328774000	0.711100000
6	1.029316000	-1.357213000	0.104126000
1	0.359678000	-0.710262000	-2.013498000
1	2.183666000	1.049607000	-0.938312000
1	2.331555000	-0.266141000	1.601662000
1	0.974238000	-2.420188000	0.303772000
27	-1.091735000	-0.470106000	0.094672000
6	0.380592000	1.778361000	0.065640000
6	-0.897466000	1.409366000	0.094259000
1	-1.762668000	2.055640000	0.219273000
1	0.735021000	2.780099000	0.28966800

Frequency (cm^{-1})	Intensity (km/mol)	Frequency (cm^{-1})	Intensity (km/mol)
101.73	1.0039	1007.3229	4.3659
174.9982	3.1987	1061.0322	17.4622
283.7798	2.4881	1137.4749	6.4358
301.4988	7.9235	1171.5031	8.5035
398.8586	12.6535	1203.5797	2.5449
443.5603	7.601	1267.4063	56.2832
554.7238	30.2211	1292.5733	5.6939
690.9326	1.6597	1324.3016	27.0673
700.1923	60.806	1447.2856	17.9785
757.6148	28.2297	1588.7912	7.7197
869.8054	75.1267	3085.6811	0.6281
884.4428	3.1464	3138.6518	8.1441
896.6659	24.1035	3153.5084	5.8311
907.3052	10.3027	3162.5044	5.9691
943.5253	3.0186	3190.6958	4.0728
971.9108	17.7255	3214.3688	3.2326
997.7536	7.8747		

Table S9. Cartesian coordinates for the optimized geometry of isomer 3c-singlet of $Co^{+}(C_2H_2)_3$ followed by its predicted frequencies (cm^{-1}) and IR intensities (km/mol).

Z	x	y	z
6	1.596727000	-0.450724000	-0.723608000
6	1.095338000	0.901763000	-0.726622000
6	1.095339000	0.901762000	0.726624000
6	1.596728000	-0.450724000	0.723606000
1	1.911681000	-1.142283000	-1.489973000
1	0.898612000	1.637438000	-1.490576000
1	0.898612000	1.637435000	1.490579000
1	1.911680000	-1.142285000	1.489972000
27	-0.289569000	-0.365602000	0.000000000
6	-2.293435000	-0.352456000	0.000000000
6	-1.922634000	0.821530000	-0.000001000
1	-1.910384000	1.892581000	0.000000000
1	-2.900204000	-1.238552000	0.000001000

Frequency (cm^{-1})	Intensity (km/mol)	Frequency (cm^{-1})	Intensity (km/mol)
51.1565	0.0544	943.4276	0.4856
110.8991	0.6841	948.724	0.202
122.0367	0.7811	951.2883	8.2319
299.7858	1.9656	963.0566	9.7249
330.2003	57.0482	1196.8664	0.2631
378.4112	9.5333	1212.5832	0.0041
395.8269	22.6793	1258.3311	1.1704
462.2036	1.0674	1354.5341	13.7436
478.2993	23.866	1361.9643	10.8485
663.5353	17.5012	1879.2797	26.979
709.9031	30.3176	3227.621	1.6663
762.7988	68.1624	3243.1164	19.2033
781.37	6.2803	3245.4865	18.548
815.4467	50.2134	3259.7461	3.1995
827.2399	4.9355	3302.0725	150.8235
830.8088	7.55	3384.6307	69.9119
873.9183	0.7044		

Table S10. Cartesian coordinates for the optimized geometry of isomer 3d-singlet of $Co^{+}(C_2H_2)_3$ followed by its predicted frequencies (cm^{-1}) and IR intensities (km/mol).

Z	x	y	z
27	-0.000001000	-0.001520000	-0.000004000
6	2.021094000	0.530352000	-0.283252000
6	1.482069000	1.470962000	0.284582000
1	1.273809000	2.403954000	0.764860000
1	2.719612000	-0.121927000	-0.763968000
6	-0.553230000	-2.012517000	-0.282031000
6	0.533359000	-2.017876000	0.282040000
1	1.446145000	-2.308286000	0.758612000
1	-1.468855000	-2.293907000	-0.758569000
6	-1.467501000	1.485505000	-0.284555000
6	-2.015782000	0.550229000	0.283225000
1	-2.720707000	-0.095147000	0.763905000
1	-1.250019000	2.416409000	-0.764796000

Frequency (cm^{-1})	Intensity (km/mol)	Frequency (cm^{-1})	Intensity (km/mol)
82.263	1.031	718.9777	48.5015
98.3382	1.8313	766.9433	25.6605
103.9119	0.0755	767.5214	27.2802
110.7719	0.0001	776.4979	159.0438
142.2013	1.8762	791.5876	5.9473
145.2922	1.5432	792.7229	6.7527
248.5955	3.0807	799.6794	0.0004
263.2667	3.5736	1918.0707	4.3359
265.1832	0.0216	1921.1863	0.5797
390.1867	3.2843	1921.9011	5.8202
390.8198	5.3336	3334.9891	104.6807
393.5589	2.5262	3336.0802	142.4098
678.1489	0.6475	3336.4767	150.8133
681.4532	0.1703	3415.8454	69.2527
684.077	0.0051	3416.8739	75.8786
714.6968	4.5124	3418.5267	3.5152
716.6392	7.1466		

Table S11. Cartesian coordinates for the optimized geometry of isomer 3e-singlet of $Co^+(C_2H_2)_3$ followed by its predicted frequencies (cm^{-1}) and IR intensities (km/mol).

Z	x	y	z
27	0.000005000	0.000948000	-0.000002000
6	-0.613054000	1.975829000	0.000008000
6	0.613000000	1.975849000	0.000008000
1	1.643876000	2.263176000	0.000011000
1	-1.643939000	2.263127000	0.000012000
6	-1.847322000	-0.996822000	-0.607788000
6	-1.847314000	-0.996840000	0.607782000
1	-2.024595000	-1.084644000	1.659286000
1	-2.024613000	-1.084598000	-1.659293000
6	1.847339000	-0.996786000	-0.607788000
6	1.847329000	-0.996809000	0.607783000
1	2.024613000	-1.084618000	1.659287000
1	2.024643000	-1.084556000	-1.659291000

Frequency (cm^{-1})	Intensity (km/mol)	Frequency (cm^{-1})	Intensity (km/mol)
43.3783	0.0026	722.5031	31.5486
55.6329	0.1095	749.3813	55.905
56.4642	0	755.6949	86.8447
105.2583	1.0063	761.8402	101.1166
122.0684	0.3653	772.1746	5.178
138.9455	0	777.3899	19.9719
181.8965	0.3407	796.491	9.081
231.6196	0.4219	1912.0939	10.3121
283.1902	0	1958.0562	0.2309
309.4341	8.0219	1964.8176	2.2146
330.6627	9.3455	3330.0033	119.8467
405.0212	0.8197	3337.1759	0.0012
643.7495	0	3338.3244	339.2023
651.8779	0.822	3410.1505	48.9417
673.2551	0	3425.4475	59.9497
692.7477	0	3428.4078	18.6814
695.5446	17.1591		

Table S12. Cartesian coordinates for the optimized geometry of isomer 3f-singlet of $Co^+(C_2H_2)_3$ followed by its predicted frequencies (cm^{-1}) and IR intensities (km/mol).

Z	x	y	z
27	0.425779000	-0.259852000	0.000026000
6	-0.703349000	1.237817000	-0.000031000
6	-2.062400000	0.982638000	-0.000009000
1	-2.817757000	1.762277000	-0.000018000
1	-0.309225000	2.252751000	-0.000081000
6	-2.370055000	-0.399526000	0.000008000
6	-1.238229000	-1.198236000	0.000000000
1	-1.336185000	-2.284304000	-0.000027000
1	-3.387393000	-0.776909000	0.000016000
6	2.444558000	0.158623000	-0.608417000
6	2.444629000	0.158655000	0.608365000
1	2.631858000	0.211370000	1.661695000
1	2.631732000	0.211011000	-1.661773000

Frequency (cm^{-1})	Intensity (km/mol)	Frequency (cm^{-1})	Intensity (km/mol)
37.0034	0.7571	969.511	1.2188
64.0726	0.5843	1001.7498	0.0012
126.2662	0.1192	1046.8075	0.6486
197.1869	0.0009	1097.8504	10.331
239.002	9.3022	1117.3636	0.459
297.968	0.0237	1262.5109	13.348
347.6094	6.6573	1369.4926	0.2875
398.5355	9.9605	1472.4271	54.6456
487.1264	1.3092	1507.7768	6.03
579.8161	1.9996	1955.9334	5.9329
647.6036	25.2874	3110.46	0.1667
650.7653	27.497	3129.7333	0.6271
703.3217	45.2382	3166.6433	0.3079
764.1611	61.7086	3179.6131	0.3306
772.0966	15.9082	3325.8235	184.5107
778.7286	0.0175	3415.2415	66.9377
786.9908	15.2053		

Table S13. Cartesian coordinates for the optimized geometry of isomer 3g-singlet of $Co^{+}(C_2H_2)_3$ followed by its predicted frequencies (cm^{-1}) and IR intensities (km/mol).

Z	x	y	z
6	2.025795000	0.696921000	0.144683000
6	0.991256000	1.612006000	-0.119665000
6	-0.346213000	1.366211000	-0.275809000
6	2.025804000	-0.696908000	0.144661000
6	0.991271000	-1.612003000	-0.119683000
6	-0.346204000	-1.366219000	-0.275778000
1	1.306890000	-2.648645000	-0.215052000
1	2.996327000	1.147615000	0.318608000
1	1.306866000	2.648653000	-0.215017000
1	-0.978197000	2.194379000	-0.625565000
1	2.996345000	-1.147594000	0.318559000
1	-0.978200000	-2.194404000	-0.625475000
27	-1.433344000	-0.000002000	0.150129000

Frequency (cm^{-1})	Intensity (km/mol)	Frequency (cm^{-1})	Intensity (km/mol)
61.3169	0.7039	1044.5704	3.3565
207.2831	1.4619	1140.0171	16.5246
236.5681	5.3133	1211.4229	12.3602
323.1299	0.4678	1299.7836	0.3371
356.7182	1.5693	1313.2655	2.0433
510.3772	7.2474	1355.2026	18.7689
523.1014	4.0226	1486.0294	28.8407
562.3129	0.6032	1487.1262	6.3772
612.3996	54.028	1548.5785	8.686
627.8656	0.8105	1576.557	23.8292
673.4645	60.2083	3017.0198	3.2468
835.2368	1.4841	3017.5479	1.0202
845.9884	0.0003	3128.3536	2.6944
903.0195	1.4931	3128.5162	0.4201
941.2878	8.3025	3169.0728	0.6274
999.2633	0.0868	3183.3828	0.3509
1041.2505	0.0209		

Table S14. Cartesian coordinates for the optimized geometry of isomer 3a-triplet of $Co^{+}(C_2H_2)_3$ followed by its predicted frequencies (cm^{-1}) and IR intensities (km/mol).

Z	x	y	z
6	1.222002000	0.705515000	-0.659519000
6	0.000000000	1.411025000	-0.659471000
6	1.222002000	-0.705515000	-0.659519000
1	0.000000000	2.492306000	-0.643098000
1	2.158415000	-1.246163000	-0.643186000
6	-1.222002000	0.705515000	-0.659519000
6	0.000000000	-1.411025000	-0.659471000
1	-2.158415000	1.246163000	-0.643186000
1	0.000000000	-2.492306000	-0.643098000
6	-1.222002000	-0.705515000	-0.659519000
1	-2.158415000	-1.246163000	-0.643186000
1	2.158415000	1.246163000	-0.643186000
27	0.000000000	0.000000000	1.022261000

Frequency (cm^{-1})	Intensity (km/mol)	Frequency (cm^{-1})	Intensity (km/mol)
241.1457	0.8851	1038.3687	0.0003
241.2661	0.8845	1151.4945	0.0002
275.3133	0.0047	1183.2918	0
416.2409	0	1183.3133	0
416.2919	0	1270.6759	0.0002
623.8661	0	1377.6191	0
623.8828	0	1486.5603	14.5925
675.4097	0	1486.573	14.5956
797.6689	103.4971	1560.747	0
917.971	1.3822	1560.7981	0
918.0248	1.3827	3196.0735	0.0001
976.0151	0.0001	3203.7236	0
988.1178	0.5954	3203.7516	0
995.2359	0	3212.7815	9.7663
995.2939	0	3212.8104	9.7674
1034.6658	4.4223	3218.6651	0.0127
1034.6835	4.4206		

Table S15. Cartesian coordinates for the optimized geometry of isomer 3b-triplet of $Co^{+}(C_2H_2)_3$ followed by its predicted frequencies (cm^{-1}) and IR intensities (km/mol).

Z	x	y	z
6	1.889039000	-0.246582000	-0.633523000
6	1.318537000	0.713084000	0.392682000
6	0.536722000	-0.539751000	0.887561000
6	1.203890000	-1.326737000	-0.157150000
1	2.623535000	-0.120835000	-1.418644000
1	2.060117000	1.097385000	1.096473000
1	0.350655000	-0.825884000	1.920766000
1	1.230272000	-2.380670000	-0.400037000
27	-1.165344000	-0.493249000	-0.093908000
6	0.354611000	1.772787000	-0.066863000
6	-0.923370000	1.413640000	-0.106976000
1	-1.786432000	2.057857000	-0.249532000
1	0.709558000	2.771212000	-0.307905000

Frequency (cm^{-1})	Intensity (km/mol)	Frequency (cm^{-1})	Intensity (km/mol)
62.376	3.8887	1009.8795	12.9691
204.7217	2.1585	1060.4555	15.2568
260.8293	2.1307	1132.9861	5.2977
289.8555	10.3783	1178.4589	11.0297
398.7514	13.7238	1202.5146	4.931
464.5388	9.4154	1261.0228	57.1077
527.8953	45.7217	1296.5089	6.0752
694.9006	71.7758	1325.2474	29.3464
708.2299	1.8413	1498.6925	22.8927
754.4073	28.2817	1595.0156	14.5034
872.5755	96.6394	3075.1782	0.6834
878.1875	28.0014	3130.2271	5.684
909.2546	4.0129	3140.1489	7.4016
930.0122	48.8753	3161.0114	17.0738
972.1453	12.728	3198.276	2.5306
987.0695	10.3376	3220.9281	1.4493
1001.4455	9.6918		

Table S16. Cartesian coordinates for the optimized geometry of isomer 3c-triplet of $Co^{+}(C_2H_2)_3$ followed by its predicted frequencies (cm^{-1}) and IR intensities (km/mol).

Z	x	y	z
6	1.581472000	-0.595406000	-0.692457000
6	1.335795000	0.917363000	-0.686831000
6	1.334673000	0.918855000	0.685747000
6	1.580379000	-0.593945000	0.694968000
1	1.845138000	-1.320769000	-1.447312000
1	1.262822000	1.677930000	-1.448981000
1	1.260588000	1.681046000	1.446159000
1	1.842868000	-1.317689000	1.451784000
27	-0.353776000	-0.301924000	-0.000523000
6	-2.366442000	-0.355298000	0.000339000
6	-2.068970000	0.839714000	0.000202000
1	-2.106095000	1.910003000	0.000209000
1	-2.934799000	-1.266278000	0.000466000

Frequency (cm^{-1})	Intensity (km/mol)	Frequency (cm^{-1})	Intensity (km/mol)
35.1924	0.0285	898.5688	1.4229
137.8848	1.294	925.3019	4.1896
157.0925	0.1347	928.3567	0.5034
225.9441	2.3746	1034.4859	6.9833
297.5613	4.8926	1122.4552	0.5621
332.9035	3.5254	1200.0559	0.1687
390.0316	36.1344	1281.7484	34.2808
455.9713	7.6286	1407.7264	1.5858
560.0596	0.9679	1490.5937	2.0487
678.1472	7.0248	1877.6423	33.9582
718.0735	30.3761	3220.5206	1.6157
747.0635	20.025	3236.2918	16.8042
754.1718	80.7787	3245.774	15.8225
764.8783	28.4016	3256.8976	5.2951
771.0437	50.8499	3305.0277	146.9455
785.8493	17.048	3387.9549	86.5285
855.6518	1.4768		

Table S17. Cartesian coordinates for the optimized geometry of isomer 3d-triplet of $Co^+(C_2H_2)_3$ followed by its predicted frequencies (cm^{-1}) and IR intensities (km/mol).

Z	x	y	z
27	-0.000003000	-0.000024000	-0.000037000
6	-1.747036000	-1.167494000	-0.291129000
6	-2.095843000	-0.150272000	0.291170000
1	-2.651567000	0.620800000	0.782160000
1	-1.712691000	-2.117344000	-0.782100000
6	1.178106000	-1.739859000	0.291195000
6	1.884611000	-0.929177000	-0.291155000
1	2.689979000	-0.424476000	-0.782184000
1	0.788231000	-2.606663000	0.782218000
6	-0.137579000	2.096710000	-0.291127000
6	0.917757000	1.890181000	0.291187000
1	1.863382000	1.985932000	0.782190000
1	-0.977344000	2.541856000	-0.782135000

Frequency (cm^{-1})	Intensity (km/mol)	Frequency (cm^{-1})	Intensity (km/mol)
103.2792	1.5646	714.4947	44.1744
116.1442	0.1494	764.6045	24.8475
116.2396	0.1488	764.646	24.9025
131.9765	0	775.4715	165.0559
142.9735	1.5313	789.6866	7.9184
143.0457	1.5307	789.7012	7.8948
259.3923	4.4964	799.2222	0
259.4321	4.4964	1926.0647	8.3701
268.689	0	1926.0842	8.3574
385.4064	1.7624	1927.6843	0.0005
388.7659	6.2145	3335.9267	102.0761
388.8368	6.2283	3336.8836	151.3321
678.9691	0.0884	3336.894	151.2921
678.9964	0.0886	3417.8931	75.3197
683.1151	0	3417.9017	75.2527
713.8682	7.8301	3420.0283	0.0008
713.8977	7.9337		

Table S18. Cartesian coordinates for the optimized geometry of isomer 3e-triplet of $Co^{+}(C_2H_2)_3$ followed by its predicted frequencies (cm^{-1}) and IR intensities (km/mol).

Z	x	y	z
27	0.000011000	0.057094000	0.000011000
6	-1.836691000	-1.078803000	0.606151000
6	-1.836745000	-1.078699000	-0.606201000
1	-1.992157000	-1.165046000	-1.660786000
1	-1.992024000	-1.165337000	1.660733000
6	1.836724000	-1.078737000	0.606150000
6	1.836777000	-1.078632000	-0.606203000
1	1.992200000	-1.164977000	-1.660787000
1	1.992066000	-1.165271000	1.660731000
6	-0.614668000	2.027120000	0.000028000
6	0.614564000	2.027159000	0.000032000
1	1.638288000	2.341372000	0.000045000
1	-1.638413000	2.341265000	0.000033000

Frequency (cm^{-1})	Intensity (km/mol)	Frequency (cm^{-1})	Intensity (km/mol)
34.7242	0.0355	725.77	34.0795
41.0151	0.098	750.6555	90.9734
45.6727	0	755.3853	87.703
104.249	0.4291	758.801	75.4036
117.1347	0.0902	776.6907	46.3252
151.1417	0	776.8222	0.6823
179.6324	0.0029	801.3768	10.2952
241.4082	0.1089	1896.0876	16.6081
259.7976	0	1977.6809	0.0045
282.3366	6.9097	1984.6694	0.998
343.8073	7.6217	3321.408	115.6387
407.9621	0.0444	3343.4515	0.0004
632.8148	0	3344.5992	346.0373
640.412	0.3417	3398.9781	50.5062
671.0536	0	3434.3175	49.3346
692.5064	14.2908	3437.0668	21.1948
694.5546	0		

Table S19. Cartesian coordinates for the optimized geometry of isomer 3f-triplet of $Co^{+}(C_2H_2)_3$ followed by its predicted frequencies (cm^{-1}) and IR intensities (km/mol).

Z	x	y	z
27	0.397015000	-0.282754000	-0.000062000
6	-0.678478000	1.235592000	-0.000176000
6	-2.029867000	1.014771000	0.000033000
1	-2.765237000	1.813766000	0.000094000
1	-0.215580000	2.220053000	-0.000351000
6	-2.372351000	-0.372469000	0.000136000
6	-1.267475000	-1.182697000	-0.000014000
1	-1.342910000	-2.270167000	-0.000042000
1	-3.397330000	-0.728233000	0.000290000
6	2.484691000	0.167714000	-0.606064000
6	2.484524000	0.167907000	0.606318000
1	2.637516000	0.207176000	1.665394000
1	2.637886000	0.206853000	-1.665115000

Frequency (cm^{-1})	Intensity (km/mol)	Frequency (cm^{-1})	Intensity (km/mol)
50.5867	0.4669	850.3109	544.4231
65.4468	2.4121	931.6175	3.0161
106.7744	0.1106	1001.6502	0.0321
171.8681	0.0287	1087.5773	15.9999
176.3889	92.0288	1098.6119	15.75
298.7386	8.4645	1165.7177	308.2761
370.3177	0.2766	1255.1913	37.5219
407.8819	15.8933	1463.8521	36.6366
479.6217	5.6185	1504.7615	21.7762
572.0002	104.9757	1982.1604	2.2844
625.0048	72.8058	3112.2521	1.0708
642.409	0.3434	3137.1647	1.4744
702.2264	23.1198	3164.8046	0.6459
715.3684	13.2399	3178.3404	0.5143
757.7454	288.8374	3331.6794	199.9788
773.7308	23.5905	3424.6643	88.5327
789.3035	37.2697		

Table S20. Cartesian coordinates for the optimized geometry of isomer 3a-quintet of $Co^+(C_2H_2)_3$ followed by its predicted frequencies (cm^{-1}) and IR intensities (km/mol).

Z	x	y	z
6	1.248624000	0.697067000	-0.666293000
6	0.000000000	1.373352000	-0.567439000
6	1.248624000	-0.697067000	-0.666293000
1	0.000000000	2.448736000	-0.439242000
1	2.171583000	-1.260271000	-0.672891000
6	-1.248624000	0.697067000	-0.666293000
6	0.000000000	-1.373352000	-0.567439000
1	-2.171583000	1.260271000	-0.672891000
1	0.000000000	-2.448736000	-0.439242000
6	-1.248624000	-0.697067000	-0.666293000
1	-2.171583000	-1.260271000	-0.672891000
1	2.171583000	1.260271000	-0.672891000
27	0.000000000	0.000000000	0.976680000

Frequency (cm^{-1})	Intensity (km/mol)	Frequency (cm^{-1})	Intensity (km/mol)
118.4706	0.0454	1052.8243	0.3754
132.7477	0.0539	1193.3261	0.0007
179.3685	24.0514	1203.2866	0.0007
359.8704	0.0432	1204.1412	0
379.9782	0	1338.0313	0.0102
610.4537	0	1392.2112	0
610.7971	0.0027	1507.3326	34.4276
672.5712	0.015	1508.1839	34.5467
766.9393	116.9437	1601.5326	0.029
923.8077	0.6478	1601.9758	0
924.1441	0.6804	3193.521	0.0007
999.2558	3.1615	3199.6711	0
1021.8559	0.0036	3199.9054	0.0003
1022.6172	0.0026	3209.1153	11.6708
1024.9092	0	3209.2923	11.6976
1037.7443	0.0014	3214.9454	0.0053
1051.0826	0.3821		

Table S21. Cartesian coordinates for the optimized geometry of isomer 3b-quintet of $Co^+(C_2H_2)_3$ followed by its predicted frequencies (cm^{-1}) and IR intensities (km/mol).

Z	x	y	z
6	1.527302000	0.090068000	-0.935188000
6	1.164928000	0.964799000	0.274337000
6	1.037284000	-0.392407000	1.003471000
6	1.394084000	-1.087803000	-0.172826000
1	1.793647000	0.313109000	-1.957649000
1	1.991130000	1.573952000	0.641700000
1	1.006323000	-0.657879000	2.051573000
1	1.610217000	-2.129559000	-0.381176000
27	-0.982600000	-0.699938000	-0.030446000
6	-0.109840000	1.774620000	0.091885000
6	-1.287210000	1.186747000	-0.151699000
1	-2.211757000	1.724100000	-0.325178000
1	-0.018645000	2.858451000	0.132898000

Frequency (cm^{-1})	Intensity (km/mol)	Frequency (cm^{-1})	Intensity (km/mol)
80.473	0.3839	991.083	6.4087
175.6457	5.8646	1082.3131	7.149
204.525	5.4828	1138.4457	11.103
249.2487	2.9779	1167.0142	10.1984
352.0347	10.1071	1207.722	0.9773
381.7502	3.3244	1269.5828	12.9023
552.7793	16.0195	1295.4004	2.7412
636.8908	23.9619	1336.4785	5.3778
674.0683	5.4111	1393.4211	7.625
715.7714	39.6606	1554.1654	3.0924
722.5528	40.0142	3095.4419	0.2933
856.5587	18.3548	3123.3515	1.314
900.9127	11.6278	3181.9839	0.9962
907.7563	4.4063	3184.338	8.632
936.6832	20.4769	3212.3831	11.6152
953.5875	4.5206	3237.3836	5.6195
980.0231	13.8372		

Table S22. Cartesian coordinates for the optimized geometry of isomer 3c-quintet of $Co^{+}(C_2H_2)_3$ followed by its predicted frequencies (cm^{-1}) and IR intensities (km/mol).

Z	x	y	z
6	1.577421000	-0.719268000	-0.733973000
6	1.577414000	0.719369000	-0.733881000
6	1.577214000	0.719267000	0.734344000
6	1.577226000	-0.719353000	0.734251000
1	1.691653000	-1.477836000	-1.492496000
1	1.691639000	1.478033000	-1.492308000
1	1.691121000	1.477841000	1.492908000
1	1.691145000	-1.478022000	1.492718000
27	-0.385552000	-0.000006000	-0.000324000
6	-2.408442000	-0.611106000	0.000239000
6	-2.408435000	0.611114000	0.000243000
1	-2.655015000	1.654347000	0.000293000
1	-2.655036000	-1.654336000	0.000287000

Frequency (cm^{-1})	Intensity (km/mol)	Frequency (cm^{-1})	Intensity (km/mol)
41.1927	0.2048	885.9045	13.5554
79.459	0	939.7716	18.5954
114.144	0.447	959.1829	0
209.9013	1.3149	978.7518	0.0344
215.4095	3.2007	1202.6376	0
260.422	2.2586	1206.5117	0.6304
341.3753	24.6958	1271.7615	1.0724
361.5495	8.1168	1307.628	12.7157
464	0	1342.4814	4.4412
646.4485	0	1926.466	25.6957
680.9846	7.7897	3232.3121	0
696.1866	5.9415	3243.575	17.2579
708.2845	86.679	3245.3053	20.7389
722.529	25.4687	3261.1485	4.4679
745.5576	99.0635	3314.9191	170.8006
764.8469	12.9983	3399.1048	104.6291
781.8157	0		

Note: the 3d quintet isomer did not converge.

Table S23. Cartesian coordinates for the optimized geometry of isomer 3e-quintet of $Co^{+}(C_2H_2)_3$ followed by its predicted frequencies (cm^{-1}) and IR intensities (km/mol).

Z	x	y	z
6	1.999195000	0.128077000	-0.618034000
6	1.999175000	0.128109000	0.618149000
1	2.352490000	0.150750000	1.631261000
1	2.352562000	0.150660000	-1.631130000
27	0.000147000	0.000073000	-0.000013000
6	-1.110729000	1.666521000	0.618111000
6	-1.110606000	1.666602000	-0.618158000
1	-1.307315000	1.961941000	-1.631011000
1	-1.307618000	1.961699000	1.630976000
6	-0.888751000	-1.794762000	0.618112000
6	-0.888615000	-1.794829000	-0.618135000
1	-1.045882000	-2.112759000	-1.631033000
1	-1.046209000	-2.112558000	1.631021000

Frequency (cm^{-1})	Intensity (km/mol)	Frequency (cm^{-1})	Intensity (km/mol)
120.0101	1.5728	702.3158	0.0018
120.0867	1.5737	702.4299	0.0024
148.6461	3.7018	706.2564	109.9217
163.2378	0	716.3659	0.0486
170.1797	0	720.7051	137.5744
170.3399	0	720.8678	137.5431
237.7157	37.5353	816.8622	0
237.7839	37.3338	1801.4559	113.859
329.7322	0.0001	1801.8314	113.5357
397.7303	0.0087	1886.2867	0.0005
397.9304	0.0024	3291.829	3.1687
402.7976	28.331	3291.9951	9.0015
602.0571	44.8907	3292.6227	347.5846
602.2284	44.5971	3361.0118	139.8427
674.9605	0.0007	3361.2215	139.8326
675.2104	0.0002	3368.022	0.0671
677.0483	0		

Table S24. Cartesian coordinates for the optimized geometry of isomer 3f-quintet of $Co^+(C_2H_2)_3$ followed by its predicted frequencies (cm^{-1}) and IR intensities (km/mol).

Z	x	y	z
27	0.422315000	-0.000071000	0.000016000
6	-1.027541000	1.376624000	0.000075000
6	-2.246781000	0.712519000	0.000035000
1	-3.194668000	1.244604000	0.000070000
1	-1.031042000	2.463478000	0.000131000
6	-2.246856000	-0.712367000	-0.000059000
6	-1.027685000	-1.376601000	-0.000063000
1	-1.031305000	-2.463454000	-0.000123000
1	-3.194798000	-1.244353000	-0.000125000
6	2.575033000	0.000180000	-0.605618000
6	2.575058000	-0.000104000	0.605574000
1	2.721010000	-0.000352000	1.666338000
1	2.720934000	0.000475000	-1.666389000

Frequency (cm^{-1})	Intensity (km/mol)	Frequency (cm^{-1})	Intensity (km/mol)
54.6735	0	984.0931	0.0255
55.7024	0.7793	997.4145	0.0814
76.1044	1.2228	1013.9215	0
221.0189	0.6198	1105.1431	19.0534
222.1893	5.0437	1131.5193	2.3853
242.5971	0	1267.3706	3.73
270.2545	2.0835	1334.8499	0.5114
291.1434	0.3215	1475.1612	67.0701
430.6059	0.7174	1501.6391	0.041
576.0795	0.0045	1991.6573	2.2567
638.3749	0	3133.3664	0.2487
655.8017	66.7816	3142.5114	0.1351
703.4159	28.0227	3153.5468	0.0504
737.8235	3.4361	3159.056	0.2064
745.3355	0	3332.573	210.7193
768.4187	86.9371	3428.2395	42.353
814.9879	28.6789		

Table S25. Cartesian coordinates for the optimized geometry of isomer 3g-quintet of $Co^+(C_2H_2)_3$ followed by its predicted frequencies (cm^{-1}) and IR intensities (km/mol).

Z	x	y	z
6	0.025199000	-1.937089000	0.707119000
6	0.053972000	-0.975145000	1.737662000
6	0.053972000	0.401688000	1.672889000
6	0.025199000	-1.937089000	-0.707119000
6	0.053972000	-0.975145000	-1.737662000
6	0.053972000	0.401688000	-1.672889000
1	0.081638000	-1.421224000	-2.729382000
1	0.012497000	-2.950101000	1.096515000
1	0.081638000	-1.421224000	2.729382000
1	0.113142000	0.909224000	2.635389000
1	0.012497000	-2.950101000	-1.096515000
1	0.113142000	0.909224000	-2.635389000
27	-0.074528000	1.372250000	0.000000000

Frequency (cm^{-1})	Intensity (km/mol)	Frequency (cm^{-1})	Intensity (km/mol)
107.7102	0.6572	1050.6853	1.188
150.6037	0.0553	1162.7727	24.1836
185.7159	4.9472	1234.5605	11.1137
289.9303	3.24	1319.1726	29.4468
331.8692	1.3811	1328.3182	0.0412
453.5932	0.2925	1396.3186	0.0315
499.0635	0.2582	1482.9451	100.6891
540.971	4.4064	1504.9133	23.5164
610.9932	106.8969	1542.1741	16.0272
645.4886	17.3975	1549.8476	10.7862
706.0877	0.0375	3104.1721	2.4324
832.2576	3.6633	3105.0257	0.1819
835.5712	0.217	3126.2857	0.2075
853.7442	0.1086	3127.3951	0.1491
990.0583	0.3053	3151.8861	0.281
1030.5271	2.566	3168.8433	0.0367
1049.2647	1.1194		

Figure S33. The optimized geometry of a second-order saddle point for $Co^{+}(C_2H_2)_3$ followed by its predicted frequencies (cm^{-1}) and intensities (km/mol).

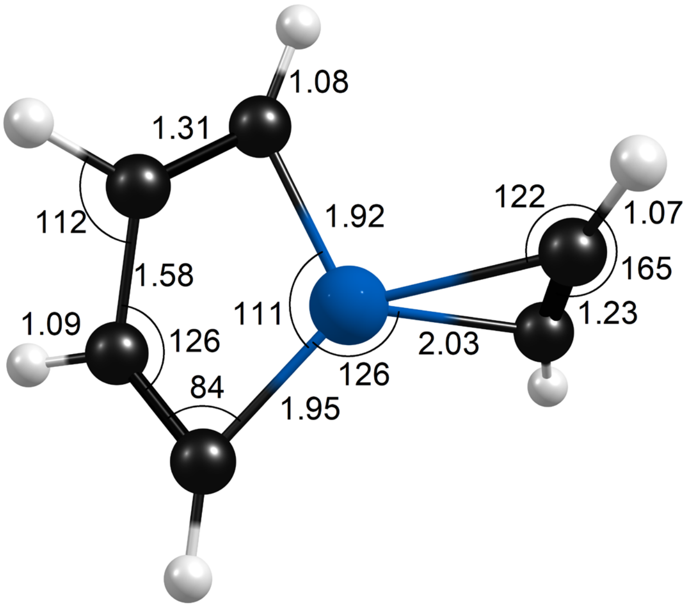

Frequency (cm^{-1})	Intensity (km/mol)	Frequency (cm^{-1})	Intensity (km/mol)
-241.2378	46.6732	780.4389	2.1714
-157.3982	9.7225	828.9639	158.7109
73.048	2.8096	865.134	49.9392
114.1409	1.3345	930.5216	51.5446
116.1314	2.9652	974.4325	27.7869
202.131	3.4434	1153.7252	14.4078
337.92	5.8462	1204.4813	66.2553
397.3042	3.5336	1488.3746	3.0575
411.4515	9.3652	1651.5136	1.9333
503.9831	23.1898	1896.6064	2.7178
563.2291	20.2758	3092.3868	11.1107
668.6605	2.5982	3107.8718	43.1278
680.5583	7.2045	3232.6811	21.5257
711.1681	45.0589	3262.4961	53.2525
726.4349	38.4063	3309.3559	179.4421
737.6401	47.9472	3391.5276	79.6062
757.7422	25.2825		

Figure S34. The optimized geometry of a transition state for $Co^+(C_2H_2)_3$ followed by its predicted frequencies (cm^{-1}) and intensities (km/mol).

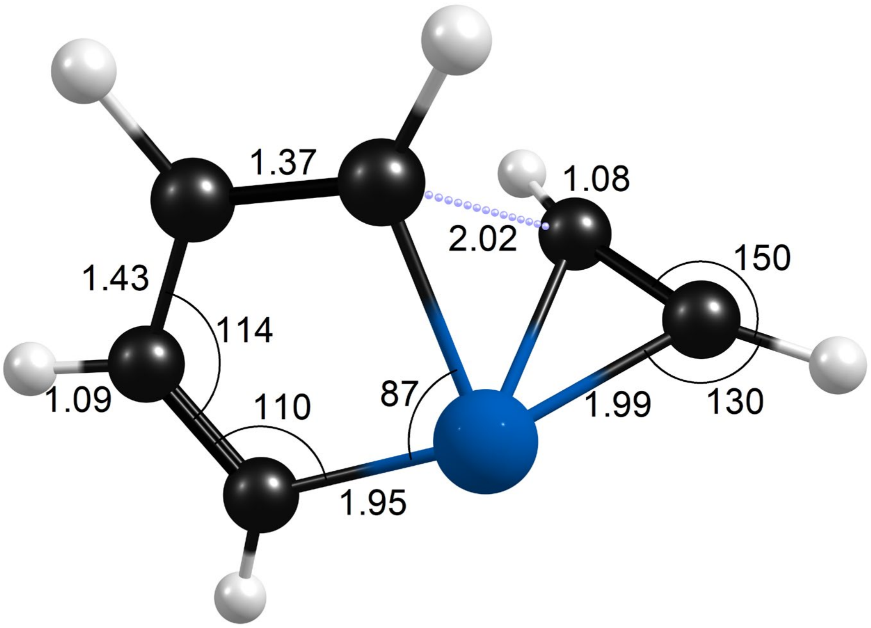

Frequency (cm^{-1})	Intensity (km/mol)	Frequency (cm^{-1})	Intensity (km/mol)
-445.8719	33.4456	954.6343	0.9265
127.5027	0.4682	991.5594	2.9079
139.1637	2.4386	1013.3315	2.3953
216.9759	2.0361	1098.8816	23.9304
309.1001	9.0033	1132.1355	13.4576
345.6019	7.4122	1235.5307	50.3953
414.1461	12.9858	1291.538	51.4857
444.7306	25.6322	1466.1829	61.9185
471.7245	4.6223	1532.977	7.5483
517.3506	70.393	1738.1774	23.4831
634.3953	74.2453	3129.3269	4.169
685.9465	116.9028	3133.5729	2.3135
708.2897	53.9575	3167.1694	1.1083
741.0354	34.8434	3220.4492	11.6875
777.6195	30.0679	3257.8857	109.407
815.7938	7.3887	3313.5798	99.3893
918.1377	6.8333		

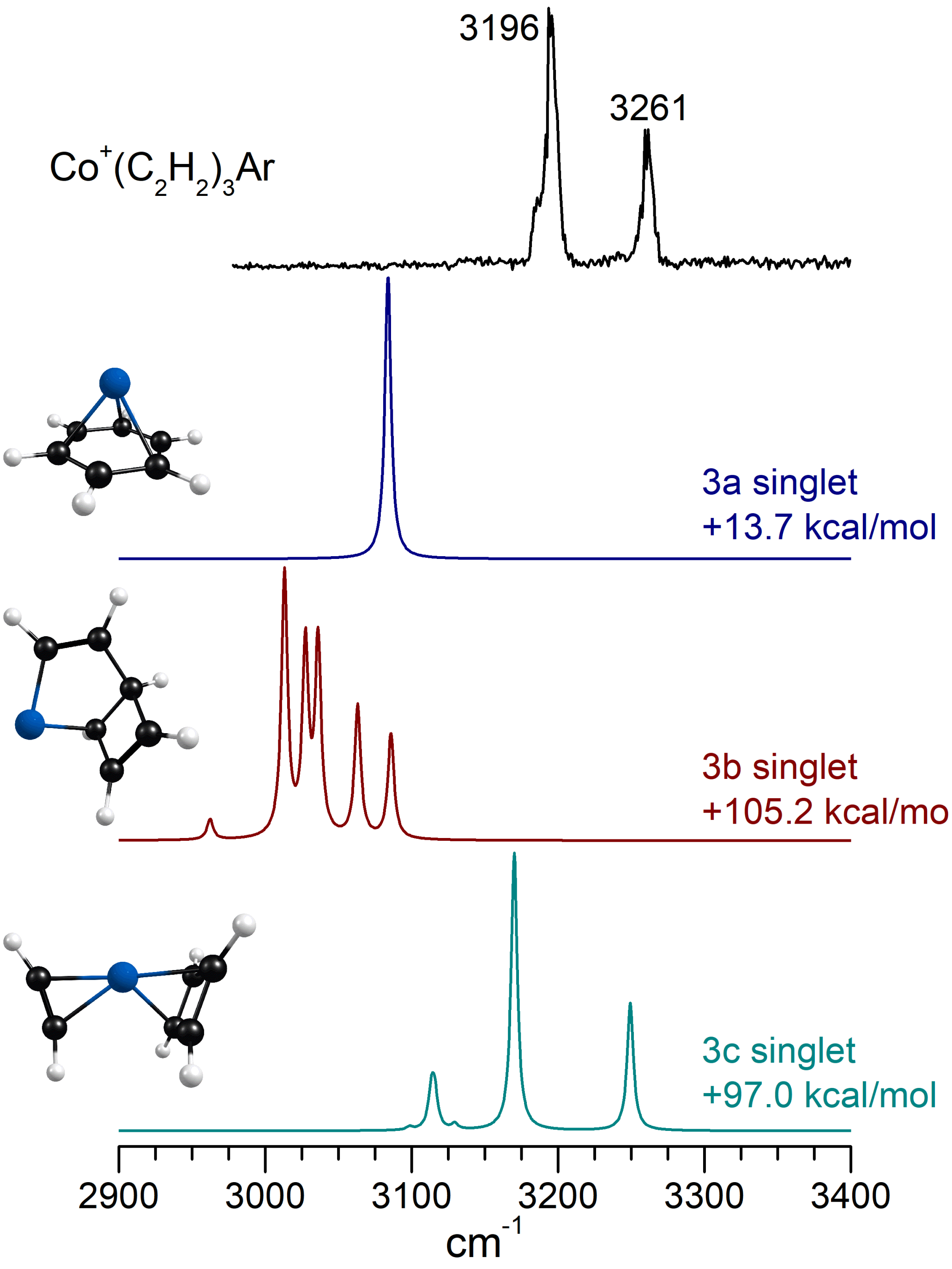

Figure S35. The experimental spectrum for $Co^{+}(C_2H_2)_3Ar$ with simulated spectra for $Co^{+}(C_2H_2)_3$ as a singlet and predicted isomers 3a-3c of singlet-$Co^{+}(C_2H_2)_3$.

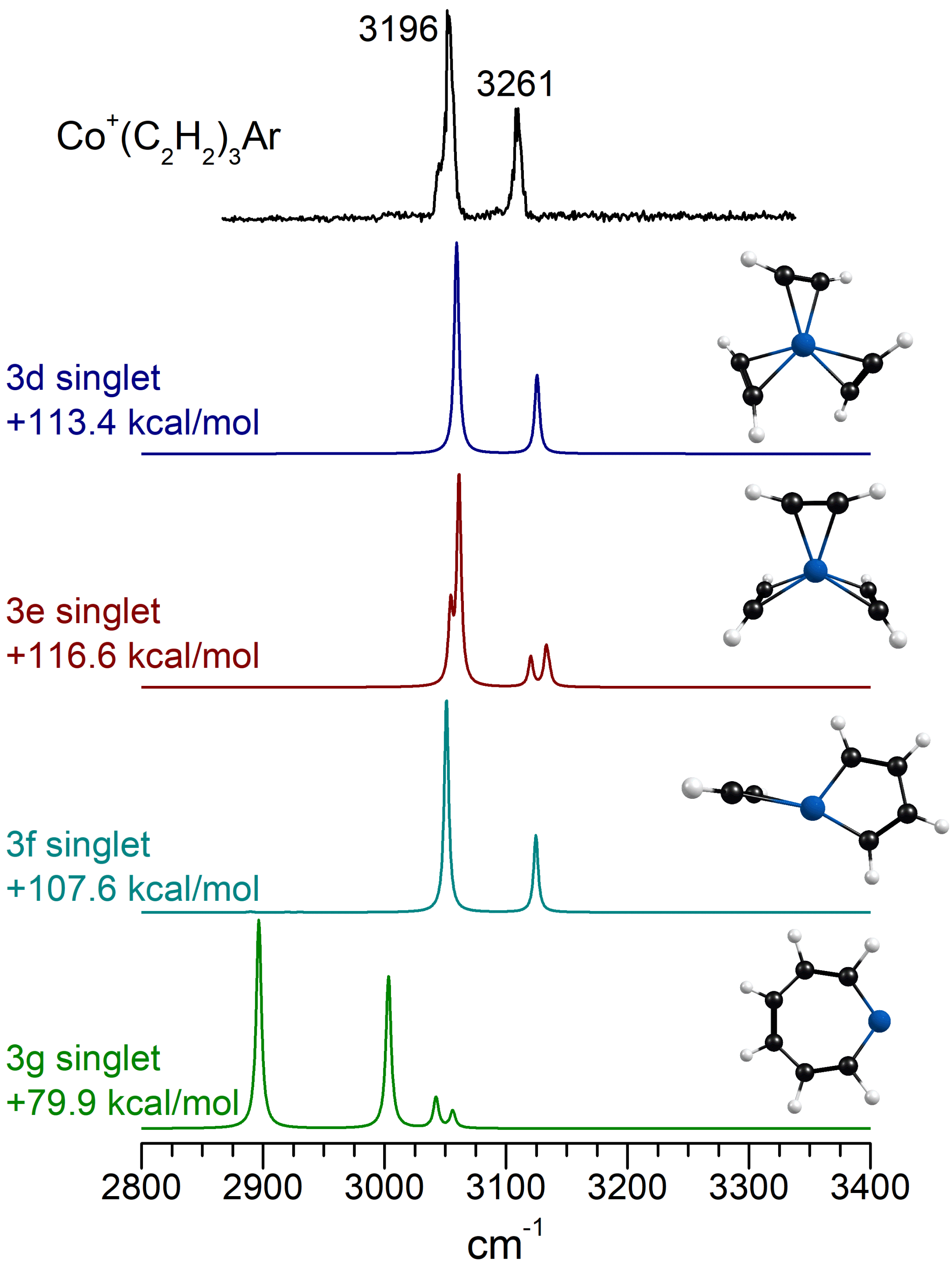

Figure S36. The experimental spectrum for $Co^{+}(C_2H_2)_3Ar$ with simulated spectra for $Co^{+}(C_2H_2)_3$ as a singlet and predicted isomers 3e-3g of singlet-$Co^{+}(C_2H_2)_3$.

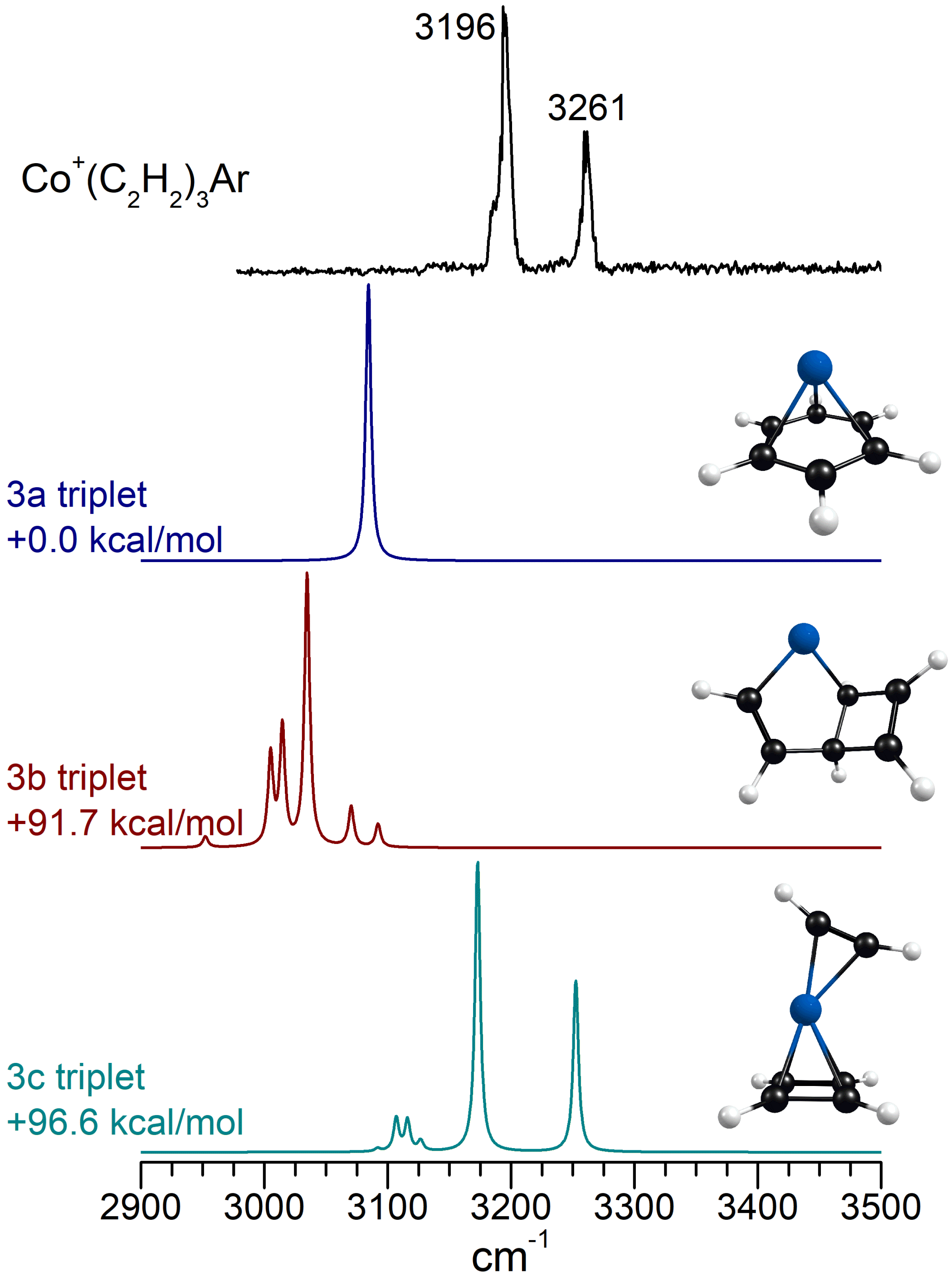

Figure S37. The experimental spectrum for $Co^+(C_2H_2)_3Ar$ with simulated spectra for $Co^+(C_2H_2)_3$ as a triplet and predicted isomers 3a-3c of singlet-$Co^+(C_2H_2)_3$.

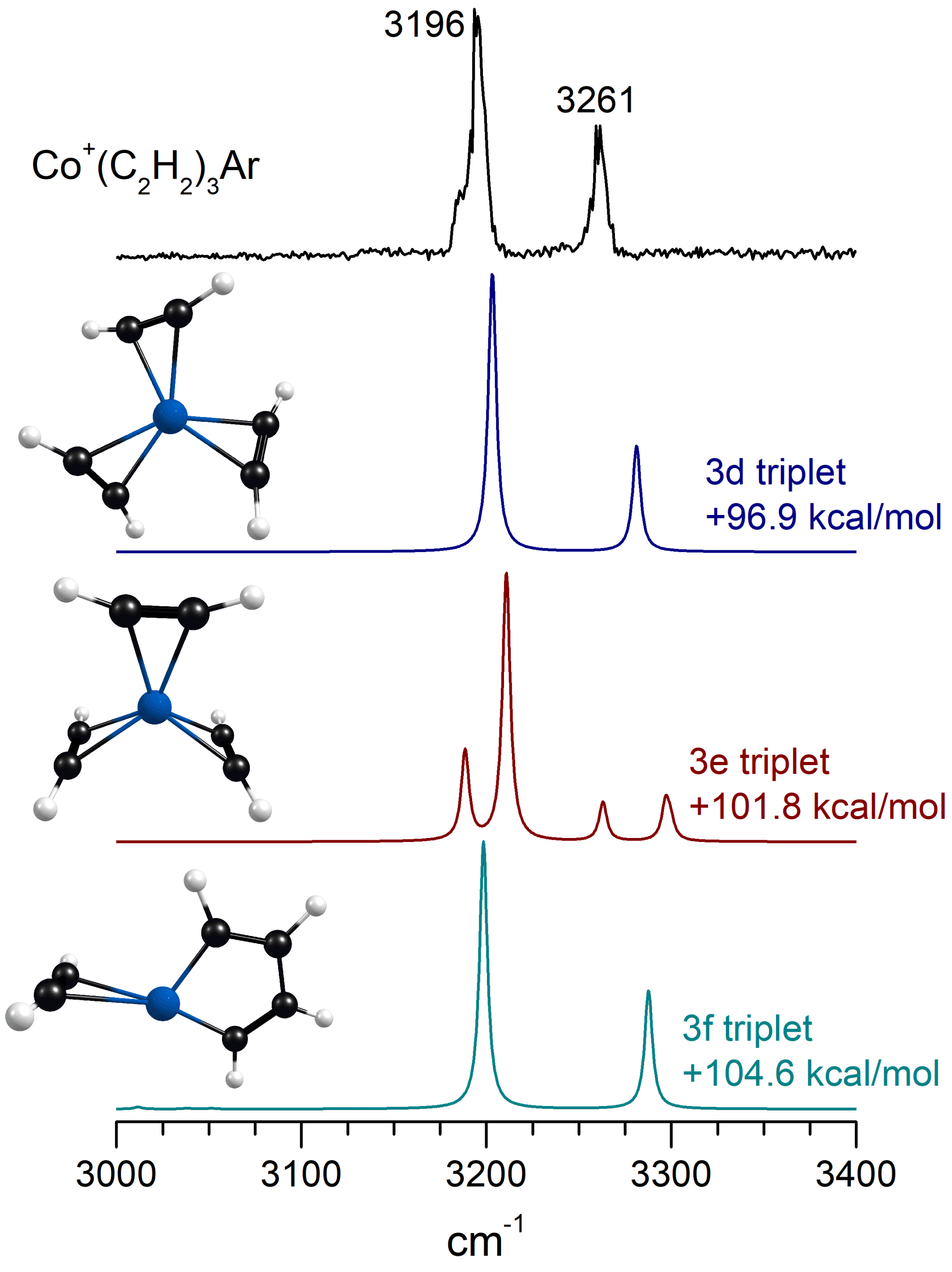

Figure S38. The experimental spectrum for $Co^+(C_2H_2)_3Ar$ with simulated spectra for $Co^+(C_2H_2)_3$ as a triplet and predicted isomers 3d-3f of triplet-$Co^+(C_2H_2)_3$.

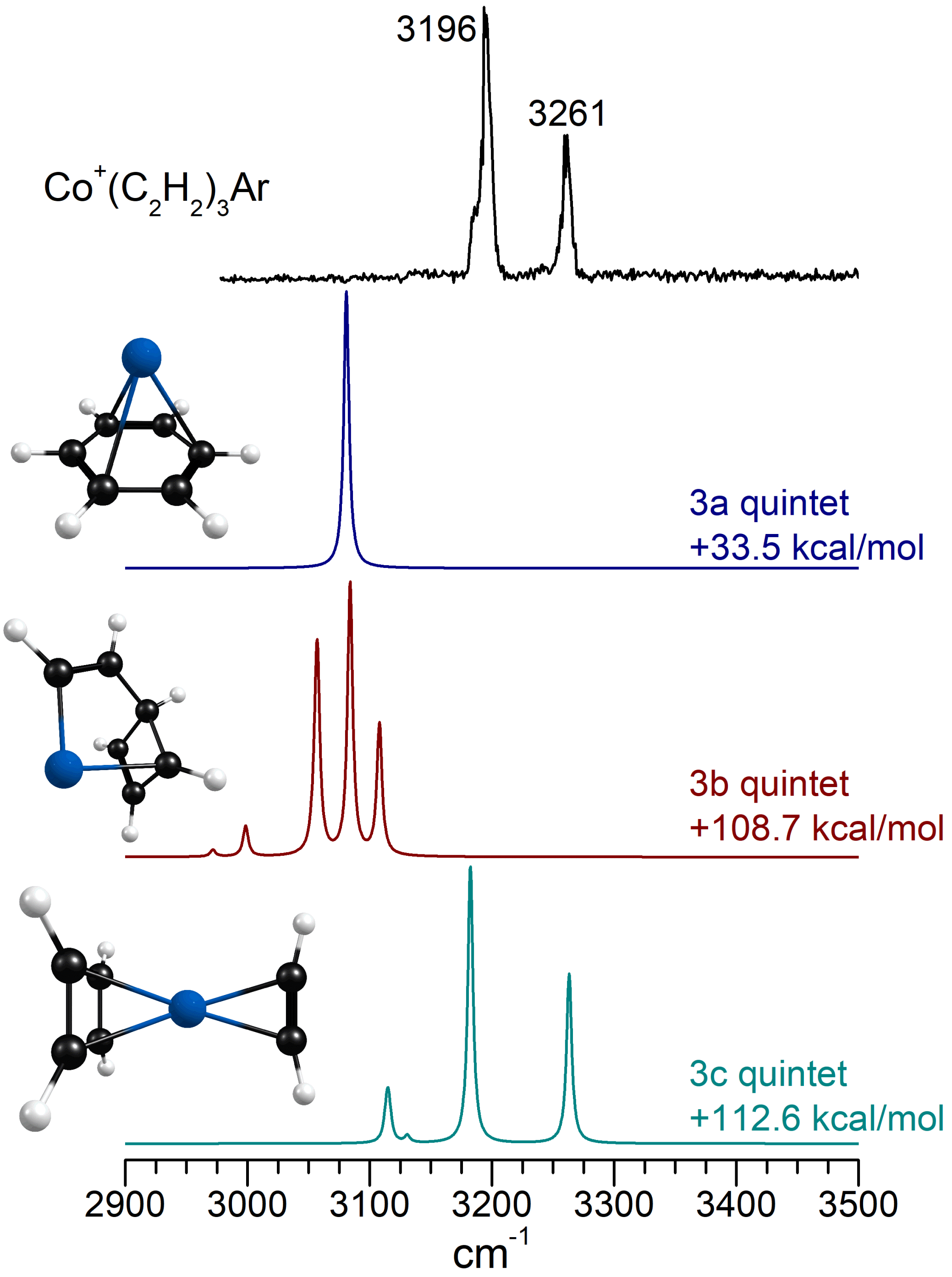

Figure S39. The experimental spectrum for $Co^{+}(C_2H_2)_3Ar$ with simulated spectra for $Co^{+}(C_2H_2)_3$ as a quintet and predicted isomers 3a-3c of quintet-$Co^{+}(C_2H_2)_3$.

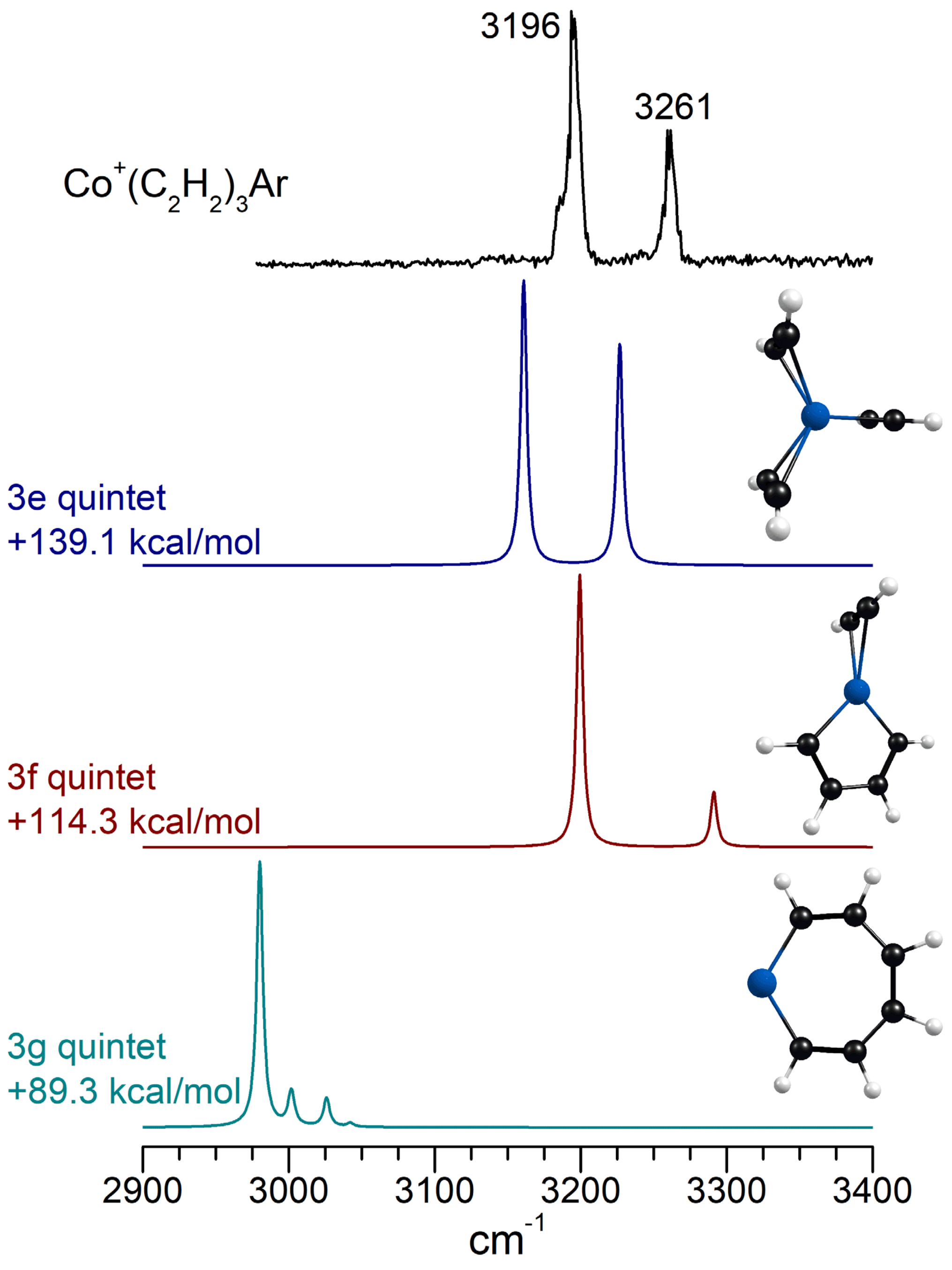

Figure S40. The experimental spectrum for $Co^{+}(C_2H_2)_3Ar$ with simulated spectra for $Co^{+}(C_2H_2)_3$ as a quintet and predicted isomers 3e-3g of quintet-$Co^{+}(C_2H_2)_3$.

Table S26. $Co^+(C_2H_2)_4$ calculated at the B3LYP/Def2TZVP level of theory using Gaussian16.

Isomer	2s + 1	E (hartree)	Relative E (kcal/mol)
4a	1	-1692.183384	+11.9
4a	3	-1692.202361	+0.0
4a	5	-1692.117459	+53.3
4b	1	-1692.149197	+33.4
4b	3	-1692.171998	+19.1
4b	5	-1692.101993	+63.0
4c	1	-1692.122521	+50.1
4c	3	-1692.14641	+35.1
4d	1	-1692.053779	+97.2
4d	3	-1692.064705	+86.4
4d	5	-1692.035337	+104.8
4e	1	-1692.056061	+91.8
4e	3	-1692.049799	+95.7
4e	5	-1692.001941	+125.8
4f	1	-1692.030755	+107.7
4f	3	-1692.046167	+98.0
4f	5	-1692.017435	+116.0
4g	1	-1692.02896	+108.8
4g	3	-1692.027475	+109.7
4g	5	-1692.006778	+122.7
4h	1	-1692.029981	+108.2
4h	3	-1692.023295	+112.4
4h	5	-1691.98703	+135.1
4i	1	-1691.999847	+141.9
4i	3	-1692.003991	+124.5
4i	5	-1691.986279	+135.6
4j	1	-1691.976264	+141.9
4j	3	-1692.002605	+125.3
4j	5	-1691.934212	+168.3
4k	3	-1692.00141	+126.1
4k	5	-1691.990459	+133.0
4l	1	-1691.970757	+145.3
4l	3	-1691.998233	+128.1
4m	1	-1691.972646	+144.1
4m	3	-1691.997246	+128.7
4n	1	-1691.971204	+145.1
4n	3	-1691.995089	+130.1
4n	5	-1691.934213	+168.3
4o	5	-1692.054897	+92.5
4p	5	-1692.046826	+97.6

Table S27. Cartesian coordinates for the optimized geometry of isomer 4a-singlet $Co^{+}(C_2H_2)_4$ followed by its predicted frequencies (cm^{-1}) and IR intensities (km/mol).

Z	x	y	z
6	1.203673000	0.704823000	1.210220000
6	1.242148000	1.410676000	-0.000145000
6	1.203675000	-0.704567000	1.210366000
1	1.245252000	2.491970000	-0.000261000
1	1.169035000	-1.239133000	2.149736000
6	1.203680000	0.704568000	-1.210363000
6	1.242152000	-1.410674000	0.000149000
1	1.169045000	1.239135000	-2.149733000
1	1.245261000	-2.491968000	0.000264000
6	1.203682000	-0.704821000	-1.210217000
1	1.169054000	-1.239584000	-2.149476000
1	1.169036000	1.239585000	2.149479000
27	-0.605773000	-0.000001000	-0.000001000
1	-2.868114000	-1.599021000	-0.000008000
6	-2.405720000	-0.628779000	-0.000004000
1	-2.868112000	1.599020000	-0.000002000
6	-2.405720000	0.628777000	0.000001000

Frequency	Intensity	Frequency	Intensity	Frequency	Intensity
41.1871	0	769.2095	53.9111	1338.3267	0.0215
97.7166	1.4615	787.2705	75.6258	1386.6765	0
98.67	1.2633	826.6833	55.6243	1497.6768	23.5704
176.5206	0.2954	922.9207	0.1003	1506.2779	22.1106
205.4274	1.8338	932.287	0.0163	1582.6017	0.2807
215.685	5.1381	998.6046	0.6854	1591.2873	0
398.2733	0	1012.8754	0	1754.0791	26.5936
401.156	3.0678	1015.5394	0.2551	3199.475	0.0056
441.1506	9.4229	1024.4747	0.0356	3204.9047	0
550.1	0.0025	1040.1854	1.5863	3205.4402	0.0562
614.0801	0.0857	1043.9888	0.1	3214.0539	7.7829
614.0889	0.0001	1054.877	1.5575	3215.136	7.9568
685.3377	0.0279	1190.5369	0.0001	3220.3815	0.0323
745.713	0	1194.204	0	3275.3062	72.5428
759.7548	1.7673	1197.5997	0.0252	3335.0018	52.2766

Table S28. Cartesian coordinates for the optimized geometry of isomer 4b-singlet $Co^+(C_2H_2)_4$ followed by its predicted frequencies (cm^{-1}) and IR intensities (km/mol).

Z	x	y	z
6	0.690420000	-1.492967000	-0.162864000
6	1.557490000	-0.668693000	-1.022836000
6	1.557468000	0.668726000	-1.022846000
6	-1.557001000	0.668683000	-1.023470000
6	-0.690269000	1.492991000	-0.163195000
6	0.690373000	1.492983000	-0.162884000
1	-1.176849000	2.317304000	0.354285000
1	1.176796000	-2.317247000	0.354858000
1	2.298174000	-1.204177000	-1.607464000
1	2.298134000	1.204226000	-1.607481000
1	-2.297507000	1.204136000	-1.608351000
1	1.176724000	2.317284000	0.354829000
27	-0.000330000	0.000010000	1.240064000
1	-1.176777000	-2.317335000	0.354316000
6	-0.690221000	-1.493015000	-0.163176000
6	-1.556979000	-0.668745000	-1.023462000
1	-2.297467000	-1.204231000	-1.608336000

Frequency	Intensity	Frequency	Intensity	Frequency	Intensity
130.7922	1.9408	836.1678	4.1243	1412.805	17.3389
191.3893	0.5275	914.4932	0	1428.7627	0.8414
230.8525	3.5473	918.5382	5.0921	1459.7901	0
242.1832	0	950.3668	0.0034	1546.7281	0.9967
267.2778	0.2938	970.9913	0.2371	1551.3635	2.9642
330.0553	0	993.0755	0	1666.9828	3.8436
355.7919	44.2062	1007.7545	3.886	1678.5167	8.7252
360.9218	2.5801	1021.7783	24.4665	3116.6763	2.7939
427.6657	0.048	1026.6693	0	3116.7117	0
635.6312	11.1324	1034.1292	0	3131.2091	0.0212
660.673	0	1208.2759	0.8204	3132.0172	0.0038
695.0299	4.3343	1225.0873	0.0687	3158.3484	0
740.4763	36.3356	1228.8077	5.8894	3158.9188	1.4024
802.7597	28.0128	1238.5634	0.1325	3174.124	1.9697
810.5172	52.5729	1377.5216	0	3174.4295	1.0998

Table S29. Cartesian coordinates for the optimized geometry of isomer 4c-singlet $Co^{+}(C_2H_2)_4$ followed by its predicted frequencies (cm^{-1}) and IR intensities (km/mol).

Z	x	y	z
27	-0.322488000	1.097128000	-0.040906000
6	1.674907000	0.298262000	0.671852000
1	2.142588000	0.957803000	1.392922000
6	1.674702000	0.247311000	-0.692489000
1	2.142103000	0.851275000	-1.460883000
6	-0.414579000	-0.714864000	1.467397000
6	-1.561495000	-0.548063000	0.750947000
6	0.917289000	-1.019103000	0.834429000
1	-2.492831000	-0.342802000	1.262864000
1	1.453899000	-1.830349000	1.327116000
6	-1.561611000	-0.602836000	-0.707902000
6	0.917159000	-1.078584000	-0.756134000
1	-2.493036000	-0.436446000	-1.233578000
1	1.453899000	-1.924240000	-1.186931000
6	-0.414775000	-0.822807000	-1.409986000
1	-0.444675000	-0.821286000	-2.493749000
1	-0.444366000	-0.632302000	2.548017000

Frequency	Intensity	Frequency	Intensity	Frequency	Intensity
100.9865	0.3967	918.0571	0.4416	1311.0921	2.5826
160.8117	0.3215	953.6372	12.3243	1322.4783	5.2865
216.8773	1.6109	953.817	9.2193	1397.8079	8.7969
220.3876	0.675	977.2274	0.491	1442.191	3.2733
301.9258	0.104	985.4786	3.4114	1523.6258	0.0642
420.6116	0.436	987.7197	0.6735	1551.1701	0.9946
432.228	4.666	1007.9906	0.0602	1619.4697	2.268
547.8525	0.0691	1020.6139	0.8771	3082.914	0.7447
557.3884	0.7332	1041.7013	5.4212	3088.2414	2.5987
590.5951	0.5216	1107.0084	1.8646	3172.2595	0.8818
763.8117	11.8193	1155.9355	5.4319	3173.8019	0.4248
780.3041	44.0676	1196.2346	0.4335	3179.3228	0.9578
818.9268	3.1265	1218.344	2.3654	3191.2958	0.3053
834.7986	46.2785	1218.8287	3.2948	3199.8493	2.1345
850.6998	0.3467	1285.7007	10.3617	3202.0198	1.5014

Table S30. Cartesian coordinates for the optimized geometry of isomer 4d-singlet $Co^{+}(C_2H_2)_4$ followed by its predicted frequencies (cm^{-1}) and IR intensities (km/mol).

Z	x	y	z
6	-1.382141000	0.314862000	1.018413000
6	-0.779322000	1.316791000	0.000002000
6	-1.382191000	0.314887000	-1.018400000
6	-2.087136000	-0.366259000	0.000010000
1	-1.478031000	0.341791000	2.094332000
1	-1.248618000	2.300310000	0.000025000
1	-1.478072000	0.341778000	-2.094321000
1	-2.859158000	-1.123073000	0.000017000
27	-0.000006000	-0.840177000	-0.000003000
6	0.779335000	1.316787000	-0.000018000
1	1.248633000	2.300305000	-0.000035000
6	1.382180000	0.314884000	1.018406000
6	1.382168000	0.314853000	-1.018410000
1	1.478082000	0.341771000	-2.094326000
1	1.478038000	0.341800000	2.094326000
6	2.087133000	-0.366268000	0.000007000
1	2.859124000	-1.123115000	0.000016000

Frequency	Intensity	Frequency	Intensity	Frequency	Intensity
164.3833	1.5066	881.3718	1.7812	1215.5889	0
220.5818	0	902.2246	8.0397	1220.3584	1.4828
269.5431	0.2458	913.8624	12.0109	1313.0062	0.4505
309.6647	5.4642	925.2843	0	1370.4095	14.4391
321.597	0.066	942.6244	18.6958	1376.2994	0
360.4362	6.9387	963.2828	3.8056	1404.8896	2.9115
370.2504	5.5832	968.1014	7.6804	1411.6956	4.8058
487.3104	0	995.0438	0	3091.682	0.1255
504.7173	1.0454	999.568	23.6014	3100.1911	0.0002
736.8849	1.6893	1014.1405	5.3665	3207.7697	0.6269
754.6056	23.079	1063.3452	28.2267	3209.0238	1.6466
803.3948	0.2086	1108.2271	0	3221.1565	0.0001
823.7584	11.9677	1147.7406	15.8161	3222.4006	12.1371
830.5547	0	1176.795	5.2482	3232.2424	11.6125
849.4634	6.0092	1211.76	1.8963	3233.5066	1.174

Table S31. Cartesian coordinates for the optimized geometry of isomer 4e-singlet $Co^+(C_2H_2)_4$ followed by its predicted frequencies (cm^{-1}) and IR intensities (km/mol).

Z	x	y	z
6	1.755093000	1.038573000	0.000281000
6	1.840966000	-0.000272000	1.011968000
6	1.755118000	-1.038563000	-0.000255000
6	1.841020000	0.000281000	-1.011938000
1	1.885730000	2.107138000	0.000570000
1	1.974845000	-0.000554000	2.083835000
1	1.885767000	-2.107127000	-0.000533000
1	1.974972000	0.000566000	-2.083795000
27	-0.000001000	-0.000010000	-0.000028000
1	-1.885760000	-2.107128000	-0.000428000
6	-1.755119000	-1.038563000	-0.000203000
6	-1.841014000	0.000224000	-1.011940000
1	-1.974947000	0.000451000	-2.083800000
6	-1.755099000	1.038571000	0.000226000
1	-1.885712000	2.107140000	0.000454000
6	-1.840967000	-0.000214000	1.011967000
1	-1.974847000	-0.000439000	2.083833000

Frequency	Intensity	Frequency	Intensity	Frequency	Intensity
78.1363	0	804.655	0	1205.6801	0
87.2486	0.9496	839.6694	0	1247.4025	5.321
135.6747	4.072	857.6686	0.0346	1254.9259	0
312.1736	0	874.7151	9.2403	1309.6916	0
329.6532	25.6188	879.0062	0	1316.4795	57.7135
340.4793	6.8511	912.6543	0	1362.7285	12.6169
343.5732	0	928.2904	0	1366.4226	0
376.3458	0	933.9111	3.5324	3226.5113	0.3483
428.1802	27.007	943.7648	24.2963	3228.7121	0
478.5757	0	956.0958	0	3233.2361	0
548.5154	0	960.3985	29.8277	3233.9014	16.1877
550.1036	16.1049	967.397	0	3268.3953	0
718.1496	0	1097.2172	0	3268.7821	34.9686
776.8506	7.1455	1150.1096	0	3272.5254	22.387
789.2049	45.9874	1205.2715	0	3274.2542	0

Table S32. Cartesian coordinates for the optimized geometry of isomer 4f-singlet $Co^{+}(C_2H_2)_4$ followed by its predicted frequencies (cm^{-1}) and IR intensities (km/mol).

Z	x	y	z
6	-1.023299000	-0.758189000	1.058451000
6	-1.934731000	0.281909000	0.352557000
6	-1.630215000	-0.601400000	-0.869972000
6	-0.995753000	-1.575861000	-0.094768000
1	-0.761485000	-0.902864000	2.096611000
1	-2.974615000	0.266490000	0.676565000
1	-1.920151000	-0.522682000	-1.908963000
1	-0.610601000	-2.566143000	-0.305618000
27	0.572657000	-0.149676000	-0.350780000
6	-1.309195000	1.636608000	0.236701000
1	-1.883327000	2.549526000	0.368721000
6	-0.012946000	1.609046000	-0.064202000
1	0.673183000	2.446169000	-0.109695000
6	2.167501000	-0.302746000	0.874185000
6	2.524847000	0.181934000	-0.202676000
1	3.139665000	0.620312000	-0.965542000
1	2.158333000	-0.677358000	1.877341000

Frequency	Intensity	Frequency	Intensity	Frequency	Intensity
84.6278	0.6161	720.3586	40.1125	1213.216	1.0734
96.0587	1.6465	742.9664	18.2644	1255.1306	29.2904
154.9454	1.0686	750.6621	58.4081	1315.9558	0.1622
172.0241	3.6187	780.8405	6.5712	1351.3502	13.4291
219.5368	0.9855	810.1838	4.6872	1422.0786	5.187
250.8654	1.0097	886.0894	16.4811	1584.1281	1.6793
299.0975	3.2601	900.6486	4.4616	1863.929	25.7237
337.6328	32.7266	910.0119	10.543	3105.8457	0.9258
413.6126	5.036	936.3146	17.3563	3147.1766	1.6266
427.6502	7.9853	960.5446	4.056	3189.2378	3.3417
453.6031	2.2614	998.058	0.8332	3190.2835	1.0028
529.5637	3.3643	1011.1103	9.8368	3219.3959	9.184
685.5467	4.0939	1073.1616	3.226	3228.1784	4.3198
688.7234	8.8363	1143.7533	11.1656	3305.6141	122.9776
712.1448	29.3543	1161.9286	11.9562	3385.1947	70.7415

Table S33. Cartesian coordinates for the optimized geometry of isomer 4g-singlet $Co^{+}(C_2H_2)_4$ followed by its predicted frequencies (cm^{-1}) and IR intensities (km/mol).

Z	x	y	z
6	-2.472156000	-0.706353000	0.276504000
6	-2.472155000	0.706357000	0.276495000
6	-1.321071000	-1.293929000	-0.234877000
1	-3.335069000	1.274937000	0.612457000
1	-1.324285000	-2.357528000	-0.461720000
6	-1.321065000	1.293924000	-0.234883000
1	-1.324271000	2.357520000	-0.461737000
1	-3.335067000	-1.274929000	0.612482000
27	0.137223000	-0.000002000	-0.363368000
6	1.789580000	1.034755000	0.300601000
6	2.253341000	-0.000113000	-0.589693000
1	2.745194000	-0.000229000	-1.552230000
1	1.892753000	2.105248000	0.347260000
1	1.082756000	0.000236000	2.278891000
6	1.420747000	0.000120000	1.253032000
1	1.892595000	-2.105244000	0.347673000
6	1.789504000	-1.034752000	0.300797000

Frequency	Intensity	Frequency	Intensity	Frequency	Intensity
30.4998	0.0649	788.3371	0.121	1256.9219	6.2776
56.2962	0.4122	805.333	42.0125	1259.2802	0.3534
134.7796	1.7777	864.4308	4.9542	1272.5077	33.0447
164.4329	14.6006	892.8006	5.6139	1331.41	26.6572
285.8017	11.7216	895.6855	13.7503	1377.7641	6.8322
291.2182	6.8005	944.8599	2.9208	1468.8726	51.8193
340.0755	2.0182	948.9804	22.939	1489.3659	14.5115
367.4553	2.1975	965.2188	39.598	3133.1362	0.0209
405.2416	12.4441	967.0687	13.1348	3138.435	0.1373
459.7706	0.3078	1000.4758	11.1933	3153.1738	0.0505
512.9696	1.2958	1042.3667	13.8151	3163.7961	1.3325
533.5478	16.0125	1103.4543	5.0306	3222.2477	2.0101
684.5405	55.5053	1124.8339	2.5783	3233.6834	9.3699
720.3924	19.2183	1153.9109	2.7547	3270.5894	19.9057
757.1395	5.1186	1210.9055	0	3275.2291	10.8935

Table S34. Cartesian coordinates for the optimized geometry of isomer 4h-singlet $Co^+(C_2H_2)_4$ followed by its predicted frequencies (cm^{-1}) and IR intensities (km/mol).

Z	x	y	z
6	1.652711000	0.605033000	-0.616096000
6	1.747985000	0.221320000	0.765889000
6	1.402013000	-1.131564000	0.423669000
6	1.293911000	-0.760158000	-0.965798000
1	1.887722000	1.491469000	-1.181906000
1	2.007843000	0.729422000	1.681311000
1	1.360770000	-2.059731000	0.968380000
1	1.148630000	-1.294299000	-1.890567000
27	-0.170130000	0.114182000	0.051551000
6	-0.569502000	2.054621000	0.137152000
6	-1.650524000	1.459627000	0.033694000
1	-2.710879000	1.312688000	0.006606000
1	0.102846000	2.881903000	0.239434000
1	-2.312571000	-1.078302000	-1.438262000
6	-1.782731000	-1.263939000	-0.529403000
6	-1.377994000	-1.664389000	0.545500000
1	-1.186051000	-2.189368000	1.455478000

Frequency	Intensity	Frequency	Intensity	Frequency	Intensity
36.2011	0.3148	699.3928	15.9395	1201.7026	0.2312
61.1539	0.4012	733.4587	6.0983	1212.0468	0.0126
138.1394	1.1726	761.9441	32.9304	1284.0849	1.345
149.3231	1.5491	771.2824	67.3837	1371.7663	14.5955
198.1269	3.3162	782.6546	17.485	1382.8067	10.0996
209.9871	1.5441	800.932	2.2059	1850.6035	11.6411
262.9247	6.7977	823.3739	2.9899	1957.7613	2.3349
293.4705	3.8389	840.4092	15.0728	3236.9787	0.8297
348.01	7.1027	852.778	22.3473	3251.9089	10.2715
379.4176	12.0734	867.3269	0.3396	3259.552	5.5025
398.771	11.6944	897.7954	1.6547	3270.8569	6.2688
471.5073	29.6337	948.7438	2.4655	3316.5702	67.9029
513.0392	35.2927	960.5947	1.0048	3354.0212	133.143
587.071	0.4789	969.6458	7.8674	3386.3276	43.1421
657.9982	3.3098	979.0839	4.8504	3439.7896	41.9107

Table S35. Cartesian coordinates for the optimized geometry of isomer 4i-singlet $Co^+(C_2H_2)_4$ followed by its predicted frequencies (cm^{-1}) and IR intensities (km/mol).

Z	x	y	z
27	0.000236000	0.000392000	-0.341231000
6	1.023476000	-0.986346000	0.800688000
6	2.352397000	-0.789365000	0.499480000
1	3.141317000	-1.454705000	0.838173000
1	0.655497000	-1.701436000	1.527243000
6	2.619711000	0.390112000	-0.255677000
6	1.518306000	1.199390000	-0.441109000
1	1.597000000	2.238873000	-0.747196000
1	3.616195000	0.620271000	-0.623858000
1	-1.594883000	-2.238390000	-0.748913000
6	-1.517188000	-1.199025000	-0.442128000
6	-2.619696000	-0.391462000	-0.255628000
1	-3.616196000	-0.623211000	-0.622731000
6	-2.353389000	0.788395000	0.499210000
1	-3.142859000	1.452898000	0.838255000
6	-1.024503000	0.986991000	0.799608000
1	-0.657138000	1.702985000	1.525594000

Frequency	Intensity	Frequency	Intensity	Frequency	Intensity
23.6119	1.4297	745.8685	7.5935	1229.8453	28.2337
45.0375	1.8792	774.0781	24.1153	1291.0888	5.5119
120.8209	0.1228	803.448	1.422	1298.1534	0.9404
196.6857	2.6761	809.0326	0.039	1461.2127	0.4725
210.4487	0.4668	927.6425	13.4427	1461.5532	0.0537
323.8601	1.7807	942.0539	3.5905	1483.1177	66.9007
329.2431	25.5648	960.7988	2.6977	1484.0767	19.915
346.2754	2.009	962.5224	22.9223	3139.9328	0.337
354.4635	6.6863	1032.622	1.1008	3140.0841	0.0158
432.4194	0.0571	1032.7253	1.267	3156.6041	2.4454
473.7332	3.2859	1083.5244	2.8454	3156.8289	0.2429
594.5016	9.3542	1083.7841	1.3427	3162.8973	1.8849
628.2833	5.1565	1100.216	1.6418	3162.9561	0.0517
676.6661	17.1346	1101.4349	0.1323	3187.2675	1.3557
689.9747	62.9093	1211.7299	10.2384	3187.7917	1.394

Table S36. Cartesian coordinates for the optimized geometry of isomer 4j-singlet $Co^+(C_2H_2)_4$ followed by its predicted frequencies (cm^{-1}) and IR intensities (km/mol).

Z	x	y	z
27	0.843496000	-0.000020000	0.000009000
6	-0.687555000	1.436890000	0.265097000
6	0.305356000	2.028238000	-0.140386000
1	0.984330000	2.780757000	-0.481429000
1	-1.678593000	1.180972000	0.585123000
6	0.305260000	-2.028210000	0.140572000
6	-0.687641000	-1.436837000	-0.264906000
1	-1.678673000	-1.180885000	-0.584931000
1	0.984200000	-2.780741000	0.481658000
6	2.833963000	-0.531570000	-0.306507000
6	2.834015000	0.531521000	0.306281000
1	3.145283000	1.417455000	0.819316000
1	3.145141000	-1.417514000	-0.819580000
6	-4.071338000	0.285789000	-0.526967000
6	-4.071415000	-0.285746000	0.526781000
1	-4.120091000	-0.795793000	1.460365000
1	-4.119874000	0.795844000	-1.460554000

Frequency	Intensity	Frequency	Intensity	Frequency	Intensity
23.4425	0.0497	401.5874	4.5572	799.6985	0.0436
49.9987	0.2195	413.8658	3.5971	806.6147	1.9463
53.4314	0.0105	642.6409	0.0076	836.7216	3.7342
72.8026	2.5977	671.001	0.637	1903.6291	5.0242
92.0275	0.9351	688.2535	0.3294	1914.9243	1.3751
103.3323	0.0528	692.0537	5.9885	1916.4272	6.883
104.1939	1.0296	698.1174	0.6907	2061.6516	9.7333
121.8492	1.2827	726.2353	33.3035	3305.7823	59.0835
125.1081	0.7519	735.254	13.7748	3311.7769	410.0901
154.4649	1.9523	749.8684	0.8674	3332.1689	121.25
166.7403	5.1852	778.3873	65.9817	3395.3147	119.6643
258.1834	2.2581	779.4874	25.0821	3402.4294	86.0064
261.7709	2.0815	782.8916	96.807	3404.6738	43.271
267.9226	0.5228	786.1192	115.7012	3410.7963	36.4625
398.747	6.7153	792.0948	153.6381	3495.1126	1.3071

Table S37. Cartesian coordinates for the optimized geometry of isomer 4l-singlet $Co^{+}(C_2H_2)_4$ followed by its predicted frequencies (cm^{-1}) and IR intensities (km/mol).

Z	x	y	z
6	-0.607808000	1.242947000	1.668481000
6	0.608112000	1.242873000	1.668417000
1	1.641594000	1.383848000	1.891512000
1	-1.641253000	1.384037000	1.891677000
27	-0.000011000	-0.069528000	0.000009000
6	-1.467624000	-1.694715000	-0.000006000
6	-2.097807000	-0.650334000	0.000006000
1	-2.898711000	0.056203000	0.000014000
1	-1.197786000	-2.727216000	-0.000017000
6	2.097641000	-0.650805000	-0.000007000
6	1.467219000	-1.695043000	-0.000004000
1	1.197161000	-2.727487000	-0.000010000
1	2.898719000	0.055535000	-0.000004000
1	1.641598000	1.383882000	-1.891481000
6	0.608112000	1.242889000	-1.668416000
6	-0.607807000	1.242933000	-1.668507000
1	-1.641255000	1.383983000	-1.891712000

Frequency	Intensity	Frequency	Intensity	Frequency	Intensity
37.7493	0.6399	338.8658	0.0628	791.7346	6.1906
41.3854	0	342.4537	8.5805	801.7833	0.2391
68.9705	1.4232	644.0978	0	818.4423	2.0505
79.0924	0	648.2958	0	1939.3519	9.1733
116.2841	0.0004	654.6165	0.0286	1939.8755	8.736
131.6773	1.5404	669.8669	6.4075	1959.9351	2.468
155.8348	0	696.5753	6.4477	1965.469	1.8524
158.9756	0.3495	704.33	0	3352.6223	31.8659
175.4806	0.5485	725.2543	1.2088	3356.3701	124.2048
183.1421	0.1318	729.4103	23.996	3368.0642	0.0017
203.7186	1.4251	741.5613	35.1811	3368.8998	225.2131
212.5639	5.1167	755.759	124.4159	3434.8708	54.7611
222.6763	1.2824	756.4362	158.269	3438.0186	43.0551
329.2556	4.7228	761.2915	0	3452.3846	43.0977
332.9329	0	774.7903	0.0064	3453.7426	20.4586

Table S38. Cartesian coordinates for the optimized geometry of isomer 4m-singlet $Co^+(C_2H_2)_4$ followed by its predicted frequencies (cm^{-1}) and IR intensities (km/mol).

Z	x	y	z
27	-0.159892000	0.137779000	0.059067000
6	0.869462000	1.878266000	-0.745217000
6	-0.301244000	1.928011000	-1.086158000
1	-1.250667000	2.189054000	-1.502872000
1	1.902434000	2.093660000	-0.581028000
6	0.829458000	-0.091845000	2.141360000
6	1.787637000	-0.069281000	1.404991000
1	2.716270000	-0.082025000	0.881537000
1	0.100097000	-0.131533000	2.919272000
6	-2.181641000	0.032909000	0.678258000
6	-2.017635000	-0.991972000	0.036702000
1	-2.096551000	-1.921042000	-0.484391000
1	-2.584485000	0.836401000	1.259034000
6	0.826882000	-2.007860000	-0.864467000
6	0.820055000	-1.231143000	-1.784532000
1	0.857105000	-0.628575000	-2.663009000
1	0.875034000	-2.758483000	-0.108969000

Frequency	Intensity	Frequency	Intensity	Frequency	Intensity
41.127	0.19	340.9851	2.1279	783.5982	46.058
53.4249	0.0691	357.5838	3.7488	794.9963	10.2943
69.2321	0.1268	646.3648	1.3528	803.1511	67.104
79.969	0.3049	659.6326	0.0971	1936.7281	13.7547
97.994	0.242	671.6987	0.6639	1939.1447	3.4511
105.4161	0.3344	673.6283	0.5816	2002.6315	0.1991
117.4378	0.5266	677.5644	1.7338	2031.7868	4.3518
126.2642	0.0462	684.1608	0.0773	3340.726	119.0173
144.9226	0.3259	697.6191	14.1729	3346.6108	105.2196
153.1777	0.713	709.7682	16.8997	3373.9062	133.6362
165.8859	1.032	748.2089	34.1068	3382.9007	138.0523
193.3819	1.3257	754.6444	80.8437	3427.728	57.7888
224.1019	4.3002	761.0529	103.4426	3430.3023	32.4713
232.0931	1.6061	769.2136	51.5059	3466.5252	26.9492
252.3715	3.134	778.3157	27.202	3479.657	11.322

Table S39. Cartesian coordinates for the optimized geometry of isomer 4n-singlet $Co^{+}(C_2H_2)_4$ followed by its predicted frequencies (cm^{-1}) and IR intensities (km/mol).

Z	x	y	z
27	0.742932000	0.000011000	0.129815000
6	1.975960000	-0.613802000	-1.400999000
6	1.975950000	0.613743000	-1.401037000
1	2.149065000	1.640858000	-1.646925000
1	2.149088000	-1.640926000	-1.646839000
6	0.593672000	-1.928873000	1.181700000
6	-0.461638000	-1.750217000	0.605606000
1	-1.442145000	-1.725800000	0.172528000
1	1.443025000	-2.258394000	1.741673000
6	0.593681000	1.928898000	1.181656000
6	-0.461643000	1.750231000	0.605588000
1	-1.442160000	1.725812000	0.172533000
1	1.443040000	2.258439000	1.741609000
6	-3.709873000	-0.000007000	-0.042850000
6	-3.369807000	-0.000015000	-1.192372000
1	-3.120648000	-0.000021000	-2.227725000
1	-4.056251000	-0.000004000	0.964394000

Frequency	Intensity	Frequency	Intensity	Frequency	Intensity
21.152	0.1677	335.1113	9.9404	792.8733	91.6577
25.3177	0.473	413.1454	0.5364	794.3543	7.9628
46.697	0.4159	648.1365	0.0093	805.6592	32.412
58.4026	0.0024	664.389	3.0902	1904.1448	12.2008
63.8921	0.3157	664.8035	0.4985	1956.8785	1.2034
75.2249	2.2544	674.2808	10.2908	1963.1286	1.8011
100.3518	1.7026	686.1318	1.4045	2061.3513	7.8396
108.2494	0.221	696.8365	0.1789	3316.4656	12.3781
115.6012	1.2241	704.7297	31.2453	3320.189	465.8198
126.1154	0.8912	728.7243	38.0434	3328.4014	112.9375
141.6154	0.0703	754.3078	96.0164	3394.2476	117.582
184.044	0.086	755.385	60.9376	3407.0607	50.9018
230.5842	0.4777	771.1307	15.9563	3416.7483	38.3121
288.6146	0.133	780.5848	82.8735	3420.1928	18.8456
312.5817	5.4925	782.2384	86.1848	3494.086	1.2855

Table S40. Cartesian coordinates for the optimized geometry of isomer 4o-singlet $Co^+(C_2H_2)_4$ followed by its predicted frequencies (cm^{-1}) and IR intensities (km/mol).

Z	x	y	z
6	-0.856122000	-1.401230000	-0.283491000
6	0.362546000	-1.932347000	-0.733660000
6	1.620487000	-1.614601000	-0.314876000
6	1.620542000	1.614573000	-0.314852000
6	2.068394000	0.668418000	0.697858000
6	2.068366000	-0.668471000	0.697850000
1	2.624187000	1.146862000	1.502303000
1	-1.740684000	-2.043712000	-0.468633000
1	0.287496000	-2.789273000	-1.398881000
1	2.413279000	-2.264341000	-0.679542000
1	2.413343000	2.264320000	-0.679484000
1	2.624136000	-1.146948000	1.502293000
27	-1.685675000	0.000025000	0.359246000
1	-1.740620000	2.043726000	-0.468691000
6	-0.856071000	1.401233000	-0.283519000
6	0.362611000	1.932332000	-0.733666000
1	0.287586000	2.789264000	-1.398881000

Frequency	Intensity	Frequency	Intensity	Frequency	Intensity
64.448	0.4712	747.3621	25.794	1332.3752	1.3026
84.041	0.2042	787.2573	7.2992	1414.5364	14.9925
149.8358	3.4856	818.0077	6.3932	1461.7985	18.7935
158.7457	0.3473	865.5785	12.8432	1474.7734	2.0828
159.0538	1.7148	925.0063	18.2808	1549.353	14.7037
302.9291	3.6954	950.2585	2.1906	1574.818	118.285
372.2592	5.8063	1007.4776	1.7662	1685.7786	7.6635
397.8554	1.7915	1008.6883	1.7885	2919.3961	13.4231
446.5278	3.8145	1013.9669	0.0004	2921.013	16.1252
475.5776	41.2066	1083.4339	0.0047	3111.0167	0.3395
504.7646	11.4589	1087.5418	0.1087	3121.3461	0.4548
546.7445	0.2274	1238.8539	4.6485	3122.3376	0.8561
610.7342	100.4919	1246.122	9.8296	3127.7913	3.1347
683.4589	20.2698	1273.2823	1.1122	3139.5046	2.6854
696.9257	0.3227	1315.0159	4.8083	3140.4533	0.679

Table S41. Cartesian coordinates for the optimized geometry of isomer 4a-triplet $Co^{+}(C_2H_2)_4$ followed by its predicted frequencies (cm^{-1}) and IR intensities (km/mol).

Z	x	y	z
6	1.214852000	1.281142000	0.698347000
6	1.214900000	1.280469000	-0.699493000
6	1.269913000	0.068137000	1.403956000
1	1.163691000	2.214977000	-1.241652000
1	1.263701000	0.068742000	2.485346000
6	1.270058000	0.066788000	-1.403938000
6	1.329813000	-1.144328000	0.705771000
1	1.263966000	0.066357000	-2.485328000
1	1.377494000	-2.078950000	1.248238000
6	1.329901000	-1.144999000	-0.704574000
1	1.377631000	-2.080144000	-1.246138000
1	1.163609000	2.216173000	1.239600000
27	-0.644355000	-0.194363000	-0.000007000
1	-3.164936000	-1.377482000	-0.000022000
6	-2.634830000	-0.444882000	-0.000022000
1	-2.563914000	1.829519000	-0.000014000
6	-2.408547000	0.769109000	-0.000020000

Frequency	Intensity	Frequency	Intensity	Frequency	Intensity
21.6334	0.0057	748.5713	73.1191	1328.2978	0.6084
75.3512	0.9128	759.9704	38.2001	1387.5125	0.0015
85.1206	0.7907	774.8762	66.7367	1502.0603	20.7307
137.0221	3.0449	918.507	0.0299	1503.962	22.1338
154.0584	0.7065	923.3883	0.2983	1592.9245	1.4233
193.4847	0.1156	997.6579	1.4779	1596.1578	0.119
343.6602	31.0449	1010.7178	0.1174	1863.0481	42.7439
408.2892	0.1841	1011.9341	1.0496	3195.0368	0.0528
412.2378	0.1064	1023.7478	0.025	3200.5265	0.0416
441.8504	1.5706	1042.5307	0.0016	3202.5357	0.2266
615.403	0.0071	1046.7542	1.9057	3210.3398	4.8059
618.4188	0.0077	1050.0261	1.843	3212.7462	5.766
675.2926	1.7399	1187.6471	0.0771	3217.6605	0.0187
694.3448	0.0596	1198.1266	0.1125	3306.291	120.2339
732.1485	35.8052	1198.9608	0.0476	3380.6221	70.5891

Table S42. Cartesian coordinates for the optimized geometry of isomer 4b-triplet $Co^+(C_2H_2)_4$ followed by its predicted frequencies (cm^{-1}) and IR intensities (km/mol).

Z	x	y	z
6	0.688889000	-1.512456000	-0.180779000
6	1.560556000	-0.669349000	-1.017151000
6	1.560556000	0.669350000	-1.017150000
6	-1.560411000	0.669352000	-1.017335000
6	-0.688841000	1.512467000	-0.180873000
6	0.688889000	1.512456000	-0.180777000
1	-1.174963000	2.347717000	0.318403000
1	1.174953000	-2.347696000	0.318569000
1	2.315539000	-1.196880000	-1.590885000
1	2.315539000	1.196881000	-1.590884000
1	-2.315350000	1.196874000	-1.591134000
1	1.174953000	2.347696000	0.318572000
27	-0.000099000	-0.000001000	1.253472000
1	-1.174963000	-2.347717000	0.318400000
6	-0.688841000	-1.512467000	-0.180875000
6	-1.560411000	-0.669351000	-1.017335000
1	-2.315351000	-1.196872000	-1.591135000

Frequency	Intensity	Frequency	Intensity	Frequency	Intensity
88.5816	1.3818	838.1001	3.7934	1416.2458	15.8611
179.3333	0.8556	913.9037	6.0305	1430.4227	1.1941
215.301	2.8255	917.1634	0	1461.7465	0
243.0206	0	951.2387	0.0844	1554.6923	1.1197
259.6571	0.8467	965.9378	0.5281	1559.8868	3.7705
321.3796	0	995.6425	0	1663.5547	3.649
345.1132	42.0247	1009.9916	4.1962	1676.1522	8.2437
372.0867	1.9003	1023.9695	22.7395	3120.8407	0
427.5363	0.1833	1027.4162	0	3121.0467	2.2748
642.0099	9.8936	1034.9011	0	3135.2541	0.0754
659.5733	0	1214.6547	1.0452	3135.9032	0.0161
695.831	4.8939	1229.8648	0.061	3156.2903	0
740.1541	42.887	1236.51	5.8343	3157.0079	1.213
798.2799	27.3531	1244.1101	0.0502	3171.937	1.8189
810.4819	49.5801	1381.4807	0	3172.3121	0.8613

Table S43. Cartesian coordinates for the optimized geometry of isomer 4c-triplet $Co^{+}(C_2H_2)_4$ followed by its predicted frequencies (cm^{-1}) and IR intensities (km/mol).

Z	x	y	z
27	-0.317772000	1.126609000	-0.000018000
6	1.671708000	0.263625000	0.682353000
1	2.143418000	0.893885000	1.426911000
6	1.671705000	0.263604000	-0.682365000
1	2.143410000	0.893843000	-1.426944000
6	-0.420271000	-0.795867000	1.444410000
6	-1.558297000	-0.582671000	0.732084000
6	0.913168000	-1.057933000	0.796477000
1	-2.492649000	-0.403043000	1.248258000
1	1.456526000	-1.884191000	1.255904000
6	-1.558299000	-0.582697000	-0.732062000
6	0.913165000	-1.057958000	-0.796446000
1	-2.492651000	-0.403083000	-1.248240000
1	1.456527000	-1.884227000	-1.255847000
6	-0.420275000	-0.795918000	-1.444384000
1	-0.455179000	-0.778419000	-2.527783000
1	-0.455172000	-0.778334000	2.527808000

Frequency	Intensity	Frequency	Intensity	Frequency	Intensity
91.6884	0.1003	919.6492	0.6351	1314.021	2.8359
168.3275	0.362	949.3276	13.9064	1323.7659	4.9252
215.3274	1.4097	953.9667	9.729	1397.9286	8.7334
222.9182	0.9519	978.7571	0.5113	1440.178	3.2604
297.875	0.07	985.1553	3.7911	1525.3674	0.2405
419.4339	0.4069	990.5946	0.6888	1562.2334	1.2988
433.1619	5.5832	1009.6347	0.209	1628.2122	2.1614
548.3341	0.0818	1020.4399	0.7929	3081.9036	0.7837
561.1937	1.1566	1041.1684	5.626	3087.2991	2.732
593.1513	0.5198	1108.4918	1.7142	3172.6536	0.7677
761.1683	12.4241	1156.8208	5.1493	3174.5828	0.4748
776.7579	45.5094	1197.9067	0.3872	3176.8478	0.9344
815.8211	3.4083	1218.7353	2.2208	3190.7176	0.2047
833.7318	42.4142	1219.2771	3.6296	3199.0033	1.8764
846.4709	0.186	1285.6296	10.5169	3199.6191	0.9909

Table S44. Cartesian coordinates for the optimized geometry of isomer 4d-triplet $Co^{+}(C_2H_2)_4$ followed by its predicted frequencies (cm^{-1}) and IR intensities (km/mol).

Z	x	y	z
6	-1.336499000	0.109889000	1.009295000
6	-0.768547000	1.361967000	0.289225000
6	-1.376107000	0.702576000	-0.957166000
6	-2.015417000	-0.276386000	-0.202973000
1	-1.562834000	-0.061314000	2.051725000
1	-1.243185000	2.301993000	0.570175000
1	-1.440183000	1.000357000	-1.993650000
1	-2.762910000	-1.023354000	-0.442747000
27	-0.000036000	-1.007796000	-0.075261000
6	0.768617000	1.361937000	0.289254000
1	1.243278000	2.301932000	0.570267000
6	1.336490000	0.109799000	1.009301000
6	1.376191000	0.702577000	-0.957137000
1	1.440297000	1.000389000	-1.993609000
1	1.562829000	-0.061423000	2.051727000
6	2.015413000	-0.276461000	-0.202976000
1	2.762850000	-1.023478000	-0.442772000

Frequency	Intensity	Frequency	Intensity	Frequency	Intensity
112.1012	2.1826	891.2692	11.1769	1215.0068	0.0005
170.8476	0.174	900.8111	5.5075	1252.5237	1.6737
221.7848	0.5246	908.6128	4.0181	1320.5934	1.9121
244.7864	2.5721	931.2929	0.5608	1342.0753	17.6481
354.6722	0.7398	951.6083	4.92	1352.7353	2.024
357.2052	2.2967	959.5772	15.9774	1434.629	2.7601
409.13	0.0451	959.7148	28.4689	1442.87	2.9621
496.0375	0.1899	999.9039	0.0772	3093.1531	0.339
507.5414	0.7503	1000.7739	9.2073	3102.7149	0.0765
751.625	4.1995	1037.9705	5.5856	3185.684	0.0691
760.8687	28.5617	1102.7833	22.9085	3186.5084	1.3474
796.9277	10.7228	1131.7271	6.56	3219.8513	4.8767
803.4628	11.4278	1154.1945	5.0064	3220.5371	5.9648
825.9204	23.7345	1176.3587	8.9591	3229.4972	7.114
855.9447	11.7048	1213.5327	0.3337	3232.6374	4.2919

Table S45. Cartesian coordinates for the optimized geometry of isomer 4e-triplet $Co^+(C_2H_2)_4$ followed by its predicted frequencies (cm^{-1}) and IR intensities (km/mol).

Z	x	y	z
6	1.454609000	-1.152305000	-0.000083000
6	1.691960000	-0.160918000	-1.032242000
6	1.995871000	0.810655000	0.000035000
6	1.692020000	-0.161066000	1.032197000
1	1.297841000	-2.218697000	-0.000150000
1	1.792158000	-0.193672000	-2.104043000
1	2.371567000	1.823183000	0.000094000
1	1.792222000	-0.193954000	2.103994000
27	-0.000002000	0.352962000	0.000051000
1	-2.371584000	1.823174000	0.000079000
6	-1.995873000	0.810652000	0.000025000
6	-1.692021000	-0.161064000	1.032194000
1	-1.792258000	-0.193955000	2.103988000
6	-1.454597000	-1.152307000	-0.000074000
1	-1.297824000	-2.218698000	-0.000133000
6	-1.691954000	-0.160926000	-1.032245000
1	-1.792159000	-0.193691000	-2.104045000

Frequency	Intensity	Frequency	Intensity	Frequency	Intensity
57.8882	0	813.0025	18.1417	1206.1245	0.0085
113.6363	0.4173	826.8361	0.8916	1258.6537	4.3195
116.5383	1.1206	837.0203	1.7415	1259.6786	0.5486
322.5542	2.9043	861.8994	0.3514	1339.363	30.8237
346.403	0.0006	862.7189	5.6079	1341.9241	0.7289
375.7867	0.0001	939.2517	8.8828	1362.4358	9.9727
375.9571	7.1384	940.2474	2.3607	1366.8384	0
388.8408	19.5249	947.7889	13.7756	3229.2236	6.5792
443.3941	39.5171	952.6096	2.3463	3231.8037	6.4044
556.17	2.805	953.9308	0	3249.266	2.3028
595.7234	5.7765	958.0576	27.1436	3253.7093	13.8072
687.2001	0	965.7819	0.0048	3264.2638	41.1078
740.6809	0	1106.0197	0	3264.72	0.0001
746.6147	14.8692	1177.0398	3.2251	3272.5419	18.6862
802.1433	57.4554	1204.5473	0	3275.0111	1.1777

Table S46. Cartesian coordinates for the optimized geometry of isomer 4f-triplet $Co^{+}(C_2H_2)_4$ followed by its predicted frequencies (cm^{-1}) and IR intensities (km/mol).

Z	x	y	z
6	-1.954391000	-1.233953000	0.664223000
6	-2.155990000	0.023687000	-0.163391000
6	-0.917251000	-0.485896000	-0.948608000
6	-0.892514000	-1.670608000	-0.065678000
1	-2.510317000	-1.657404000	1.490695000
1	-3.098035000	0.027310000	-0.716379000
1	-0.809575000	-0.479382000	-2.032512000
1	-0.315439000	-2.585174000	-0.052004000
27	0.743859000	0.224962000	-0.172923000
6	-1.779985000	1.365286000	0.384558000
1	-2.497663000	2.014978000	0.875854000
6	-0.511108000	1.679507000	0.149645000
1	-0.037155000	2.646557000	0.309951000
6	2.550988000	-0.410349000	0.897205000
6	2.891285000	-0.164465000	-0.242984000
1	3.372014000	0.017299000	-1.182568000
1	2.425768000	-0.677426000	1.926058000

Frequency	Intensity	Frequency	Intensity	Frequency	Intensity
40.96	2.3762	721.0195	9.2913	1202.3028	3.4249
70.0587	1.2458	761.1697	30.7932	1268.8715	38.2439
90.8652	0.082	766.1175	70.8773	1301.5435	15.9514
116.1001	3.116	787.1655	0.4123	1318.7155	21.7442
217.7058	1.1605	872.2996	117.8208	1508.3075	31.4177
233.4967	2.5556	880.487	6.5245	1611.1146	12.6008
263.201	5.2846	912.4686	4.9255	1966.9851	2.106
293.0818	3.2645	932.5035	26.8572	3071.9563	0.2241
329.5024	2.3705	971.5454	22.2416	3115.1339	3.2385
406.5581	9.4209	985.0794	11.1076	3120.559	6.2714
477.0351	1.0226	993.9949	12.6793	3162.3758	0.899
529.556	31.6113	1018.8729	13.616	3197.2223	1.4567
650.551	0.8008	1059.6951	7.1728	3220.8055	0.1235
692.8994	31.3408	1121.0806	3.0616	3331.6743	181.4911
700.8752	53.6413	1181.0203	9.5444	3422.3736	67.2864

Table S47. Cartesian coordinates for the optimized geometry of isomer 4g-triplet $Co^{+}(C_2H_2)_4$ followed by its predicted frequencies (cm^{-1}) and IR intensities (km/mol).

Z	x	y	z
6	-2.408779000	-0.709139000	0.333230000
6	-2.408765000	0.709170000	0.333216000
6	-1.293934000	-1.265651000	-0.258918000
1	-3.245238000	1.291261000	0.708775000
1	-1.282636000	-2.314347000	-0.549609000
6	-1.293910000	1.265649000	-0.258944000
1	-1.282583000	2.314343000	-0.549639000
1	-3.245267000	-1.291205000	0.708793000
27	0.168742000	-0.000022000	-0.477282000
6	1.724913000	1.031914000	0.398397000
6	2.288390000	-0.000068000	-0.437292000
1	2.896013000	-0.000154000	-1.330647000
1	1.810444000	2.103957000	0.454977000
1	0.724660000	0.000184000	2.246181000
6	1.210266000	0.000097000	1.282173000
1	1.810350000	-2.103927000	0.455311000
6	1.724854000	-1.031891000	0.398551000

Frequency	Intensity	Frequency	Intensity	Frequency	Intensity
54.2092	0.2472	766.5031	3.9037	1245.0436	9.8723
79.0843	0.1022	800.7347	46.6406	1260.9467	0.3973
97.1277	0.4503	840.4016	1.035	1310.0264	8.4556
241.0333	7.6515	861.962	2.6098	1339.1811	26.4319
306.088	1.1087	882.0129	7.7746	1371.1342	8.0326
314.5793	12.026	948.8765	4.1034	1484.6918	40.3574
368.7377	1.4356	950.3809	5.6252	1492.9049	9.7636
372.9986	5.7708	963.6189	14.0157	3126.5472	0.7247
409.5288	26.2984	977.2445	4.8437	3129.9712	0.0313
434.261	3.0277	984.1533	11.8608	3155.7368	0.0331
470.9113	9.4681	1036.7274	8.0012	3167.9751	0.5526
527.7529	4.4908	1096.8685	3.8928	3226.5248	2.1078
697.9776	49.7922	1108.7149	2.9442	3240.5108	10.0717
735.8253	8.3124	1129.6488	2.7689	3266.3192	21.3461
738.8147	10.3584	1210.7869	0	3272.3908	9.7458

Table S48. Cartesian coordinates for the optimized geometry of isomer 4h-triplet $Co^{+}(C_2H_2)_4$ followed by its predicted frequencies (cm^{-1}) and IR intensities (km/mol).

Z	x	y	z
6	1.645998000	0.919327000	-0.448208000
6	1.634490000	0.363715000	0.965711000
6	1.645957000	-0.919408000	0.448232000
6	1.634485000	-0.363798000	-0.965690000
1	1.791600000	1.900501000	-0.870384000
1	1.724475000	0.787422000	1.952760000
1	1.791482000	-1.900594000	0.870411000
1	1.724510000	-0.787507000	-1.952736000
27	-0.255486000	-0.000007000	-0.000034000
6	-1.040749000	2.036935000	-0.246984000
6	-1.736178000	1.415029000	0.539839000
1	-2.498586000	1.099214000	1.220853000
1	-0.588869000	2.772724000	-0.874470000
1	-2.498553000	-1.099194000	-1.221000000
6	-1.736222000	-1.414959000	-0.539876000
6	-1.040910000	-2.036810000	0.247100000
1	-0.589162000	-2.772551000	0.874738000

Frequency	Intensity	Frequency	Intensity	Frequency	Intensity
82.358	0.0109	674.6818	0.0077	1127.7764	0.6755
92.3572	1.0165	696.9388	25.0971	1199.729	0.0023
110.4252	0.3065	704.9022	0.0414	1288.0156	25.466
111.6673	0.1781	722.1226	7.7341	1382.7489	0.4078
150.762	2.9435	754.9777	74.2422	1478.3144	0.681
180.4053	0.8448	758.9026	76.8809	1935.2097	5.5863
217.37	1.3429	771.1524	8.5928	1935.793	12.1452
232.4892	5.2475	773.5675	24.3458	3233.1267	0.1235
246.8685	6.163	780.117	15.9989	3247.3842	6.5714
302.0341	3.3067	809.5893	13.8101	3257.6828	8.024
338.9088	1.1592	872.5212	9.586	3268.103	4.3516
359.7935	9.0675	885.7297	0.1807	3346.5288	152.4003
367.0637	11.8238	902.4235	2.471	3346.6947	107.1089
573.8965	0.0996	944.6381	1.3497	3433.2556	81.8512
671.9889	1.3908	1026.8073	9.7433	3434.1776	31.4377

Table S49. Cartesian coordinates for the optimized geometry of isomer 4i-triplet $Co^{+}(C_2H_2)_4$ followed by its predicted frequencies (cm^{-1}) and IR intensities (km/mol).

Z	x	y	z
27	-0.000004000	0.000012000	-0.338125000
6	0.983320000	-0.952241000	0.850871000
6	2.308742000	-0.783467000	0.543059000
1	3.086051000	-1.442710000	0.919990000
1	0.587333000	-1.613211000	1.611590000
6	2.607329000	0.354236000	-0.276766000
6	1.540628000	1.177532000	-0.515608000
1	1.618654000	2.184795000	-0.912660000
1	3.608117000	0.528738000	-0.663521000
1	-1.618614000	-2.184805000	-0.912668000
6	-1.540605000	-1.177543000	-0.515610000
6	-2.607316000	-0.354259000	-0.276771000
1	-3.608099000	-0.528771000	-0.663534000
6	-2.308749000	0.783452000	0.543052000
1	-3.086072000	1.442685000	0.919975000
6	-0.983333000	0.952248000	0.850875000
1	-0.587356000	1.613216000	1.611600000

Frequency	Intensity	Frequency	Intensity	Frequency	Intensity
30.9704	1.5794	754.0249	10.0624	1231.3124	33.4906
61.5316	2.5574	768.2082	15.6696	1258.7227	2.7321
134.2042	0.0079	799.5126	3.5524	1295.5364	2.8564
212.0786	1.6336	808.8942	0.8952	1455.6726	0.018
219.0281	0.0575	920.9769	13.2511	1466.373	3.9717
327.4617	1.2352	943.6738	8.6219	1502.6451	42.7468
337.1492	1.9662	952.5216	24.5288	1502.9989	14.363
353.0345	27.7675	957.6329	0.5364	3139.816	0.1068
414.6966	2.1204	1009.1472	2.2923	3140.1218	0.0155
431.7702	0.0288	1017.6487	0.1268	3155.2381	3.1135
469.336	2.2265	1071.9024	3.2378	3155.4202	0.0601
610.3048	8.7145	1073.7335	5.8208	3169.3416	5.3262
629.6496	10.2424	1097.3046	2.804	3169.3772	0.8144
668.8615	23.6161	1097.8883	0.3095	3197.5015	3.0594
691.5054	72.3842	1198.4497	13.6252	3198.4678	2.6489

Table S50. Cartesian coordinates for the optimized geometry of isomer 4j-triplet $Co^{+}(C_2H_2)_4$ followed by its predicted frequencies (cm^{-1}) and IR intensities (km/mol).

Z	x	y	z
27	0.836967000	0.000002000	0.000005000
6	2.853499000	-0.520957000	-0.321704000
6	2.853520000	0.520918000	0.321645000
1	3.144422000	1.397538000	0.861504000
1	3.144365000	-1.397582000	-0.861575000
6	0.290224000	-2.037939000	0.157664000
6	-0.685564000	-1.456958000	-0.297827000
1	-1.661075000	-1.193474000	-0.656352000
1	0.965661000	-2.776248000	0.534975000
6	0.290229000	2.037947000	-0.157670000
6	-0.685520000	1.456986000	0.297935000
1	-1.661025000	1.193522000	0.656488000
1	0.965661000	2.776258000	-0.534988000
6	-4.064145000	-0.310620000	0.512556000
6	-4.064120000	0.310614000	-0.512617000
1	-4.112406000	0.864743000	-1.420681000
1	-4.112452000	-0.864758000	1.420613000

Frequency	Intensity	Frequency	Intensity	Frequency	Intensity
20.0041	0.0953	395.0187	7.6339	796.1882	4.152
49.2092	0.2229	399.6592	7.1893	800.1787	3.4288
56.3043	0.0069	642.7882	0.0058	827.0456	1.8326
70.9377	2.5629	670.0069	0.5663	1917.9462	7.5862
95.2475	1.0942	683.4096	0.1084	1922.0737	10.9106
109.3785	0.6244	688.6583	4.195	1922.4514	0.6127
118.5612	0.0019	694.8244	1.4319	2061.8868	9.1559
130.3566	0.4958	717.4203	28.3325	3310.2	66.2802
137.8095	1.5365	729.3405	13.8407	3315.0974	379.9891
149.7726	1.895	742.4163	0.6515	3335.172	127.6647
162.9004	3.9502	774.6991	27.0664	3395.92	118.379
261.0405	3.2235	776.5782	24.0805	3404.5122	86.2651
267.0898	2.4405	779.5104	80.1044	3406.9251	41.5498
271.3913	2.2104	780.2252	186.128	3415.7871	41.899
394.4388	2.2854	790.186	153.6311	3495.6618	1.2846

Table S51. Cartesian coordinates for the optimized geometry of isomer 4k-triplet $Co^{+}(C_2H_2)_4$ followed by its predicted frequencies (cm^{-1}) and IR intensities (km/mol).

Z	x	y	z
27	-0.375184000	-0.000004000	0.000000000
6	1.069814000	0.765322000	0.947492000
6	2.358070000	0.428250000	0.553868000
1	3.253872000	0.794510000	1.043600000
1	0.889484000	1.384411000	1.825397000
6	2.358066000	-0.428244000	-0.553903000
6	1.069811000	-0.765371000	-0.947474000
1	0.889463000	-1.384516000	-1.825335000
1	3.253865000	-0.794473000	-1.043664000
6	-1.870649000	1.373215000	-0.541918000
6	-0.891452000	2.059522000	-0.274310000
1	-0.230168000	2.884120000	-0.118094000
1	-2.842615000	1.072523000	-0.871607000
1	-0.230157000	-2.884092000	0.118288000
6	-0.891472000	-2.059496000	0.274387000
6	-1.870702000	-1.373183000	0.541855000
1	-2.842700000	-1.072475000	0.871435000

Frequency	Intensity	Frequency	Intensity	Frequency	Intensity
15.7888	0.4319	661.0248	58.9738	1113.1751	0.4932
70.8948	0.7761	686.6559	2.7401	1237.9941	4.7131
85.325	0.5247	692.3602	3.6697	1330.1627	0.4925
106.5455	6.3891	716.2454	12.6236	1450.6039	56.1419
136.3698	3.7593	749.6389	1.2695	1494.3589	0.5173
178.0207	0.0746	761.1071	115.7248	1906.6773	9.958
191.4305	8.8344	768.4568	19.3515	1911.9354	6.542
232.5388	6.7911	775.2984	0.2061	3118.0394	0.4742
278.3211	1.2209	794.9301	6.7797	3122.2189	1.4476
356.1807	4.817	805.891	12.7265	3171.1725	0.0324
376.9952	0.8446	809.2671	0.0593	3185.0753	0.2209
389.7729	5.9957	927.3596	3.5788	3341.1126	63.6526
427.895	27.3343	989.0492	0.2689	3342.5708	160.4774
469.0156	1.1598	1067.533	0.3222	3420.1396	78.714
590.3231	8.74	1091.3116	4.7657	3420.8698	29.4656

Table S52. Cartesian coordinates for the optimized geometry of isomer 4l-triplet $Co^+(C_2H_2)_4$ followed by its predicted frequencies (cm^{-1}) and IR intensities (km/mol).

Z	x	y	z
27	-0.246372000	0.039877000	0.002016000
6	-0.375931000	2.190429000	-0.213050000
6	-1.348386000	1.676739000	-0.745449000
1	-2.264326000	1.490910000	-1.266600000
1	0.344818000	2.870789000	0.185533000
6	1.127421000	0.051048000	2.041747000
6	1.869784000	0.607646000	1.273621000
1	2.593680000	1.098234000	0.664411000
1	0.568373000	-0.426406000	2.814041000
6	-1.812811000	-1.150669000	0.759996000
6	-1.081442000	-1.948766000	0.193165000
1	-0.638944000	-2.821563000	-0.235392000
1	-2.602596000	-0.681971000	1.309331000
6	1.556601000	-1.184727000	-1.278768000
6	1.047296000	-0.400806000	-2.038521000
1	0.683642000	0.246686000	-2.803664000
1	2.072205000	-1.898717000	-0.678526000

Frequency	Intensity	Frequency	Intensity	Frequency	Intensity
11.3562	0.3052	357.3769	0.7905	782.892	8.2941
46.0209	0.5026	376.4785	5.0215	798.5622	36.9017
68.1528	0.0363	650.7852	0.1331	803.7879	56.4188
90.8497	0.0824	652.6766	0.362	1928.1868	21.9816
92.8563	0.7926	672.2792	0.0582	1931.2548	3.4131
111.8369	0.0167	676.0957	1.4962	2028.0892	2.2947
126.1324	0.0286	678.4259	0.0844	2028.6726	2.3325
134.8159	0.7073	685.8459	0.0794	3339.107	108.9969
145.5464	0.2077	702.2588	17.4361	3339.6531	117.4307
149.1156	1.0774	707.7633	15.6339	3382.0782	116.2592
161.4793	0.4809	742.3853	27.691	3382.3459	154.3254
207.2146	0.9192	762.1705	79.4274	3423.2434	84.7507
208.6703	0.9296	763.7858	149.671	3424.5612	13.87
247.3509	9.0676	770.4924	1.5558	3477.9496	15.2924
248.4617	0.9502	779.6098	91.0447	3478.5473	14.3441

Table S53. Cartesian coordinates for the optimized geometry of isomer 4m-triplet $Co^{+}(C_2H_2)_4$ followed by its predicted frequencies (cm^{-1}) and IR intensities (km/mol).

Z	x	y	z
6	0.518870000	-1.695397000	1.466501000
6	1.494479000	-0.996389000	1.574513000
1	2.390187000	-0.453508000	1.772526000
1	-0.293592000	-2.385325000	1.466519000
27	-0.000120000	0.228525000	0.000074000
6	-0.517862000	-1.694248000	-1.468013000
6	-1.493847000	-0.995674000	-1.575468000
1	-2.389857000	-0.453139000	-1.773067000
1	0.294949000	-2.383765000	-1.468627000
6	1.055034000	1.570788000	-1.233699000
6	1.844481000	0.665907000	-1.000365000
1	2.707590000	0.035486000	-0.987408000
1	0.576239000	2.455730000	-1.597663000
1	-0.577401000	2.453645000	1.600339000
6	-1.055807000	1.568938000	1.235292000
6	-1.844865000	0.663976000	1.000927000
1	-2.707774000	0.033292000	0.987248000

Frequency	Intensity	Frequency	Intensity	Frequency	Intensity
53.4526	0.0609	367.923	0.9452	780.982	39.1638
68.2368	0.0416	374.5265	2.9218	798.7401	68.1582
72.9192	0.435	640.8228	0.0876	802.7146	20.4512
99.4231	1.5483	643.6364	0.0004	1920.3795	27.162
103.3919	0.0292	671.5429	1.0814	1923.979	2.7132
114.4965	0.0002	673.4082	0.4306	2025.2712	1.4293
122.1249	0.036	676.6626	1.8797	2026.6923	3.5008
141.805	0.0467	686.5774	2.6309	3336.8503	87.3493
142.7249	1.3531	707.175	11.4261	3337.1277	131.3849
144.9767	1.6708	718.6742	29.3633	3382.9169	175.3922
157.4813	1.088	738.7751	17.3395	3383.3219	89.1315
216.0786	2.7657	755.2179	62.6169	3417.4276	87.8494
226.6687	0.8023	765.9836	139.3927	3418.8369	17.046
252.2463	0.2962	774.7706	3.0394	3477.922	23.3206
264.0197	8.9152	779.1623	90.5996	3478.6786	10.2904

Table S54. Cartesian coordinates for the optimized geometry of isomer 4n-triplet $Co^{+}(C_2H_2)_4$ followed by its predicted frequencies (cm^{-1}) and IR intensities (km/mol).

Z	x	y	z
27	-0.760031000	-0.000047000	-0.076649000
6	-0.485662000	1.949984000	-1.200266000
6	0.547486000	1.717631000	-0.609734000
1	1.519713000	1.638314000	-0.164291000
1	-1.326316000	2.294206000	-1.763160000
6	-0.485618000	-1.950006000	-1.200206000
6	0.547525000	-1.717636000	-0.609665000
1	1.519751000	-1.638316000	-0.164220000
1	-1.326246000	-2.294270000	-1.763115000
6	-2.190205000	0.615719000	1.258651000
6	-2.190212000	-0.615595000	1.258732000
1	-2.417873000	-1.633475000	1.501764000
1	-2.417852000	1.633635000	1.501542000
6	3.780359000	0.000021000	0.107080000
6	3.421157000	0.000063000	1.250847000
1	3.156371000	0.000099000	2.282427000
1	4.144321000	-0.000017000	-0.894050000

Frequency	Intensity	Frequency	Intensity	Frequency	Intensity
19.8313	0.1826	345.4003	8.8352	796.1371	87.6575
21.3335	0.4435	420.9855	0.0401	799.163	1.5959
48.7649	0.4912	646.4867	0.0004	812.1285	29.6647
55.3957	0.0065	661.4019	0.1913	1885.4363	18.4491
69.5137	0.4557	663.0686	6.6538	1974.6449	0.5378
74.2987	1.8203	673.6242	4.3103	1980.991	1.0615
101.6798	1.1472	689.884	2.5305	2061.0414	8.28
108.2209	0.1838	699.2568	0.1241	3315.0897	14.4074
117.3422	0.8161	707.0065	26.361	3319.2156	109.9273
125.216	1.8757	736.2625	45.1504	3319.9499	504.5094
149.2291	0.1435	752.8196	93.0376	3393.1991	116.2485
175.5011	0.0001	766.5695	6.6676	3394.8466	53.5443
237.7787	0.6961	767.4595	74.1768	3424.8967	33.6188
268.9332	1.0204	782.1074	97.0648	3428.0363	21.6643
289.8334	5.1873	786.4577	104.3614	3493.1363	1.3452

Table S55. Cartesian coordinates for the optimized geometry of isomer 4a-quintet $Co^{+}(C_2H_2)_4$ followed by its predicted frequencies (cm^{-1}) and IR intensities (km/mol).

Z	x	y	z
6	0.635193000	1.448545000	0.701378000
6	0.634641000	1.449588000	-0.699299000
6	1.215524000	0.380575000	1.400790000
1	0.162618000	2.258689000	-1.239510000
1	1.196718000	0.368845000	2.482322000
6	1.214464000	0.382683000	-1.400759000
6	1.832047000	-0.668819000	0.699015000
1	1.194828000	0.372575000	-2.482292000
1	2.290119000	-1.485964000	1.240442000
6	1.831534000	-0.667749000	-0.701036000
1	2.289199000	-1.484074000	-1.244044000
1	0.163600000	2.256845000	1.243165000
27	-0.488092000	-0.641714000	-0.000005000
1	-3.084913000	-1.392897000	-0.000153000
6	-2.420979000	-0.523505000	-0.000073000
1	-3.712710000	1.338869000	0.000049000
6	-2.829252000	0.714246000	0.000010000

Frequency	Intensity	Frequency	Intensity	Frequency	Intensity
2.5499	0.0755	769.9003	97.9924	1326.3881	0.6153
86.2979	0.0001	842.8868	0.6383	1390.2692	0.0002
89.0944	0.7733	925.4733	1.9765	1503.6746	27.0508
135.5177	0.376	927.1745	0.0441	1506.4389	26.5915
154.4031	0.3575	998.0737	2.1966	1538.6842	34.4507
180.2502	6.7384	1022.5502	0.5209	1594.5704	0.0118
198.6904	4.8369	1024.0211	0.0351	1599.8058	1.5431
392.6911	1.003	1028.8013	0.1148	3027.0913	1.4815
395.2992	0.3159	1043.8275	0.2617	3195.6348	0.0055
518.2608	95.6579	1047.63	0.9206	3202.265	0.4105
535.2422	35.1523	1048.6912	31.594	3204.4166	1.0483
613.6567	0.0276	1053.2795	9.17	3207.7758	2.7363
614.0354	0.1764	1190.8979	0.1613	3211.6877	9.5292
688.811	0.0355	1200.7899	0.0001	3212.9547	10.3551
755.9281	39.8581	1202.0369	0.2016	3220.0621	2.3416

Table S56. Cartesian coordinates for the optimized geometry of isomer 4b-quintet $Co^+(C_2H_2)_4$ followed by its predicted frequencies (cm^{-1}) and IR intensities (km/mol).

Z	x	y	z
6	-0.039419000	-1.578605000	0.397040000
6	-0.394858000	-0.676462000	1.505644000
6	-0.395006000	0.675947000	1.505709000
6	2.235587000	0.667569000	-0.255792000
6	1.087388000	1.570056000	-0.354390000
6	-0.039659000	1.578346000	0.397257000
1	1.212251000	2.394166000	-1.051777000
1	-0.673067000	-2.457624000	0.307875000
1	-0.807606000	-1.171459000	2.379088000
1	-0.807850000	1.170773000	2.379205000
1	3.196750000	1.173131000	-0.272067000
1	-0.673349000	2.457358000	0.308342000
27	-1.500709000	0.000078000	-0.675350000
1	1.212364000	-2.393693000	-1.052504000
6	1.087481000	-1.569872000	-0.354778000
6	2.235625000	-0.667303000	-0.255929000
1	3.196816000	-1.172811000	-0.272274000

Frequency	Intensity	Frequency	Intensity	Frequency	Intensity
63.3759	0.4757	830.3508	17.5914	1432.8364	12.8553
114.4774	0.0013	904.3464	12.7486	1445.3096	4.511
155.2041	14.7328	931.44	6.8229	1473.774	4.5409
164.7145	1.9201	949.3207	2.4881	1606.3223	4.1147
188.9822	1.6494	952.2382	4.6299	1624.7425	33.6328
257.2455	0.1982	1000.1792	7.2151	1642.6239	24.8115
300.4202	0.7127	1014.1905	0.3124	1700.2648	35.8576
348.0691	10.6745	1022.3285	5.3268	3127.9468	5.3111
377.5971	3.6957	1025.4679	0.0001	3129.106	3.0773
606.4344	3.2205	1045.7429	0.1791	3141.2241	0.0593
661.0111	13.6485	1240.4558	3.2817	3147.551	0.2067
684.0247	3.739	1257.5381	1.2776	3150.0021	0.5517
735.4442	65.603	1269.3759	1.6266	3153.7077	4.5791
789.3295	17.822	1273.8785	0.6635	3160.1031	2.3043
826.4361	26.1042	1400.0838	0.094	3167.2624	3.0702

Table S57. Cartesian coordinates for the optimized geometry of isomer 4d-quintet $Co^{+}(C_2H_2)_4$ followed by its predicted frequencies (cm^{-1}) and IR intensities (km/mol).

Z	x	y	z
6	-1.233300000	0.171011000	1.038238000
6	-0.637252000	1.352393000	0.192523000
6	-1.747324000	0.991564000	-0.790116000
6	-2.256141000	0.003753000	0.037918000
1	-1.296911000	0.057603000	2.114517000
1	-0.801802000	2.318219000	0.677064000
1	-2.045384000	1.394596000	-1.747683000
1	-3.154133000	-0.598934000	-0.002200000
27	-0.123850000	-1.187431000	0.009473000
6	0.815839000	1.249175000	-0.303787000
1	1.049810000	2.111134000	-0.933695000
6	1.910046000	0.943527000	0.712702000
6	1.297137000	-0.099611000	-0.963941000
1	1.387159000	-0.327143000	-2.022030000
1	2.267253000	1.471110000	1.586132000
6	2.315920000	-0.194417000	0.055501000
1	3.148401000	-0.870337000	0.197898000

Frequency	Intensity	Frequency	Intensity	Frequency	Intensity
58.8549	0.9151	855.7603	6.818	1212.7275	0.9345
123.2801	0.0362	885.7725	1.277	1271.2707	0.6074
159.1911	0.8605	891.7373	21.8829	1324.9469	9.3454
201.6571	3.4257	920.175	4.7472	1336.112	14.9272
267.5879	1.3271	929.3477	1.0728	1337.044	9.5541
296.0433	17.9027	945.3533	7.844	1419.9055	33.0717
310.2101	21.5139	958.9726	2.5761	1446.4518	25.6434
419.7275	0.0755	1012.2657	3.4427	3058.3269	0.1829
514.0813	0.6172	1017.9912	0.7542	3065.6998	0.5856
637.1836	61.5001	1049.8646	3.4067	3149.2731	2.4934
668.639	38.6866	1122.568	24.2433	3172.4453	2.5708
723.1358	127.265	1138.6112	2.153	3202.0154	1.9171
759.8017	37.2836	1151.0877	4.9292	3204.03	1.6574
770.9598	10.454	1183.8014	4.0898	3226.1896	0.444
799.0882	35.4162	1205.8134	0.6264	3226.5599	1.1454

Table S58. Cartesian coordinates for the optimized geometry of isomer 4e-quintet $Co^{+}(C_2H_2)_4$ followed by its predicted frequencies (cm^{-1}) and IR intensities (km/mol).

Z	x	y	z
6	1.715585000	0.932578000	0.694286000
6	1.981216000	-0.554066000	0.703847000
6	1.981235000	-0.554068000	-0.703834000
6	1.715600000	0.932569000	-0.694292000
1	1.634394000	1.690724000	1.456761000
1	2.280249000	-1.266079000	1.457770000
1	2.280281000	-1.266095000	-1.457740000
1	1.634417000	1.690705000	-1.456778000
27	0.000007000	-0.399337000	-0.000004000
1	-2.280227000	-1.266088000	-1.457781000
6	-1.981204000	-0.554073000	-0.703855000
6	-1.715640000	0.932572000	-0.694272000
1	-1.634470000	1.690728000	-1.456739000
6	-1.715630000	0.932545000	0.694306000
1	-1.634460000	1.690674000	1.456800000
6	-1.981191000	-0.554109000	0.703830000
1	-2.280211000	-1.266150000	1.457732000

Frequency	Intensity	Frequency	Intensity	Frequency	Intensity
87.0141	0	741.92	19.7487	1201.6299	0.2985
110.2195	0.5322	772.2788	0	1274.3123	2.2654
124.4884	0.3591	785.9092	32.664	1285.5435	0
125.2669	0.296	822.9218	0.0007	1288.5585	46.4386
256.7508	5.3509	830.8734	0	1373.771	0.0003
285.0825	1.2045	848.325	0	1431.3924	0.1583
287.399	0	856.6831	2.4319	1438.2011	2.6804
303.797	1.8139	918.1403	0.2722	3226.4167	2.4373
321.4972	11.1025	937.0075	0	3227.0043	0
381.0055	16.1118	965.477	1.0736	3243.1541	0.0001
490.5308	0	1009.0703	2.1296	3243.4858	24.6106
575.4514	13.378	1009.4058	21.7256	3245.8396	16.7481
688.6225	23.8311	1093.1574	0.255	3246.2435	20.5699
714.0257	0.0028	1145.985	0.0565	3262.9114	11.1323
726.2402	65.5044	1200.8326	0	3264.6469	3.1092

Table S59. Cartesian coordinates for the optimized geometry of isomer 4f-quintet $Co^+(C_2H_2)_4$ followed by its predicted frequencies (cm^{-1}) and IR intensities (km/mol).

Z	x	y	z
6	-1.668285000	-0.885877000	1.063385000
6	-2.086846000	0.042456000	-0.085868000
6	-1.214609000	-0.917986000	-0.938496000
6	-0.932240000	-1.706578000	0.198545000
1	-1.887460000	-0.895392000	2.120962000
1	-3.148085000	-0.023003000	-0.329421000
1	-1.181275000	-1.111718000	-2.003013000
1	-0.416939000	-2.648773000	0.337939000
27	0.669561000	0.135022000	-0.262843000
6	-1.617141000	1.477965000	0.051597000
1	-2.386663000	2.241575000	0.155692000
6	-0.316363000	1.786925000	0.064047000
1	0.047858000	2.801847000	0.175031000
6	2.557904000	-0.000328000	0.877034000
6	2.810023000	-0.392251000	-0.244478000
1	3.204191000	-0.758488000	-1.170269000
1	2.495576000	0.322394000	1.895249000

Frequency	Intensity	Frequency	Intensity	Frequency	Intensity
41.5787	1.0793	685.9296	7.4407	1209.4145	0.8215
63.0233	0.3409	708.5946	35.0697	1274.1318	23.9148
95.7135	1.2368	736.2492	34.6579	1306.616	1.3105
102.3121	0.4818	766.1258	70.6288	1343.0523	5.2631
158.836	10.1664	781.7757	14.7109	1406.1542	5.043
190.1124	2.3124	855.7357	20.3481	1568.0365	2.2504
250.1449	0.4738	898.4798	1.483	1968.047	2.3695
267.6649	2.4365	911.5999	14.5127	3086.2626	0.1121
294.6326	5.7951	935.6909	16.0304	3110.4951	0.0028
360.1411	6.1937	955.0649	5.1776	3173.2071	0.6238
383.2796	2.0887	986.5234	16.6043	3187.9809	1.3847
532.8203	11.5767	1000.7427	3.7281	3204.0512	4.2693
634.3854	26.1149	1081.4028	7.3667	3234.19	2.9425
644.606	4.3692	1141.0618	10.748	3335.5439	187.5849
682.8943	9.531	1168.4425	13.9224	3426.8408	64.442

Table S60. Cartesian coordinates for the optimized geometry of isomer 4g-quintet $Co^{+}(C_2H_2)_4$ followed by its predicted frequencies (cm^{-1}) and IR intensities (km/mol).

Z	x	y	z
6	-2.700039000	-0.468570000	-0.001043000
6	-2.438025000	0.920858000	0.015446000
6	-1.578457000	-1.286634000	0.013903000
1	-3.243442000	1.649091000	0.050386000
1	-1.709322000	-2.366894000	0.026167000
6	-1.099109000	1.295157000	-0.003353000
1	-0.851245000	2.352227000	0.009628000
1	-3.715982000	-0.851328000	-0.014558000
27	0.074494000	-0.267762000	-0.024602000
6	1.723116000	0.975851000	-0.524363000
6	2.086350000	-0.369700000	-0.864984000
1	2.303277000	-0.894877000	-1.782192000
1	1.640501000	1.897656000	-1.075811000
1	1.801080000	1.200994000	1.817971000
6	1.840886000	0.639124000	0.898363000
1	2.316289000	-1.619103000	1.112380000
6	2.073194000	-0.729117000	0.552742000

Frequency	Intensity	Frequency	Intensity	Frequency	Intensity
46.3733	0.2089	758.4909	13.3445	1264.795	4.1999
68.7968	1.4566	783.4147	1.8338	1271.8293	0.9769
128.0089	1.1511	813.2914	3.0832	1323.875	13.8186
221.2654	0.1289	849.7446	1.1473	1344.7456	26.1975
250.5911	4.5321	857.5711	2.3289	1369.5852	11.0377
277.4291	0.4725	912.4104	10.2269	1463.5256	66.4809
319.9947	0.0523	949.1964	1.2011	1494.5453	6.494
334.4419	2.834	956.38	6.9129	3130.4651	0.3396
367.1583	11.5085	971.7451	10.2401	3146.1033	0.7274
487.5229	0.7678	1003.2815	0.2202	3161.689	0.7288
494.951	0.261	1032.7776	5.7955	3170.2149	0.2896
513.8143	4.0383	1108.368	9.6158	3233.4279	3.3845
652.6835	60.1748	1122.7287	1.1565	3247.5501	20.4711
734.1121	5.489	1139.9838	0.5186	3254.1928	14.4698
742.1566	53.7281	1208.3686	0.0173	3268.9609	8.8152

Table S61. Cartesian coordinates for the optimized geometry of isomer 4h-quintet $Co^+(C_2H_2)_4$ followed by its predicted frequencies (cm^{-1}) and IR intensities (km/mol).

Z	x	y	z
6	2.371235000	0.044531000	-0.712356000
6	2.371092000	0.044474000	0.712482000
6	1.381106000	-1.008145000	0.738316000
6	1.381273000	-1.008075000	-0.738485000
1	2.918835000	0.580497000	-1.472396000
1	2.918536000	0.580373000	1.472679000
1	1.123365000	-1.751323000	1.478870000
1	1.123520000	-1.751085000	-1.479201000
27	-0.454847000	0.062134000	0.000036000
6	-0.152628000	2.188118000	-0.000033000
6	-1.357269000	1.990817000	0.000034000
1	-2.423841000	2.070044000	0.000089000
1	0.824769000	2.619217000	-0.000085000
1	-2.295250000	-1.350810000	-1.658647000
6	-2.131955000	-1.236035000	-0.607814000
6	-2.131864000	-1.236218000	0.607721000
1	-2.295012000	-1.351329000	1.658541000

Frequency	Intensity	Frequency	Intensity	Frequency	Intensity
49.5707	0	649.9157	0.018	1208.5761	0.1577
77.6528	0.3743	665.3467	3.4489	1238.7029	1.4657
91.5177	0.9786	670.1296	0.987	1279.3662	13.9827
99.7601	0.0009	686.6276	19.5462	1327.3561	10.6135
107.3474	0.3317	697.8946	9.4594	1343.2982	3.359
115.7923	0.3319	719.5429	14.2275	1937.8253	12.5286
128.0216	2.7144	751.7426	82.9921	1961.2328	3.5821
145.9571	0.4806	754.4609	24.0269	3210.3638	0.86
205.3653	0.5499	761.3573	47.6797	3222.809	5.1391
250.8911	2.9787	779.1401	27.0031	3232.7335	4.8009
275.3346	5.0838	787.9465	3.8584	3251.2869	3.1516
294.283	4.9042	892.7608	10.3036	3341.1723	160.1015
352.7065	2.6069	928.0789	1.6961	3344.634	121.2686
487.0222	1.8882	945.2326	5.6739	3428.3404	56.2158
645.3176	83.3375	1006.5451	3.2684	3429.8015	42.5835

Table S62. Cartesian coordinates for the optimized geometry of isomer 4i-quintet $Co^{+}(C_2H_2)_4$ followed by its predicted frequencies (cm^{-1}) and IR intensities (km/mol).

Z	x	y	z
27	-0.000001000	-0.098964000	0.000032000
6	1.542656000	-1.226860000	0.493074000
6	2.688298000	-0.472309000	0.388690000
1	3.628903000	-0.757404000	0.854495000
1	1.554384000	-2.245802000	0.869327000
6	2.585290000	0.726538000	-0.384171000
6	1.310735000	1.140737000	-0.697540000
1	1.140977000	2.072183000	-1.229692000
1	3.478355000	1.258725000	-0.701119000
1	-1.554321000	-2.245833000	-0.869300000
6	-1.542621000	-1.226884000	-0.493066000
6	-2.688265000	-0.472323000	-0.388776000
1	-3.628831000	-0.757409000	-0.854665000
6	-2.585312000	0.726520000	0.384100000
1	-3.478401000	1.258713000	0.700972000
6	-1.310780000	1.140700000	0.697582000
1	-1.141058000	2.072128000	1.229778000

Frequency	Intensity	Frequency	Intensity	Frequency	Intensity
45.8584	0.6786	760.6341	24.9336	1238.5095	23.3713
47.9315	5.2203	761.1517	1.3864	1306.2503	1.8001
101.7285	2.9836	776.9616	35.4035	1317.4009	1.046
156.6944	2.4173	783.2618	1.6229	1467.8378	8.5702
185.764	1.9829	916.5039	17.2127	1479.0565	0.14
227.9084	0.5425	940.9114	18.9366	1488.9971	82.4343
318.2678	3.1321	954.8658	0.9232	1491.1886	26.1814
338.7599	3.0195	974.6406	0.7785	3133.966	0.0092
354.564	1.2354	1029.591	4.1944	3133.994	0.0208
438.4712	0.1556	1034.8747	0.3895	3147.1881	0.0841
463.2674	0.9762	1085.0639	0.9129	3147.3567	0.1035
516.1968	8.0377	1093.3131	14.2751	3160.7384	3.0166
560.5117	2.644	1107.7901	0.9823	3161.6293	1.1112
636.8274	8.6703	1116.8198	1.8777	3164.732	0.0858
639.1232	70.5442	1236.7	10.6654	3165.0601	0.4615

Table S63. Cartesian coordinates for the optimized geometry of isomer 4j-quintet $Co^{+}(C_2H_2)_4$ followed by its predicted frequencies (cm^{-1}) and IR intensities (km/mol).

Z	x	y	z
6	-0.380864000	1.720334000	0.552184000
6	0.620446000	1.748593000	1.270434000
1	1.341837000	2.062231000	1.999247000
1	-1.323546000	1.924084000	0.081540000
27	0.754115000	-0.000024000	0.094733000
6	-3.707268000	-0.000147000	0.095305000
6	-3.518409000	0.000420000	-1.088127000
1	-3.403878000	0.000922000	-2.146378000
1	-3.919887000	-0.000650000	1.138526000
6	0.620878000	-1.749657000	1.268946000
6	-0.380421000	-1.721040000	0.550687000
1	-1.323041000	-1.924695000	0.079880000
1	1.342324000	-2.063758000	1.997506000
1	3.465067000	0.000717000	-0.866383000
6	2.422405000	0.000705000	-1.126582000
6	1.421490000	0.000889000	-1.865213000
1	0.870467000	0.001207000	-2.787545000

Frequency	Intensity	Frequency	Intensity	Frequency	Intensity
15.8565	0.2477	392.5931	18.1141	778.6473	86.5051
31.1568	0.4117	420.307	9.5318	785.1205	110.9308
56.0849	2.4193	584.7104	79.2518	815.0744	3.9492
59.3165	0.0039	620.0732	6.0519	1770.7901	136.9799
81.2321	1.6752	644.6953	0.0157	1828.0225	83.3959
119.1892	1.2109	655.782	0.5564	1889.765	3.4592
120.9742	2.0109	663.1305	4.6098	2063.1368	6.9423
145.8903	5.4329	674.3005	29.3673	3272.0735	83.8023
163.0266	0.2423	682.2261	1.5555	3296.1573	0.469
169.7234	0.5617	696.7732	76.4373	3297.5664	311.1061
177.6095	0.0286	698.5792	8.6307	3337.7031	88.076
242.9228	50.2916	710.2086	120.9341	3369.7509	103.0559
244.0895	15.7115	716.9897	0.8607	3375.1104	51.2131
335.8552	2.3198	737.6997	67.7483	3399.0822	108.6375
387.7912	0.0268	738.3576	101.7898	3498.4744	1.3673

Table S64. Cartesian coordinates for the optimized geometry of isomer 4k-quintet $Co^{+}(C_2H_2)_4$ followed by its predicted frequencies (cm^{-1}) and IR intensities (km/mol).

Z	x	y	z
27	-0.261183000	-0.051437000	0.000002000
6	1.179518000	-0.062382000	1.388027000
6	2.390861000	0.016839000	0.714275000
1	3.342410000	0.083126000	1.236818000
1	1.210824000	-0.101517000	2.474434000
6	2.390863000	0.016815000	-0.714267000
6	1.179523000	-0.062437000	-1.388020000
1	1.210831000	-0.101611000	-2.474426000
1	3.342412000	0.083092000	-1.236811000
6	-1.443988000	1.888855000	-0.604278000
6	-1.443951000	1.888907000	0.604203000
1	-1.505324000	2.004931000	1.663893000
1	-1.505418000	2.004773000	-1.663977000
1	-0.538689000	-2.905748000	-0.000079000
6	-1.273624000	-2.130759000	-0.000013000
6	-2.212058000	-1.370455000	0.000067000
1	-3.107974000	-0.790547000	0.000140000

Frequency	Intensity	Frequency	Intensity	Frequency	Intensity
34.2873	0.0692	644.7517	0.0877	1136.2899	2.4101
57.9512	0.3615	650.4879	0.1381	1269.4607	3.6575
72.4075	0.0869	660.4328	63.638	1335.6122	1.3342
98.1846	0.1026	678.4898	1.4275	1479.0981	62.4623
100.3856	0.1444	689.5386	20.6503	1495.5837	0.5577
120.286	1.1566	733.6137	0.1603	2004.6884	1.0492
189.9943	2.18	743.095	2.6179	2010.9344	2.9686
194.4514	3.6005	772.9574	89.4915	3122.8815	0.0756
205.7384	1.0907	774.1382	74.376	3132.0738	1.0942
251.0842	5.2419	791.8681	49.9953	3143.8048	3.3224
251.0995	2.0836	802.5497	31.611	3150.4195	2.7212
262.0286	0.0087	983.09	1.6069	3364.503	160.4716
285.0946	0.3961	996.4321	0.4451	3365.8315	155.8776
451.5203	0.2062	1013.9705	2.3445	3458.2567	29.0938
577.0332	0.7813	1113.9425	16.0799	3461.2931	26.1755

Table S65. Cartesian coordinates for the optimized geometry of isomer 4n-quintet $Co^{+}(C_2H_2)_4$ followed by its predicted frequencies (cm^{-1}) and IR intensities (km/mol).

Z	x	y	z
27	-0.754081000	0.000093000	-0.094744000
6	0.380022000	1.721797000	-0.548998000
6	-0.621257000	1.750827000	-1.267281000
1	-1.342713000	2.065469000	-1.995601000
1	1.322558000	1.925348000	-0.077980000
6	-2.422443000	-0.002184000	1.126526000
6	-1.421564000	-0.002847000	1.865193000
1	-0.870530000	-0.003915000	2.787516000
1	-3.465079000	-0.002188000	0.866231000
6	-0.620009000	-1.747394000	-1.272084000
6	0.381252000	-1.719627000	-0.553749000
1	1.323950000	-1.923595000	-0.083232000
1	-1.341298000	-2.060475000	-2.001238000
6	3.518377000	-0.001383000	1.088126000
6	3.707226000	0.000442000	-0.095307000
1	3.919841000	0.002059000	-1.138528000
1	3.403851000	-0.003001000	2.146376000

Frequency	Intensity	Frequency	Intensity	Frequency	Intensity
15.8611	0.2476	392.6062	18.1129	778.6485	86.5062
31.1556	0.4118	420.283	9.5337	785.1207	110.9343
56.0854	2.4189	584.7118	79.2408	815.0725	3.9481
59.3292	0.0039	620.0542	6.0603	1770.8187	136.9762
81.2397	1.6752	644.6992	0.0157	1828.0009	83.4194
119.193	1.211	655.783	0.5564	1889.7589	3.4534
120.9712	2.0115	663.1359	4.6131	2063.1346	6.9423
145.8922	5.4326	674.298	29.3423	3272.0946	83.8184
163.0305	0.2421	682.2276	1.5537	3296.1532	0.471
169.7235	0.5614	696.7897	76.4319	3297.5617	311.0606
177.602	0.0287	698.584	8.6222	3337.7285	88.0975
242.9076	50.2917	710.1909	120.9559	3369.7419	103.064
244.0955	15.7223	716.9759	0.8601	3375.1019	51.1965
335.8415	2.3141	737.6856	67.8648	3399.0797	108.6371
387.804	0.0271	738.3484	101.6852	3498.4716	1.3673

Table S66. Cartesian coordinates for the optimized geometry of isomer 4o-quintet $Co^{+}(C_2H_2)_4$ followed by its predicted frequencies (cm^{-1}) and IR intensities (km/mol).

Z	x	y	z
6	0.712111000	1.772869000	0.595639000
6	-0.471868000	2.239915000	0.117411000
6	-1.536308000	1.577932000	-0.581388000
6	-1.198867000	-1.847231000	0.581212000
6	-2.055893000	-0.784502000	0.421329000
6	-2.169672000	0.368580000	-0.420752000
1	-3.013892000	-0.970670000	0.907123000
1	1.316479000	2.497544000	1.143894000
1	-0.657780000	3.311132000	0.210708000
1	-2.090322000	2.255406000	-1.224357000
1	-1.609868000	-2.619860000	1.224207000
1	-3.145903000	0.364337000	-0.905909000
27	1.542104000	0.151755000	0.000199000
1	1.778232000	-2.192610000	-1.144555000
6	1.044250000	-1.599870000	-0.595999000
6	-0.026070000	-2.288851000	-0.118177000
1	0.000163000	-3.375709000	-0.212143000

Frequency	Intensity	Frequency	Intensity	Frequency	Intensity
38.126	2.128	768.6967	14.7993	1369.0175	8.2524
92.2731	0.0607	775.5758	0.1175	1452.7864	144.4799
138.7999	0.2154	855.9198	19.4174	1495.4798	170.1969
154.6253	1.9006	890.7138	7.1011	1504.7218	0.79
221.2947	0.4609	925.2015	11.4514	1567.752	13.1134
244.7354	5.8307	978.366	69.4974	1571.2791	7.8484
338.3586	2.152	981.2579	0.5079	1585.9511	18.7753
409.2749	11.93	1014.0747	1.6992	3075.8796	0.133
461.707	5.0846	1018.8414	14.9507	3076.5565	0.0256
466.6906	7.7226	1051.3007	1.1247	3090.6777	0.3166
536.6581	2.4071	1171.1039	8.9785	3094.6933	0.6426
568.7267	0.4521	1200.2366	24.0895	3095.639	0.1024
655.6015	22.9697	1323.4472	3.062	3102.6989	4.1361
663.9983	96.4737	1331.8389	85.2841	3149.7598	0.0875
739.2188	2.274	1358.5823	2.403	3150.3886	0.0026

Table S67. Cartesian coordinates for the optimized geometry of isomer 4p-quintet $Co^{+}(C_2H_2)_4$ followed by its predicted frequencies (cm^{-1}) and IR intensities (km/mol).

Z	x	y	z
6	-2.610041000	-0.705380000	0.000010000
6	-1.634391000	-1.718708000	0.000013000
6	-0.257322000	-1.623051000	0.000006000
6	-2.610067000	0.705309000	-0.000003000
6	-1.634455000	1.718674000	-0.000014000
6	-0.257380000	1.623071000	-0.000012000
1	-2.064759000	2.717934000	-0.000024000
1	-3.620226000	-1.102692000	0.000018000
1	-2.064658000	-2.717984000	0.000022000
1	0.245314000	-2.592335000	0.000005000
1	-3.620266000	1.102584000	-0.000003000
1	0.245211000	2.592378000	-0.000015000
27	0.833992000	0.000032000	-0.000003000
6	3.007526000	-0.000094000	-0.605181000
6	3.007522000	0.000064000	0.605192000
1	3.146614000	0.000016000	1.666050000
1	3.146626000	-0.000067000	-1.666038000

Frequency	Intensity	Frequency	Intensity	Frequency	Intensity
60.6225	0	638.1614	0	1336.0709	27.646
61.7301	1.2056	692.7603	20.9635	1395.3107	1.6864
78.4729	1.1982	706.9785	0	1487.3992	88.8713
113.2113	0.103	767.5686	76.3422	1508.195	13.3686
148.9802	0	805.4022	23.5627	1550.104	23.1917
170.3798	1.6023	839.2691	0.3888	1558.2923	11.1986
242.848	0.0035	846.3976	5.2098	1994.9788	0.7614
264.7478	5.2995	853.3162	0.018	3079.1088	0.2159
303.1359	2.0138	995.7477	0	3079.5653	2.7143
315.2659	1.6949	1029.5766	1.7643	3119.1294	3.3762
463.7057	1.1548	1051.6315	0	3120.2411	0.3681
503.6833	3.4071	1056.7069	1.9221	3148.6202	0.1912
505.1163	0	1174.7549	7.7879	3166.0306	0.9874
606.7008	78.1338	1243.4488	7.1611	3341.1598	193.7416
630.2374	4.6997	1324.7034	2.1075	3436.0352	50.5531

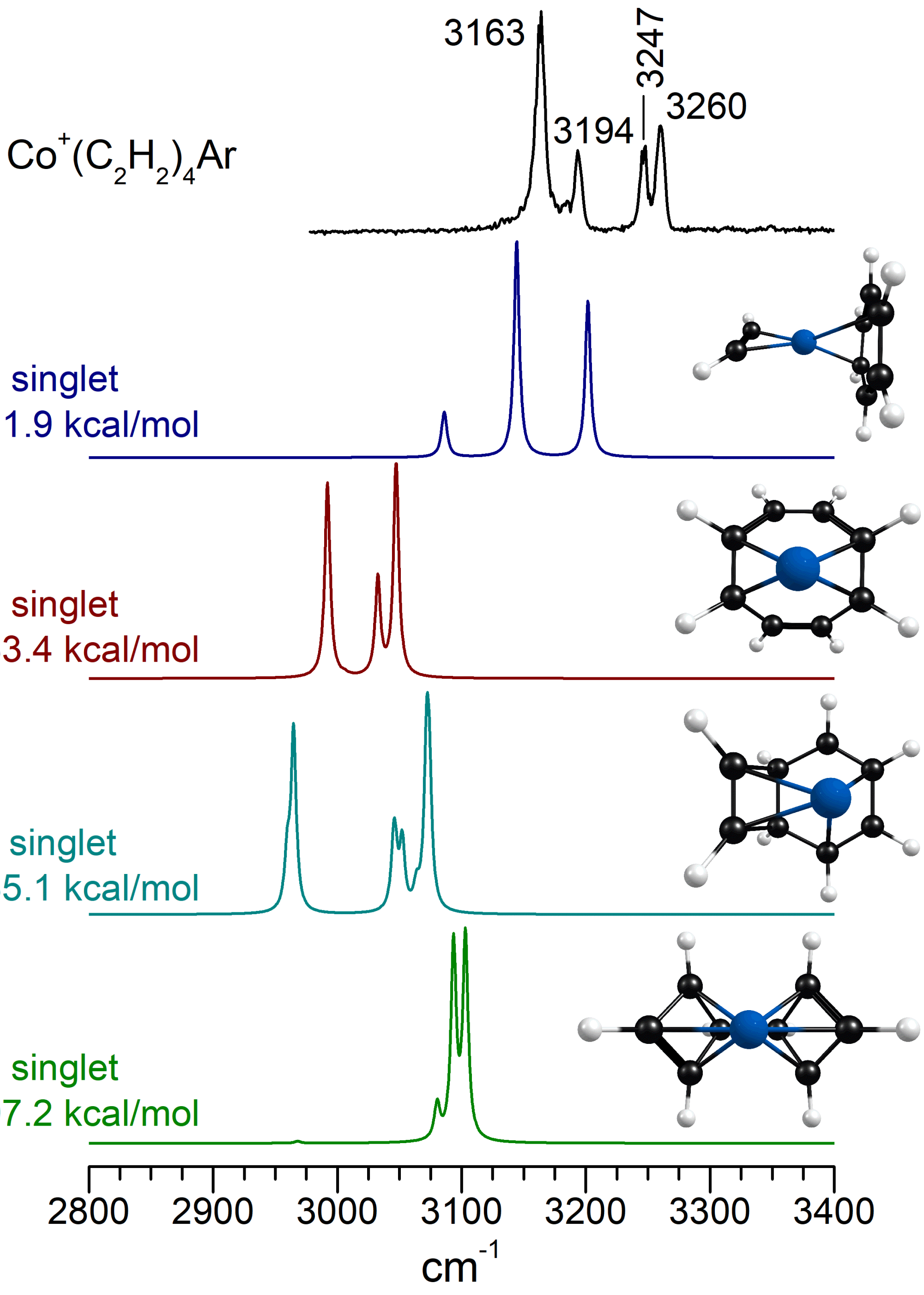

Figure S41. The experimental spectrum for $Co^{+}(C_2H_2)_4Ar$ with simulated spectra for $Co^{+}(C_2H_2)_4$ as a singlet and predicted isomers 4a-4d of singlet-$Co^{+}(C_2H_2)_4$.

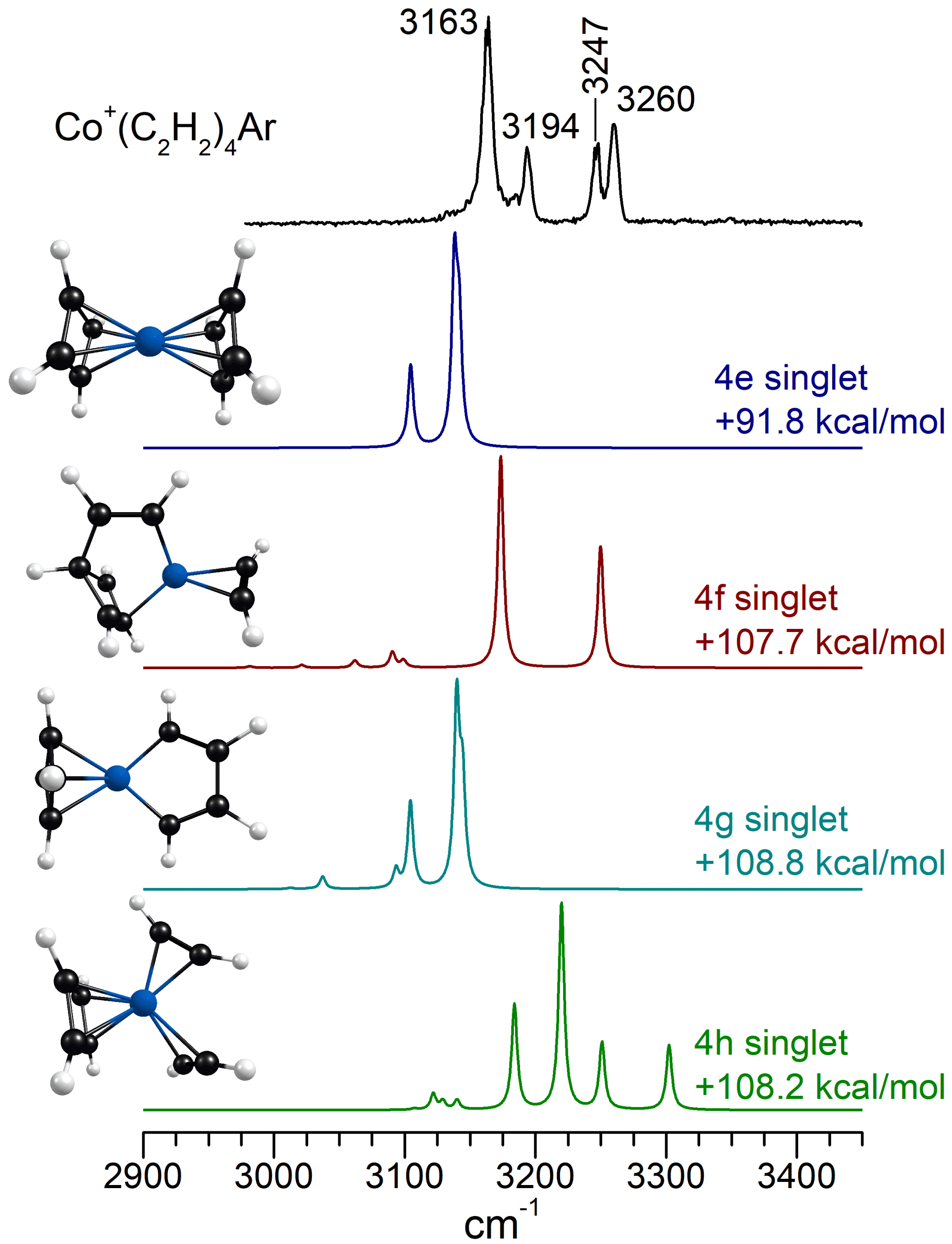

Figure S42. The experimental spectrum for $Co^+(C_2H_2)_4Ar$ with simulated spectra for $Co^+(C_2H_2)_4$ as a singlet and predicted isomers 4e-4h of singlet-$Co^+(C_2H_2)_4$.

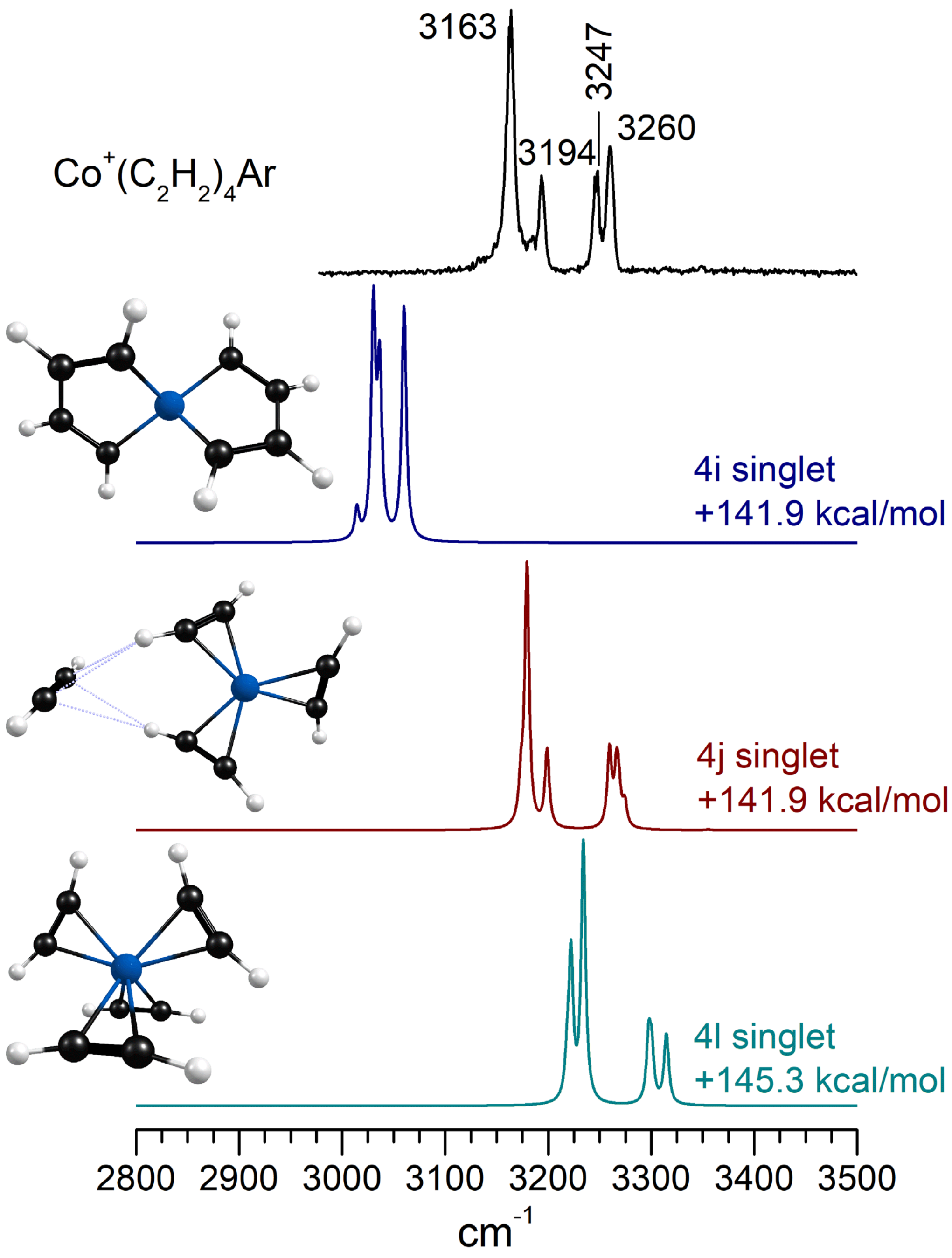

Figure S43. The experimental spectrum for $Co^{+}(C_2H_2)_4Ar$ with simulated spectra for $Co^{+}(C_2H_2)_4$ as a singlet and predicted isomers 4i-4l of singlet-$Co^{+}(C_2H_2)_4$.

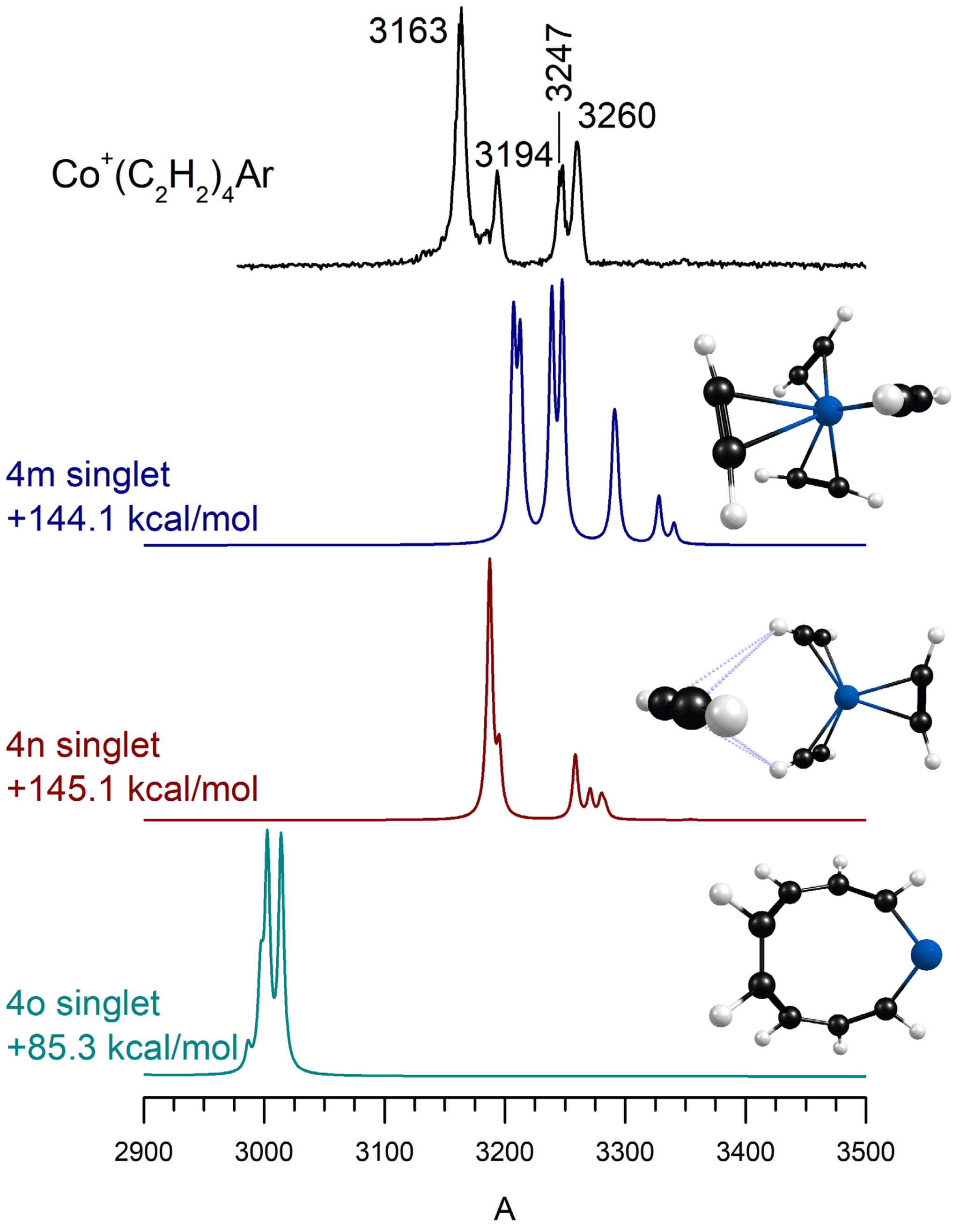

Figure S44. The experimental spectrum for $Co^+(C_2H_2)_4Ar$ with simulated spectra for $Co^+(C_2H_2)_4$ as a singlet and predicted isomers 4m-4o of singlet-$Co^+(C_2H_2)_4$.

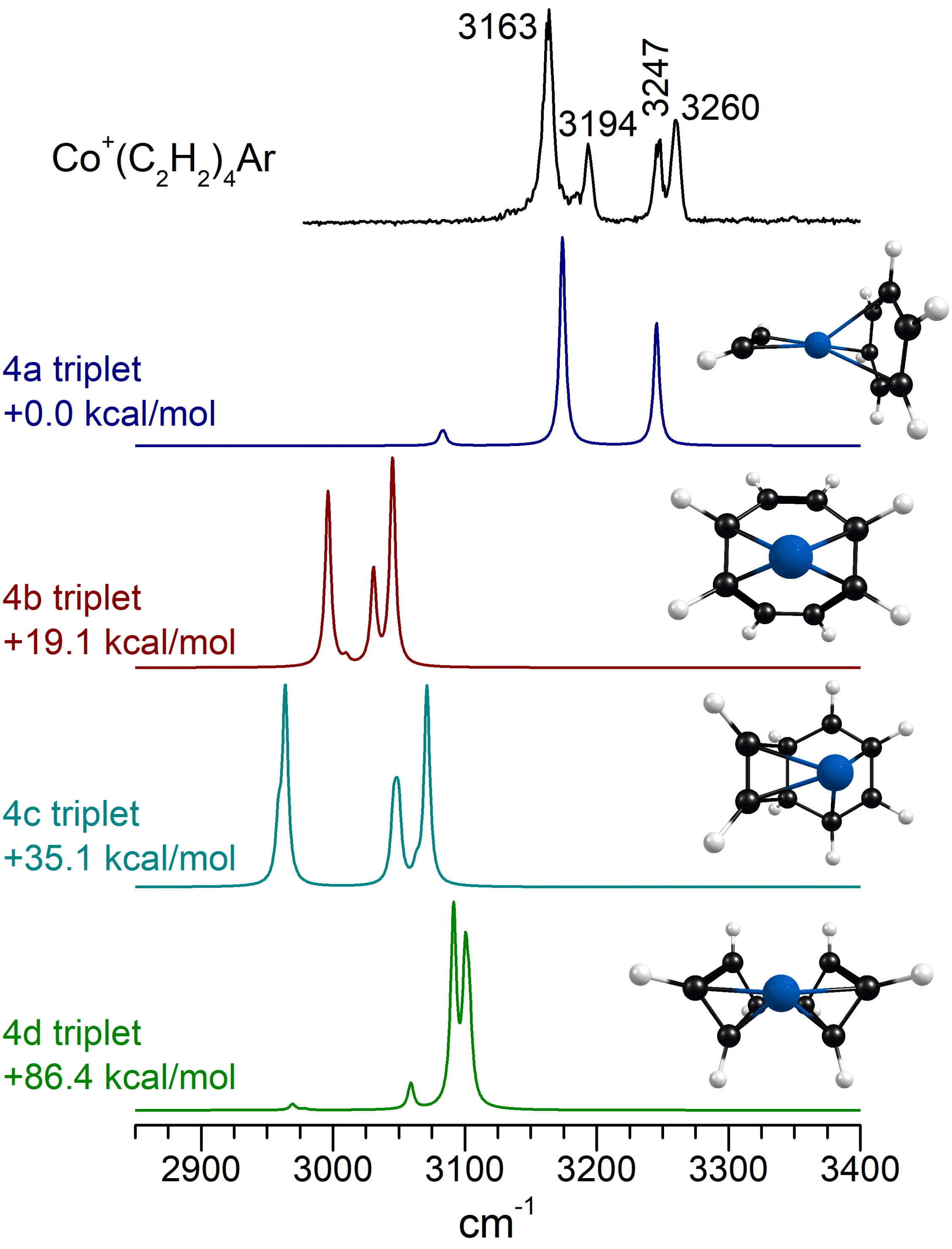

Figure S45. The experimental spectrum for $Co^{+}(C_2H_2)_4Ar$ with simulated spectra for $Co^{+}(C_2H_2)_4$ as a triplet and predicted isomers 4a-4d of triplet-$Co^{+}(C_2H_2)_4$.

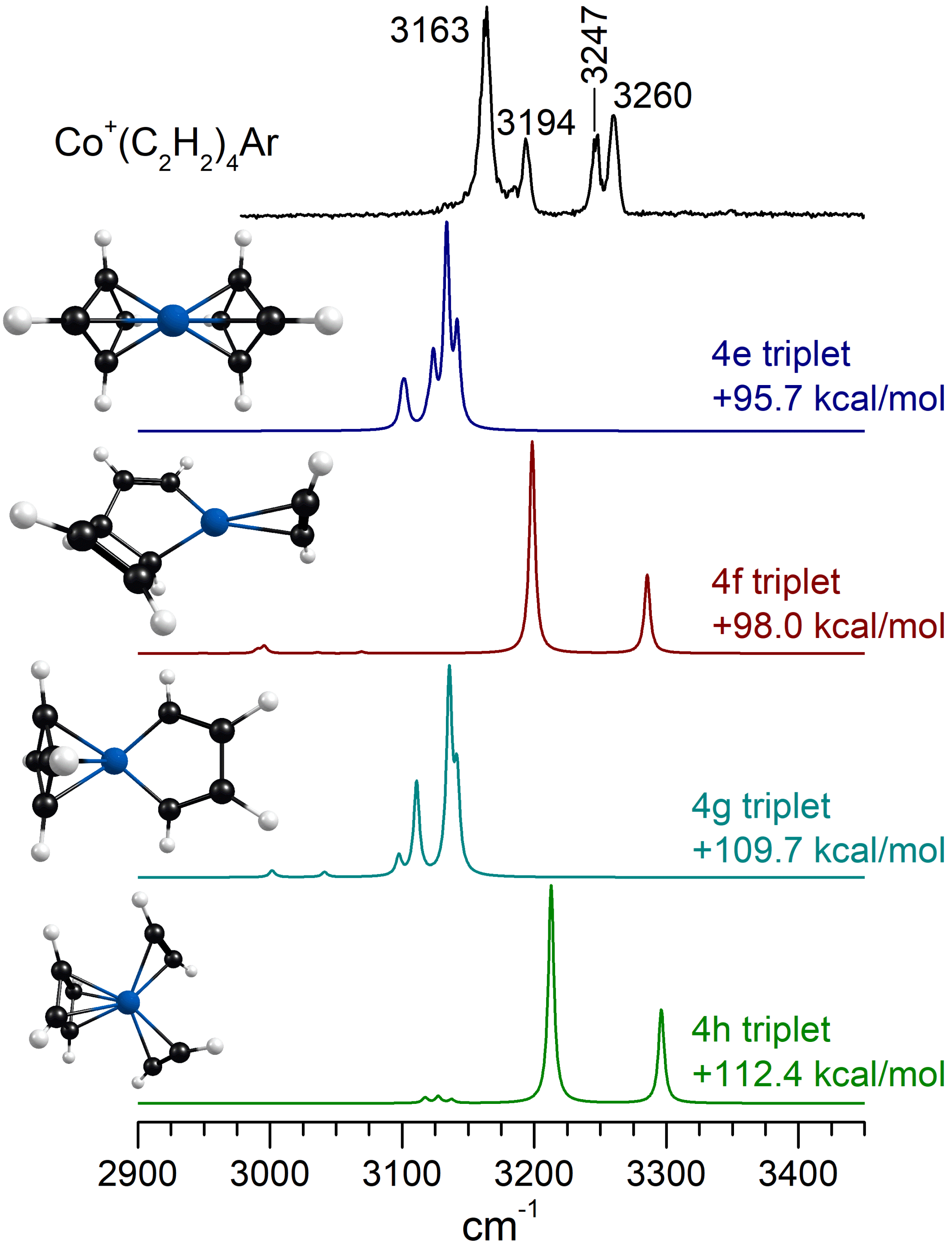

Figure S46. The experimental spectrum for $Co^+(C_2H_2)_4Ar$ with simulated spectra for $Co^+(C_2H_2)_4$ as a triplet and predicted isomers 4e-4h of triplet-$Co^+(C_2H_2)_4$.

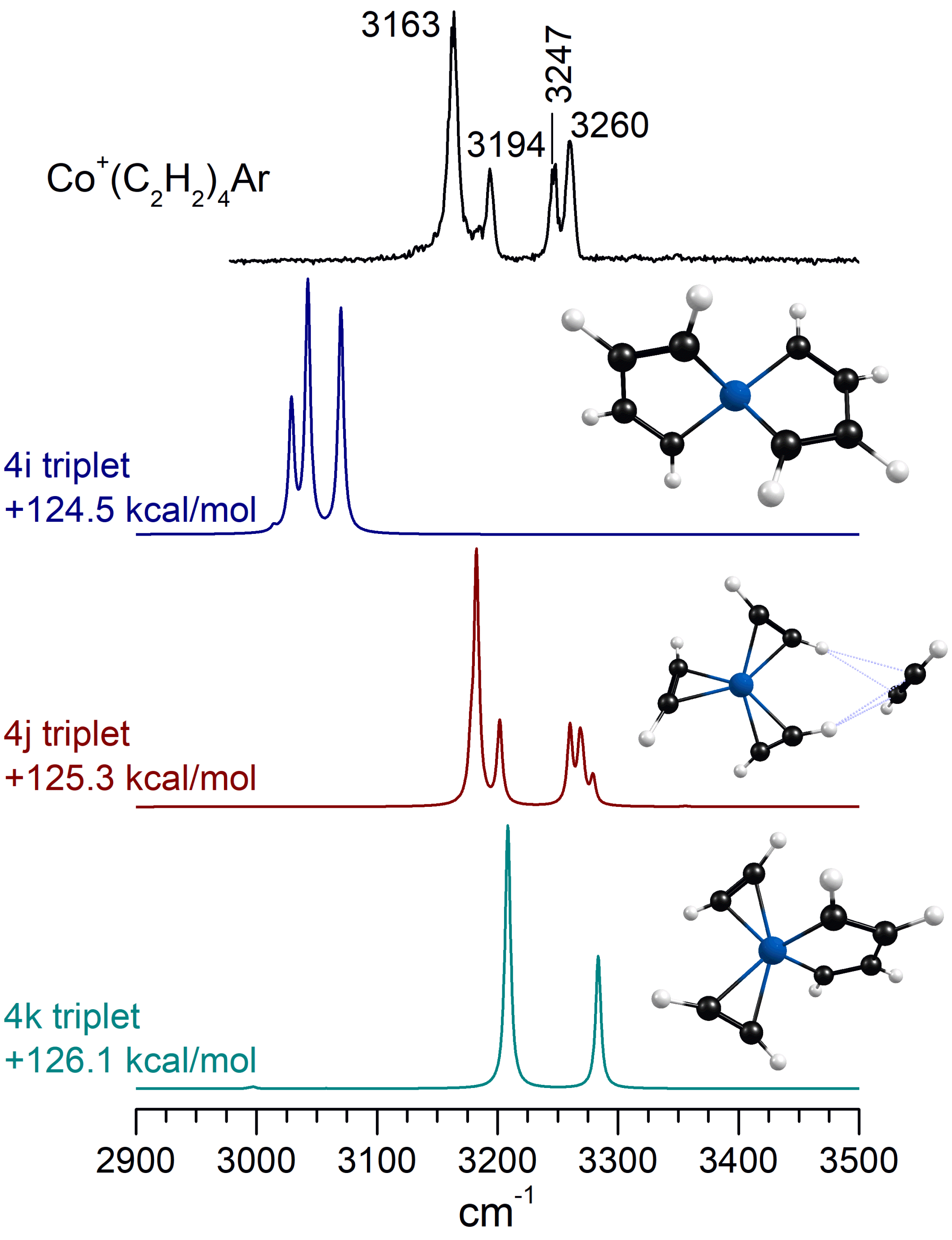

Figure S47. The experimental spectrum for $Co^+(C_2H_2)_4Ar$ with simulated spectra for $Co^+(C_2H_2)_4$ as a triplet and predicted isomers 4i-4k of triplet-$Co^+(C_2H_2)_4$.

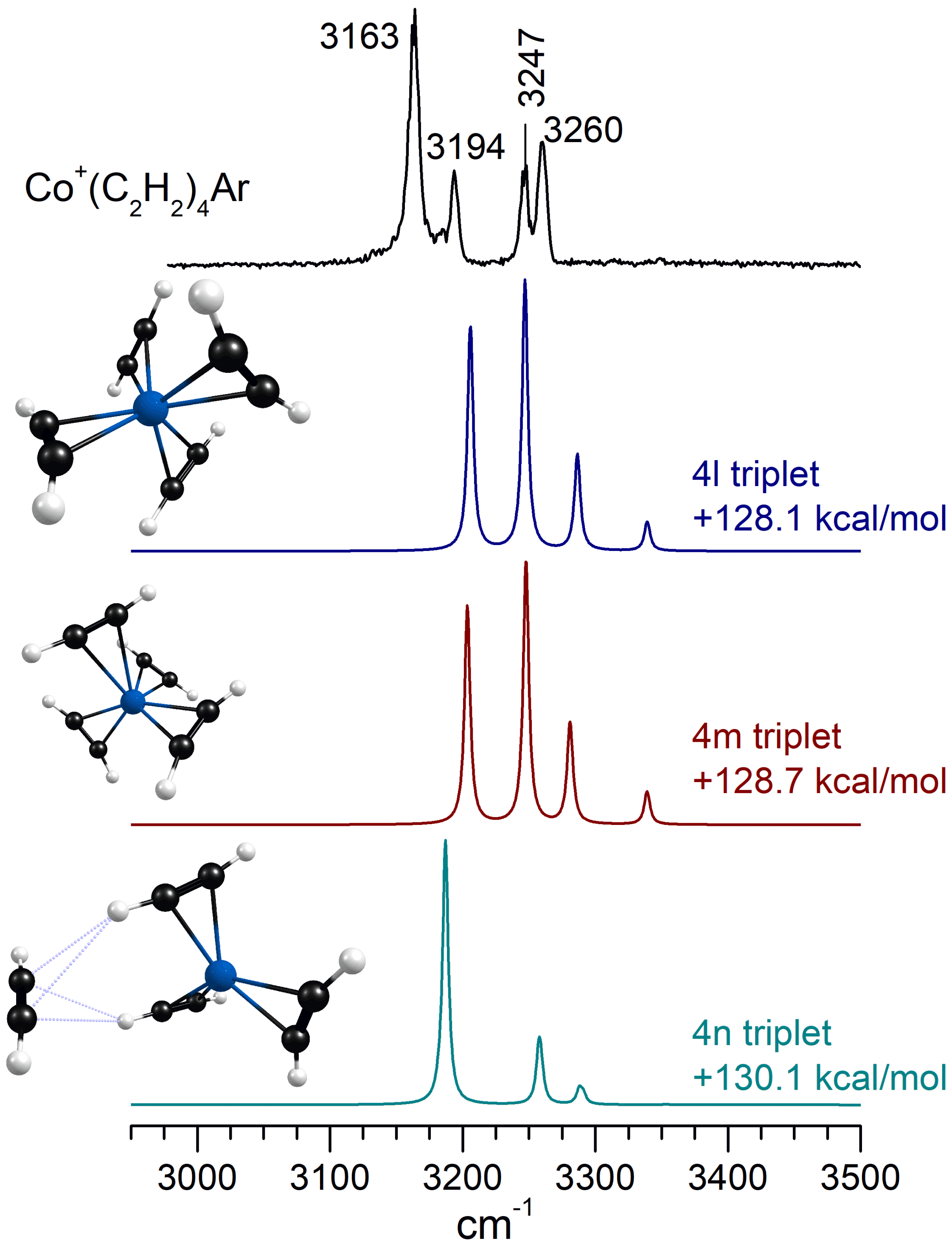

Figure S48. The experimental spectrum for $Co^+(C_2H_2)_4Ar$ with simulated spectra for $Co^+(C_2H_2)_4$ as a triplet and predicted isomers 4l-4n of triplet-$Co^+(C_2H_2)_4$.

Table S68. $Co^+(C_2H_2)_5$ calculated at the B3LYP/Def2TZVP level of theory using Gaussian16.

Isomer	2s + 1	E (hartree)	Relative E (kcal/mol)
5a	1	-1769.565345	+26.1
5a	3	-1769.589324	+11.0
5a	5	-1769.531624	+47.3
5b	1	-1769.562626	+27.8
5b	3	-1769.570044	+23.1
5b	5	-1769.539116	+42.6
5c	1	-1769.606929	+0.0
5c	3	-1769.563182	+27.5
5c	5	-1769.535650	+44.7
5d	1	-1769.508233	+61.9
5d	3	-1769.557808	+30.8
5d	5	-1769.523052	+52.6
5e	1	-1769.527298	+50.0
5e	3	-1769.553920	+33.3
5e	5	-1769.485419	+76.2
5f	3	-1769.547721	+37.2
5g	3	-1769.545740	+38.4
5g	5	-1769.509604	+61.1
5h	1 `	-1769.448461	+99.4
5h	3	-1769.525461	+51.1
5h	5	-1769.437574	+106.3
5i	1	-1769.460965	+91.6
5i	3	-1769.472548	+84.3
5i	5	-1769.481008	+79.0
5j	1	-1769.416100	+119.7
5j	3	-1769.430686	+110.6
5k	1	-1769.396215	+132.2
5k	3	-1769.403737	+127.5
5k	5	-1769.382247	+141.0
5l	1	-1769.386139	+138.5
5l	3	-1769.400133	+129.8
5l	5	-1769.374782	+145.7
5m	1	-1769.423095	+115.4
5m	3	-1769.392383	+134.6
5m	5	-1769.412572	+122.0
5n	1 `	-1769.367446	+150.3
5n	3	-1769.385372	+139.0
5n	5	-1769.336294	+169.8
5o	1	-1769.319380	+180.4
5o	3	-1769.345606	+164.0
5o	5	-1769.275938	+207.7

Isomer	2s + 1	E (hartree)	Relative E (kcal/mol)
5p	3	-1769.342998	+165.6
5p	5	-1769.330631	+173.4
5q	1	-1769.315533	+182.9
5q	3	-1769.340700	+167.1
5r	1	-1769.311496	+185.4
5r	3	-1769.339546	+167.8
5s	1	-1769.337529	+169.1
5s	3	-1769.336702	+169.6
5s	5	-1769.421122	+116.6
5t	1	-1769.337423	+169.1
5t	3	-1769.335995	+170.0
5t	5	-1769.421340	+116.5
5u	1	-1769.445375	+101.4
5v	1	-1769.422896	+115.5
5v	5	-1769.399626	+130.1
5w	1	-1769.417279	+119.0
5w	5	-1769.397546	+131.4
5x	1	-1769.399174	+130.4
5x	5	-1769.348866	+161.9

Table S69. Cartesian coordinates for the optimized geometry of isomer 5a-singlet of $Co^{+}(C_2H_2)_5$.

Z	x	y	z
1	2.157006000	-1.728130000	1.758990000
6	1.681840000	-1.199049000	0.943025000
6	2.368236000	-0.350125000	0.169994000
6	1.691659000	0.327877000	-0.938793000
6	0.496254000	-0.147131000	-1.419198000
6	-0.185533000	-1.335739000	-0.750787000
6	0.185524000	-1.335708000	0.750831000
6	-0.496255000	-0.147063000	1.419190000
6	-1.691664000	0.327927000	0.938770000
6	-2.368241000	-0.350117000	-0.169989000
6	-1.681849000	-1.199077000	-0.942985000
1	3.421836000	-0.160783000	0.326362000
1	2.238688000	1.068353000	-1.515169000
1	0.130153000	0.178184000	-2.387157000
1	-2.157027000	-1.728200000	-1.758916000
1	-3.421840000	-0.160780000	-0.326368000
1	-2.238687000	1.068430000	1.515115000
1	-0.130162000	0.178278000	2.387143000
1	0.180560000	-2.252558000	-1.224525000
1	-0.180580000	-2.252502000	1.224608000
27	0.000009000	1.416257000	-0.000016000

Table S70. Predicted frequencies (cm^{-1}) and IR intensities (km/mol) for isomer 5a-singlet of $Co^+(C_2H_2)_5$.

Frequency (cm^{-1})	Intensity (km/mol)	Frequency (cm^{-1})	Intensity (km/mol)
89.4283	0.7875	1037.4926	7.9719
157.7188	1.9858	1079.7428	2.298
189.7128	1.6935	1174.0719	0.0399
227.9116	0.1159	1189.9768	0.8412
268.687	0.0567	1193.8186	0.2012
298.142	2.4319	1196.0812	1.387
330.7167	13.5448	1269.1121	0.0003
416.0442	0.7216	1274.9132	2.8526
452.4977	6.77	1319.9466	0.0008
517.3713	2.9349	1323.4338	7.6704
538.4712	0.0981	1383.416	0.9156
593.6089	5.11	1385.0353	5.8392
603.0212	1.9751	1423.1706	2.6861
663.3594	5.5643	1438.8359	10.1428
742.6801	36.2077	1531.9796	1.4766
748.5805	38.5906	1545.8473	5.9374
779.9009	15.5993	1661.0045	11.1549
815.7116	0.1888	1667.5914	3.185
841.2679	0.0408	3030.046	1.7803
915.25	2.0857	3036.6028	1.8906
926.3767	2.5253	3144.2371	3.7043
949.6676	4.5547	3144.6736	0.6887
956.66	44.2434	3164.3646	0.3826
972.5336	4.9569	3164.7816	0.0708
991.0653	0.6685	3188.4699	0.1927
1005.1064	0.7398	3188.557	0.3047
1008.3282	0.5144	3205.6079	0.0337
1011.3105	1.6778	3205.6364	0.0515
1022.8678	1.2557		

Table S71. Cartesian coordinates for the optimized geometry of isomer 5b-singlet of $Co^{+}(C_2H_2)_5$.

Z	x	y	z
27	-0.177677000	0.000003000	0.154456000
6	-1.578953000	1.248999000	-0.063863000
6	-2.851071000	0.706765000	0.021801000
1	-3.761608000	1.296958000	0.050621000
1	-1.450840000	2.322017000	-0.193132000
6	-2.851068000	-0.706770000	0.021803000
6	-1.578947000	-1.248998000	-0.063862000
1	-1.450828000	-2.322014000	-0.193140000
1	-3.761601000	-1.296968000	0.050628000
6	1.551237000	1.209615000	-0.778803000
6	1.698903000	1.213881000	0.620168000
6	1.497810000	0.000101000	-1.484249000
1	1.736922000	2.151726000	1.156617000
1	1.377911000	0.000180000	-2.558352000
6	1.787077000	-0.000104000	1.317382000
6	1.551218000	-1.209514000	-0.778981000
1	1.887958000	-0.000183000	2.394337000
1	1.465976000	-2.146967000	-1.311425000
6	1.698879000	-1.213985000	0.619993000
1	1.736878000	-2.151909000	1.156305000
1	1.466006000	2.147146000	-1.311110000

Table S72. Predicted frequencies (cm^{-1}) and IR intensities (km/mol) for isomer 5b-singlet of $Co^{+}(C_2H_2)_5$.

Frequency (cm^{-1})	Intensity (km/mol)	Frequency (cm^{-1})	Intensity (km/mol)
10.6527	0.0014	1044.4446	0.6512
72.6278	1.7816	1051.9261	1.6227
121.7542	0.5587	1053.5935	0.758
170.026	0.6117	1103.7892	2.2816
210.5131	2.1512	1116.3813	0.329
228.2687	3.6196	1189.4063	0.0004
253.4288	3.5558	1195.136	0.2583
326.2017	4.3054	1195.7326	0.2854
394.9954	0.6182	1260.2166	4.1874
403.0331	13.8591	1343.7349	1.7598
406.9895	0.1638	1344.3112	0.2456
479.8619	0.001	1386.5776	0.0002
590.0323	2.2274	1470.0257	51.5232
616.1649	0.0293	1486.5901	1.8192
617.5171	1.1895	1499.8402	21.7881
673.2213	53.4229	1505.7964	19.6258
686.3929	5.1698	1583.5212	0.952
772.0821	0.9088	1591.8381	0.774
780.4118	65.412	3125.5102	3.2433
794.1375	0.5968	3129.3674	1.1069
918.1683	0.0432	3161.6476	1.1599
932.2417	1.6193	3175.0449	0.6126
962.2352	4.6102	3198.1277	0.1224
988.6681	3.0506	3203.9377	0.0197
999.9743	0.8896	3203.9824	0.2701
1010.2231	0.5685	3214.1171	3.6748
1018.1841	0.3977	3215.6372	4.2047
1024.7889	0.1866	3221.227	0.3985
1044.3516	0.7369		

Table S73. Cartesian coordinates for the optimized geometry of isomer 5c-singlet of $Co^{+}(C_2H_2)_5$.

Z	x	y	z
27	0.208220000	-0.000185000	-0.000174000
6	1.920917000	-0.128812000	1.014418000
6	1.921305000	1.014227000	0.128775000
1	1.992059000	2.081204000	0.264421000
1	1.992268000	-0.264179000	2.081388000
1	1.992508000	-2.081323000	-0.264226000
6	1.921576000	-1.014327000	-0.128828000
1	1.992560000	0.264090000	-2.081423000
6	1.921315000	0.128689000	-1.014448000
6	-1.425603000	0.799423000	-1.158872000
6	-1.430444000	1.404656000	0.112003000
6	-1.424898000	-0.605058000	-1.270743000
1	-1.395913000	2.481556000	0.197851000
1	-1.384222000	-1.068756000	-2.246286000
6	-1.423995000	0.605398000	1.270983000
6	-1.431282000	-1.404348000	-0.111737000
1	-1.382558000	1.068973000	2.246549000
1	-1.397464000	-2.481260000	-0.197683000
6	-1.425542000	-0.799115000	1.159097000
1	-1.385581000	-1.411423000	2.048930000
1	-1.385680000	1.411720000	-2.048717000

Table S74. Predicted frequencies (cm^{-1}) and IR intensities (km/mol) for isomer 5c-singlet of $Co^{+}(C_2H_2)_5$.

Frequency (cm^{-1})	Intensity (km/mol)	Frequency (cm^{-1})	Intensity (km/mol)
3.3788	0	1017.6644	0.0016
151.3572	0.6268	1040.7046	1.3518
152.1984	0.5826	1042.711	1.4213
257.515	1.9365	1046.3462	0.0015
308.6653	7.2723	1185.301	0.0018
314.5043	6.6341	1186.2786	0.0275
408.3517	0.0002	1189.0998	0.0006
410.7404	0.0082	1206.7268	0.0001
424.0645	16.7571	1207.1891	0
424.7163	16.7117	1276.0068	3.7828
501.6855	44.1206	1366.0571	4.3942
608.562	0.0026	1370.3773	8.5803
616.8484	0.0005	1377.0233	4.1863
622.4488	0.0036	1380.5779	0
677.0035	0.0233	1493.7325	16.4817
811.6788	33.4597	1494.6381	16.2826
832.6717	0.1879	1565.6839	0.0687
833.2962	0.2186	1567.1998	0.0062
842.2215	32.6889	3202.3712	0.0028
877.7366	0.0001	3207.3726	0.0003
932.854	1.7239	3207.7053	0.0023
933.736	1.464	3216.8614	3.1397
953.8104	0	3217.2843	3.1389
962.2358	0.0001	3222.7548	0.0084
965.0833	7.5929	3235.6445	0
965.3692	7.6812	3252.5296	3.6451
1000.3411	0.0189	3252.5956	3.6704
1000.7083	0.0068	3267.2876	5.6993
1002.3786	0.038		

Table S75. Cartesian coordinates for the optimized geometry of isomer 5d-singlet of $Co^{+}(C_2H_2)_5$.

Z	x	y	z
6	1.328193000	1.465765000	-0.485377000
6	1.926117000	0.759208000	0.577667000
6	1.375720000	-0.314169000	1.360255000
6	0.350772000	-1.287031000	1.168053000
6	-0.649501000	-1.621641000	0.177038000
6	-1.837990000	-1.081429000	-0.431434000
6	-2.605087000	0.019235000	-0.141224000
6	-2.177566000	1.109054000	0.676736000
6	-0.947267000	1.642538000	0.542036000
6	-0.073494000	1.440705000	-0.617077000
1	-0.504368000	1.699711000	-1.581222000
1	-0.598710000	2.364117000	1.274901000
1	-2.867615000	1.520493000	1.406131000
1	-3.631066000	0.005225000	-0.494417000
1	-2.291292000	-1.794315000	-1.112396000
1	-0.663163000	-2.704589000	0.068250000
1	0.619079000	-2.177278000	1.730096000
1	2.106213000	-0.685116000	2.070957000
1	2.985055000	0.914458000	0.750820000
1	1.966862000	1.977710000	-1.196870000
27	0.842208000	-0.515327000	-0.736159000

Table S76. Predicted frequencies (cm^{-1}) and IR intensities (km/mol) for isomer 5d-singlet of $Co^+(C_2H_2)_5$.

Frequency (cm^{-1})	Intensity (km/mol)	Frequency (cm^{-1})	Intensity (km/mol)
87.3021	0.4166	1016.4058	0.2887
134.487	0.6031	1048.2188	0.3326
179.6185	1.4744	1116.0949	1.8397
203.2267	2.2945	1181.3571	4.1178
223.1724	1.1583	1214.2434	1.4285
268.1101	2.1509	1235.617	1.7591
278.9629	0.8837	1291.2152	3.1647
325.8252	2.6159	1295.4545	1.6915
350.9428	3.6898	1358.5654	2.0111
406.442	2.8811	1393.9874	2.6765
433.0157	4.2521	1420.2314	4.9452
463.317	4.7878	1449.3304	2.9687
528.5082	2.8278	1457.9474	0.2339
635.0244	6.9163	1490.3652	2.4276
670.0921	4.5591	1494.5325	9.8358
699.5721	35.4142	1511.4426	3.6876
731.8772	8.781	1544.7505	12.9768
781.6858	72.5351	1609.8211	18.251
825.8029	19.8358	3098.6158	0.1278
835.4169	4.4877	3126.2966	0.2289
843.1371	12.7792	3127.0329	0.974
861.1952	4.3167	3146.0429	0.0893
895.6027	0.7704	3152.099	1.1773
914.8201	11.5701	3152.8488	0.1293
944.7717	3.6191	3163.3346	1.1333
947.1404	0.7754	3164.5784	0.0997
980.7073	1.4927	3170.6801	0.9024
989.8375	0.9756	3175.037	1.5911
1007.9417	2.4706		

Table S77. Cartesian coordinates for the optimized geometry of isomer 5e-singlet of $Co^{+}(C_2H_2)_5$.

Z	x	y	z
6	-1.401162000	0.000024000	1.419459000
6	-1.376852000	-1.205619000	0.704911000
6	-1.376890000	1.205638000	0.704860000
1	-1.329201000	-2.147328000	1.233987000
1	-1.329263000	2.147370000	1.233898000
6	-1.376867000	-1.205646000	-0.704860000
6	-1.376892000	1.205610000	-0.704909000
1	-1.329218000	-2.147377000	-1.233898000
1	-1.329269000	2.147321000	-1.233984000
6	-1.401176000	-0.000034000	-1.419458000
1	-1.405769000	-0.000056000	-2.500270000
1	-1.405733000	0.000046000	2.500270000
27	0.352212000	0.000001000	-0.000003000
6	1.696833000	-1.436610000	-0.614452000
6	1.696817000	-1.436633000	0.614458000
1	1.938446000	-1.674985000	1.629112000
1	1.938484000	-1.674944000	-1.629106000
1	1.938524000	1.674930000	-1.629106000
6	1.696846000	1.436628000	-0.614452000
1	1.938443000	1.674997000	1.629110000
6	1.696816000	1.436643000	0.614455000

Table S78. Predicted frequencies (cm^{-1}) and IR intensities (km/mol) for isomer 5e-singlet of $Co^+(C_2H_2)_5$.

Frequency (cm^{-1})	Intensity (km/mol)	Frequency (cm^{-1})	Intensity (km/mol)
20.886	0	995.877	0.5678
133.2232	0.0328	1007.5955	0.8235
155.1531	0	1017.7281	0
165.8204	1.1685	1019.8643	0.7058
178.7497	0.2471	1036.9128	0.4862
209.4423	0.144	1043.6631	0.1805
254.9599	3.2486	1058.7047	1.0699
256.9687	0.7912	1186.638	0
258.3216	6.0528	1191.7075	0.0496
330.0569	30.1484	1197.5724	0.2626
346.781	15.1239	1371.9717	0.6719
382.1256	0	1384.6228	0
401.0117	5.0743	1493.9097	22.2478
414.4359	0	1508.7815	16.9972
422.6274	14.28	1574.6899	1.0809
610.4359	0	1587.8171	0
616.979	0.0776	1885.6632	26.9643
673.2233	0.0003	1894.6118	26.6039
679.89	0	3199.5481	0.0013
690.7171	1.0998	3203.9886	0
728.9095	48.85	3206.4068	0.0025
737.7121	72.2855	3212.9879	2.9985
745.019	0	3217.8126	3.4343
749.4114	7.7863	3221.5594	0.0033
771.4255	26.6255	3325.5181	0.0001
779.7147	3.0041	3326.3857	155.7902
804.6844	58.4738	3400.7048	39.1966
912.6517	0.2932	3401.6688	49.6141
944.091	2.3936		

Table S79. Cartesian coordinates for the optimized geometry of isomer 5h-singlet of $Co^{+}(C_2H_2)_5$.

Z	x	y	z
6	0.281546000	1.849122000	0.713523000
6	-0.369650000	2.111079000	-0.543450000
6	-0.572812000	0.975269000	-1.264479000
6	2.509793000	-0.757470000	-0.545361000
6	1.653528000	-1.806024000	-0.021362000
6	0.398523000	-1.618055000	0.399860000
1	2.070107000	-2.808998000	0.051654000
1	-0.041451000	2.378321000	1.605496000
1	-0.787140000	3.084733000	-0.789751000
1	-0.971227000	0.826520000	-2.259624000
1	3.342528000	-1.012856000	-1.187300000
1	-0.146894000	-2.415800000	0.899772000
27	-0.661617000	-0.022256000	0.209944000
1	1.518805000	0.570941000	1.831628000
6	1.251903000	0.859989000	0.815556000
6	2.276325000	0.509335000	-0.192883000
1	2.885300000	1.326752000	-0.562568000
6	-2.709795000	-0.571887000	0.252756000
6	-2.175177000	-1.413801000	-0.446619000
1	-1.899600000	-2.248626000	-1.053011000
1	-3.371877000	0.074587000	0.789979000

Table S80. Predicted frequencies (cm^{-1}) and IR intensities (km/mol) for isomer 5h-singlet of $Co^+(C_2H_2)_5$.

Frequency (cm^{-1})	Intensity (km/mol)	Frequency (cm^{-1})	Intensity (km/mol)
44.3172	0.0618	943.2134	2.6035
80.3451	1.2644	987.2593	1.2059
143.3678	0.6938	1004.0712	1.7369
172.7554	4.5397	1031.9329	12.0248
186.3386	1.1435	1043.5317	3.5228
195.0575	0.7596	1075.508	1.5077
226.4594	3.5589	1152.0667	20.5721
256.6688	1.8205	1197.3495	2.165
262.6108	1.2677	1227.772	1.703
307.9805	0.7481	1248.9786	26.9482
346.3787	7.5634	1293.8917	44.3244
382.4308	2.9423	1392.9997	3.67
432.7555	1.9025	1441.0262	1.0754
454.9356	2.9539	1468.1068	6.2698
525.3433	0.4998	1533.3885	12.1292
552.5084	2.7437	1585.4207	7.0409
648.6891	35.5787	1664.8143	1.675
663.1628	8.4829	1948.599	2.914
694.4358	21.0405	3105.7069	0.1608
711.8045	13.7732	3112.0781	1.0053
757.9485	25.6108	3132.1637	3.7514
772.8143	38.4875	3134.7156	2.4294
782.667	24.1829	3151.0626	0.4987
789.4831	21.8563	3168.5491	2.4868
795.819	1.6626	3201.6177	0.224
808.8521	2.4689	3208.8973	7.35
878.0469	13.1748	3344.2301	138.4885
925.7654	0.7402	3433.2604	57.9365
938.0978	5.7724		

Table S81. Cartesian coordinates for the optimized geometry of isomer 5i-singlet of $Co^{+}(C_2H_2)_5$.

Z	x	y	z
6	-2.505459000	-0.820503000	0.402103000
6	-2.291962000	0.023084000	-0.680713000
6	-1.125634000	0.695411000	-1.033362000
6	2.505482000	-0.820442000	-0.402096000
6	2.291931000	0.023095000	0.680744000
6	1.125562000	0.695362000	1.033402000
1	3.154165000	0.230463000	1.316242000
1	-3.531080000	-1.030730000	0.682576000
1	-3.154216000	0.230433000	-1.316188000
1	-0.976710000	0.951033000	-2.088236000
1	3.531114000	-1.030637000	-0.682551000
1	0.976596000	0.950910000	2.088287000
27	-0.000003000	1.683309000	0.000005000
1	-1.716745000	-1.752730000	2.161739000
6	-1.474305000	-1.424969000	1.156773000
6	1.474350000	-1.424901000	-1.156814000
1	1.716809000	-1.752603000	-2.161795000
1	0.520839000	-2.044851000	1.405166000
6	-0.205595000	-1.652728000	0.700307000
6	0.205648000	-1.652710000	-0.700366000
1	-0.520781000	-2.044816000	-1.405239000

Table S82. Predicted frequencies (cm^{-1}) and IR intensities (km/mol) for isomer 5i-singlet of $Co^+(C_2H_2)_5$.

Frequency (cm^{-1})	Intensity (km/mol)	Frequency (cm^{-1})	Intensity (km/mol)
91.0786	0.6084	996.1452	0.5036
106.2861	1.4576	1013.2701	1.2178
106.8571	0.328	1071.8849	9.265
156.2875	1.5527	1109.0005	0.0393
160.5172	0.7412	1170.1004	18.9877
203.1227	1.667	1188.5023	8.7691
255.0653	2.3778	1224.7773	3.4416
283.201	1.402	1241.4976	19.3704
349.5466	0.9354	1256.6282	0.4328
358.5789	0.8009	1284	7.4321
446.3119	11.8731	1379.7494	12.1711
454.1505	1.5813	1434.0742	12.0498
482.6377	0	1438.2877	3.6117
527.6515	15.5626	1487.7979	5.3305
540.8328	8.1315	1495.1816	3.0085
637.031	12.4727	1505.3407	4.9358
646.6116	7.9228	1550.8761	15.9948
650.5824	89.0931	1585.6216	12.4001
677.1743	64.0074	3048.0449	4.3846
772.2671	0.3296	3048.4598	0.5651
822.4062	3.8363	3094.5226	5.9095
830.0796	11.4331	3094.6239	0.0272
851.6306	8.3821	3145.8	0
860.0641	1.736	3153.1868	0.8488
947.9618	4.1735	3167.6877	0.3867
948.6387	7.6757	3167.7161	0.1716
964.1709	8.7261	3180.2606	1.1194
966.8094	0.4592	3180.3526	0.0143
987.5528	6.0116		

Table S83. Cartesian coordinates for the optimized geometry of isomer 5j-singlet of $Co^{+}(C_2H_2)_5$.

Z	x	y	z
6	0.141962000	1.504509000	0.911062000
6	1.493173000	0.798430000	0.595113000
6	1.261417000	1.406144000	-0.801276000
6	0.187334000	2.159533000	-0.369856000
1	-0.301194000	1.792810000	1.853028000
1	2.335312000	1.204978000	1.153069000
1	1.838863000	1.382740000	-1.713202000
1	-0.419718000	2.924599000	-0.831957000
27	-0.401580000	-0.000837000	-0.192464000
6	1.496630000	-0.791911000	0.595009000
1	2.340434000	-1.194851000	1.153069000
6	0.148522000	-1.504109000	0.910727000
6	1.267763000	-1.400515000	-0.801452000
1	1.845187000	-1.374390000	-1.713318000
1	-0.293333000	-1.794635000	1.852620000
6	0.197048000	-2.158780000	-0.370250000
1	-0.406494000	-2.926518000	-0.832519000
6	-2.286089000	-0.005511000	0.630146000
6	-2.369081000	-0.004566000	-0.598019000
1	-2.763934000	-0.004363000	-1.594472000
1	-2.564542000	-0.007102000	1.662976000

Table S84. Predicted frequencies (cm^{-1}) and IR intensities (km/mol) for isomer 5j-singlet of $Co^+(C_2H_2)_5$.

Frequency (cm^{-1})	Intensity (km/mol)	Frequency (cm^{-1})	Intensity (km/mol)
65.6891	0.021	969.6436	9.1889
109.7857	0.1418	971.9807	3.1428
152.3269	0.4658	976.6037	14.2731
158.3585	10.3466	1012.5005	0.0391
166.4739	0.1041	1024.1358	9.5689
197.1573	3.1672	1062.0192	20.9577
239.2075	2.9296	1095.5274	4.0348
354.3913	0.1994	1152.8884	3.6641
360.0531	0.0063	1156.7641	0.9732
383.2753	9.5448	1184.1307	4.6284
395.5922	16.6184	1214.7168	0.1291
424.43	3.9043	1221.8929	1.9362
524.2463	0.095	1298.9047	0.4157
533.0613	10.16	1350.7185	6.7624
679.6181	6.5906	1355.0955	17.3542
728.9183	58.9195	1469.8872	6.177
732.1323	39.8134	1477.9443	1.9812
736.3888	10.3119	1878.5224	38.7241
763.1408	12.3639	3094.3103	0.2794
776.0454	3.2105	3104.251	0.9772
811.1522	15.2093	3212.0386	0.6104
813.4199	15.6499	3212.8917	1.378
847.5566	8.8705	3224.0924	0.1191
867.6852	1.3448	3225.7094	1.8934
901.6426	5.1406	3241.1282	2.9874
904.2319	2.4823	3243.3362	2.7322
924.446	3.5628	3315.8758	92.2049
926.1434	0.3909	3393.8725	52.5417
954.9676	14.0707		

Table S85. Cartesian coordinates for the optimized geometry of isomer 5k-singlet of $Co^{+}(C_2H_2)_5$.

Z	x	y	z
6	1.647518000	-0.721180000	-1.024169000
6	2.086177000	0.369505000	0.000207000
6	1.647318000	-0.721559000	1.024144000
6	1.558807000	-1.679389000	-0.000199000
1	1.720945000	-0.774032000	-2.099909000
1	3.165253000	0.505875000	0.000400000
1	1.720586000	-0.774809000	2.099874000
1	1.423575000	-2.751575000	-0.000405000
27	-0.025576000	-0.016716000	-0.000016000
6	1.383075000	1.770399000	0.000226000
6	0.058088000	1.815500000	-0.000301000
1	-0.653941000	2.625454000	-0.000580000
1	2.025818000	2.645922000	0.000679000
1	-3.595552000	-0.154817000	1.297592000
6	-2.687149000	-0.150241000	0.703832000
6	-1.419152000	-0.234797000	1.248736000
1	-1.265721000	-0.546956000	2.278806000
1	-3.595516000	-0.155261000	-1.297539000
6	-2.687124000	-0.150378000	-0.703765000
6	-1.419102000	-0.234805000	-1.248660000
1	-1.265639000	-0.546787000	-2.278777000

Table S86. Predicted frequencies (cm^{-1}) and IR intensities (km/mol) for isomer 5k-singlet of $Co^+(C_2H_2)_5$.

Frequency (cm^{-1})	Intensity (km/mol)	Frequency (cm^{-1})	Intensity (km/mol)
56.2598	1.1622	980.721	23.1763
102.1782	2.1748	991.7481	6.2896
102.5749	0.0006	1024.4428	2.0612
128.8738	0.0017	1026.7847	30.1304
202.4003	9.3453	1041.5896	3.0034
223.233	5.3163	1093.1935	3.2786
288.1948	11.0691	1108.4465	2.3439
351.2563	13.6447	1160.6431	22.0472
385.8625	7.4152	1203.4353	129.9327
395.415	12.4488	1209.1578	9.8153
414.2817	4.3396	1210.6687	2.6524
417.6489	0.1365	1237.4793	19.6342
493.7188	1.5224	1241.1107	11.0727
530.1771	8.7022	1388.1662	4.6528
578.6982	4.5467	1418.4818	6.9649
682.2074	52.7399	1467.7475	24.4495
691.4801	34.6007	1474.9043	37.1114
724.1692	11.4491	1569.9454	1.1743
768.4049	0.248	3113.2421	6.9913
770.5685	0.1659	3139.7722	0.0012
775.0447	0.0262	3143.37	2.2726
802.6604	1.8246	3146.2577	10.1251
853.4009	8.643	3165.2872	0.0584
885.5778	2.3208	3177.6941	0.1381
909.0578	4.9419	3217.1883	4.6325
910.492	9.6213	3233.7393	4.6903
937.9104	30.2999	3242.9489	5.1211
949.0865	7.9774	3250.3501	8.8195
977.5445	30.6209		

Table S87. Cartesian coordinates for the optimized geometry of isomer 5l-singlet of $Co^+(C_2H_2)_5$.

Z	x	y	z
6	1.485881000	1.010143000	-0.579131000
6	2.057210000	0.000140000	0.445181000
6	1.486097000	-1.010785000	-0.578270000
6	1.255265000	-0.000791000	-1.526745000
1	1.600500000	2.080890000	-0.653273000
1	3.147219000	0.000250000	0.489904000
1	1.600475000	-2.081639000	-0.651288000
1	0.972474000	-0.001291000	-2.571262000
27	-0.432437000	0.000035000	-0.206191000
6	1.364996000	0.000637000	1.774737000
1	1.907808000	0.000911000	2.714321000
6	0.055100000	0.000692000	1.663726000
1	-0.700288000	0.001116000	2.436360000
6	-1.941169000	-1.345299000	0.180835000
6	-1.074366000	-1.973054000	-0.424638000
1	-0.579590000	-2.782893000	-0.914213000
1	-2.867817000	-1.179862000	0.687575000
1	-2.868613000	1.180008000	0.685046000
6	-1.941345000	1.345159000	0.179332000
6	-1.073912000	1.972918000	-0.425312000
1	-0.578924000	2.782989000	-0.914297000

Table S88. Predicted frequencies (cm^{-1}) and IR intensities (km/mol) for isomer 5l-singlet of $Co^{+}(C_2H_2)_5$.

Frequency (cm^{-1})	Intensity (km/mol)	Frequency (cm^{-1})	Intensity (km/mol)
32.9279	2.2226	892.3253	5.6648
92.2336	0.1334	908.0274	8.5877
119.8297	0.2567	914.354	0.3857
168.8788	0.4605	936.6613	15.3642
179.2176	3.2968	987.9488	6.2252
190.6788	14.1441	1012.3843	0.6265
220.1751	4.2659	1018.1559	9.6326
247.3191	0.5049	1070.6508	4.9547
282.255	1.5902	1146.5096	11.6982
301.6193	5.1427	1175.2139	12.5389
324.6694	6.0828	1222.7409	2.6675
343.5042	25.8934	1254.5319	38.8516
413.7628	11.9816	1321.7844	0.366
421.0796	4.7868	1395.753	5.5532
438.1723	4.41	1431.2344	1.2276
467.6196	0.0027	1657.1452	20.5211
558.6799	11.829	1889.2205	6.1602
682.8151	23.3484	1894.0738	11.2488
688.224	14.8177	3084.4214	1.9901
691.2351	11.7554	3158.8978	1.4425
707.2563	4.2902	3201.3639	2.2722
727.7055	6.6666	3215.7617	1.0146
747.0334	88.0443	3230.9816	0.0162
794.4493	10.9889	3235.7298	2.3983
802.5266	4.9157	3338.79	26.6783
811.7392	1.5632	3342.0537	102.3561
817.3739	0.0004	3414.3745	57.1836
860.0861	2.0101	3416.1476	21.8974
882.68	5.9937		

Table S89. Cartesian coordinates for the optimized geometry of isomer 5m-singlet of $Co^{+}(C_2H_2)_5$.

Z	x	y	z
6	1.422504000	-1.240708000	0.738462000
6	1.422156000	-1.244547000	-0.734741000
6	1.901017000	0.083437000	-0.745669000
6	1.901225000	0.087642000	0.742037000
1	1.274742000	-1.988905000	1.498868000
1	1.272814000	-1.996610000	-1.491041000
1	2.286886000	0.739432000	-1.507304000
1	2.288085000	0.747379000	1.499905000
27	0.000057000	0.089649000	-0.000502000
1	-2.282795000	0.733721000	1.514553000
6	-1.898652000	0.080891000	0.749348000
6	-1.420035000	-1.247304000	0.731837000
1	-1.269600000	-2.002550000	1.484729000
6	-1.424555000	-1.237785000	-0.741384000
1	-1.277964000	-1.982732000	-1.505207000
6	-1.904007000	0.090100000	-0.738127000
1	-2.292510000	0.753062000	-1.492351000
6	0.000651000	2.114533000	0.615652000
6	-0.000525000	2.114650000	-0.615561000
1	0.001728000	2.485287000	1.618989000
1	-0.001599000	2.485951000	-1.618710000

Table S90. Predicted frequencies (cm^{-1}) and IR intensities (km/mol) for isomer 5m-singlet of $Co^+(C_2H_2)_5$.

Frequency (cm^{-1})	Intensity (km/mol)	Frequency (cm^{-1})	Intensity (km/mol)
82.8436	0.106	920.4953	11.2726
89.2988	0.0002	935.22	0.5223
169.0957	0.0002	951.621	0.0002
197.1568	4.0566	961.025	0.2238
204.7147	4.7288	990.7128	0.0494
211.9847	5.8286	1003.4633	0.0007
322.1622	8.0107	1003.6112	16.7286
348.3974	12.663	1149.2753	0.1444
356.4058	0.0032	1201.7106	1.4289
369.3182	0.4165	1205.0186	0.0009
371.8607	2.7803	1207.7856	0.0041
383.9065	6.2125	1319.5679	0.3224
439.9867	42.0718	1326.2792	17.8061
462.4489	45.9684	1330.9207	3.8482
595.9847	0.427	1340.954	11.853
612.4935	0.0008	1422.905	14.3061
707.9565	0.0003	1430.3068	0.0004
740.2243	36.1738	1877.88	21.0631
763.5644	14.655	3244.7221	0.0001
763.8432	1.1018	3248.8639	0.3863
811.474	0.006	3260.158	1.1416
816.542	0.1798	3263.6667	7.639
825.2508	72.5104	3265.7478	0.0003
833.9114	9.9193	3268.2723	11.2664
863.4821	0.0035	3277.2637	12.5942
864.3277	11.6735	3280.3862	1.3019
887.438	0.5256	3327.9414	78.1231
891.7878	0.0031	3400.5714	39.9659
913.968	1.9157		

Table S91. Cartesian coordinates for the optimized geometry of isomer 5n-singlet of $Co^{+}(C_2H_2)_5$.

Z	x	y	z
6	1.574624000	-0.000016000	-0.720056000
6	1.967474000	1.198876000	0.092462000
6	1.375960000	-0.000006000	0.801754000
6	1.967458000	-1.198902000	0.092481000
1	0.992885000	-0.000018000	-1.630237000
1	3.005399000	1.476767000	0.236302000
1	0.647754000	0.000009000	1.625745000
1	3.005378000	-1.476807000	0.236327000
27	-0.706123000	0.000011000	0.186766000
6	0.918632000	2.221469000	-0.118261000
1	1.159567000	3.280903000	-0.098595000
6	-0.335311000	1.793487000	-0.342428000
1	-1.152259000	2.424881000	-0.668540000
6	-2.536956000	-0.000022000	-0.614764000
6	-2.645186000	0.000028000	0.614377000
1	-3.038805000	0.000059000	1.611978000
1	-2.781578000	-0.000064000	-1.657903000
1	-1.152281000	-2.424863000	-0.668558000
6	-0.335333000	-1.793477000	-0.342432000
6	0.918598000	-2.221479000	-0.118239000
1	1.159511000	-3.280917000	-0.098560000

Table S92. Predicted frequencies (cm^{-1}) and IR intensities (km/mol) for isomer 5n-singlet of $Co^{+}(C_2H_2)_5$.

Frequency (cm^{-1})	Intensity (km/mol)	Frequency (cm^{-1})	Intensity (km/mol)
100.0998	1.1445	963.5985	22.794
125.1573	1.432	976.3867	0.4437
174.6439	1.8466	1001.1967	0.9153
190.7198	1.7138	1007.0137	3.2152
218.9547	1.1035	1057.6738	4.0896
259.2193	2.3967	1082.3564	3.6578
283.1327	3.4128	1089.7713	16.7667
355.511	1.6569	1115.7944	20.9815
362.3286	2.8576	1126.298	8.0018
372.42	1.7799	1206.6725	7.8732
440.7411	6.7421	1207.1811	2.5735
480.7805	13.5251	1263.5477	56.2667
493.4496	6.7912	1268.018	24.7535
546.3481	8.473	1332.5024	0.2737
554.0942	0.0056	1362.0123	11.9922
674.3282	21.4624	1539.6885	4.1681
683.8156	18.4013	1551.0089	2.797
718.0565	53.3753	1858.0945	4.2638
745.7279	1.6306	2976.8418	2.9661
759.209	25.5577	3147.5165	10.8528
764.7205	20.5292	3147.6155	4.0173
764.9849	13.111	3163.1258	0.0238
766.6062	3.5603	3164.0331	0.2198
774.0665	17.5289	3191.4735	0.6415
855.3386	51.1943	3191.5842	4.8283
875.4254	0.367	3202.8353	5.8635
889.0238	6.6113	3307.9774	114.9921
907.3549	31.3141	3383.7922	57.4402
930.6587	50.7998		

Table S93. Cartesian coordinates for the optimized geometry of isomer 5o-singlet of $Co^{+}(C_2H_2)_5$.

Z	x	y	z
27	-0.002262000	-0.720545000	0.001005000
6	-2.031556000	-1.201864000	0.146271000
6	-1.443168000	-2.194342000	-0.273830000
1	-1.240662000	-3.178327000	-0.641079000
1	-2.795514000	-0.516923000	0.459224000
6	-0.588057000	1.298465000	-0.171163000
6	0.588858000	1.296799000	0.171627000
1	1.582739000	1.597704000	0.438494000
1	-1.581839000	1.598614000	-0.439295000
6	1.447221000	-2.195657000	0.269724000
6	2.034100000	-1.199917000	-0.142445000
1	2.794776000	-0.509069000	-0.450229000
1	1.240074000	-3.180339000	0.632085000
6	4.386487000	1.547692000	0.497434000
6	4.136277000	1.932157000	-0.610046000
1	3.953475000	2.299266000	-1.592797000
1	4.650497000	1.227799000	1.478229000
6	-4.135254000	1.934728000	0.607488000
6	-4.385075000	1.546547000	-0.498775000
1	-4.648732000	1.223314000	-1.478560000
1	-3.952726000	2.305030000	1.589083000

Table S94. Predicted frequencies (cm^{-1}) and IR intensities (km/mol) for isomer 5o-singlet of $Co^{+}(C_2H_2)_5$.

Frequency (cm^{-1})	Intensity (km/mol)	Frequency (cm^{-1})	Intensity (km/mol)
21.6879	0.0946	741.155	16.192
26.4552	0.2114	768.7919	0.677
34.3762	0.2149	771.5501	0.3595
49.3038	0.0131	778.6118	91.9152
56.1811	0.0017	778.8729	79.7596
61.4398	0.0629	789.144	167.7021
71.6199	1.2716	791.7744	121.5825
74.9137	3.9003	791.8877	127.468
90.7903	0.0433	794.0882	2.6881
94.4759	2.0151	798.5052	110.9077
110.8368	0.6425	811.149	0.0582
116.6292	0.9556	836.0256	9.7989
128.526	0.6195	848.9003	2.154
135.2972	0.0192	1898.4245	5.1832
170.9757	3.6485	1902.9961	4.5998
181.2169	8.1774	1909.0007	1.2435
260.5797	1.3978	2061.827	15.1252
268.6129	0.462	2062.0709	4.4558
273.0096	2.1649	3289.582	212.3482
408.8738	8.3327	3304.0757	332.3451
412.9228	6.3067	3304.6257	269.0455
421.0665	1.6912	3375.4614	94.816
641.8207	0.0017	3395.8305	46.2855
641.9482	0.0103	3395.9547	170.7719
670.4105	0.193	3396.2269	103.4813
670.7309	0.6035	3399.1902	26.3829
697.3888	0.7304	3495.6195	1.7481
706.8911	12.4827	3495.7098	0.9367
725.4802	0.1356		

Table S95. Cartesian coordinates for the optimized geometry of isomer 5q-singlet of $Co^{+}(C_2H_2)_5$.

Z	x	y	z
27	-0.542357000	-0.000281000	-0.140856000
6	-1.128751000	0.597280000	-2.059219000
6	-1.130426000	-0.628128000	-2.049296000
1	-1.187653000	-1.658673000	-2.329043000
1	-1.183213000	1.623406000	-2.355310000
6	-0.238598000	2.127838000	0.622352000
6	0.855240000	1.650442000	0.405864000
1	1.888149000	1.402747000	0.262450000
1	-1.114606000	2.685812000	0.866531000
6	-0.239955000	-2.119560000	0.650479000
6	0.854431000	-1.644540000	0.431891000
1	1.887579000	-1.397832000	0.288613000
1	-1.116844000	-2.674422000	0.898510000
6	4.224260000	0.005911000	0.661579000
6	4.146086000	-0.006121000	-0.534501000
1	4.126164000	-0.016873000	-1.599083000
1	4.339103000	0.016568000	1.720293000
6	-2.946138000	0.006302000	0.712063000
6	-2.324818000	0.011905000	1.742369000
1	-1.844365000	0.017134000	2.694216000
1	-3.578649000	0.001751000	-0.145543000

Table S96. Predicted frequencies (cm^{-1}) and IR intensities (km/mol) for isomer 5q-singlet of $Co^+(C_2H_2)_5$.

Frequency (cm^{-1})	Intensity (km/mol)	Frequency (cm^{-1})	Intensity (km/mol)
5.2039	0.1175	696.4864	0.9529
18.0088	0.0719	702.7156	27.1109
50.3304	0.2901	712.9007	17.1078
64.5542	0.0259	752.9865	91.8663
67.5738	0.1813	758.6904	18.3761
68.6879	1.1071	771.9383	30.5528
79.1533	0.1659	780.3699	162.0784
97.666	2.7121	785.3748	23.9467
108.8432	1.2874	790.5439	144.3259
116.46	1.0342	795.8731	5.8003
119.1682	0.0097	801.3673	32.6622
126.0489	0.0175	804.9615	116.92
137.5156	0.1269	818.0194	0.039
138.0435	0.247	1911.6553	12.8118
151.9358	1.1685	1972.6793	3.6941
166.2727	0.13	1976.3756	1.7283
176.7426	1.5066	2037.3408	6.5402
208.3717	0.5223	2062.1976	8.399
271.0481	0.7514	3315.5105	40.9918
278.2636	6.6029	3323.0018	527.9083
292.1059	3.5963	3336.1223	102.5616
383.0402	0.5223	3384.0682	132.6387
641.9315	0.0106	3395.7407	118.7971
661.1713	0.0682	3415.4516	41.4697
667.9254	1.0829	3435.8453	51.086
674.8214	1.2587	3438.2561	14.1133
683.2944	0.0234	3481.9122	9.38
683.6659	2.3229	3495.5542	1.317
688.7656	5.8259		

Table S97. Cartesian coordinates for the optimized geometry of isomer 5r-singlet of $Co^{+}(C_2H_2)_5$.

Z	x	y	z
6	3.225098000	-0.277329000	0.804531000
6	3.244948000	-0.715145000	-0.342436000
1	3.526397000	-1.218822000	-1.243785000
1	3.472712000	-0.048176000	1.820193000
6	0.828336000	1.778239000	1.384893000
6	0.122384000	0.800488000	1.538403000
1	-0.579279000	0.024491000	1.774984000
1	1.348736000	2.712116000	1.404670000
6	0.905653000	0.382565000	-2.227166000
6	0.185159000	-0.446813000	-1.707594000
1	-0.523398000	-1.183194000	-1.381500000
1	1.447390000	1.062467000	-2.849582000
6	-2.431329000	-1.705189000	0.589927000
6	-1.616342000	-2.527306000	0.904701000
1	-0.936253000	-3.292787000	1.196842000
1	-3.186471000	-0.997810000	0.320025000
6	-5.026322000	0.674808000	-0.874809000
6	-5.069265000	1.002915000	0.276352000
1	-5.141445000	1.305095000	1.293865000
1	-5.023058000	0.395293000	-1.901259000

Table S98. Predicted frequencies (cm^{-1}) and IR intensities (km/mol) for isomer 5r-singlet of $Co^+(C_2H_2)_5$.

Frequency (cm^{-1})	Intensity (km/mol)	Frequency (cm^{-1})	Intensity (km/mol)
3.506	0.0159	698.3546	0.0677
12.5064	0.4543	707.1458	24.3578
20.2443	0.4869	729.7638	37.5158
23.9121	0.0364	754.0011	85.2558
28.9954	0.4267	757.9352	56.9963
51.0671	0.5065	768.3763	73.9209
68.1884	0.0757	771.3753	17.5229
70.2372	0.3592	782.3428	186.141
74.9733	2.113	786.0638	13.8555
83.4204	1.216	797.1801	13.0022
96.8742	0.0339	807.1338	40.2308
104.6125	1.4861	815.0014	114.9188
125.3388	2.4887	819.9628	58.9822
128.6255	1.1794	1902.548	12.9769
130.8749	1.4706	1956.4307	1.3956
141.9905	0.0055	1962.9913	1.7517
184.5515	0.0532	2052.6298	17.7124
230.437	0.5078	2067.2227	1.8557
290.0194	0.2683	3310.8376	19.9416
313.8183	5.006	3315.2801	479.2248
335.7694	10.2772	3328.3991	110.0506
414.815	0.5183	3352.3665	278.9815
630.8667	0.0074	3406.2665	100.2155
649.5217	0.4561	3406.735	50.6838
664.9341	0.0517	3415.8722	37.3829
672.1994	7.6538	3419.3622	18.0949
679.8845	8.7314	3474.3461	1.6918
683.0617	17.3287	3505.259	0.5624
688.52	0.1621		

Table S99. Cartesian coordinates for the optimized geometry of isomer 5s-singlet of $Co^{+}(C_2H_2)_5$.

Z	x	y	z
27	-0.877621000	0.000015000	0.000604000
6	-2.136940000	1.392532000	-0.001352000
6	-3.294965000	0.750115000	-0.003312000
1	-4.244155000	1.281412000	-0.004958000
1	-1.879325000	2.443131000	-0.000853000
6	-3.294989000	-0.750019000	-0.003184000
6	-2.136983000	-1.392467000	-0.001121000
1	-1.879394000	-2.443072000	-0.000446000
1	-4.244196000	-1.281285000	-0.004730000
6	1.522556000	-0.000146000	0.754987000
6	0.368969000	0.000030000	1.403305000
1	0.104254000	0.000121000	2.451969000
1	2.480723000	-0.000240000	1.271626000
1	2.484511000	-0.000347000	-1.260462000
6	1.524746000	-0.000229000	-0.746749000
6	0.373072000	-0.000156000	-1.398447000
1	0.111340000	-0.000174000	-2.447859000
6	5.223482000	-0.598938000	-0.003298000
6	5.223164000	0.599232000	-0.003170000
1	5.264227000	1.662631000	-0.003101000
1	5.265114000	-1.662316000	-0.003442000

Table S100. Predicted frequencies (cm^{-1}) and IR intensities (km/mol) for isomer 5s-singlet of $Co^{+}(C_2H_2)_5$.

Frequency (cm^{-1})	Intensity (km/mol)	Frequency (cm^{-1})	Intensity (km/mol)
14.6149	0.1042	879.1931	11.4592
15.7771	0.0296	905.9767	11.3159
36.855	0	910.2287	5.9108
49.0512	0.6133	924.0861	0
50.2519	0.4678	934.0661	0.0001
57.5509	4.1546	1013.4026	46.3902
89.7965	0.2394	1022.153	42.1748
113.9736	0	1042.7901	51.3297
236.9729	0.2102	1055.7945	0.1312
237.518	0.5613	1205.4147	74.4192
290.2046	0.9946	1215.881	67.3949
312.7936	53.3517	1264.7521	21.1842
313.4412	0.0009	1291.0818	14.4454
448.4636	0.0107	1604.3143	23.3407
448.6107	0	1609.3835	0.3816
449.0642	0.0002	1626.7428	0
614.8767	118.9141	1627.1021	0.0071
618.9211	100.4912	2064.3066	14.3186
628.5219	2.2687	3119.2518	1.8922
629.5824	0.0055	3129.3468	1.4691
634.1208	0	3129.9779	108.0975
655.5275	4.4398	3140.7528	16.4812
743.8449	7.8565	3211.2249	47.6249
749.7016	14.7555	3213.3425	0.6623
758.3871	0	3213.4108	51.7661
763.6017	0	3215.656	1.5103
772.1373	96.7658	3402.8239	108.0422
784.3565	158.8705	3501.9243	1.1382
871.678	13.4927		

Table S101. Cartesian coordinates for the optimized geometry of isomer 5t-singlet of $Co^{+}(C_2H_2)_5$.

Z	x	y	z
27	0.729550000	0.156105000	0.000001000
6	-1.047637000	0.764095000	-0.000010000
6	-0.891503000	2.078430000	-0.000010000
1	-1.748441000	2.749116000	-0.000017000
1	-1.914460000	0.110581000	-0.000012000
6	0.491988000	2.661854000	0.000000000
6	1.537028000	1.848797000	0.000012000
1	2.604744000	2.021054000	0.000022000
1	0.608395000	3.743393000	-0.000001000
1	1.137247000	-0.757272000	2.443630000
6	1.226223000	-0.999474000	1.393173000
6	1.663515000	-2.071206000	0.750031000
1	2.025941000	-2.948749000	1.280849000
6	1.663526000	-2.071200000	-0.750030000
1	2.025961000	-2.948739000	-1.280849000
6	1.226245000	-0.999463000	-1.393172000
1	1.137285000	-0.757255000	-2.443628000
6	-4.336787000	-0.905228000	-0.599091000
6	-4.336790000	-0.905201000	0.599091000
1	-4.374688000	-0.917656000	1.662435000
1	-4.374676000	-0.917735000	-1.662435000

Table S102. Predicted frequencies (cm^{-1}) and IR intensities (km/mol) for isomer 5t-singlet of $Co^{+}(C_2H_2)_5$.

Frequency (cm^{-1})	Intensity (km/mol)	Frequency (cm^{-1})	Intensity (km/mol)
12.664	0.1523	873.0275	11.9909
22.8346	0.061	908.1587	16.291
32.9137	0.084	910.2619	1.2036
48.3298	0.8822	921.9536	0.0003
49.0552	0.4373	925.4379	0.027
64.5452	4.1742	1021.2232	40.8796
86.8308	0.5451	1023.48	57.3288
118.3355	0.2412	1047.293	25.1019
238.1988	1.7601	1059.0173	0.8431
247.7563	1.6384	1212.0455	71.2945
289.0499	0.317	1215.8006	65.8243
309.9779	0.1032	1269.4931	53.1099
311.3731	49.1323	1294.8801	0.5128
447.1742	0.0039	1603.8915	17.7774
448.0795	0.4757	1608.7806	2.0295
450.7728	0.0042	1627.2778	0
614.3795	133.4714	1627.3368	0.3948
629.557	1.5722	2064.3581	11.7872
629.6834	0.3232	3125.2142	7.0132
631.1959	77.0236	3129.4112	1.4626
632.894	6.1503	3137.6053	26.2331
654.9483	18.4115	3140.8218	17.2526
748.5785	11.11	3156.2442	289.1686
749.1979	15.5634	3211.5921	47.2006
759.5214	0.0335	3213.7108	0.6018
770.2256	86.9392	3214.7653	22.737
783.8458	1.0115	3403.953	105.1648
785.2573	139.5314	3502.9966	0.5293
870.751	13.9621		

Table S103. Cartesian coordinates for the optimized geometry of isomer 5u-singlet of $Co^+(C_2H_2)_5$.

Z	x	y	z
6	-2.953056000	-0.690096000	-0.000044000
6	-1.893150000	-1.612768000	0.000037000
6	-0.537297000	-1.434375000	0.000091000
6	-2.953062000	0.690079000	-0.000053000
6	-1.893165000	1.612761000	0.000011000
6	-0.537311000	1.434380000	0.000062000
1	-2.202521000	2.657028000	0.000020000
1	-3.936775000	-1.144405000	-0.000093000
1	-2.202497000	-2.657038000	0.000063000
1	0.127708000	-2.301773000	0.000168000
1	-3.936786000	1.144379000	-0.000108000
1	0.127688000	2.301783000	0.000109000
27	0.473294000	0.000007000	0.000005000
6	1.665202000	-0.000021000	-1.466783000
6	2.783085000	-0.000017000	-0.758136000
1	3.756726000	-0.000034000	-1.243969000
1	1.476470000	-0.000044000	-2.528856000
1	3.756773000	0.000012000	1.243849000
6	2.783115000	0.000009000	0.758052000
6	1.665260000	0.000025000	1.466741000
1	1.476563000	0.000042000	2.528820000

Table S104. Predicted frequencies (cm^{-1}) and IR intensities (km/mol) for isomer 5u-singlet of $Co^{+}(C_2H_2)_5$.

Frequency (cm^{-1})	Intensity (km/mol)	Frequency (cm^{-1})	Intensity (km/mol)
73.6817	0.4857	989.3288	9.6175
79.964	0.831	1018.293	35.5194
96.0339	0	1034.7911	53.5923
136.4557	1.2629	1040.6638	0
225.049	0.0471	1046.9479	4.9191
244.5375	0	1164.9467	2.7279
264.1077	0.7918	1208.5888	6.3315
284.0948	0.0088	1223.5949	57.4458
351.8945	0.0289	1269.5681	72.8069
354.772	1.6068	1313.5837	0.2861
380.1504	0	1334.0692	3.9747
466.1412	0.0247	1341.561	15.0795
558.7343	5.5695	1473.0099	30.0726
583.952	0	1496.3485	9.5051
611.1693	7.0105	1571.1062	33.4636
614.8274	9.5591	1585.8562	0.0746
667.6903	98.1408	1605.9554	16.9737
678.9946	87.8008	1638.3939	0.1519
680.5359	0	3080.3644	13.784
681.5822	0.5853	3081.6628	0.043
755.3479	11.8851	3114.6889	8.2749
764.4881	0	3115.7282	3.359
849.976	1.6326	3120.5763	1.5755
861.2475	0.1093	3132.2866	16.9101
865.503	7.8316	3171.0385	0.2418
890.788	3.4242	3185.9008	0.0133
907.2973	3.1068	3244.5729	38.1098
914.149	0	3244.7397	4.3348
939.9819	0		

Table S105. Cartesian coordinates for the optimized geometry of isomer 5v-singlet of $Co^{+}(C_2H_2)_5$.

Z	x	y	z
6	1.972073000	-0.826388000	-0.005246000
6	1.972066000	0.826399000	-0.005212000
6	1.213894000	0.676003000	1.300679000
6	1.213905000	-0.676051000	1.300654000
1	2.978250000	-1.235884000	0.072466000
1	2.978239000	1.235901000	0.072519000
1	0.873091000	1.430416000	2.001958000
1	0.873112000	-1.430495000	2.001905000
27	-0.722003000	-0.000010000	0.313302000
6	1.103121000	1.531826000	-0.981148000
1	1.534362000	2.205354000	-1.718176000
6	-0.213328000	1.329322000	-0.914116000
1	-0.947217000	1.808772000	-1.549871000
6	-2.664714000	0.000008000	-0.526854000
6	-2.739475000	-0.000018000	0.690838000
1	-3.026442000	-0.000084000	1.723684000
1	-2.840627000	0.000113000	-1.580766000
1	-0.947205000	-1.808718000	-1.549940000
6	-0.213319000	-1.329290000	-0.914166000
6	1.103132000	-1.531784000	-0.981207000
1	1.534377000	-2.205279000	-1.718264000

Table S106. Predicted frequencies (cm^{-1}) and IR intensities (km/mol) for isomer 5v-singlet of $Co^+(C_2H_2)_5$.

Frequency (cm^{-1})	Intensity (km/mol)	Frequency (cm^{-1})	Intensity (km/mol)
89.0053	0.5419	955.3711	1.5903
132.0566	0.1618	963.2265	0.3207
159.5905	0.4275	1013.5637	2.1312
178.0122	6.5954	1023.585	8.4963
213.7929	7.3532	1098.0498	0.7081
215.0185	1.741	1106.4742	7.0892
250.7434	0.0342	1125.5717	1.0421
254.0418	0.041	1136.1152	21.4925
332.4578	1.9966	1195.6424	7.8307
368.8317	1.4027	1255.0666	40.934
377.488	9.7142	1282.8354	14.8576
405.4003	0.9508	1297.1103	0.1708
470.0099	4.6274	1311.2382	2.3166
534.6044	0.6688	1336.7229	1.3126
577.3811	0.9488	1571.0318	2.1339
639.8165	9.3382	1581.006	1.4044
647.2157	10.7854	1586.0598	4.1672
684.6623	12.5378	1937.4304	9.1618
702.4788	0.0001	3103.7135	1.6251
733.6337	13.7038	3109.1972	5.0348
747.1126	52.391	3133.9221	1.4582
768.6187	28.9399	3134.8033	1.7505
774.1051	58.9634	3160.8044	0.2247
783.7118	2.4189	3186.2652	1.5763
850.9048	22.6448	3186.8084	0.1113
897.0243	0.8345	3187.2785	0.5952
897.5886	1.0111	3326.8152	152.9834
913.266	1.5027	3420.5277	65.3123
920.5789	6.6976		

Table S107. Cartesian coordinates for the optimized geometry of isomer 5w-singlet of $Co^{+}(C_2H_2)_5$.

Z	x	y	z
6	2.856169000	-0.348904000	-0.324807000
6	1.912778000	-1.116229000	-1.008810000
6	0.536518000	-1.029792000	-0.940828000
6	2.688177000	0.836379000	0.387020000
6	1.546922000	1.623767000	0.559320000
6	0.223786000	1.336462000	0.292480000
1	1.742222000	2.631389000	0.921382000
1	3.884967000	-0.674289000	-0.432781000
1	2.319382000	-1.868282000	-1.681194000
1	-0.013575000	-1.820279000	-1.467980000
1	3.607103000	1.276606000	0.757874000
1	-0.489640000	2.141765000	0.485110000
27	-0.745558000	-0.102334000	-0.100958000
6	-0.229899000	-1.181207000	1.468100000
6	-1.458611000	-1.011470000	1.550037000
1	-2.426737000	-1.131150000	1.991918000
1	0.734170000	-1.537349000	1.770556000
1	-2.036564000	1.023892000	-2.304988000
6	-2.370420000	0.746041000	-1.328904000
6	-2.960167000	0.527999000	-0.290083000
1	-3.662790000	0.422454000	0.504815000

Table S108. Predicted frequencies (cm^{-1}) and IR intensities (km/mol) for isomer 5w-singlet of $Co^{+}(C_2H_2)_5$.

Frequency (cm^{-1})	Intensity (km/mol)	Frequency (cm^{-1})	Intensity (km/mol)
46.2196	0.508	858.296	0.9353
81.8348	0.8462	884.4848	18.5215
93.173	0.2993	930.4333	8.1016
107.7778	1.1255	967.9905	0.3997
140.6584	1.2689	1014.704	1.503
150.0413	0.9536	1050.4512	1.6767
173.3876	5.0831	1072.9435	2.0864
194.7114	12.2344	1184.2355	1.4982
265.6585	4.8197	1237.553	5.2091
270.0545	4.8417	1296.4949	1.6159
305.8836	4.8176	1305.2775	47.2402
348.6249	13.5246	1336.651	3.2525
361.9503	1.3539	1483.3496	13.0616
391.9876	22.8476	1486.2657	15.7783
486.2154	4.3503	1582.0038	23.1001
558.3564	0.3354	1588.2762	2.7382
577.3932	5.1797	1821.4581	15.6704
584.7001	0.581	1970.5701	11.1427
641.9305	24.84	3023.8674	2.4812
653.3097	0.3426	3074.3528	3.1725
681.7314	13.6751	3121.7661	0.0635
696.9398	20.0719	3128.3735	0.0274
705.6062	43.2006	3163.7783	0.0151
739.4728	1.4514	3178.6815	0.0339
746.9283	19.1078	3310.126	51.2033
761.2205	66.3378	3363.8442	107.1302
772.6094	24.2937	3375.356	47.7684
814.6695	39.3287	3453.3285	76.4565
851.4405	6.9486		

Table S109. Cartesian coordinates for the optimized geometry of isomer 5x-singlet of $Co^{+}(C_2H_2)_5$.

Z	x	y	z
6	-2.348936000	-0.590559000	0.712476000
6	-2.348946000	-0.590565000	-0.712456000
6	-1.195965000	-0.120399000	1.254676000
1	-3.202970000	-0.896351000	-1.308260000
1	-1.061668000	0.147427000	2.298755000
6	-1.195984000	-0.120404000	-1.254674000
1	-1.061700000	0.147411000	-2.298757000
1	-3.202954000	-0.896336000	1.308294000
27	0.251050000	0.306232000	-0.000013000
6	0.895407000	-1.500921000	-0.724058000
6	0.895395000	-1.500926000	0.724053000
1	0.451191000	-2.131775000	1.473779000
1	0.451225000	-2.131771000	-1.473796000
1	2.657879000	-0.148219000	-1.472522000
6	2.027600000	-0.579338000	-0.711053000
1	2.657849000	-0.148229000	1.472560000
6	2.027587000	-0.579344000	0.711075000
6	0.839342000	2.197135000	0.000009000
6	-0.405525000	2.157454000	0.000000000
1	1.810924000	2.648775000	0.000020000
1	-1.417986000	2.508006000	-0.000004000

Table S110. Predicted frequencies (cm^{-1}) and IR intensities (km/mol) for isomer 5x-singlet of $Co^{+}(C_2H_2)_5$.

Frequency (cm^{-1})	Intensity (km/mol)	Frequency (cm^{-1})	Intensity (km/mol)
55.5431	1.371	939.3844	0.9566
76.796	2.5436	949.2123	5.2912
111.3026	2.6808	954.3997	9.1134
132.3663	0.0819	959.5615	2.1535
136.3243	2.5309	979.3902	12.0906
171.9707	0.4374	996.5193	5.1355
281.6971	7.1524	1077.3992	3.927
311.3937	1.6811	1087.5126	1.7524
337.0753	6.3356	1155.618	80.1421
368.5128	23.7654	1210.1083	0.0097
392.2088	12.2018	1223.1241	22.0145
444.1607	2.543	1242.5925	49.8373
453.9035	36.7195	1280.9669	2.6365
456.1016	2.008	1341.6724	24.3991
531.5496	11.0748	1389.286	5.291
561.7451	3.1409	1463.5372	65.1916
562.4771	22.8278	1534.4893	11.2827
686.9025	74.8008	1804.1168	7.8428
738.0806	3.0286	3143.7446	0.4123
757.5737	42.5258	3149.3732	2.7632
770.4534	11.8315	3165.091	0.1849
772.4371	2.1577	3174.8073	1.0387
781.8396	0.3638	3240.4336	2.7901
810.2163	36.0015	3258.9261	15.3792
828.4053	6.5114	3270.0945	10.6157
846.91	0.0992	3283.9459	9.486
857.345	23.5208	3313.1355	64.9207
880.0268	183.4025	3378.2353	44.0325
882.5606	0.0786		

Table S111. Cartesian coordinates for the optimized geometry of isomer 5a-triplet of $Co^{+}(C_2H_2)_5$.

Z	x	y	z
1	2.134794000	-1.701065000	1.794338000
6	1.670600000	-1.185170000	0.963634000
6	2.368798000	-0.346064000	0.190295000
6	1.713827000	0.317795000	-0.941038000
6	0.522897000	-0.158269000	-1.427042000
6	-0.177131000	-1.333255000	-0.752903000
6	0.177259000	-1.333254000	0.752879000
6	-0.522844000	-0.158321000	1.427037000
6	-1.713823000	0.317654000	0.941062000
6	-2.368743000	-0.346223000	-0.190290000
6	-1.670483000	-1.185258000	-0.963653000
1	3.419681000	-0.155545000	0.362900000
1	2.273547000	1.044804000	-1.521302000
1	0.165947000	0.161467000	-2.400337000
1	-2.134648000	-1.701167000	-1.794365000
1	-3.419639000	-0.155775000	-0.362898000
1	-2.273598000	1.044601000	1.521352000
1	-0.165913000	0.161417000	2.400337000
1	0.184481000	-2.256919000	-1.217154000
1	-0.184301000	-2.256945000	1.217116000
27	-0.000092000	1.417679000	0.000005000

Table S112. Predicted frequencies (cm^{-1}) and IR intensities (km/mol) for isomer 5a-triplet of $Co^{+}(C_2H_2)_5$.

Frequency (cm^{-1})	Intensity (km/mol)	Frequency (cm^{-1})	Intensity (km/mol)
67.7969	0.6822	1037.4944	7.4917
150.2263	2.2899	1081.9964	2.2595
186.305	1.2835	1175.3787	0.0485
228.479	0.1358	1190.9703	0.9881
264.7944	0.1374	1195.3583	0.2667
295.6039	1.6619	1197.5874	1.4904
327.129	13.3429	1270.594	0.0222
415.4909	0.8718	1274.4289	2.763
453.8178	6.7965	1319.8005	0.0013
518.1036	2.3238	1325.3341	7.9059
541.532	0.0875	1383.9541	0.7771
592.0362	5.9459	1385.4756	5.9106
602.1375	2.2481	1423.4704	2.613
663.8034	5.1683	1439.3164	9.6949
740.2672	35.3486	1534.7261	1.6378
749.0474	39.2589	1549.1051	5.987
782.9151	18.788	1662.9855	10.0956
815.546	0.2616	1669.0552	2.4767
838.6622	0.0404	3026.9668	1.762
915.0689	2.1259	3033.9405	1.9685
926.3482	1.3756	3149.0552	3.1737
949.1401	2.9726	3149.3053	0.6726
959.1077	42.8207	3165.6452	0.2781
971.2498	6.1693	3166.1302	0.06
990.6822	0.6679	3187.585	0.175
1005.0211	0.4222	3187.6793	0.3072
1009.6736	0.374	3204.9146	0.0677
1012.233	2.1527	3204.9384	0.0687
1022.5781	1.4787		

Table S113. Cartesian coordinates for the optimized geometry of isomer 5b-triplet of $Co^{+}(C_2H_2)_5$.

Z	x	y	z
27	0.211833000	-0.000001000	-0.193268000
6	1.610340000	1.262618000	0.005130000
6	2.856843000	0.719614000	0.027054000
1	3.770282000	1.306285000	0.054683000
1	1.426550000	2.332233000	0.061128000
6	2.856844000	-0.719614000	0.027054000
6	1.610342000	-1.262618000	0.005130000
1	1.426553000	-2.332234000	0.061132000
1	3.770283000	-1.306284000	0.054682000
6	-1.754727000	1.214221000	-0.586129000
6	-1.849631000	0.000013000	-1.282211000
6	-1.569019000	1.212226000	0.805553000
1	-1.979042000	0.000023000	-2.356061000
1	-1.478956000	2.148774000	1.338705000
6	-1.754733000	-1.214208000	-0.586151000
6	-1.476500000	-0.000012000	1.502352000
1	-1.813263000	-2.151228000	-1.122915000
1	-1.314088000	-0.000022000	2.571221000
6	-1.569024000	-1.212238000	0.805531000
1	-1.478963000	-2.148796000	1.338667000
1	-1.813252000	2.151251000	-1.122877000

Table S114. Predicted frequencies (cm^{-1}) and IR intensities (km/mol) for isomer 5b-triplet of $Co^+(C_2H_2)_5$.

Frequency (cm^{-1})	Intensity (km/mol)	Frequency (cm^{-1})	Intensity (km/mol)
16.5199	0.0021	1045.0687	0.1714
64.6379	1.359	1048.7051	0.4016
116.1911	0.5247	1052.3229	1.775
154.7975	3.4463	1099.1211	0.352
183.9628	1.4754	1099.5869	2.8687
201.5066	1.725	1191.8702	0
217.2654	2.711	1198.3884	0.8352
392.7927	1.2109	1199.1108	0.0669
396.8252	0.0212	1247.3912	26.2754
403.6408	3.0381	1267.9384	213.0318
413.17	0.2086	1343.2584	0.189
464.2353	0.1149	1388.7023	0.0005
584.1498	27.3819	1471.9971	91.8572
615.6903	0.2351	1503.6593	24.9224
615.7578	0.0188	1506.4421	22.3014
646.3045	82.4684	1526.1863	13.5967
688.3089	0.9477	1591.4483	0.8012
756.7404	0.0324	1595.691	0.206
774.2319	59.8497	3136.6978	0.0437
802.0166	7.686	3142.0317	2.6626
922.0363	0.1262	3159.668	0.7973
931.0151	1.6188	3169.7055	0.9878
935.6788	2.9857	3198.0541	0.1281
985.6936	65.4943	3204.0469	0.0172
993.2364	0.649	3204.7001	0.1815
1000.4491	0.8142	3213.6749	5.9268
1016.7409	0.8642	3214.2471	7.0849
1023.8312	0.2028	3220.2525	0.5105
1026.9732	0.1495		

Table S115. Cartesian coordinates for the optimized geometry of isomer 5c-triplet of $Co^{+}(C_2H_2)_5$.

Z	x	y	z
6	-1.650571000	-0.703461000	1.210743000
6	-1.682423000	1.404682000	0.000438000
1	-1.643684000	-1.242748000	2.148274000
1	-1.695863000	2.486112000	0.000774000
6	-1.682665000	-1.404604000	-0.000439000
6	-1.650413000	0.703534000	-1.210745000
1	-1.696284000	-2.486031000	-0.000773000
1	-1.643390000	1.242820000	-2.148275000
6	-1.650553000	-0.702709000	-1.211181000
1	-1.643640000	-1.241409000	-2.149050000
1	-1.643514000	1.241479000	2.149053000
27	0.321166000	0.000017000	-0.000036000
6	2.167310000	-0.762754000	-0.693653000
6	2.167258000	-0.762664000	0.693812000
1	2.270007000	-1.522414000	1.452182000
1	2.270127000	-1.522598000	-1.451919000
1	2.270350000	1.522259000	-1.452108000
6	2.167474000	0.762524000	-0.693739000
1	2.270248000	1.522440000	1.451992000
6	2.167422000	0.762612000	0.693712000

Table S116. Predicted frequencies (cm^{-1}) and IR intensities (km/mol) for isomer 5c-triplet of $Co^+(C_2H_2)_5$.

Frequency (cm^{-1})	Intensity (km/mol)	Frequency (cm^{-1})	Intensity (km/mol)
26.7759	0	1025.5771	0.0016
63.8743	0.47	1041.3302	0.1886
75.244	0.0996	1045.5408	1.315
83.3916	0.3051	1055.2087	1.1732
91.2076	0.625	1128.3417	8.4763
167.1781	3.7279	1185.7838	0.0391
195.1204	2.8949	1195.3883	0
333.3347	1.0194	1199.0817	0.0064
367.1982	2.073	1199.8563	0
392.6257	0	1278.84	25.1979
407.24	0.1295	1317.2829	0.0332
594.7166	0	1373.9229	31.7836
612.7618	0	1389.3872	0
615.8891	0.0286	1460.7966	0.0228
678.6413	0.0716	1502.8735	23.1777
688.9412	0.0143	1508.0803	23.7107
752.9309	146.7573	1594.3429	0.0162
764.8329	7.8825	1602.1406	0
783.9301	11.8259	3195.2787	0.0066
813.8922	0	3201.3706	0
848.8886	0.4581	3201.8632	0.0104
893.6808	0	3211.2321	4.4576
911.5947	42.4213	3212.0939	4.4581
915.4576	0.45	3217.7198	0.0063
923.2168	0.1602	3222.6408	0
999.2725	2.5243	3240.4074	4.8337
1012.6353	0	3250.5154	3.6576
1017.2757	0.01	3261.1547	4.7172
1021.5856	8.2039		

Table S117. Cartesian coordinates for the optimized geometry of isomer 5d-triplet of $Co^{+}(C_2H_2)_5$.

Z	x	y	z
6	1.733899000	0.021545000	-0.670787000
6	2.021516000	-0.662706000	0.612719000
6	1.155837000	-1.460979000	1.225035000
6	-0.113665000	-1.933521000	0.646696000
6	-1.357787000	-1.407434000	0.416063000
6	-1.978683000	-0.073444000	0.394723000
6	-1.584757000	1.220647000	0.596134000
6	-0.399542000	1.899717000	1.138302000
6	0.781854000	1.986948000	0.539504000
6	1.138797000	1.264040000	-0.705094000
1	1.186195000	1.837913000	-1.630839000
1	1.561816000	2.607563000	0.971725000
1	-0.589405000	2.496058000	2.026733000
1	-2.392210000	1.926861000	0.406340000
1	-3.006029000	-0.149332000	0.053758000
1	-2.070591000	-2.153582000	0.079501000
1	-0.089633000	-3.008649000	0.472353000
1	1.442783000	-1.951951000	2.151057000
1	2.991688000	-0.466088000	1.059966000
1	2.233060000	-0.342716000	-1.568740000
27	-0.357499000	-0.219443000	-1.080801000

Table S118. Predicted frequencies (cm^{-1}) and IR intensities (km/mol) for isomer 5d-triplet of $Co^+(C_2H_2)_5$.

Frequency (cm^{-1})	Intensity (km/mol)	Frequency (cm^{-1})	Intensity (km/mol)
12.4806	0.25	1036.389	0.4559
110.9741	0.0099	1041.1956	7.677
144.9351	0.1939	1045.5541	4.1605
172.8293	0.174	1187.6321	0.1807
208.1325	0.4077	1218.6747	4.5314
235.0454	1.2969	1224.7688	1.982
301.4433	2.1669	1317.9472	1.9712
342.4793	1.0208	1360.9345	0.0262
348.3679	4.1472	1367.6479	2.3118
404.952	1.6589	1399.8688	5.29
407.9295	4.5183	1424.1111	0.5233
515.4984	19.1078	1463.127	0.0175
535.0889	0.2417	1478.5944	0.4807
622.7061	18.4577	1526.3757	0.5017
671.3415	31.6992	1583.8732	6.6067
722.6636	18.6627	1608.6688	12.1944
725.7623	4.7695	1713.4828	0.7284
783.2695	29.5873	1716.4523	0.5851
794.9641	14.8951	3093.6951	1.5391
797.5222	30.3753	3103.8919	1.0032
836.2074	1.0257	3105.7488	0.4134
867.2689	10.0906	3109.3817	0.75
911.9101	1.2971	3136.5028	0.3311
934.8876	0.0085	3137.5669	0.3776
951.0007	1.5681	3140.7007	0.1307
990.5232	3.8755	3153.5181	0.5843
994.044	1.5561	3155.2498	0.3593
998.3987	9.2314	3157.2115	0.2729
1032.8959	5.1604		

Table S119. Cartesian coordinates for the optimized geometry of isomer 5e-triplet of $Co^{+}(C_2H_2)_5$.

Z	x	y	z
6	-2.717342000	-0.453993000	0.521343000
6	-2.554944000	-0.325986000	-0.856214000
6	-1.935431000	0.297202000	1.391388000
1	-3.176119000	-0.903064000	-1.529416000
1	-2.069053000	0.203869000	2.461055000
6	-1.617353000	0.563250000	-1.372479000
6	-1.004768000	1.207667000	0.885958000
1	-1.518710000	0.690866000	-2.442742000
1	-0.473057000	1.861224000	1.566312000
6	-0.850678000	1.353373000	-0.504574000
1	-0.246815000	2.154814000	-0.909979000
1	-3.460938000	-1.133768000	0.916395000
27	0.974952000	-0.110882000	-0.031756000
6	0.354592000	-2.082096000	-0.327551000
6	1.267134000	-2.099421000	0.490868000
1	2.019230000	-2.388025000	1.195691000
1	-0.439486000	-2.364680000	-0.985216000
1	2.097501000	2.453658000	0.512851000
6	2.323859000	1.466744000	0.165791000
1	3.698779000	-0.156361000	-0.648021000
6	2.942426000	0.502476000	-0.274450000

Table S120. Predicted frequencies (cm^{-1}) and IR intensities (km/mol) for isomer 5e-triplet of $Co^+(C_2H_2)_5$.

Frequency (cm^{-1})	Intensity (km/mol)	Frequency (cm^{-1})	Intensity (km/mol)
29.8098	0.9654	988.9351	1.9804
49.7816	0.383	1001.0867	7.7159
70.2296	0.4255	1013.4344	0.3091
91.137	0.5797	1029.0097	0.2605
101.2277	0.5815	1041.7192	0.0634
109.3198	0.028	1053.353	3.399
117.1328	0.0656	1062.6984	1.4842
143.899	1.0464	1185.3734	0.376
185.6236	10.1411	1202.4418	0.1396
281.8226	11.493	1205.5394	0.0694
290.5508	2.2834	1342.0203	0.7992
389.635	2.2147	1391.3201	0.0628
406.2379	1.9203	1510.7632	19.0274
410.6297	0.3226	1512.0766	10.3949
424.4902	0.9227	1615.4322	1.8021
616.7296	0.0639	1619.9744	1.1555
617.7525	0.1286	1902.8493	26.3134
676.1145	1.1309	1909.5181	6.8598
687.2565	0.8102	3176.4519	0.0777
698.4312	21.901	3184.1056	0.113
712.099	3.3437	3187.8455	0.5191
721.2143	39.4801	3196.5041	0.3231
730.599	96.004	3200.6254	0.109
744.3938	7.9205	3207.2863	0.0429
754.6775	74.866	3326.9987	107.9976
763.587	65.4997	3334.6539	106.731
786.5433	11.9665	3405.1774	62.7089
887.7184	0.0718	3415.1188	35.5175
903.3889	6.9458		

Table S121. Cartesian coordinates for the optimized geometry of isomer 5f-triplet of $Co^{+}(C_2H_2)_5$.

Z	x	y	z
6	0.516798000	1.547253000	-0.679652000
6	1.460862000	0.823383000	-1.550226000
6	1.634434000	-0.499446000	-1.554866000
6	1.634703000	-0.499850000	1.554512000
6	0.909516000	-1.445994000	0.685492000
6	0.909430000	-1.445834000	-0.685976000
1	0.515058000	-2.333035000	1.176920000
1	-0.118386000	2.273259000	-1.179867000
1	1.983042000	1.430132000	-2.282914000
1	2.294480000	-0.947666000	-2.290296000
1	2.294939000	-0.948280000	2.289645000
1	0.514916000	-2.332749000	-1.177579000
27	-0.870099000	-0.287480000	0.000110000
1	-0.118249000	2.272932000	1.180553000
6	0.516866000	1.547055000	0.680067000
6	1.461102000	0.822971000	1.550291000
1	1.983434000	1.429535000	2.283024000
6	-2.721625000	0.726675000	0.000011000
6	-2.927179000	-0.480950000	-0.000054000
1	-3.396514000	-1.444417000	0.000008000
1	-2.829483000	1.790672000	-0.000046000

Table S122. Predicted frequencies (cm^{-1}) and IR intensities (km/mol) for isomer 5f-triplet of $Co^+(C_2H_2)_5$.

Frequency (cm^{-1})	Intensity (km/mol)	Frequency (cm^{-1})	Intensity (km/mol)
53.7338	0.283	990.2448	0.0067
84.9015	0.0133	1006.8116	2.2881
111.8481	0.3853	1028.7619	11.9314
128.2244	0.0892	1029.815	4.5044
144.1246	2.388	1036.8252	0.3464
178.2106	0.4178	1217.2596	0.2412
237.1835	0.2797	1229.3194	0.0243
242.4861	0.0045	1234.3569	3.601
285.1087	0.7331	1246.912	1.5153
305.9745	19.5633	1381.1734	0
351.3501	2.6069	1415.5078	12.3553
357.2189	22.6084	1434.5805	0.6091
389.8278	2.1238	1462.4403	0.0498
413.244	0.3284	1575.9463	1.6305
635.8951	16.4352	1613.3957	0.7872
664.5542	0.3811	1685.6249	0.3703
667.0926	0.2555	1696.2975	0.903
686.5079	15.6888	1914.1189	31.4821
714.8902	18.2227	3114.5991	1.2515
732.6205	48.0445	3130.4319	0.2854
737.9906	68.4419	3130.5142	1.0489
762.0139	1.0716	3145.1789	1.6375
801.1531	7.5274	3154.6696	0.0003
811.135	46.8796	3155.7667	0.0738
835.1639	8.8071	3170.7698	0.3892
909.6884	0.7268	3171.2119	0.4481
924.3273	0.4061	3323.2383	120.6621
963.9934	4.8799	3406.8579	68.1783
969.481	0.4391		

Table S123. Cartesian coordinates for the optimized geometry of isomer 5g-triplet of $Co^{+}(C_2H_2)_5$.

Z	x	y	z
6	1.529818000	0.668724000	1.568277000
6	0.762309000	1.556813000	0.678979000
6	0.762333000	1.556756000	-0.679064000
6	0.762318000	-1.556802000	-0.678960000
6	1.529915000	-0.668730000	-1.568206000
6	1.529922000	0.668617000	-1.568250000
1	2.073273000	-1.180516000	-2.356208000
1	2.073081000	1.180492000	2.356356000
1	0.264368000	2.386206000	1.173511000
1	0.264407000	2.386107000	-1.173680000
1	0.264383000	-2.386182000	-1.173520000
1	2.073285000	1.180344000	-2.356287000
27	-0.905936000	0.000005000	-0.000053000
1	2.073072000	-1.180348000	2.356435000
6	1.529813000	-0.668628000	1.568322000
6	0.762297000	-1.556771000	0.679084000
1	0.264349000	-2.386126000	1.173671000
6	-2.794245000	0.620288000	-0.000029000
6	-2.794240000	-0.620286000	0.000040000
1	-3.185689000	-1.619022000	-0.000075000
1	-3.185700000	1.619023000	0.000087000

Table S124. Predicted frequencies (cm^{-1}) and IR intensities (km/mol) for isomer 5g-triplet of $Co^{+}(C_2H_2)_5$.

Frequency (cm^{-1})	Intensity (km/mol)	Frequency (cm^{-1})	Intensity (km/mol)
9.1533	0.3449	999.6372	0
52.7747	0.0019	1013.4656	3.6998
60.8112	0.3038	1035.295	0.0002
103.3971	0	1035.3129	11.667
117.2553	1.0999	1037.9537	0
183.8005	0.0668	1236.229	1.132
228.4581	2.5987	1244.7041	0.0078
232.3354	0	1255.9094	3.8312
261.4854	0	1261.3166	0.4582
317.0379	25.2574	1392.8807	0
344.634	1.1423	1425.7518	11.1079
397.0894	7.5535	1442.3489	0.7361
402.4858	0.4838	1469.1708	0
466.5937	0.0003	1616.0651	0.2723
646.1856	18.3818	1631.3376	1.1419
659.3529	0	1676.248	2.7402
685.6454	10.1784	1691.9542	1.5899
699.1423	0	1838.1724	39.2322
725.6504	57.5999	3134.816	0
731.4016	62.1944	3135.0305	2.3
756.1237	10.6253	3146.5629	0.4669
764.4653	45.0295	3149.5218	0.0052
795.3134	8.638	3151.4512	0
809.214	46.0201	3155.1068	0.2673
841.2288	5.8867	3166.8785	1.3612
910.4373	3.7217	3167.8391	0.5327
935.5617	0	3298.3552	91.0915
958.6516	0.7442	3367.7044	62.6491
963.9586	4.9295		

Table S125. Cartesian coordinates for the optimized geometry of isomer 5h-triplet of $Co^{+}(C_2H_2)_5$.

Z	x	y	z
6	-0.766527000	1.649046000	0.734289000
6	-0.766549000	1.649052000	-0.734279000
6	-1.269663000	0.616912000	1.446760000
1	-0.395191000	2.527272000	-1.246744000
1	-1.289892000	0.668008000	2.529428000
6	-1.269693000	0.616916000	-1.446744000
1	-1.289952000	0.668020000	-2.529411000
1	-0.395148000	2.527259000	1.246751000
27	0.875708000	0.152305000	-0.000023000
6	-1.781900000	-0.631762000	-0.795272000
6	-1.781895000	-0.631761000	0.795297000
1	-2.704161000	-0.991027000	1.253130000
1	-2.704170000	-0.991031000	-1.253097000
1	-0.144495000	-2.248738000	-1.423640000
6	-0.671060000	-1.669060000	-0.676082000
1	-0.144459000	-2.248709000	1.423655000
6	-0.671057000	-1.669061000	0.676098000
6	2.638705000	-0.853039000	-0.000013000
6	2.876517000	0.357742000	0.000030000
1	2.780755000	-1.914657000	-0.000027000
1	3.421315000	1.281460000	0.000085000

Table S126. Predicted frequencies (cm^{-1}) and IR intensities (km/mol) for isomer 5h-triplet of $Co^+(C_2H_2)_5$.

Frequency (cm^{-1})	Intensity (km/mol)	Frequency (cm^{-1})	Intensity (km/mol)
60.9941	0.1026	1003.8199	0.7188
74.7563	0.1095	1017.0924	0.2981
95.99	0.8066	1023.5335	0.9194
114.5437	0.1179	1050.0165	4.9988
128.2846	0.4455	1121.0791	2.3871
161.5002	0.5209	1165.7467	1.3891
193.1187	1.6586	1202.7731	1.9061
247.2631	4.9916	1221.605	3.2153
329.9631	26.2548	1223.9239	1.093
394.0039	1.0469	1293.9951	12.8906
411.2602	1.6501	1325.5629	3.6878
427.0929	0.8	1331.4608	3.2378
541.9305	0.0104	1404.061	9.3555
561.514	0.8292	1443.187	2.3106
595.5589	0.0145	1566.799	2.8387
678.0192	0.7214	1586.9782	3.4528
723.9578	51.6705	1653.9783	6.4382
747.5266	70.0968	1867.5627	46.7812
751.0046	36.1184	3080.4682	1.1231
762.2873	1.0516	3085.7491	4.9164
772.0464	39.0535	3172.6849	0.5679
811.0624	6.2295	3174.8987	0.5651
833.77	52.031	3184.5071	0.1385
849.8731	0.0028	3189.6638	0.0149
928.0583	1.5835	3197.7663	0.6768
943.1639	18.5744	3209.617	0.1478
961.7917	2.8613	3309.5993	104.3721
985.3197	0.5283	3384.5077	67.1863
994.8211	9.2738		

Table S127. Cartesian coordinates for the optimized geometry of isomer 5i-triplet of $Co^{+}(C_2H_2)_5$.

Z	x	y	z
6	-2.347603000	-1.168553000	0.395498000
6	-2.202945000	-0.343809000	-0.781595000
6	-1.309971000	0.637776000	-1.062184000
6	2.657080000	-0.618746000	-0.386479000
6	2.353569000	0.458808000	0.515173000
6	1.214735000	1.172036000	0.692976000
1	3.220226000	0.816446000	1.071817000
1	-3.371411000	-1.357971000	0.704434000
1	-3.001903000	-0.477283000	-1.514349000
1	-1.328103000	1.060136000	-2.072625000
1	3.694578000	-0.647680000	-0.705850000
1	1.258688000	1.956464000	1.480914000
27	-0.365548000	1.751167000	0.042427000
1	-1.620374000	-2.177567000	2.088194000
6	-1.352893000	-1.730885000	1.135954000
6	1.833592000	-1.573787000	-0.911407000
1	2.219154000	-2.155858000	-1.742398000
1	0.745377000	-1.669259000	1.586685000
6	0.025613000	-1.727171000	0.775829000
6	0.500230000	-1.840855000	-0.503258000
1	-0.174867000	-2.217821000	-1.265402000

Table S128. Predicted frequencies (cm^{-1}) and IR intensities (km/mol) for isomer 5i-triplet of $Co^{+}(C_2H_2)_5$.

Frequency (cm^{-1})	Intensity (km/mol)	Frequency (cm^{-1})	Intensity (km/mol)
57.4922	2.2508	1011.3888	2.513
100.0159	1.1436	1041.395	7.5466
119.188	0.9179	1099.2371	3.3404
133.8631	1.824	1163.487	15.5427
191.032	3.3056	1173.1664	8.4773
208.0666	4.431	1230.0573	3.1928
239.7095	0.9204	1249.6533	4.4022
286.9016	0.7004	1304.6777	39.4039
319.563	12.2095	1310.1275	16.8429
373.9734	2.0877	1349.4789	21.967
381.7858	23.1175	1355.8387	6.7678
436.2776	3.7985	1456.5261	26.6854
463.658	2.7385	1469.5258	45.6849
514.9031	12.9009	1490.8951	61.3062
549.7936	22.0026	1537.6389	25.3789
588.3305	29.5476	1559.6056	11.0938
642.03	1.0824	1592.5826	53.9022
675.7957	75.2918	1613.2766	1.5614
760.3998	7.3415	2874.838	9.1954
818.3277	17.7004	3044.0864	0.4384
826.9809	2.915	3080.2683	1.1311
842.4353	7.6516	3095.295	0.557
895.5076	29.6609	3143.1153	0.8203
896.8259	77.9282	3147.3589	0.3144
926.3282	32.2547	3150.3506	0.084
928.7911	26.2948	3156.179	2.0976
956.3209	14.3533	3165.1792	0.2403
961.5889	15.5635	3166.0656	0.3118
1004.3843	10.5551		

Table S129. Cartesian coordinates for the optimized geometry of isomer 5j-triplet of $Co^{+}(C_2H_2)_5$.

Z	x	y	z
6	0.622189000	1.295704000	1.042088000
6	1.868894000	0.759718000	0.305761000
6	1.155765000	1.337801000	-0.928590000
6	0.192918000	1.972441000	-0.135092000
1	0.428982000	1.467215000	2.090478000
1	2.805743000	1.256390000	0.557790000
1	1.452472000	1.450565000	-1.961107000
1	-0.564789000	2.718020000	-0.338841000
27	-0.624343000	0.000004000	-0.250819000
6	1.868887000	-0.759736000	0.305749000
1	2.805736000	-1.256419000	0.557759000
6	0.622184000	-1.295724000	1.042074000
6	1.155743000	-1.337792000	-0.928609000
1	1.452449000	-1.450554000	-1.961126000
1	0.428970000	-1.467241000	2.090462000
6	0.192897000	-1.972437000	-0.135113000
1	-0.564814000	-2.718011000	-0.338864000
6	-2.527434000	-0.000017000	0.782882000
6	-2.756253000	0.000028000	-0.415720000
1	-3.204572000	0.000075000	-1.387831000
1	-2.557662000	-0.000067000	1.850791000

Table S130. Predicted frequencies (cm^{-1}) and IR intensities (km/mol) for isomer 5j-triplet of $Co^{+}(C_2H_2)_5$.

Frequency (cm^{-1})	Intensity (km/mol)	Frequency (cm^{-1})	Intensity (km/mol)
53.7162	2.4762	963.1589	4.0012
69.7333	0.2426	970.6209	4.6927
80.5331	0.2929	1000.0801	0.0005
129.8618	2.6823	1001.0393	5.1565
167.1857	3.3057	1047.8013	1.5857
198.3063	0.8066	1118.7043	21.6255
220.7433	1.1892	1147.6718	9.8501
222.5083	16.5868	1161.3569	5.691
293.9176	3.5461	1181.5216	4.4947
313.4371	3.2071	1221.5562	0.0002
363.2315	6.9819	1223.1766	0.9402
439.7369	0.6889	1297.2432	0.4883
486.2422	0.0329	1334.1463	1.2424
498.5155	0.5317	1357.0638	13.7983
663.7156	3.9914	1377.2882	1.8238
673.0905	13.4511	1420.7019	6.239
733.5404	1.1056	1427.8599	1.5642
757.6099	65.3291	1933.7927	32.3735
758.8154	4.4158	3091.1193	1.3677
766.6624	13.2192	3100.9988	6.1238
795.3641	9.0807	3194.5396	0.2819
796.1894	5.4767	3196.0112	0.2514
827.5172	5.7295	3225.4385	0.0337
848.0654	0.5964	3226.7798	2.8767
896.3902	10.7836	3229.8757	6.861
903.757	5.3985	3232.1423	1.4207
914.5731	2.2809	3339.0013	136.7502
928.0936	0.0035	3425.2521	75.4275
956.8534	23.4872		

Table S131. Cartesian coordinates for the optimized geometry of isomer 5k-triplet of $Co^{+}(C_2H_2)_5$.

Z	x	y	z
6	1.860118000	-0.719675000	-1.021010000
6	2.388969000	0.310106000	0.000085000
6	1.859843000	-0.719629000	1.021092000
6	1.516288000	-1.619409000	0.000006000
1	1.908927000	-0.772856000	-2.098859000
1	3.473171000	0.419447000	0.000222000
1	1.908472000	-0.772812000	2.098949000
1	1.149951000	-2.638678000	-0.000028000
27	-0.230617000	-0.128996000	-0.000028000
6	1.660155000	1.632058000	-0.000037000
6	0.323219000	1.629327000	-0.000056000
1	-0.324717000	2.493986000	-0.000119000
1	2.227567000	2.559856000	-0.000114000
1	-3.653224000	-0.387687000	1.300799000
6	-2.760729000	-0.181726000	0.717565000
6	-1.548875000	0.114235000	1.270358000
1	-1.372848000	0.351788000	2.314171000
1	-3.653294000	-0.387786000	-1.300677000
6	-2.760768000	-0.181775000	-0.717510000
6	-1.548948000	0.114161000	-1.270385000
1	-1.372970000	0.351604000	-2.314231000

Table S132. Predicted frequencies (cm^{-1}) and IR intensities (km/mol) for isomer 5k-triplet of $Co^{+}(C_2H_2)_5$.

Frequency (cm^{-1})	Intensity (km/mol)	Frequency (cm^{-1})	Intensity (km/mol)
58.1941	0.2353	986.975	22.0107
64.5105	1.2413	988.1618	1.0493
92.1324	0.9064	1001.6558	7.3327
122.7835	3.2485	1082.3139	1.7945
161.275	3.233	1087.1133	5.1554
219.63	1.4563	1094.1948	8.5505
231.1462	0.3103	1138.2358	9.9762
273.1362	0.0964	1170.999	11.0621
372.63	2.3909	1214.692	1.3211
390.3703	0.0589	1234.699	19.4844
401.5575	11.4872	1254.4792	64.7263
413.365	7.1037	1267.6215	6.396
526.8994	0.8852	1305.0494	0.2595
567.3495	4.0437	1344.3895	12.5492
611.4989	19.5208	1419.3177	4.6318
683.1376	73.9835	1443.636	27.4788
695.0273	18.2702	1516.6795	19.8321
710.016	12.9782	1554.8173	12.052
738.9811	22.1618	3099.1254	0.499
742.9637	5.7598	3135.8526	2.4562
756.772	1.5055	3149.1923	0.282
780.1925	1.434	3160.1182	0.0578
884.46	8.2578	3174.2601	2.5315
903.6939	9.4117	3177.9952	0.6829
912.2337	10.7011	3193.339	1.1788
934.2214	7.2023	3218.5929	3.6789
935.3522	21.8313	3229.3483	7.2226
962.7413	10.6471	3235.0252	5.5441
967.5753	6.1541		

Table S133. Cartesian coordinates for the optimized geometry of isomer 5l-triplet of $Co^{+}(C_2H_2)_5$.

Z	x	y	z
6	1.040593000	0.029066000	-1.036660000
6	2.210967000	0.713947000	-0.253511000
6	2.862139000	-0.654091000	-0.368994000
6	1.819810000	-1.205742000	-1.033733000
1	0.594343000	0.445572000	-1.942363000
1	2.742727000	1.498264000	-0.799469000
1	3.839363000	-1.017724000	-0.080212000
1	1.650292000	-2.180281000	-1.470460000
27	-0.653116000	0.014569000	0.126587000
6	1.740349000	1.176524000	1.088034000
1	2.444451000	1.650594000	1.767762000
6	0.459561000	0.980896000	1.386660000
1	0.000081000	1.300062000	2.321664000
6	-2.110654000	-1.729141000	0.522590000
6	-0.990676000	-2.066810000	0.838736000
1	-0.079139000	-2.511183000	1.175466000
1	-3.150224000	-1.580014000	0.334866000
1	-3.048110000	0.146883000	-1.760992000
6	-2.551700000	0.854247000	-1.135313000
6	-2.082908000	1.768068000	-0.500557000
1	-1.744522000	2.652675000	-0.007640000

Table S134. Predicted frequencies (cm^{-1}) and IR intensities (km/mol) for isomer 5l-triplet of $Co^+(C_2H_2)_5$.

Frequency (cm^{-1})	Intensity (km/mol)
42.2443	0.0075
52.4066	0.3067
69.238	0.2606
77.703	0.3886
100.568	2.632
130.413	1.9169
136.3904	0.8261
170.991	1.7465
192.3213	2.7913
218.1532	0.8268
241.668	0.3341
250.9547	4.5563
283.4606	1.0222
305.3438	1.9577
385.7308	11.4167
464.5597	4.7265
511.1866	45.8308
646.4626	0.6196
653.4544	0.5728
671.8244	0.5525
696.7309	6.3334
697.372	50.0623
707.7716	13.9878
747.4306	26.5228
766.8283	37.6177
774.5126	51.0824
787.9763	26.2844
795.3478	44.2115
850.6336	76.0164
871.2496	98.4235
907.3862	5.6405
939.4752	62.1807
965.8402	9.2553
987.9491	6.2539
1010.1408	11.2764
1025.0515	9.795
1075.33	5.1987
1134.6598	4.6669
1182.1942	6.8564
1204.0356	1.285
1283.0443	29.0305
1309.7239	5.7148
1327.8576	23.8089
1529.8287	22.7471
1611.1085	10.29
1985.6459	2.7667
2011.3542	1.6006
3051.6937	3.435
3075.7985	5.6167
3103.8679	1.9955
3138.2363	1.6652
3197.3356	0.7118
3222.2662	0.7058
3361.6664	114.4398
3368.6	154.1911
3453.845	49.3679
3463.0332	37.0742

Table S135. Cartesian coordinates for the optimized geometry of isomer 5m-triplet of $Co^{+}(C_2H_2)_5$.

Z	x	y	z
6	-1.693229000	-1.410623000	0.000426000
6	-0.706829000	-1.685365000	-1.030891000
6	0.247858000	-2.030159000	-0.000305000
6	-0.706063000	-1.685394000	1.031000000
1	-2.758062000	-1.248334000	0.000823000
1	-0.744382000	-1.786584000	-2.102363000
1	1.248297000	-2.432573000	-0.000676000
1	-0.742820000	-1.786643000	2.102497000
27	-0.154319000	0.000000000	-0.000131000
1	-0.742804000	1.786647000	2.102497000
6	-0.706055000	1.685397000	1.031000000
6	-1.693228000	1.410624000	0.000434000
1	-2.758061000	1.248340000	0.000839000
6	-0.706833000	1.685364000	-1.030891000
1	-0.744394000	1.786580000	-2.102362000
6	0.247860000	2.030159000	-0.000311000
1	1.248299000	2.432572000	-0.000690000
6	3.168100000	-0.000003000	0.599830000
6	3.168136000	0.000000000	-0.599801000
1	3.221068000	-0.000006000	1.663474000
1	3.221176000	0.000001000	-1.663442000

Table S136. Predicted frequencies (cm^{-1}) and IR intensities (km/mol) for isomer 5m-triplet of $Co^{+}(C_2H_2)_5$.

Frequency (cm^{-1})	Intensity (km/mol)	Frequency (cm^{-1})	Intensity (km/mol)
4.8385	0.0016	935.6995	1.1936
47.0053	4.9685	947.0772	11.8322
64.1127	0	951.6376	27.2475
70.8911	0	952.5432	2.1923
76.2648	0.0376	955.4422	0
78.1997	1.316	965.7076	0.2436
119.3608	0.3849	969.3087	4.6578
121.9067	1.8203	1105.8099	0
312.4771	2.8716	1199.343	1.29
350.0363	0.8051	1206.0241	0
374.5053	0	1207.7004	0.1228
383.0038	16.8563	1259.3726	1.1552
386.1351	6.6644	1260.1576	6.6333
434.9433	23.1427	1342.0183	27.3981
570.5641	1.1559	1345.0971	0.7508
601.6632	6.9435	1363.8887	7.9227
640.1124	0	1367.5857	0
659.904	0.1616	2055.3854	26.5477
685.0463	0	3240.6353	2.3749
734.8663	0	3244.8194	0.5359
747.5945	10.3747	3256.6866	3.1836
777.9912	82.9622	3260.5883	9.9851
790.0974	154.1142	3263.4511	30.0594
794.7568	54.3558	3263.8806	0
804.0152	1.3477	3274.7035	17.5115
810.5202	4.7275	3277.704	2.6187
833.8266	2.4043	3395.0744	117.143
858.5626	7.6768	3494.3029	0.8973
859.4053	0.0018		

Table S137. Cartesian coordinates for the optimized geometry of isomer 5n-triplet of $Co^{+}(C_2H_2)_5$.

Z	x	y	z
6	-1.440982000	0.000000000	-0.763217000
6	-1.962143000	-1.193474000	0.000002000
6	-1.440982000	0.000000000	0.763221000
6	-1.962143000	1.193474000	0.000002000
1	-0.761501000	0.000000000	-1.615398000
1	-3.024703000	-1.410357000	0.000002000
1	-0.761498000	0.000000000	1.615399000
1	-3.024703000	1.410357000	0.000003000
27	0.692480000	0.000000000	-0.000005000
6	-0.961064000	-2.274519000	0.000000000
1	-1.293381000	-3.308996000	0.000000000
6	0.357281000	-1.952087000	-0.000003000
1	1.075726000	-2.762349000	-0.000003000
6	2.616240000	-0.618456000	0.000008000
6	2.616240000	0.618456000	0.000007000
1	2.999384000	1.617140000	0.000008000
1	2.999384000	-1.617141000	0.000011000
1	1.075727000	2.762348000	-0.000002000
6	0.357281000	1.952086000	-0.000002000
6	-0.961064000	2.274519000	0.000001000
1	-1.293380000	3.308996000	0.000001000

Table S138. Predicted frequencies (cm^{-1}) and IR intensities (km/mol) for isomer 5n-triplet of $Co^{+}(C_2H_2)_5$.

Frequency (cm^{-1})	Intensity (km/mol)	Frequency (cm^{-1})	Intensity (km/mol)
59.6622	2.1857	980.034	0
74.1706	0	994.9917	2.1593
143.6612	0.104	1015.0524	0
206.4072	6.3169	1023.7134	2.554
258.1797	1.3112	1026.5861	0.2337
261.416	0	1084.9467	0
282.3042	0.7472	1120.5435	17.3927
329.2454	0.3369	1159.1511	0.82
369.085	13.3167	1167.5224	4.1152
376.9403	0.0421	1183.2282	1.2465
389.134	11.0083	1232.201	10.0886
470.8948	0	1289.9477	1.5862
494.4468	9.8632	1293.6007	27.3105
503.4658	22.0418	1348.956	0.0013
545.3895	30.5776	1387.2189	8.4153
705.2734	0.4644	1514.3142	96.4376
711.1194	0	1526.6058	9.234
724.4775	48.1565	1858.238	1.0152
757.1402	60.2624	3090.0101	21.4464
762.6626	0.6168	3109.7263	1.3632
778.8072	0	3144.7593	0.1564
799.2068	2.5677	3145.0938	1.9202
825.323	0.096	3161.0322	0.1179
832.9478	0.7496	3161.3865	0.0057
846.3597	0	3178.3437	4.2783
856.4678	26.8568	3178.5959	8.7923
864.2966	1.1914	3329.6427	68.8111
911.2699	43.8796	3401.0968	35.7023
978.9946	0.8457		

Table S139. Cartesian coordinates for the optimized geometry of isomer 5o-triplet of $Co^{+}(C_2H_2)_5$.

Z	x	y	z
27	0.000007000	-0.716115000	0.000068000
6	-1.468824000	-2.191091000	-0.300050000
6	-2.044438000	-1.222762000	0.181633000
1	-2.783022000	-0.535278000	0.544677000
1	-1.250308000	-3.151935000	-0.715762000
6	0.574497000	1.318489000	0.211234000
6	-0.574561000	1.318511000	-0.210663000
1	-1.555827000	1.600288000	-0.537141000
1	1.555746000	1.600256000	0.537764000
6	1.468902000	-2.191098000	0.299854000
6	2.044476000	-1.222639000	-0.181616000
1	2.783027000	-0.535039000	-0.544514000
1	1.250424000	-3.152041000	0.715356000
6	-4.398025000	1.508918000	-0.477895000
6	-4.108197000	1.959512000	0.594374000
1	-3.889886000	2.385264000	1.545647000
1	-4.696961000	1.130048000	-1.427080000
6	4.398163000	1.508979000	0.477531000
6	4.107983000	1.959536000	-0.594658000
1	3.889361000	2.385256000	-1.545874000
1	4.697409000	1.130146000	1.426634000

Table S140. Predicted frequencies (cm^{-1}) and IR intensities (km/mol) for isomer 5o-triplet of $Co^+(C_2H_2)_5$.

Frequency (cm^{-1})	Intensity (km/mol)	Frequency (cm^{-1})	Intensity (km/mol)
17.7891	0.1697	730.5017	16.8335
23.5337	0.2573	754.8571	0.262
33.9124	0.2354	760.6459	5.1376
53.7766	0.0048	778.4006	61.1494
57.6423	0.0022	778.4409	84.9929
60.4748	0.0903	783.6193	108.9794
69.3706	1.2167	784.1762	116.9683
72.465	3.6815	789.0681	139.8166
95.2202	1.873	790.0407	169.8139
96.0731	0.0339	792.2187	47.355
114.9414	0.5423	804.0347	0.0418
132.7326	0.6797	820.8527	4.2782
133.0831	0.7167	836.8219	0.9091
142.6349	0.4237	1913.3093	3.4465
162.6318	3.0849	1914.2144	11.3399
173.2572	6.4287	1918.844	1.8903
265.2699	1.8975	2062.0613	14.2029
271.2862	0.7078	2062.285	4.1232
273.5557	4.2932	3297.1052	204.7508
399.4248	7.3705	3309.7169	337.1115
404.1475	2.7404	3310.1196	223.9793
407.1504	9.7029	3383.4808	86.6604
642.203	0.0013	3396.457	32.4913
642.2793	0.0093	3396.5414	201.6294
669.5443	0.2084	3401.3928	97.7039
669.7965	0.5523	3403.635	15.4584
693.406	0.9834	3496.1886	2.0405
698.2136	8.6951	3496.2342	0.5824
714.2016	0.0217		

Table S141. Cartesian coordinates for the optimized geometry of isomer 5p-triplet of $Co^{+}(C_2H_2)_5$.

Z	x	y	z
27	-1.168505000	0.036928000	0.123823000
6	0.309822000	-0.828771000	0.973650000
6	1.536575000	-0.442747000	0.559431000
1	2.465999000	-0.768713000	1.016710000
1	0.102336000	-1.527469000	1.780876000
6	1.494333000	0.442585000	-0.585784000
6	0.237355000	0.738365000	-0.973104000
1	-0.049155000	1.349196000	-1.825794000
1	2.388022000	0.823685000	-1.070625000
6	-3.059786000	1.445275000	-0.304014000
6	-2.333116000	1.963338000	0.511047000
1	-1.782497000	2.552956000	1.213113000
1	-3.762242000	1.094390000	-1.027059000
1	-0.991787000	-2.619194000	-0.906412000
6	-1.812452000	-2.066232000	-0.506104000
6	-2.829605000	-1.585996000	-0.057307000
1	-3.800456000	-1.325587000	0.301205000
6	5.415890000	-0.360185000	-0.418652000
6	5.391495000	0.563968000	0.342716000
1	5.404220000	1.385535000	1.018256000
1	5.472120000	-1.179454000	-1.094763000

Table S142. Predicted frequencies (cm^{-1}) and IR intensities (km/mol) for isomer 5p-triplet of $Co^{+}(C_2H_2)_5$.

Frequency (cm^{-1})	Intensity (km/mol)	Frequency (cm^{-1})	Intensity (km/mol)
9.8926	0.0112	772.8942	89.85
14.5961	0.0168	778.4458	155.5354
29.2284	0.004	779.7547	21.8165
43.2318	2.1629	794.6045	49.203
47.1861	0.0858	796.0301	13.8005
56.8746	0.543	805.1096	43.7995
67.0209	0.3258	922.6554	3.487
78.9883	0.1433	979.5708	27.1646
91.2828	1.7895	997.5007	1.0409
117.6864	0.2691	1063.2901	16.9564
147.3098	0.1699	1078.0985	2.1298
178.9114	2.4827	1218.2828	61.7164
190.1157	1.0897	1280.5124	141.1106
205.5352	3.1188	1490.8727	59.2996
261.2966	1.8248	1547.7943	2.0863
290.4958	3.4576	1990.0017	1.8617
357.1132	7.2238	2004.6343	4.0639
401.6689	2.7082	2067.6424	4.3124
439.088	1.1341	3131.7968	0.8768
542.1309	21.1572	3137.4697	0.3213
618.3887	83.3774	3160.8697	0.2587
629.5252	0.0062	3170.811	1.7627
641.4537	0.555	3355.7515	157.988
645.2492	2.4305	3367.661	144.7442
656.0844	6.3219	3407.6273	100.7652
689.2855	30.9459	3452.6886	44.0738
695.0862	8.2048	3458.7878	38.6399
745.6392	2.0294	3506.4408	1.0963
769.2163	83.1771		

Table S143. Cartesian coordinates for the optimized geometry of isomer 5q-triplet of $Co^{+}(C_2H_2)_5$.

Z	x	y	z
27	0.592968000	-0.058686000	-0.191111000
6	0.216938000	-2.226738000	-0.228523000
6	-0.789033000	-1.636766000	-0.584160000
1	-1.769670000	-1.343719000	-0.901713000
1	0.972473000	-2.936679000	0.026537000
6	2.960177000	-0.173286000	0.192268000
6	2.527172000	-0.762228000	1.151852000
1	2.260880000	-1.294439000	2.036562000
1	3.475454000	0.315130000	-0.603266000
6	0.640744000	0.852538000	-2.106063000
6	0.794603000	1.763817000	-1.306560000
1	0.926999000	2.711720000	-0.831239000
1	0.526443000	0.249749000	-2.982110000
6	0.324382000	1.363042000	1.938644000
6	-0.768999000	1.032827000	1.555823000
1	-1.784822000	0.810907000	1.302602000
1	1.254858000	1.695291000	2.337382000
6	-4.042961000	0.454459000	-0.317216000
6	-4.130862000	-0.441682000	0.473799000
1	-4.252094000	-1.234257000	1.174508000
1	-4.013615000	1.254927000	-1.018466000

Table S144. Predicted frequencies (cm^{-1}) and IR intensities (km/mol) for isomer 5q-triplet of $Co^{+}(C_2H_2)_5$.

Frequency (cm^{-1})	Intensity (km/mol)	Frequency (cm^{-1})	Intensity (km/mol)
18.8672	0.053	690.3033	3.0468
43.9911	0.5163	698.4908	14.3118
46.9227	0.0205	707.8871	38.3775
54.4924	2.2488	749.1448	15.6082
61.3125	0.5943	759.5395	117.7367
72.7317	0.5358	767.6383	116.0274
77.0426	0.2422	778.6388	68.3166
86.7373	0.5164	781.7196	58.8926
100.4558	0.8985	787.8725	18.262
111.5493	0.381	789.0655	121.4854
117.3098	0.1069	793.8125	13.8318
127.8363	0.4919	807.4541	54.7938
131.6644	0.2325	824.4402	55.0651
133.4954	1.0351	1929.0282	13.3193
163.5967	1.0771	1941.9026	6.6633
165.7175	0.4441	2017.1375	0.8329
202.9451	1.1806	2025.0061	6.0481
225.7633	0.6446	2062.8596	6.82
234.115	2.8973	3324.465	138.7621
260.3002	4.2622	3341.2673	87.9111
344.4962	2.8013	3342.4513	335.6757
359.2312	2.1186	3378.5984	130.3188
641.9438	0.0127	3397.6008	112.1705
662.9661	0.08	3423.6491	71.8137
665.4103	0.9487	3425.3782	19.3307
672.2967	2.3149	3463.5334	11.3445
677.8223	2.7633	3472.8497	18.9207
680.2278	2.3841	3497.2566	1.2178
684.1191	0.6369		

Table S145. Cartesian coordinates for the optimized geometry of isomer 5r-triplet of $Co^+(C_2H_2)_5$.

Z	x	y	z
27	-0.830236000	0.164929000	-0.093606000
6	-0.370102000	0.507072000	2.298455000
6	0.675749000	0.191624000	1.785237000
1	1.663178000	-0.047032000	1.449489000
1	-1.211498000	0.820913000	2.871922000
6	-0.329155000	1.906350000	-1.214729000
6	0.662448000	1.194127000	-1.256524000
1	1.641227000	0.782673000	-1.396240000
1	-1.043889000	2.695571000	-1.313341000
6	-2.771855000	-0.321505000	0.599859000
6	-2.838730000	-0.015592000	-0.586339000
1	-3.200625000	0.173899000	-1.575694000
1	-3.045150000	-0.650561000	1.579651000
6	4.050416000	0.587297000	0.179289000
6	3.935248000	-0.567182000	-0.121426000
1	3.880129000	-1.596302000	-0.387562000
1	4.194332000	1.607808000	0.447123000
6	0.050277000	-2.235417000	-0.081563000
6	0.151900000	-1.898665000	-1.232512000
1	0.259037000	-1.706146000	-2.275290000
1	-0.017544000	-2.622550000	0.908829000

Table S146. Predicted frequencies (cm^{-1}) and IR intensities (km/mol) for isomer 5r-triplet of $Co^{+}(C_2H_2)_5$.

Frequency (cm^{-1})	Intensity (km/mol)	Frequency (cm^{-1})	Intensity (km/mol)
31.7962	0.1285	701.7195	4.3475
38.9122	0.3881	714.1479	14.7271
56.9413	0.122	734.0894	34.1894
63.5628	1.0153	745.9472	37.879
67.6518	0.7806	761.1512	49.7798
72.3471	0.8422	772.6274	82.2844
82.1479	0.3804	779.2959	124.1368
94.7056	0.0195	780.8979	110.7163
100.7864	1.3589	787.6901	75.5425
110.0798	0.0374	790.127	10.5481
120.6762	0.0296	800.8596	107.6343
125.7857	0.3889	804.3894	12.3034
139.248	3.4346	824.2702	34.9809
147.8202	2.1801	1903.9161	23.3394
154.1472	0.3664	1930.1351	8.0546
165.6874	1.7445	2011.8304	4.4854
202.1133	1.9802	2035.0458	4.599
243.8683	1.0265	2062.9041	6.698
250.6	1.8635	3323.6987	138.6254
274.8743	8.0729	3331.5714	97.3652
362.2963	2.4839	3343.4093	292.3684
394.3094	1.9648	3386.4146	129.4345
643.0809	0.0511	3398.581	109.7865
646.0242	0.1599	3409.5912	73.9276
661.4008	3.1067	3412.3683	34.3617
666.9291	0.9653	3459.4472	21.8994
671.9919	0.6681	3483.1696	11.3372
682.818	4.4176	3498.2399	1.1856
683.9685	3.2704		

Table S147. Cartesian coordinates for the optimized geometry of isomer 5s-triplet of $Co^+(C_2H_2)_5$.

Z	x	y	z
27	-0.821258000	-0.000003000	0.000058000
6	-2.064910000	1.323907000	0.597082000
6	-3.187550000	0.643577000	0.378501000
1	-4.142309000	0.999621000	0.762167000
1	-1.892418000	2.243556000	1.134623000
6	-3.187516000	-0.643504000	-0.378728000
6	-2.064867000	-1.323864000	-0.597160000
1	-1.892320000	-2.243519000	-1.134671000
1	-4.142232000	-0.999526000	-0.762522000
6	1.427468000	-0.562394000	0.505847000
6	0.333667000	-1.231332000	0.857917000
1	0.174022000	-2.040580000	1.552656000
1	2.404769000	-0.788109000	0.933392000
1	2.404831000	0.787946000	-0.933206000
6	1.427506000	0.562270000	-0.505691000
6	0.333746000	1.231253000	-0.857795000
1	0.174144000	2.040506000	-1.552537000
6	5.063766000	-0.452385000	-0.392983000
6	5.063792000	0.452486000	0.392798000
1	5.107467000	1.255869000	1.089604000
1	5.107395000	-1.255768000	-1.089793000

Table S148. Predicted frequencies (cm^{-1}) and IR intensities (km/mol) for isomer 5s-triplet of $Co^{+}(C_2H_2)_5$.

Frequency (cm^{-1})	Intensity (km/mol)	Frequency (cm^{-1})	Intensity (km/mol)
17.5851	0.059	888.8388	3.3837
21.627	0.0802	893.8321	2.5526
37.6861	0.0052	896.9152	2.2166
59.34	1.8936	922.4364	2.4904
60.5835	10.6649	925.2214	0.2733
81.6429	3.5377	986.6152	52.5638
96.8996	0.9863	1011.1659	42.5376
113.5907	1.7116	1046.5554	23.7995
144.2299	53.977	1057.3931	0.0191
201.5834	0.3622	1204.5038	52.0575
204.9057	0.2712	1234.7249	0.0666
220.8889	1.8741	1234.7575	51.5886
342.3878	3.4169	1276.0307	16.0597
384.0918	0.1449	1482.6131	0.3337
443.9278	4.7334	1523.415	2.1647
486.1317	3.1105	1608.1677	0.7137
593.0251	4.6676	1612.2744	4.6971
605.6413	1.2766	2062.6896	21.6817
624.8464	85.1154	3094.3386	3.2375
634.9323	0.0004	3104.6164	162.0264
654.3712	15.5774	3114.892	2.2429
663.1851	18.3289	3126.9971	15.3793
731.092	25.3093	3238.4543	28.3244
758.3536	11.694	3238.4735	0.495
764.4347	2.5555	3248.7509	1.1257
770.1956	32.2656	3249.0147	38.1678
772.752	92.8527	3401.1323	111.0313
786.5737	156.9314	3500.3119	1.079
843.1569	0.5017		

Table S149. Cartesian coordinates for the optimized geometry of isomer 5t-triplet of $Co^{+}(C_2H_2)_5$.

Z	x	y	z
27	0.762906000	0.132355000	-0.054718000
6	-1.055865000	0.677928000	-0.076105000
6	-0.773981000	1.975154000	0.012492000
1	-1.558400000	2.701088000	0.223144000
1	-1.984189000	0.130239000	0.019968000
6	0.616892000	2.529003000	-0.150315000
6	1.709032000	1.776723000	-0.038330000
1	2.755479000	2.020303000	-0.135645000
1	0.685053000	3.591538000	-0.379090000
1	2.183467000	-0.388720000	2.223212000
6	1.856605000	-0.720700000	1.250132000
6	1.865454000	-1.926076000	0.686572000
1	2.255772000	-2.790874000	1.221051000
6	1.327275000	-2.165869000	-0.690805000
1	1.631879000	-3.087632000	-1.183971000
6	0.527028000	-1.293119000	-1.299024000
1	0.075348000	-1.315508000	-2.278629000
6	-4.586619000	-0.583212000	-0.328091000
6	-4.413517000	-0.778473000	0.841205000
1	-4.298564000	-0.962000000	1.882968000
1	-4.778133000	-0.420173000	-1.362007000

Table S150. Predicted frequencies (cm^{-1}) and IR intensities (km/mol) for isomer 5t-triplet of $Co^{+}(C_2H_2)_5$.

Frequency (cm^{-1})	Intensity (km/mol)	Frequency (cm^{-1})	Intensity (km/mol)
13.5965	0.149	878.4577	0.7757
22.9224	0.0106	893.0848	2.1886
30.4527	0.0333	900.2295	2.7044
54.6772	3.3412	918.5881	5.7521
63.6459	2.2575	920.7074	0.9346
85.2333	4.2818	994.1491	56.0619
86.0984	0.909	1017.7119	19.5103
120.5346	19.5096	1048.9949	24.4624
125.9522	38.9871	1058.7643	1.9233
193.0112	0.4399	1207.1929	50.9294
204.015	0.4853	1235.5768	38.2691
220.6039	1.1372	1240.0879	25.4718
342.6185	3.4338	1278.6976	0.261
379.3667	0.0493	1486.6263	4.6489
446.8996	4.2085	1516.1845	0.6935
486.3806	4.7256	1607.12	1.9457
595.1332	8.9258	1610.4154	7.1717
603.935	3.0741	2065.2243	8.4045
625.9222	81.8043	3109.5346	1.5463
631.929	0.0043	3113.1966	2.6979
648.8008	6.1629	3121.824	14.8197
661.409	28.7171	3125.3183	18.3714
733.2722	32.1167	3193.6587	226.5424
759.2032	13.5538	3240.7581	14.6646
765.7253	1.0199	3242.1282	17.2001
770.1211	92.0397	3243.9076	16.6479
784.6617	142.1205	3404.3014	104.6157
790.6568	12.003	3503.3474	0.7383
859.1325	1.7603		

Table S151. Cartesian coordinates for the optimized geometry of isomer 5a-quintet of $Co^{+}(C_2H_2)_5$.

Z	x	y	z
1	1.235703000	-0.820975000	2.488415000
6	1.114500000	-0.729362000	1.416829000
6	1.818754000	0.227732000	0.726797000
6	1.818784000	0.227861000	-0.726662000
6	1.114593000	-0.729152000	-1.416885000
6	0.000017000	-1.532021000	-0.783490000
6	-0.000033000	-1.532142000	0.783245000
6	-1.114631000	-0.729393000	1.416754000
6	-1.818817000	0.227726000	0.726691000
6	-1.818720000	0.227900000	-0.726766000
6	-1.114482000	-0.729106000	-1.416955000
1	2.529619000	0.857530000	1.246651000
1	2.529678000	0.857741000	-1.246377000
1	1.235889000	-0.820632000	-2.488470000
1	-1.235680000	-0.820538000	-2.488556000
1	-2.529560000	0.857800000	-1.246531000
1	-2.529718000	0.857516000	1.246506000
1	-1.235930000	-0.821034000	2.488327000
1	0.000007000	-2.556023000	-1.156177000
1	-0.000028000	-2.556207000	1.155764000
27	0.000009000	1.310539000	0.000115000

Table S152. Predicted frequencies (cm^{-1}) and IR intensities (km/mol) for isomer 5a-quintet of $Co^{+}(C_2H_2)_5$.

Frequency (cm^{-1})	Intensity (km/mol)	Frequency (cm^{-1})	Intensity (km/mol)
67.0197	1.2462	1014.7753	2.1447
132.332	0	1111.4663	3.9168
176.515	0.058	1174.7571	0
212.8276	1.0644	1177.0174	0.4196
241.6296	0.5789	1194.4242	3.48
285.1161	0.8086	1207.8184	0.0707
311.771	0.419	1228.5171	3.8548
440.4927	0	1248.4679	0
473.8512	0.4104	1315.3655	18.2092
475.0214	0	1361.901	8.4814
512.5812	5.9582	1371.7464	0
565.2751	2.1382	1395.6469	13.2072
582.7864	0.9535	1411.8331	0.2396
603.576	0.7582	1421.0913	5.3356
728.5912	36.998	1439.209	9.1631
763.7099	84.3085	1484.501	34.7327
774.6518	0	1551.3065	5.1395
788.0668	2.7065	1560.994	0
818.514	40.8302	3076.3704	0.1458
881.2701	0	3087.0107	0.7972
894.6854	0.6761	3179.0828	0
934.7741	3.7537	3180.1523	0.2865
938.6427	1.2342	3184.3909	0.3194
944.2667	0	3187.5241	4.5148
944.5614	0.3231	3194.2455	0
946.3274	3.873	3198.8277	7.5678
972.9228	0	3198.8456	6.7497
977.0829	5.9454	3202.762	0.0462
1002.7985	3.0943		

Table S153. Cartesian coordinates for the optimized geometry of isomer 5b-quintet of $Co^{+}(C_2H_2)_5$.

Z	x	y	z
27	-0.311571000	0.000147000	-0.000286000
6	-1.760776000	1.393547000	-0.000089000
6	-2.970979000	0.714735000	0.000171000
1	-3.927090000	1.232930000	0.000369000
1	-1.802334000	2.479997000	-0.000109000
6	-2.970709000	-0.715376000	0.000167000
6	-1.760252000	-1.393736000	-0.000090000
1	-1.801406000	-2.480201000	-0.000109000
1	-3.926623000	-1.233932000	0.000358000
6	1.840366000	1.400152000	-0.000134000
6	1.817309000	0.700929000	1.210851000
6	1.817631000	0.700435000	-1.210843000
1	1.810203000	1.241157000	2.148006000
1	1.810791000	1.240281000	-2.148221000
6	1.817623000	-0.700334000	1.211130000
6	1.817947000	-0.700830000	-1.210548000
1	1.810739000	-1.240180000	2.148507000
1	1.811325000	-1.241061000	-2.147706000
6	1.841004000	-1.400036000	0.000439000
1	1.846479000	-2.481608000	0.000660000
1	1.845364000	2.481728000	-0.000355000

Table S154. Predicted frequencies (cm^{-1}) and IR intensities (km/mol) for isomer 5b-quintet of $Co^{+}(C_2H_2)_5$.

Frequency (cm^{-1})	Intensity (km/mol)	Frequency (cm^{-1})	Intensity (km/mol)
15.3363	0	1041.157	0.2781
31.451	0.6017	1052.9252	1.8452
49.1483	1.2326	1056.0494	0.6581
64.2359	0.0216	1119.0161	11.2215
92.8515	0.1459	1140.0738	3.3468
151.5985	6.1721	1187.8098	0.0255
183.6112	2.913	1202.5002	0.0015
222.0218	0	1203.6559	0
284.0517	10.4577	1283.7501	1.3075
400.0432	0.0001	1324.6829	0.0575
405.1463	0	1341.8856	0.9893
443.5474	0.1065	1391.2967	0
589.0536	0.9646	1483.3983	60.9547
616.3424	0.0624	1497.8271	0.6577
616.9525	0	1508.8213	22.1608
662.082	57.2542	1510.192	26.5301
696.0074	0.0142	1606.5118	0.0508
726.9005	0	1611.5289	0
741.6216	5.9322	3123.616	0.0688
756.3958	110.9731	3133.7292	1.5024
913.8841	1.0906	3145.8983	5.6532
919.4443	0.0128	3151.8449	3.9891
988.6695	0.5046	3192.8771	0.001
992.0341	0.0036	3199.16	0
1003.0538	3.6888	3200.1466	0.0088
1020.0835	0	3209.4546	2.7659
1021.7788	0	3210.6202	2.7352
1025.2383	0	3216.2336	0.0029
1028.8135	0.0277		

Table S155. Cartesian coordinates for the optimized geometry of isomer 5c-quintet of $Co^{+}(C_2H_2)_5$.

Z	x	y	z
6	-1.880619000	1.401187000	0.023955000
6	-1.442276000	0.705941000	1.168064000
6	-2.316322000	0.700221000	-1.091601000
1	-1.214758000	1.248125000	2.078500000
1	-2.669710000	1.238910000	-1.960845000
6	-1.442284000	-0.706797000	1.167545000
6	-2.316325000	-0.699405000	-1.092117000
1	-1.214774000	-1.249653000	2.077583000
1	-2.669715000	-1.237451000	-1.961758000
6	-1.880629000	-1.401196000	0.022923000
1	-1.902124000	-2.482941000	0.030704000
1	-1.902106000	2.482926000	0.032533000
27	0.530835000	-0.000161000	0.396175000
6	2.702511000	-0.000267000	0.519348000
6	2.297408000	1.029046000	-0.428160000
1	2.404250000	2.101003000	-0.472929000
1	3.242682000	-0.000701000	1.453028000
1	2.404153000	-2.100635000	-0.474808000
6	2.297359000	-1.028714000	-0.429080000
1	1.582151000	0.001066000	-2.402457000
6	1.915747000	0.000599000	-1.376920000

Table S156. Predicted frequencies (cm^{-1}) and IR intensities (km/mol) for isomer 5c-quintet of $Co^+(C_2H_2)_5$.

Frequency (cm^{-1})	Intensity (km/mol)	Frequency (cm^{-1})	Intensity (km/mol)
17.6647	0.0014	1028.8576	0.5892
43.7734	0.6317	1038.7236	0.5466
50.4227	0.0025	1045.5641	1.6311
109.7027	0.9065	1052.8646	0.978
118.6929	0.0054	1179.4306	0.2867
200.574	1.6888	1200.3465	2.1407
207.6501	2.509	1200.3834	0.0026
224.1887	1.2781	1202.019	0.0009
332.5204	2.4301	1210.4084	0.0622
413.0901	1.5654	1247.2498	1.7846
428.3483	0.2193	1320.7087	4.7355
466.6221	0.1377	1326.1371	4.0233
613.3354	0.0519	1334.9995	14.1556
614.7903	0.1078	1387.2798	0.0934
660.8604	8.1382	1501.4925	18.0194
669.8756	5.4412	1502.7487	19.0328
705.015	0.169	1597.3082	3.6313
707.87	123.5026	1611.1086	3.1148
741.7346	65.2712	3169.7007	0.3774
761.0157	0.9185	3180.8633	3.2551
894.8226	0.0059	3191.097	0.3325
909.3194	17.7676	3198.2784	0.2173
909.7251	1.2826	3204.8374	0.4715
913.4214	19.003	3210.9046	0.0281
960.4379	1.9445	3233.0889	0.4569
966.3829	0.0056	3243.947	10.2534
987.837	1.9067	3247.5249	11.5882
991.1281	1.3443	3263.433	3.3944
1012.8142	1.3426		

Table S157. Cartesian coordinates for the optimized geometry of isomer 5d-quintet of $Co^{+}(C_2H_2)_5$.

Z	x	y	z
6	1.662368000	0.949695000	-0.289846000
6	2.251730000	-0.072258000	0.493633000
6	1.594230000	-1.004036000	1.344108000
6	0.390313000	-1.593936000	1.142610000
6	-0.426057000	-1.608073000	-0.093716000
6	-1.662379000	-0.949791000	-0.289490000
6	-2.251737000	0.072465000	0.493590000
6	-1.594232000	1.004545000	1.343734000
6	-0.390315000	1.594364000	1.142016000
6	0.426045000	1.608043000	-0.094321000
1	0.308023000	2.519972000	-0.679649000
1	-0.025889000	2.267260000	1.915717000
1	-2.159561000	1.333469000	2.210678000
1	-3.336023000	0.112983000	0.473380000
1	-2.316358000	-1.412537000	-1.025860000
1	-0.308050000	-2.520231000	-0.678692000
1	0.025900000	-2.266561000	1.916553000
1	2.159564000	-1.332649000	2.211166000
1	3.336017000	-0.112760000	0.473460000
1	2.316346000	1.412165000	-1.026389000
27	0.000009000	-0.000267000	-1.368306000

Table S158. Predicted frequencies (cm^{-1}) and IR intensities (km/mol) for isomer 5d-quintet of $Co^+(C_2H_2)_5$.

Frequency (cm^{-1})	Intensity (km/mol)	Frequency (cm^{-1})	Intensity (km/mol)
55.4537	0.2148	1004.0319	12.9962
128.3092	0.4832	1011.191	0.4126
148.003	0.1861	1107.9436	1.3253
152.8083	2.4958	1154.3196	0.9132
202.6288	1.2158	1223.3808	13.9322
253.3432	0.5204	1238.3914	0.0689
282.518	5.497	1249.8749	1.1648
310.6032	0.1755	1254.3461	0.0559
321.4374	0.6898	1374.6776	0.0353
367.0882	7.8017	1381.7939	3.6995
406.8773	0.0194	1414.2423	14.7357
440.6704	0.6998	1424.2839	0.0152
487.9656	0.3346	1438.0353	0.8844
595.7078	31.087	1456.0825	1.9632
612.1085	28.1342	1479.5487	0.0067
702.9275	3.2584	1489.5751	4.2977
706.0144	26.303	1569.7435	6.9628
749.0181	39.2443	1570.2171	15.5054
758.7431	63.1966	3092.3479	1.4342
804.1859	7.5965	3092.6574	4.0798
821.5134	2.3092	3119.4268	0.2737
859.587	0.0708	3119.7977	0.2668
876.5257	0.6694	3122.2586	0.2405
891.7104	7.3912	3123.3602	0.3107
916.0222	0.3224	3148.5748	0.4335
931.8304	1.0501	3148.8029	0.0064
953.7014	0.0594	3162.1712	0.9555
975.6518	11.4639	3162.5159	0.1982
994.7584	2.0832		

Table S159. Cartesian coordinates for the optimized geometry of isomer 5e-quintet of $Co^{+}(C_2H_2)_5$.

Z	x	y	z
6	-1.788505000	-0.453351000	1.400886000
6	-0.867946000	-1.256470000	0.703908000
6	-2.691295000	0.326584000	0.701460000
1	-0.261509000	-1.972596000	1.245113000
1	-3.415515000	0.925461000	1.237972000
6	-0.868048000	-1.254918000	-0.706475000
6	-2.691393000	0.328139000	-0.700252000
1	-0.261726000	-1.969876000	-1.249355000
1	-3.415685000	0.928214000	-1.235326000
6	-1.788713000	-0.450252000	-1.401535000
1	-1.808241000	-0.472513000	-2.483145000
1	-1.807872000	-0.478014000	2.482447000
27	0.978631000	0.101788000	-0.000052000
6	2.633264000	-0.866583000	-0.631701000
6	2.632835000	-0.867395000	0.631486000
1	3.089187000	-1.128460000	1.573154000
1	3.090270000	-1.126396000	-1.573397000
1	0.748199000	2.444983000	-1.619024000
6	0.787790000	2.052371000	-0.620179000
1	0.747304000	2.442833000	1.621923000
6	0.787436000	2.051556000	0.622576000

Table S160. Predicted frequencies (cm^{-1}) and IR intensities (km/mol) for isomer 5e-quintet of $Co^+(C_2H_2)_5$.

Frequency (cm^{-1})	Intensity (km/mol)	Frequency (cm^{-1})	Intensity (km/mol)
20.9015	0.0001	974.7018	1.2259
78.1845	0.6056	988.6866	4.1688
81.1866	0.0311	1011.4651	0.4278
96.6313	0.8482	1027.8097	0.1394
98.234	0.3588	1037.8401	0.3001
109.7276	0.3182	1045.9909	1.5438
171.5893	1.8767	1058.5441	1.1586
191.6027	0.0542	1181.7488	0.5071
199.5868	4.0209	1201.5539	0.6391
268.9756	125.4344	1203.25	0.1452
385.9182	10.1227	1347.724	11.771
413.1131	0.0113	1389.0153	0.1382
416.7365	0.6542	1504.7668	18.101
431.5898	7.338	1504.8009	16.1121
456.5607	0.8169	1599.1088	0.2026
534.9048	117.7745	1617.6316	2.8939
614.7479	0.3376	1675.8547	201.3352
615.4676	0.4288	1818.9161	23.2496
647.9765	90.7094	3173.189	0.6983
691.8104	19.5167	3183.6721	2.4421
703.0635	85.3337	3188.2602	0.256
705.1823	2.118	3196.2976	0.0705
712.2046	3.7283	3202.7924	0.0137
731.3857	73.8724	3208.8159	0.0496
752.6795	48.1196	3216.6397	39.8307
766.0294	39.9355	3271.4536	50.4627
797.5428	15.1712	3279.0398	86.8601
880.5534	0.0149	3345.8043	71.3535
901.1762	1.8019		

Table S161. Cartesian coordinates for the optimized geometry of isomer 5g-quintet of $Co^{+}(C_2H_2)_5$.

Z	x	y	z
6	-1.732973000	-0.868606000	1.272728000
6	-2.164734000	-1.242606000	0.001553000
6	-1.734843000	-0.870049000	-1.270630000
6	-0.310576000	1.954909000	-0.001610000
6	-0.433309000	1.393916000	-1.292267000
6	-0.872053000	0.143432000	-1.765866000
1	-0.030654000	2.029201000	-2.072339000
1	-2.109282000	-1.506244000	2.065137000
1	-2.882000000	-2.054650000	0.002556000
1	-2.112592000	-1.508333000	-2.061830000
1	0.096907000	2.958860000	-0.002578000
1	-0.639946000	0.006072000	-2.815854000
27	0.913127000	0.078177000	0.000346000
1	-0.636990000	0.010137000	2.815694000
6	-0.870072000	0.145893000	1.765720000
6	-0.432640000	1.396108000	1.290045000
1	-0.029880000	2.032794000	2.068922000
6	2.082438000	-1.622400000	-0.000400000
6	2.856529000	-0.672354000	-0.000577000
1	3.709403000	-0.023655000	-0.000956000
1	1.654018000	-2.604432000	-0.000275000

Table S162. Predicted frequencies (cm^{-1}) and IR intensities (km/mol) for isomer 5g-quintet of $Co^{+}(C_2H_2)_5$.

Frequency (cm^{-1})	Intensity (km/mol)	Frequency (cm^{-1})	Intensity (km/mol)
33.4002	0.1834	974.0894	2.8552
58.9541	0.9047	974.5819	3.0958
62.0048	0.069	985.7032	0.1592
88.0293	0.8399	996.2029	0.3158
88.5639	0.268	1186.4767	1.7828
132.8063	0.1534	1196.1494	0.6452
192.3857	0.0022	1300.4863	0.9682
220.8785	0.2957	1327.2669	2.4121
290.0551	39.5024	1347.5972	0.0013
311.2464	4.5948	1444.1137	0.0823
329.1678	0.0103	1470.3761	0.288
375.7247	4.2393	1471.0429	4.679
415.4228	0.8202	1485.0923	14.595
447.3352	0.6875	1494.7177	8.2212
567.7516	2.7824	1547.3084	1.5993
655.4686	1.4952	1553.6271	2.1303
695.4995	79.1723	1576.7442	1.2792
708.2352	23.3151	1908.1807	39.9575
723.2329	3.1448	3150.0017	0.6319
747.6744	17.5361	3151.8748	0.0348
754.3664	65.1632	3157.4363	0.3046
763.1825	14.2122	3163.675	0.2637
772.9289	0.0341	3167.202	0.0673
801.6172	17.8245	3174.8714	0.1273
814.9147	0.2829	3179.9539	0.1295
893.6532	0.03	3183.252	0.5481
919.0801	0.0001	3318.6906	141.0103
932.1597	2.2674	3399.4307	103.8677
938.8503	3.5036		

Table S163. Cartesian coordinates for the optimized geometry of isomer 5h-quintet of $Co^{+}(C_2H_2)_5$.

Z	x	y	z
6	0.117567000	2.009301000	0.980794000
6	-0.450618000	2.326842000	-0.297809000
6	-0.528408000	1.335250000	-1.234650000
6	2.583515000	-0.467138000	-0.341637000
6	2.050819000	-1.728516000	0.008845000
6	0.698759000	-1.902414000	0.209058000
1	2.727970000	-2.580072000	-0.014578000
1	-0.232556000	2.495064000	1.884967000
1	-0.868514000	3.319969000	-0.456331000
1	-0.803449000	1.591347000	-2.255644000
1	3.506181000	-0.433394000	-0.912305000
1	0.390249000	-2.902379000	0.512939000
27	-0.564844000	-0.417324000	-0.229022000
1	1.365321000	0.590203000	1.990844000
6	1.100003000	1.042118000	1.040432000
6	1.931568000	0.733999000	-0.091476000
1	2.179862000	1.574967000	-0.731588000
6	-2.575102000	-0.852667000	0.909120000
6	-2.824751000	-0.921512000	-0.270710000
1	-3.173243000	-1.008641000	-1.276958000
1	-2.461155000	-0.830894000	1.970450000

Table S164. Predicted frequencies (cm^{-1}) and IR intensities (km/mol) for isomer 5h-quintet of $Co^{+}(C_2H_2)_5$.

Frequency (cm^{-1})	Intensity (km/mol)	Frequency (cm^{-1})	Intensity (km/mol)
31.8586	1.0446	976.3482	50.6178
51.4751	0.2822	985.4569	2.7034
80.6631	1.372	1004.9759	10.605
98.9166	1.6923	1018.8812	2.1
115.6271	0.1913	1078.0907	0.8457
151.0403	8.2853	1106.6968	5.829
165.1152	3.1521	1152.3075	6.7417
191.6226	2.5234	1180.8759	2.6084
216.2156	7.3998	1245.0653	2.4349
240.49	3.8268	1272.9515	16.9848
254.0089	0.5942	1320.0381	4.2582
359.9618	5.16	1371.4198	16.2788
393.3047	1.1389	1460.4575	10.7853
431.6817	2.6434	1469.3071	23.6
480.3488	5.2904	1487.9241	26.7174
530.2413	8.0171	1508.4781	27.0635
544.0239	4.8371	1555.9613	21.8989
634.0226	0.0043	2008.1234	2.8835
674.7887	6.8289	3104.1869	2.1068
684.6871	52.3871	3111.0807	0.6309
701.3592	60.6468	3126.952	3.2782
740.7184	4.3541	3135.1521	1.3434
768.4179	62.593	3151.0356	0.4543
798.9327	36.8757	3159.8967	1.9977
818.6101	9.1148	3167.3599	1.4963
851.0621	8.3427	3178.1506	0.4548
871.0498	4.501	3361.3914	163.576
931.4789	10.2319	3456.6432	30.5864
966.596	9.145		

Table S165. Cartesian coordinates for the optimized geometry of isomer 5i-quintet of $Co^{+}(C_2H_2)_5$.

Z	x	y	z
6	-2.587398000	-0.870464000	0.518808000
6	-2.630086000	-0.062855000	-0.677689000
6	-1.710380000	0.869992000	-1.036674000
6	2.699657000	-0.840944000	-0.479938000
6	2.712108000	0.267948000	0.430088000
6	1.698067000	1.128739000	0.721793000
1	3.694655000	0.473281000	0.852634000
1	-3.555912000	-1.097128000	0.956056000
1	-3.550213000	-0.159039000	-1.252848000
1	-1.943075000	1.410495000	-1.954974000
1	3.675226000	-1.120552000	-0.865809000
1	1.959526000	1.870099000	1.484162000
27	-0.128083000	1.400722000	0.021100000
1	-1.674462000	-1.724073000	2.200225000
6	-1.496074000	-1.340017000	1.200647000
6	1.636743000	-1.557040000	-0.976801000
1	1.842015000	-2.176782000	-1.844315000
1	0.612740000	-1.400147000	1.546675000
6	-0.140149000	-1.380621000	0.764985000
6	0.292629000	-1.559364000	-0.534478000
1	-0.452959000	-1.827887000	-1.275953000

Table S166. Predicted frequencies (cm^{-1}) and IR intensities (km/mol) for isomer 5i-quintet of $Co^{+}(C_2H_2)_5$.

Frequency (cm^{-1})	Intensity (km/mol)	Frequency (cm^{-1})	Intensity (km/mol)
67.373	0.6727	1025.1705	8.3981
96.5985	0.5306	1050.2217	1.9903
111.1247	0.2516	1107.432	0.0544
121.7104	0.6107	1188.0237	4.3924
192.7631	0.2945	1194.1413	7.467
193.8669	1.4194	1243.2722	7.0528
235.8068	5.754	1259.2947	9.1429
266.9138	1.0583	1313.9423	8.6863
331.6673	2.992	1343.6816	47.3636
332.0409	3.0945	1363.616	7.1428
388.2545	26.4516	1367.3294	6.8388
416.4218	7.9354	1468.2563	24.3267
455.4544	12.905	1475.9963	114.3864
514.4347	14.0201	1494.9807	118.2285
560.5174	23.488	1526.0672	79.961
635.0062	20.7946	1549.1319	4.1944
637.5899	23.1665	1580.3629	61.8542
660.9981	80.7571	1600.9289	1.1832
803.3392	9.4858	3041.6841	0.892
816.0009	4.2222	3086.3382	1.1841
831.0816	7.6471	3108.2507	1.3855
851.2715	10.4462	3110.9933	3.7505
917.9203	10.9128	3142.6352	0.9445
929.0548	1.8288	3145.8248	0.3027
957.3562	2.7481	3151.6996	0.0487
975.9792	26.3707	3159.3178	0.3649
988.5423	5.0109	3163.3234	0.559
1005.2607	3.8132	3166.9656	1.9713
1018.0787	49.3921		

Table S167. Cartesian coordinates for the optimized geometry of isomer 5k-quintet of $Co^{+}(C_2H_2)_5$.

Z	x	y	z
6	1.476086000	-1.059793000	-0.578278000
6	2.579469000	-0.015718000	-0.291533000
6	2.486705000	-0.586962000	1.127043000
6	1.503523000	-1.497578000	0.772300000
1	1.195413000	-1.555014000	-1.500745000
1	3.538197000	-0.204221000	-0.778070000
1	3.020813000	-0.373371000	2.042794000
1	0.998759000	-2.287077000	1.312565000
27	-0.350640000	0.100291000	-0.136825000
6	2.091549000	1.390159000	-0.472358000
6	0.774331000	1.617513000	-0.510616000
1	0.362950000	2.607640000	-0.680856000
1	2.823502000	2.190006000	-0.563331000
1	-3.833973000	0.736668000	1.203578000
6	-2.937875000	0.420982000	0.675070000
6	-1.692647000	0.941070000	0.951126000
1	-1.566161000	1.756452000	1.656512000
1	-3.979272000	-0.881606000	-0.742578000
6	-3.013490000	-0.570769000	-0.351477000
6	-1.814340000	-1.094107000	-0.790504000
1	-1.812810000	-1.966113000	-1.440243000

Table S168. Predicted frequencies (cm^{-1}) and IR intensities (km/mol) for isomer 5k-quintet of $Co^{+}(C_2H_2)_5$.

Frequency (cm^{-1})	Intensity (km/mol)	Frequency (cm^{-1})	Intensity (km/mol)
35.104	2.2689	1007.8927	7.5615
62.3491	0.9203	1010.4384	4.7458
78.2349	0.5635	1030.1859	2.4623
110.4916	2.2791	1098.3803	7.4145
175.2429	2.3731	1102.5767	8.5239
204.8777	1.4517	1112.9007	0.3824
208.097	1.1806	1156.8854	8.6721
239.5725	7.0672	1177.2071	11.4628
319.1666	1.6191	1212.1644	0.7486
326.5364	2.1994	1247.3275	15.4174
376.004	2.2976	1283.5532	19.345
408.8895	4.2944	1309.0401	1.5744
467.6805	1.7515	1319.0918	1.7704
530.0124	15.316	1346.267	17.7281
538.0321	15.8215	1431.7526	9.504
635.0844	48.0063	1475.1098	9.5698
646.6324	30.6914	1487.5475	53.9691
713.179	47.1017	1572.0037	5.4245
741.9082	9.4242	3079.5182	0.7607
761.793	6.5358	3123.8808	0.3705
772.9656	16.0561	3130.543	0.6237
802.5325	12.4923	3142.3951	0.0551
884.1462	11.776	3152.8591	0.026
894.3361	2.3426	3155.9904	0.1258
919.282	3.4955	3168.2674	0.1856
929.0495	34.5088	3180.0616	4.557
944.3944	6.8379	3205.8224	3.8577
963.5451	5.9036	3227.2678	2.0551
970.7534	2.7153		

Table S169. Cartesian coordinates for the optimized geometry of isomer 5l-quintet of $Co^{+}(C_2H_2)_5$.

Z	x	y	z
6	2.519231000	0.943069000	-0.155929000
6	2.290957000	-0.574908000	-0.071948000
6	1.328772000	-0.311651000	-1.252704000
6	1.641308000	1.056771000	-1.238942000
1	3.126128000	1.632505000	0.411800000
1	3.172217000	-1.154306000	-0.355580000
1	0.948380000	-0.964405000	-2.028237000
1	1.379712000	1.882549000	-1.888188000
27	-0.616928000	-0.039533000	0.082396000
6	1.688635000	-1.060283000	1.219514000
1	2.380333000	-1.499787000	1.937535000
6	0.386173000	-0.926669000	1.502100000
1	0.000728000	-1.265551000	2.460515000
6	-2.620468000	-0.896288000	-0.937308000
6	-1.880850000	-1.830948000	-0.746182000
1	-1.318534000	-2.730284000	-0.622934000
1	-3.342987000	-0.136700000	-1.136645000
1	-2.174215000	1.281262000	2.060389000
6	-1.756478000	1.632012000	1.141450000
6	-1.343310000	2.175102000	0.142515000
1	-1.038517000	2.784869000	-0.678743000

Table S170. Predicted frequencies (cm^{-1}) and IR intensities (km/mol) for isomer 5l-quintet of $Co^{+}(C_2H_2)_5$.

Frequency (cm^{-1})	Intensity (km/mol)	Frequency (cm^{-1})	Intensity (km/mol)
20.4235	0.0551	859.2162	15.9854
47.5841	0.0541	892.3988	0.8635
64.4143	0.2594	919.4601	10.1682
79.6566	0.2555	928.2567	20.5557
81.1113	0.04	974.6299	1.2763
102.3247	0.7581	998.6268	6.7143
107.4353	0.3763	1006.9077	6.0976
160.0135	11.1573	1105.8461	4.7687
180.521	0.893	1150.8451	8.882
200.8662	0.7088	1175.1346	12.5963
215.5725	10.3271	1212.2579	0.9174
230.6104	3.1486	1294.8677	13.8279
252.5628	0.7451	1319.0383	0.5227
264.9773	2.7287	1343.2563	8.8616
361.9779	5.6863	1417.7252	2.9878
404.7936	5.7755	1575.5798	10.7236
529.9076	10.7232	1995.5521	1.6204
617.7701	47.5773	2014.1633	4.9965
642.091	0.2067	3068.3917	1.6447
647.6262	1.0001	3101.4147	2.532
676.5346	2.0847	3132.9338	2.623
680.1988	2.3445	3182.0327	2.4351
690.1308	6.0557	3200.4034	0.6803
709.4217	40.8377	3234.5085	0.8987
738.3089	39.4896	3360.9719	149.5886
765.9205	94.0266	3368.2909	150.0108
776.8262	70.3781	3454.1546	36.9726
784.5961	28.2162	3464.1642	25.1304
801.8936	51.2846		

Table S171. Cartesian coordinates for the optimized geometry of isomer 5m-quintet of $Co^{+}(C_2H_2)_5$.

Z	x	y	z
6	-0.580358000	-1.237765000	0.984601000
6	-1.859624000	-0.722036000	0.248786000
6	-1.520834000	-1.817514000	-0.757197000
6	-0.428222000	-2.245327000	-0.020672000
1	-0.373509000	-1.288278000	2.048129000
1	-2.772702000	-0.933721000	0.811640000
1	-1.993149000	-2.157407000	-1.667837000
1	0.233329000	-3.095715000	-0.121217000
27	0.816555000	0.000148000	0.000158000
1	0.232212000	3.095873000	0.121534000
6	-0.429034000	2.245271000	0.020787000
6	-1.521692000	1.817063000	0.757013000
1	-1.994336000	2.156740000	1.667562000
6	-1.859833000	0.721486000	-0.249089000
1	-2.772849000	0.932844000	-0.812166000
6	-0.580566000	1.237689000	-0.984552000
1	-0.373426000	1.288270000	-2.048020000
6	2.850607000	0.604687000	-0.091022000
6	2.850741000	-0.604096000	0.090753000
1	3.120002000	1.629275000	-0.240793000
1	3.120341000	-1.628639000	0.240461000

Table S172. Predicted frequencies (cm^{-1}) and IR intensities (km/mol) for isomer 5m-quintet of $Co^{+}(C_2H_2)_5$.

Frequency (cm^{-1})	Intensity (km/mol)	Frequency (cm^{-1})	Intensity (km/mol)
45.8658	0.1244	943.814	16.3124
77.6867	0.5718	956.8301	2.8103
107.9304	1.5645	1013.9357	0.6143
116.9548	0.6475	1014.5359	0.0359
124.1946	1.5708	1058.3005	2.1567
156.2064	1.1021	1131.2559	18.7172
191.9624	0.0966	1147.1777	1.2181
245.3782	4.9571	1157.4762	4.8167
278.8746	19.62	1192.1134	3.1044
281.7669	3.3597	1211.1538	0.4814
317.692	1.9622	1217.5372	0.5518
359.0396	2.1327	1285.7508	0.0845
417.7049	0.3638	1329.4817	5.6176
481.1662	2.2783	1344.013	15.4658
613.7476	23.9763	1344.0349	11.6612
649.8675	14.4441	1418.8827	26.4205
657.4435	3.0289	1426.5633	1.0667
699.3692	13.4413	1925.0892	33.6352
726.3957	98.4883	3053.9174	0.6681
735.9009	14.5455	3061.7003	3.4163
740.6568	81.3784	3162.9922	4.5105
762.5607	6.6126	3163.5279	1.6364
776.2053	2.7662	3200.0519	0.2385
779.2201	2.7926	3200.1038	0.9858
865.1786	6.7017	3226.3756	0.6016
886.158	1.1955	3226.5114	0.3334
889.3962	20.2782	3333.6812	124.2211
917.1057	9.6661	3415.3325	86.976
925.1768	1.5386		

Table S173. Cartesian coordinates for the optimized geometry of isomer 5n-quintet of $Co^{+}(C_2H_2)_5$.

Z	x	y	z
6	1.125855000	0.000005000	-0.919929000
6	1.939568000	1.098169000	-0.204743000
6	2.520592000	0.000010000	0.681182000
6	1.939573000	-1.098153000	-0.204738000
1	1.014220000	0.000001000	-2.004177000
1	2.718364000	1.574542000	-0.820806000
1	3.301944000	0.000013000	1.427630000
1	2.718373000	-1.574523000	-0.820800000
27	-0.795983000	-0.000007000	-0.181110000
6	1.011953000	2.102048000	0.356355000
1	1.367285000	2.975722000	0.899075000
6	-0.291702000	1.840330000	0.122352000
1	-1.083339000	2.575251000	0.273803000
6	-3.130104000	0.000052000	-0.390314000
6	-2.925297000	-0.000039000	0.799891000
1	-2.864198000	-0.000125000	1.866590000
1	-3.429330000	0.000136000	-1.416426000
1	-1.083321000	-2.575290000	0.273718000
6	-0.291693000	-1.840352000	0.122309000
6	1.011962000	-2.102044000	0.356348000
1	1.367297000	-2.975706000	0.899084000

Table S174. Predicted frequencies (cm^{-1}) and IR intensities (km/mol) for isomer 5n-quintet of $Co^{+}(C_2H_2)_5$.

Frequency (cm^{-1})	Intensity (km/mol)	Frequency (cm^{-1})	Intensity (km/mol)
60.1379	0.1934	937.2246	0.652
74.4516	0.6318	965.02	19.3056
105.1498	0.0035	1009.1137	7.291
106.1637	0.0784	1022.7261	16.596
153.1906	39.3685	1029.6309	48.3497
179.1821	1.9264	1035.5085	11.7875
231.7305	4.354	1078.5825	12.7896
240.9754	0.688	1095.4033	5.8573
279.6009	7.7877	1160.4862	36.0397
284.4421	1.2751	1188.6145	1.5471
341.1093	17.2078	1223.1557	23.388
390.9958	65.0575	1232.6801	27.0206
402.1338	5.8912	1247.2242	4.4946
417.2348	16.3599	1267.7109	4.5123
501.1953	11.4978	1325.5167	9.2871
503.5409	0.1051	1483.4691	207.7368
638.9334	1.6939	1502.5234	21.5935
653.718	5.4725	2010.5425	10.6978
661.624	82.1582	2957.1993	0.4207
693.2112	5.673	2958.345	0.4336
704.854	42.4083	3095.408	0.4097
752.0747	241.6704	3096.2872	0.6421
771.0164	43.7829	3096.6786	4.5936
806.2131	15.3988	3136.3435	0.1232
815.9876	59.3883	3136.3546	0.0428
817.7313	49.5763	3215.9556	1.9895
886.6792	1.5048	3355.7482	182.4378
894.5851	214.5431	3451.9642	32.4998
935.166	32.7066		

Table S175. Cartesian coordinates for the optimized geometry of isomer 5o-quintet of $Co^{+}(C_2H_2)_5$.

Z	x	y	z
27	-0.000002000	0.614366000	0.000002000
6	-0.614411000	-0.447951000	1.737201000
6	0.614591000	-0.447912000	1.737160000
1	1.653528000	-0.607166000	1.949659000
1	-1.653326000	-0.607262000	1.949763000
6	0.625885000	2.564829000	-0.000090000
6	-0.625988000	2.564798000	-0.000040000
1	-1.594813000	3.032916000	-0.000021000
1	1.594684000	3.033002000	-0.000143000
6	0.614453000	-0.448055000	-1.737131000
6	-0.614547000	-0.448083000	-1.737079000
1	-1.653484000	-0.607411000	-1.949526000
1	1.653379000	-0.607309000	-1.949684000
6	-4.121033000	-0.891456000	-0.000010000
6	-3.614921000	-1.977695000	0.000035000
1	-3.206033000	-2.960587000	0.000075000
1	-4.617476000	0.050055000	-0.000048000
6	3.614938000	-1.977664000	0.000002000
6	4.121044000	-0.891422000	-0.000060000
1	4.617473000	0.050095000	-0.000114000
1	3.206054000	-2.960557000	0.000055000

Table S176. Predicted frequencies (cm^{-1}) and IR intensities (km/mol) for isomer 5o-quintet of $Co^{+}(C_2H_2)_5$.

Frequency (cm^{-1})	Intensity (km/mol)	Frequency (cm^{-1})	Intensity (km/mol)
15.4573	1.1786	666.8213	53.6976
20.1451	0.8811	672.02	64.7173
24.5531	0	687.005	0
36.3111	0.0907	699.4081	1.1966
50.4287	1.6602	730.9014	119.4284
55.1663	0	734.721	0
59.3972	2.9523	745.8854	105.575
61.8474	0.008	750.659	81.5449
80.5316	0.0175	777.5518	0
81.7703	2.3308	778.7472	167.2085
118.7105	0.75	783.7404	191.6198
122.2336	1.1236	785.4527	28.3323
142.4462	7.0984	817.2721	7.6773
165.4936	0	1739.5968	140.0911
172.8548	0.0304	1854.4515	57.243
183.4981	0	1899.3973	7.3715
248.3361	5.4246	2063.2587	10.7119
251.5978	43.7047	2063.5338	3.0462
355.1702	7.768	3249.7805	55.7762
377.3743	0	3301.4112	0.0001
384.0776	18.6191	3303.3131	368.4623
435.4462	6.6216	3310.4009	60.7956
584.8559	104.7767	3378.1412	67.9877
642.4018	0.6168	3383.7351	70.5744
643.1033	0	3399.859	38.901
647.2701	4.7306	3400.1233	173.1836
649.733	0	3499.2503	2.0855
655.3579	0.556	3499.3563	0.6198
656.5773	0.9206		

Table S177. Cartesian coordinates for the optimized geometry of isomer 5p-quintet of $Co^{+}(C_2H_2)_5$.

Z	x	y	z
27	-1.165112000	0.049204000	-0.000035000
6	0.271334000	0.127411000	1.388474000
6	1.485207000	0.103520000	0.714657000
1	2.442511000	0.080436000	1.230108000
1	0.300239000	0.168181000	2.474973000
6	1.485347000	0.103247000	-0.714233000
6	0.271604000	0.126875000	-1.388293000
1	0.300717000	0.167236000	-2.474802000
1	2.442755000	0.079970000	-1.229483000
6	-3.178556000	1.272244000	-0.000562000
6	-2.277350000	2.076271000	-0.000149000
1	-1.579794000	2.884898000	-0.000054000
1	-4.045522000	0.650065000	-0.000677000
1	-2.310826000	-2.063656000	-1.663622000
6	-2.255449000	-1.945024000	-0.604003000
6	-2.255573000	-1.944796000	0.604466000
1	-2.311328000	-2.062948000	1.664119000
6	5.319391000	-0.651617000	-0.000253000
6	5.375825000	0.544799000	0.000002000
1	5.462604000	1.605090000	0.000230000
1	5.305996000	-1.715352000	-0.000483000

Table S178. Predicted frequencies (cm^{-1}) and IR intensities (km/mol) for isomer 5p-quintet of $Co^+(C_2H_2)_5$.

Frequency (cm^{-1})	Intensity (km/mol)	Frequency (cm^{-1})	Intensity (km/mol)
14.3747	0.033	769.9023	71.0171
14.8535	0.0444	772.4459	102.4728
29.2621	0.0079	773.7255	94.7297
34.9906	0.0695	780.2267	130.7191
47.1797	2.1597	790.334	51.268
57.9753	0.366	801.0969	28.7593
72.7757	0.0712	987.1798	1.4414
79.6667	0.1591	998.1892	1.0126
99.5139	0.1345	1024.6287	2.4048
101.4164	0.0294	1111.9525	15.0521
120.6691	1.1081	1135.8998	0.0342
190.0814	1.0337	1264.3797	5.9741
193.5802	3.3438	1333.2493	6.7664
207.5887	1.5038	1479.0651	58.6183
250.8525	4.9873	1494.9538	0.293
251.4312	1.8968	2004.5123	0.8114
260.5961	0.1582	2010.9479	2.5147
285.481	0.2667	2067.0191	4.8053
452.8603	0.3077	3124.8407	0.6161
577.6126	0.5679	3131.5548	0.1631
630.5072	0.0002	3144.3145	3.3606
644.7114	0.0895	3151.0305	0.179
647.4687	0.299	3365.5934	157.9021
650.3067	0.13	3367.0539	152.2066
665.8807	51.4762	3406.4468	102.3273
677.5533	1.4867	3459.1711	29.9622
689.1435	24.2104	3462.3454	27.1339
732.3837	0.1168	3505.3326	1.0282
742.016	0.8435		

Table S179. Cartesian coordinates for the optimized geometry of isomer 5s-quintet of $Co^{+}(C_2H_2)_5$.

Z	x	y	z
27	-1.100578000	-1.001732000	-0.232812000
6	-1.009626000	1.103213000	1.000024000
6	-2.363143000	1.271666000	0.757238000
1	-3.011443000	1.567784000	1.574612000
1	-0.671081000	1.048500000	2.029097000
6	-2.935591000	0.983856000	-0.522963000
6	-2.331817000	0.084090000	-1.358264000
1	-2.703702000	-0.037117000	-2.371675000
1	-3.907625000	1.417257000	-0.753640000
6	1.378012000	-0.692364000	0.831937000
6	0.295370000	-1.485783000	1.099360000
1	0.396506000	-2.315658000	1.793031000
1	2.391757000	-0.989788000	1.096267000
1	2.064631000	0.850415000	-0.534810000
6	1.223715000	0.498444000	0.053267000
6	0.010226000	1.162812000	-0.019954000
1	-0.145560000	1.869960000	-0.827696000
6	4.929934000	0.028159000	-0.920689000
6	5.015538000	0.839487000	-0.043532000
1	5.131530000	1.563053000	0.727947000
1	4.894881000	-0.689112000	-1.705769000

Table S180. Predicted frequencies (cm^{-1}) and IR intensities (km/mol) for isomer 5s-quintet of $Co^{+}(C_2H_2)_5$.

Frequency (cm^{-1})	Intensity (km/mol)	Frequency (cm^{-1})	Intensity (km/mol)
11.5079	0.2236	981.8861	56.7788
19.3304	0.195	998.5248	22.349
31.9677	0.0121	1009.1696	4.4559
53.1285	2.5105	1012.1274	21.3995
80.1766	0.6853	1074.3876	2.6862
107.356	1.7485	1111.5597	5.5111
107.9172	0.6971	1118.2529	17.1914
163.1942	1.5678	1189.168	5.3568
194.8262	1.0258	1239.3815	2.7449
230.0431	12.841	1263.1975	34.642
254.4687	0.9358	1285.7165	16.7216
375.8267	4.459	1373.1689	25.1436
403.2547	1.5798	1460.7423	1.3505
444.2488	3.6978	1462.4697	30.8117
479.7968	10.0764	1468.1853	3.4587
552.7621	4.9661	1513.6138	37.2894
566.0139	0.7313	1547.3973	13.8968
630.4495	0.0024	2066.111	6.1971
649.3296	0.254	3116.269	0.1559
704.0517	44.1239	3117.3188	1.5591
714.4763	53.6073	3153.1165	0.9663
756.0203	1.1217	3153.6746	0.3946
770.4649	90.2235	3160.4879	2.1165
782.5227	148.8923	3167.0443	7.7045
817.7803	0.1314	3176.4106	19.4861
864.1626	3.4906	3180.2581	2.7968
873.0934	5.6899	3404.5684	103.4462
945.6347	21.3643	3503.5755	1.3862
951.6269	2.7808		

Table S181. Cartesian coordinates for the optimized geometry of isomer 5t-quintet of $Co^{+}(C_2H_2)_5$.

Z	x	y	z
27	1.355463000	0.196982000	-0.728176000
6	-0.648165000	0.097817000	0.665735000
6	-0.903887000	1.422144000	0.345990000
1	-1.912487000	1.698748000	0.058561000
1	-1.394529000	-0.643049000	0.397564000
6	0.140733000	2.399120000	0.306371000
6	1.434031000	2.015028000	0.075338000
1	2.232447000	2.745891000	0.167461000
1	-0.144010000	3.449102000	0.355933000
1	0.759216000	0.313531000	2.293011000
6	0.459475000	-0.339146000	1.479973000
6	1.046558000	-1.591015000	1.386853000
1	1.603322000	-1.975748000	2.234280000
6	1.004803000	-2.347661000	0.173042000
1	1.193276000	-3.418373000	0.235088000
6	0.866955000	-1.709033000	-1.029420000
1	0.714717000	-2.297128000	-1.929991000
6	-4.173296000	-0.364534000	-0.914192000
6	-4.393530000	-0.329722000	0.262858000
1	-4.630168000	-0.305943000	1.300105000
1	-4.021333000	-0.403528000	-1.966560000

Table S182. Predicted frequencies (cm^{-1}) and IR intensities (km/mol) for isomer 5t-quintet of $Co^{+}(C_2H_2)_5$.

Frequency (cm^{-1})	Intensity (km/mol)	Frequency (cm^{-1})	Intensity (km/mol)
14.2699	0.3907	980.7752	53.3063
22.1963	0.2312	998.7068	22.6015
36.3882	0.0157	1008.1234	17.7161
56.6817	2.4431	1011.2915	2.7475
82.1685	0.6893	1072.44	1.1008
103.0195	0.8036	1113.1149	6.5289
107.3724	1.7998	1119.66	18.9297
162.6907	1.3661	1188.4704	7.6167
193.8013	0.8614	1239.1162	1.4345
230.6415	11.6902	1267.8504	33.9879
254.5978	1.2537	1288.4863	12.5858
374.7598	5.2802	1372.2032	19.9745
403.4995	2.1665	1460.2178	3.9182
444.3679	3.7949	1462.8724	30.1699
479.8737	8.5557	1469.3	1.1099
552.9237	5.4974	1511.6076	42.3706
566.6365	0.847	1545.9249	20.29
632.1241	0.0021	2065.6534	5.2797
650.8444	0.1575	3115.7864	0.1732
702.9497	43.0034	3116.7711	0.1606
712.9684	57.0237	3152.8527	0.8786
755.7468	0.4649	3153.2006	0.2429
771.4181	82.4624	3157.5624	7.5343
782.9384	136.6876	3165.5264	7.0666
819.9067	0.0025	3177.9821	11.5001
867.6914	3.5656	3179.7739	2.2554
879.0198	5.3148	3403.8283	102.0993
944.913	18.7777	3502.8733	1.1027
952.5717	5.5274		

Table S183. Cartesian coordinates for the optimized geometry of isomer 5v-quintet of $Co^{+}(C_2H_2)_5$.

Z	x	y	z
6	1.958331000	-0.711718000	0.602454000
6	1.905588000	0.945674000	0.297821000
6	0.873616000	0.941268000	1.404959000
6	0.913183000	-0.381305000	1.647217000
1	2.936744000	-0.968005000	1.010011000
1	2.862782000	1.387631000	0.576443000
1	0.351593000	1.768004000	1.870921000
1	0.430985000	-1.019966000	2.377074000
27	-0.842071000	-0.103182000	-0.339055000
6	1.402252000	1.443681000	-1.000057000
1	2.109624000	1.933135000	-1.668659000
6	0.122170000	1.273198000	-1.365649000
1	-0.287825000	1.679088000	-2.287246000
6	-2.809198000	0.714477000	0.657070000
6	-3.019492000	-0.451266000	0.416348000
1	-3.329854000	-1.460648000	0.249437000
1	-2.740741000	1.749793000	0.911173000
1	-0.184775000	-2.436811000	-1.505009000
6	0.213464000	-1.702678000	-0.808009000
6	1.498510000	-1.661071000	-0.431375000
1	2.236826000	-2.307868000	-0.904333000

Table S184. Predicted frequencies (cm^{-1}) and IR intensities (km/mol) for isomer 5v-quintet of $Co^{+}(C_2H_2)_5$.

Frequency (cm^{-1})	Intensity (km/mol)	Frequency (cm^{-1})	Intensity (km/mol)
36.5051	0.1499	952.4456	6.683
55.2515	0.2191	966.3585	0.0223
81.7234	0.6797	1002.3979	28.0091
125.8411	3.2121	1006.0323	34.0372
144.2375	1.0812	1072.4976	7.0689
192.0507	0.5417	1076.4347	11.2454
203.7371	3.0417	1114.6502	32.9494
217.8132	0.8579	1142.2371	2.7795
235.3278	6.1263	1179.4025	12.3623
282.303	0.8993	1247.8977	38.8641
323.6823	0.8826	1262.1119	26.931
360.3015	4.6069	1301.342	2.4899
375.8413	4.4523	1331.0539	1.5484
460.7673	3.8795	1359.0227	3.4905
486.2822	0.9111	1535.0615	18.5637
562.6064	6.732	1554.4783	3.8971
636.076	0.0409	1586.0335	13.2071
640.8679	2.2796	2003.2415	0.9687
661.3347	6.9017	3082.9383	0.9737
672.2424	0.6982	3089.3822	1.3085
707.1682	127.5421	3103.6626	0.0251
738.8218	18.6022	3105.8476	0.8625
770.4474	79.5764	3132.5458	2.837
791.4217	43.3178	3138.2288	1.7412
832.161	44.2079	3179.4689	0.0729
855.9944	4.8294	3205.4695	1.0196
864.3063	9.0352	3355.3072	165.8081
908.6554	45.1571	3450.3453	45.1046
917.9437	0.5446		

Table S185. Cartesian coordinates for the optimized geometry of isomer 5w-quintet of $Co^{+}(C_2H_2)_5$.

Z	x	y	z
6	2.807009000	0.702675000	-0.085508000
6	1.828120000	1.715221000	-0.166298000
6	0.455601000	1.615142000	-0.224866000
6	2.807011000	-0.702644000	-0.085617000
6	1.828126000	-1.715181000	-0.166564000
6	0.455607000	-1.615103000	-0.225114000
1	2.260041000	-2.713913000	-0.189238000
1	3.814458000	1.103274000	-0.029223000
1	2.260028000	2.713958000	-0.188812000
1	-0.031380000	2.585780000	-0.346867000
1	3.814462000	-1.103246000	-0.029397000
1	-0.031367000	-2.585723000	-0.347278000
27	-0.665478000	0.000006000	-0.051411000
6	-2.360119000	-0.604463000	-1.535493000
6	-2.360089000	0.604678000	-1.535451000
1	-2.462289000	1.662986000	-1.631999000
1	-2.462376000	-1.662759000	-1.632115000
1	-0.051532000	-0.000451000	2.827027000
6	-1.000599000	-0.000272000	2.340372000
6	-2.125516000	-0.000078000	1.907021000
1	-3.153041000	0.000084000	1.621089000

Table S186. Predicted frequencies (cm^{-1}) and IR intensities (km/mol) for isomer 5w-quintet of $Co^{+}(C_2H_2)_5$.

Frequency (cm^{-1})	Intensity (km/mol)	Frequency (cm^{-1})	Intensity (km/mol)
26.111	0	842.4095	0.6355
37.9823	0.4441	848.9268	4.9139
67.3003	0.0984	857.1498	0.1642
80.538	0.0099	981.0377	2.6096
85.5793	0.0045	1024.2391	1.0904
108.9772	0.6018	1045.0159	0.3849
126.0235	0.3888	1059.2641	0.2882
165.9719	2.1613	1182.0176	10.1607
186.7584	0.3442	1245.5891	3.4256
191.2752	0.0799	1328.5522	3.4903
196.2959	3.9487	1335.2281	25.5843
217.6285	0.1609	1395.3895	2.4457
259.6999	5.2826	1490.8675	79.2188
302.5973	2.1081	1514.1363	9.4332
334.2271	2.8292	1547.1042	25.1553
435.5024	3.5101	1561.5003	19.7658
495.5117	2.5092	1999.6648	0.4048
513.7438	0.1724	2023.6364	6.6008
604.3226	87.1243	3064.0573	5.1662
637.8182	11.8305	3064.2794	4.0287
640.0971	0.1817	3112.3864	9.3344
646.9569	0.3363	3113.3538	1.1589
675.7904	0.0213	3145.2502	0.8312
677.9177	1.5414	3163.373	2.748
679.3778	6.3088	3365.2093	137.335
769.5965	85.9566	3377.8681	135.3305
773.5541	60.0658	3458.1678	33.602
786.0674	51.9676	3473.8749	15.935
802.9469	55.8131		

Table S187. Cartesian coordinates for the optimized geometry of isomer 5x-quintet of $Co^{+}(C_2H_2)_5$.

Z	x	y	z
6	-2.286062000	-1.108946000	0.779002000
6	-2.570980000	-0.729155000	-0.555509000
6	-0.984041000	-0.917794000	1.224888000
1	-3.578254000	-0.815401000	-0.952837000
1	-0.745296000	-1.175414000	2.252612000
6	-1.502646000	-0.249948000	-1.301684000
1	-1.678282000	0.039195000	-2.335448000
1	-3.060241000	-1.531790000	1.413870000
27	0.133327000	-0.226315000	-0.235753000
6	2.109120000	-0.563087000	-0.971379000
6	1.978176000	-1.395797000	0.182874000
1	1.954591000	-2.458670000	0.362955000
1	2.300529000	-0.750580000	-2.016540000
1	2.370278000	1.668055000	-0.264893000
6	2.176314000	0.620080000	-0.107777000
1	1.841615000	-0.027357000	2.088858000
6	1.872243000	-0.195804000	1.025223000
6	-0.541329000	2.646574000	0.889412000
6	-0.561901000	2.823330000	-0.297317000
1	-0.540373000	2.541867000	1.948748000
1	-0.597770000	3.043885000	-1.338369000

Table S188. Predicted frequencies (cm^{-1}) and IR intensities (km/mol) for isomer 5x-quintet of $Co^{+}(C_2H_2)_5$.

Frequency (cm^{-1})	Intensity (km/mol)	Frequency (cm^{-1})	Intensity (km/mol)
33.7779	1.4695	904.1296	9.4185
51.7097	0.7906	950.4763	1.818
58.9114	0.2973	961.2777	9.6514
61.1804	1.5189	974.4229	9.8877
78.1144	1.0333	1003.6554	0.1314
88.2346	1.4447	1025.7836	4.3423
107.3142	1.9036	1111.6673	8.1533
121.3363	1.0981	1117.7368	1.2121
190.5173	0.1586	1134.9051	0.1795
233.2731	1.6158	1208.185	0.0187
269.7293	0.2925	1266.9417	3.4012
308.2803	2.1037	1272.4773	1.1116
324.4809	0.1221	1320.6135	13.8319
353.0327	8.7025	1339.5474	11.977
478.7264	0.3825	1372.6354	7.7036
512.7175	1.3236	1466.9985	63.3789
550.9021	2.2069	1494.8772	0.9925
630.7847	0.1874	2053.7656	21.6286
649.872	69.0188	3132.1517	0.2472
654.5654	4.2779	3141.0511	0.1797
699.0034	4.7427	3156.6984	1.6678
726.3693	70.7969	3164.1652	1.2981
754.4401	10.7641	3235.5565	6.3579
774.138	55.7242	3253.1315	9.152
776.5174	6.2365	3256.188	11.7139
792.9327	123.3165	3272.5865	7.0523
815.8062	2.3442	3398.4028	110.3303
821.1543	0.208	3496.9803	1.62
857.3654	3.457		

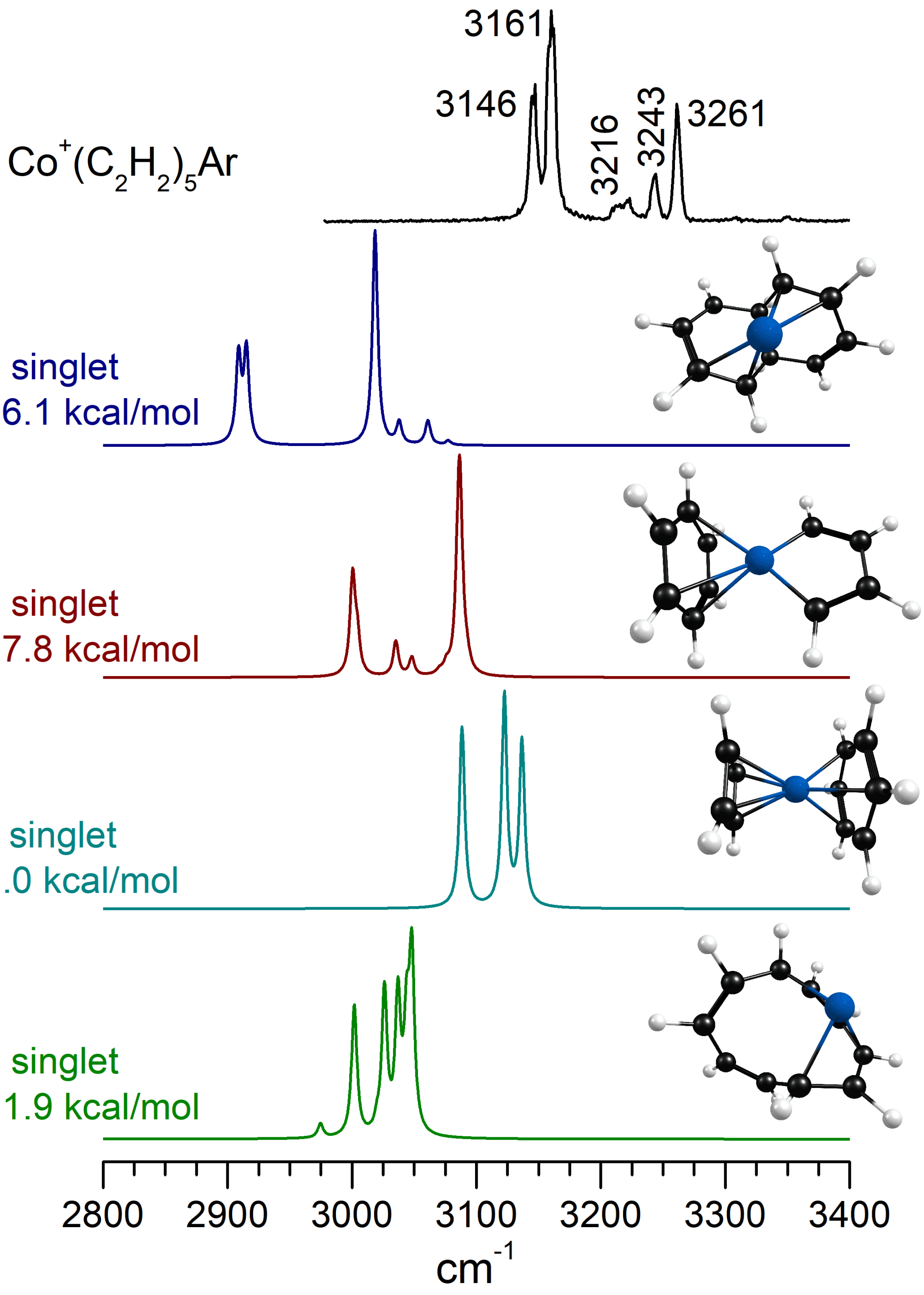

Figure S49. The experimental spectrum for $Co^{+}(C_2H_2)_5Ar$ with simulated spectra for $Co^{+}(C_2H_2)_5$ as a singlet and predicted isomers 5a-5d of singlet-$Co^{+}(C_2H_2)_5$.

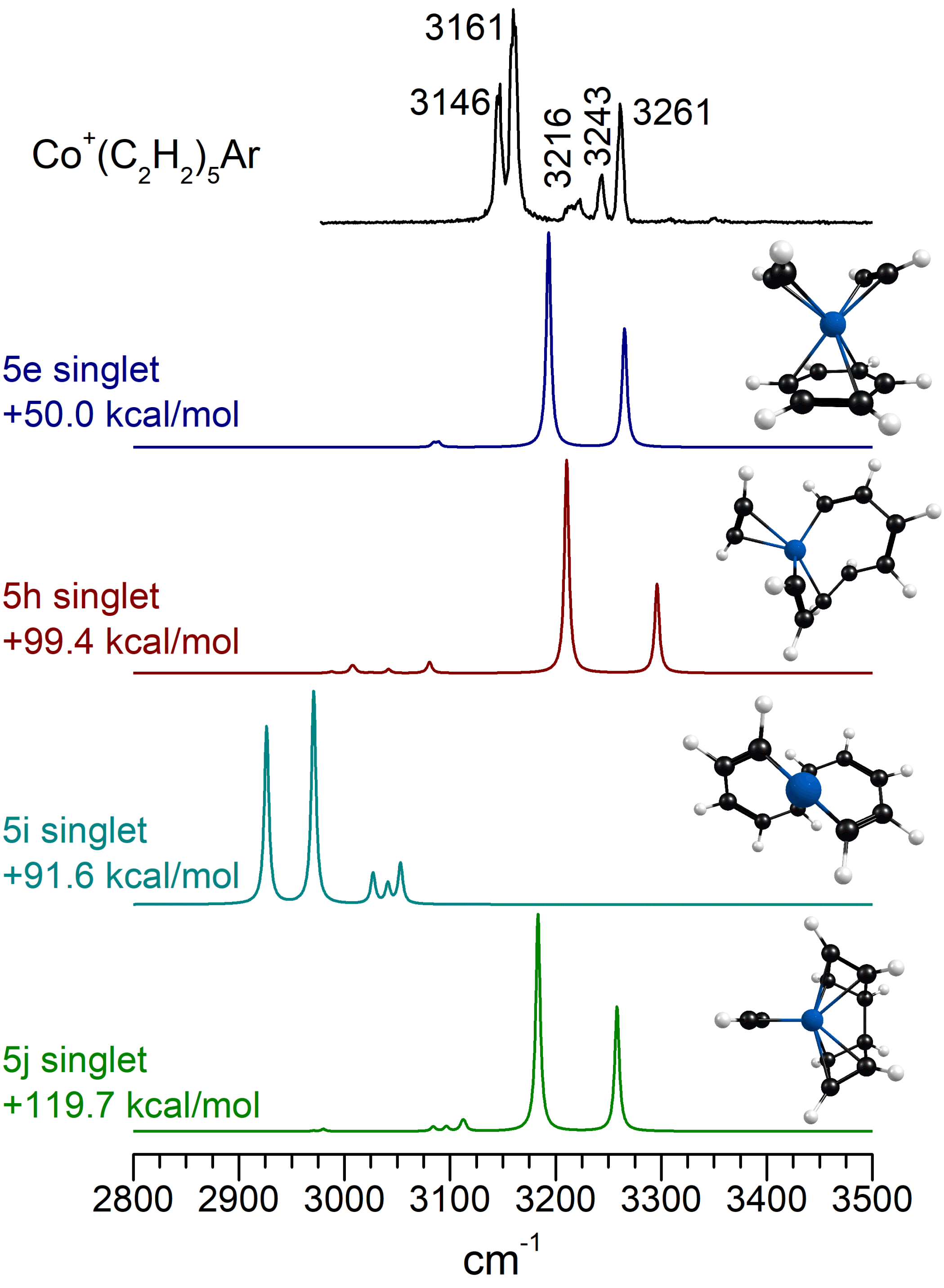

Figure S50. The experimental spectrum for $Co^{+}(C_2H_2)_5Ar$ with simulated spectra for $Co^{+}(C_2H_2)_5$ as a singlet and predicted isomers 5e-5j of singlet-$Co^{+}(C_2H_2)_5$.

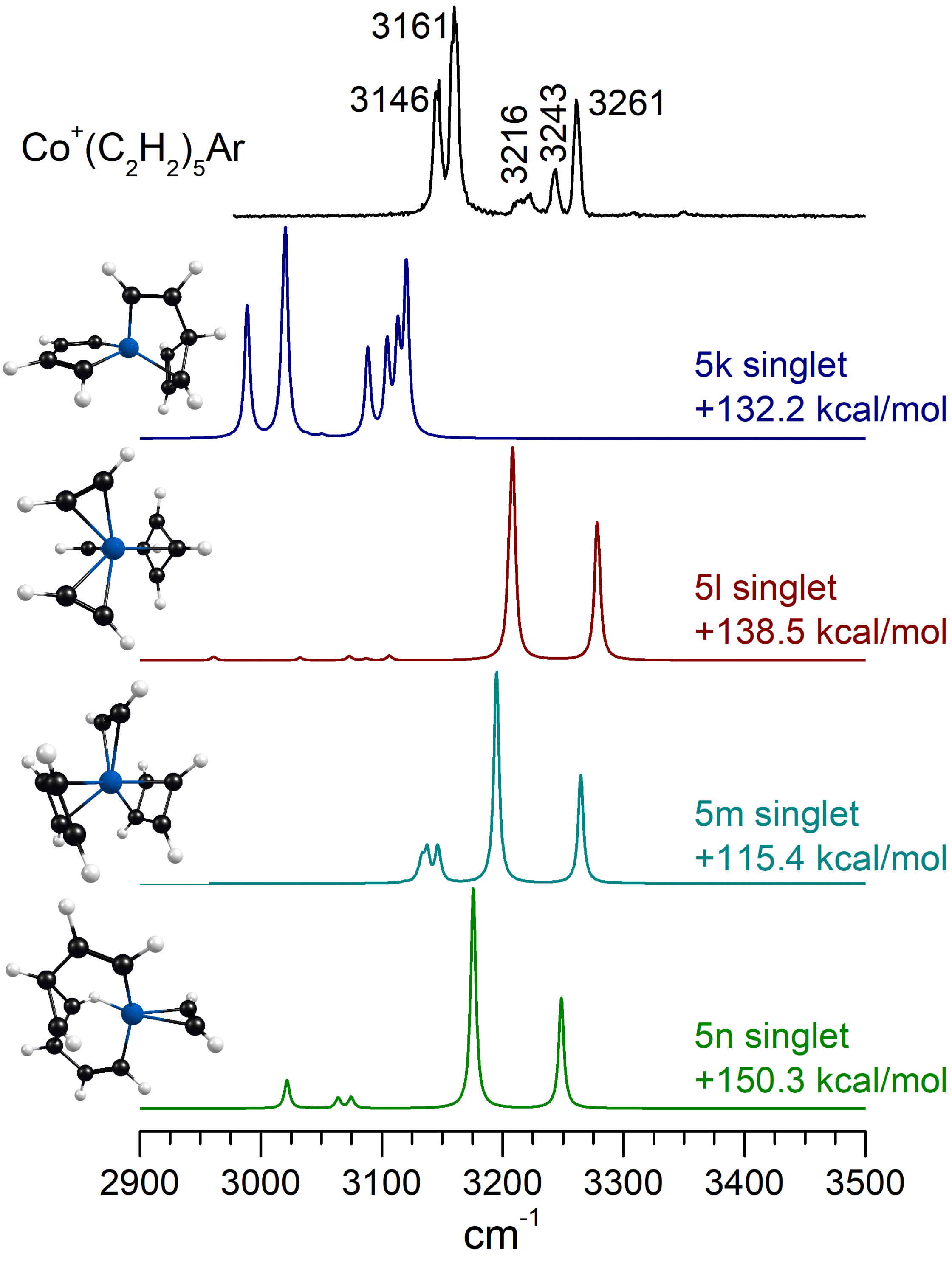

Figure S51. The experimental spectrum for $Co^{+}(C_2H_2)_5Ar$ with simulated spectra for $Co^{+}(C_2H_2)_5$ as a singlet and predicted isomers 5k-5n of singlet-$Co^{+}(C_2H_2)_5$.

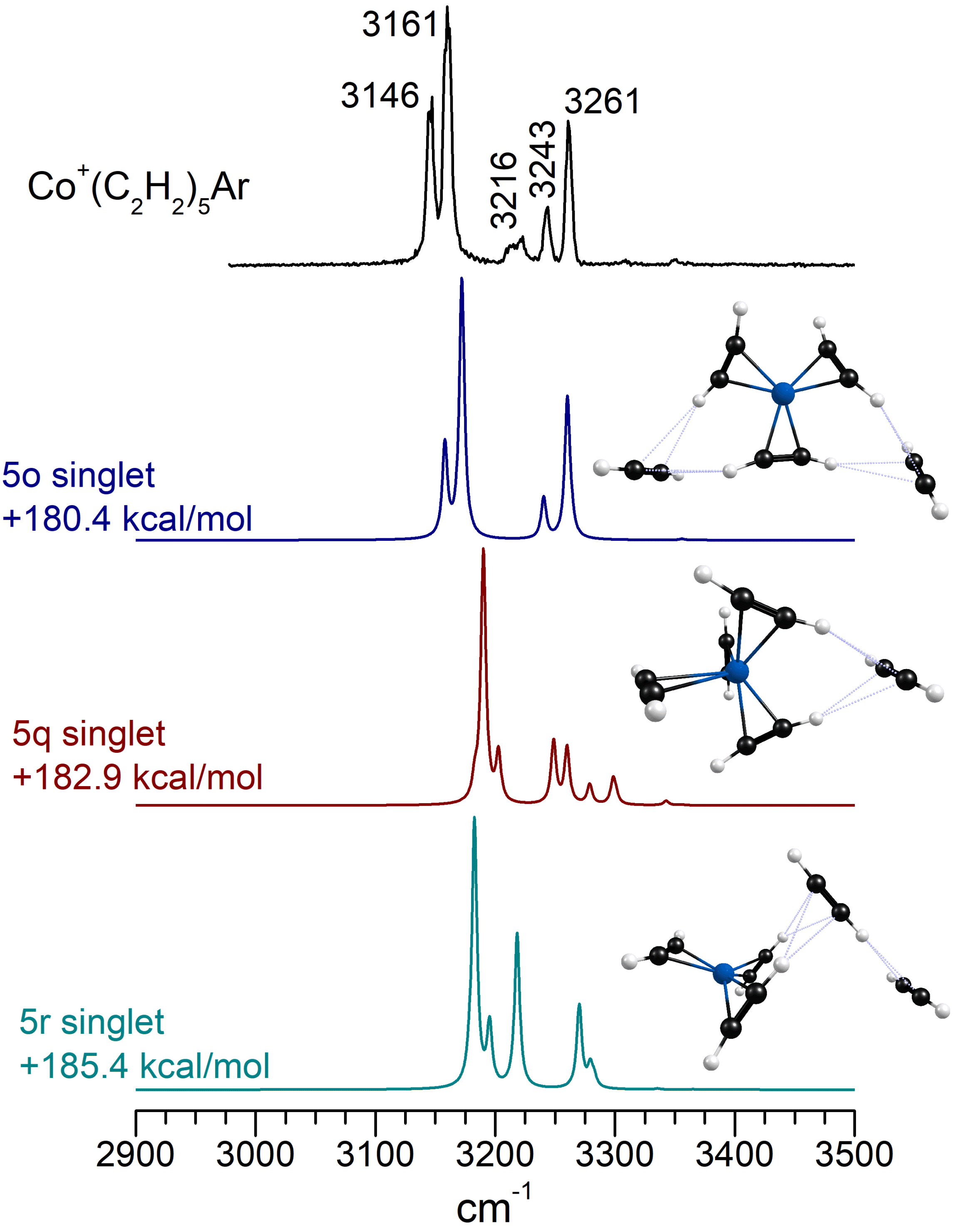

Figure S52. The experimental spectrum for $Co^{+}(C_2H_2)_5Ar$ with simulated spectra for $Co^{+}(C_2H_2)_5$ as a singlet and predicted isomers 5o-5r of singlet-$Co^{+}(C_2H_2)_5$.

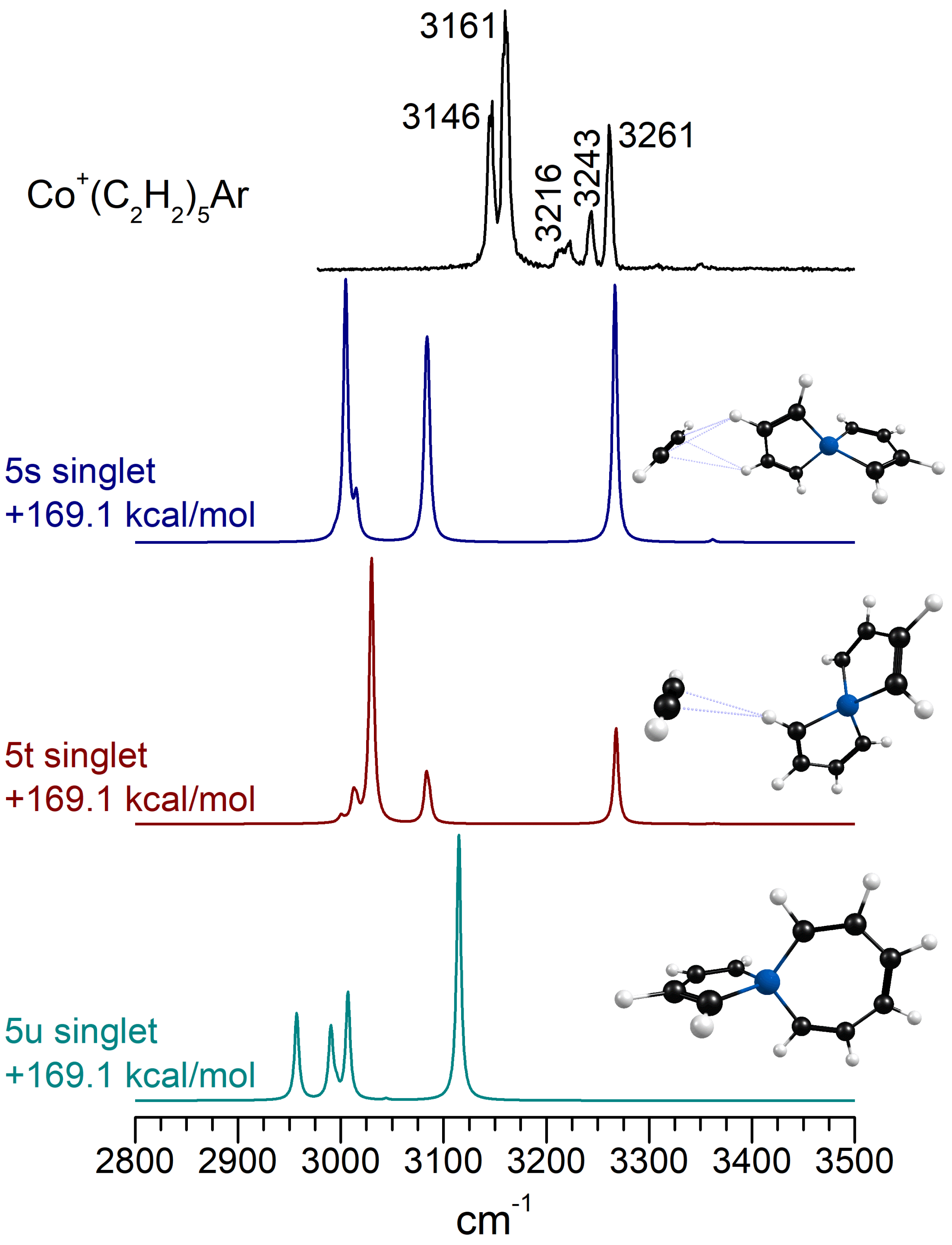

Figure S53. The experimental spectrum for $Co^{+}(C_2H_2)_5Ar$ with simulated spectra for $Co^{+}(C_2H_2)_5$ as a singlet and predicted isomers 5s-5u of singlet-$Co^{+}(C_2H_2)_5$.

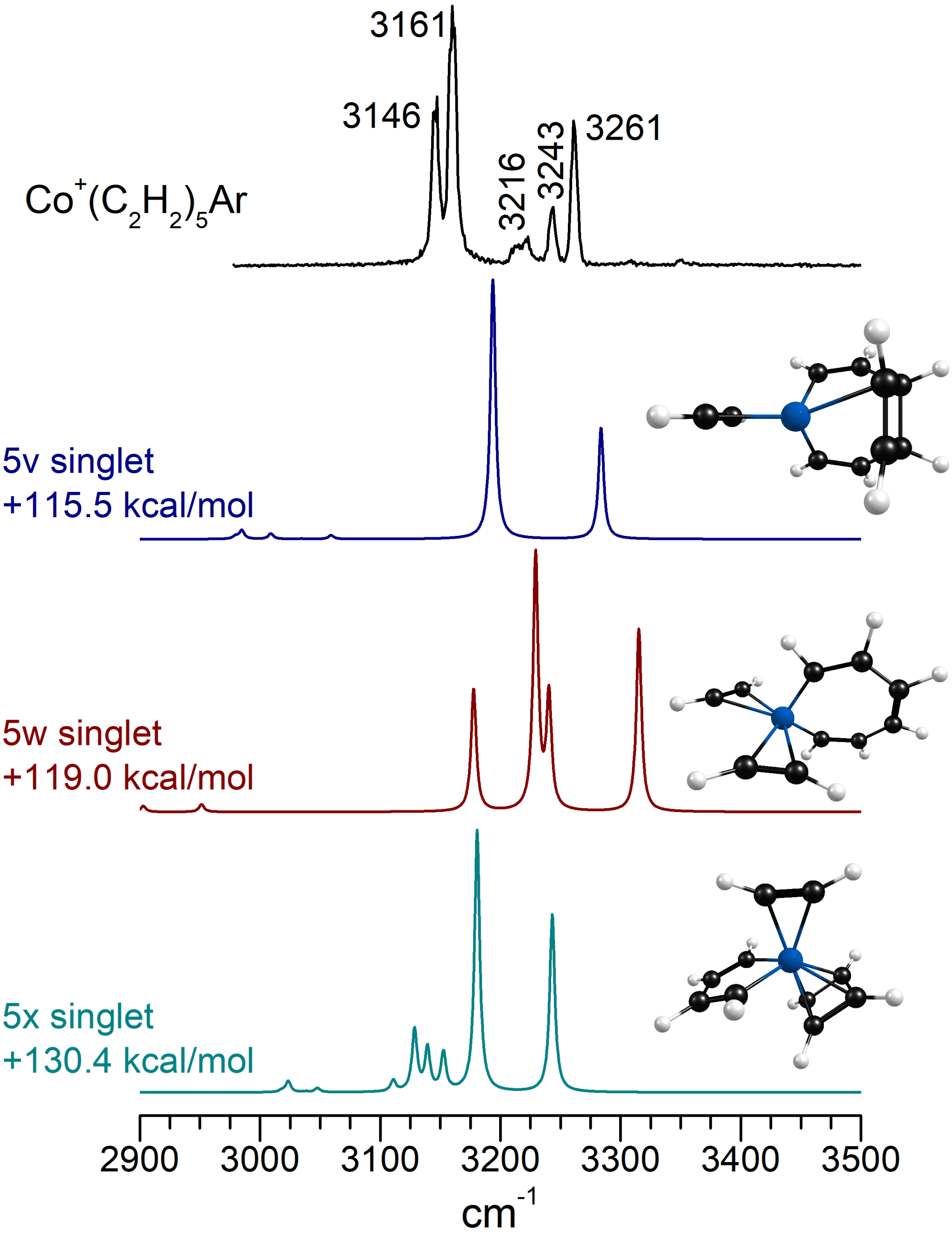

Figure S54. The experimental spectrum for $Co^{+}(C_2H_2)_5Ar$ with simulated spectra for $Co^{+}(C_2H_2)_5$ as a singlet and predicted isomers 5v-5x of singlet-$Co^{+}(C_2H_2)_5$.

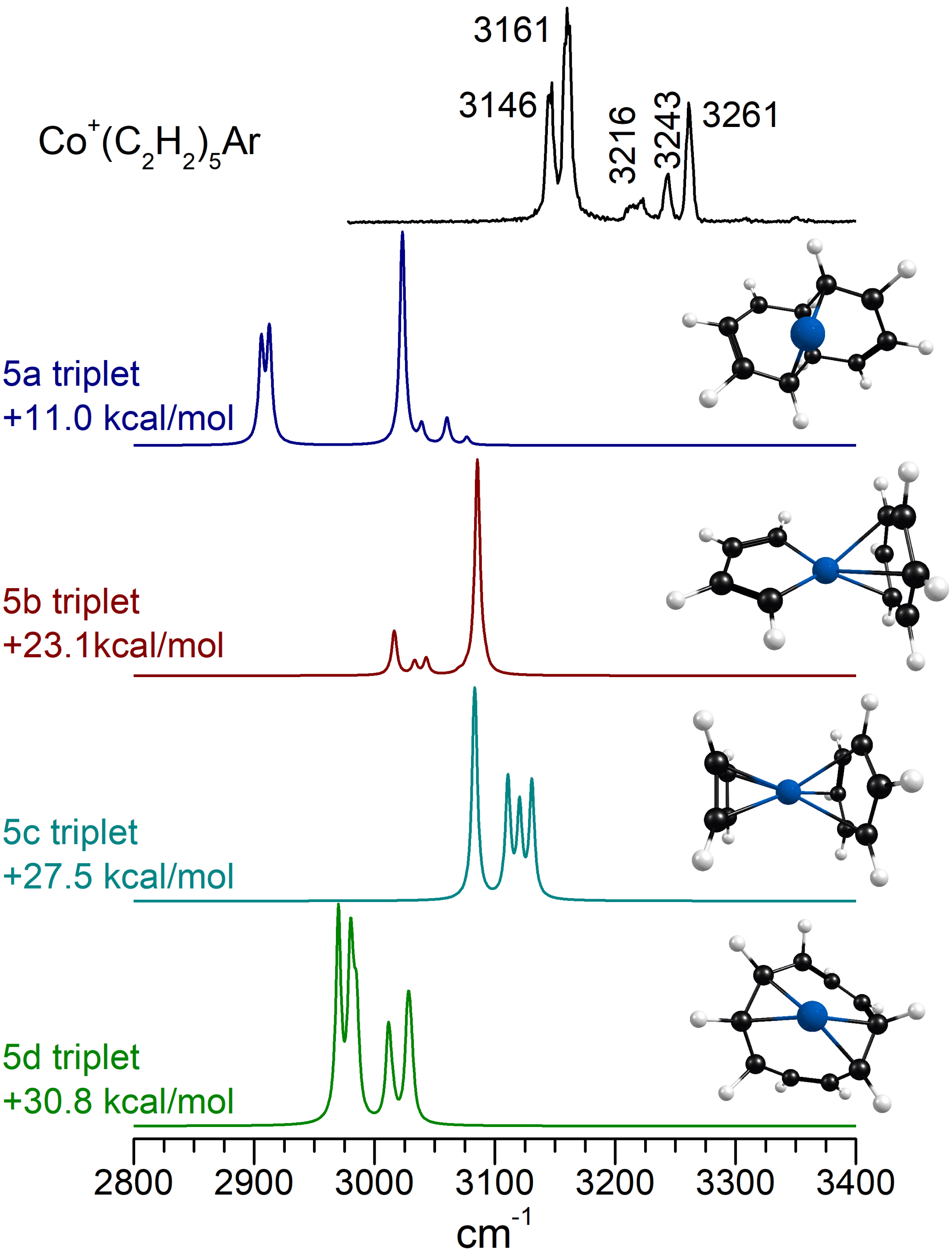

Figure S55. The experimental spectrum for $Co^{+}(C_2H_2)_5Ar$ with simulated spectra for $Co^{+}(C_2H_2)_5$ as a triplet and predicted isomers 5a-5d of triplet-$Co^{+}(C_2H_2)_5$.

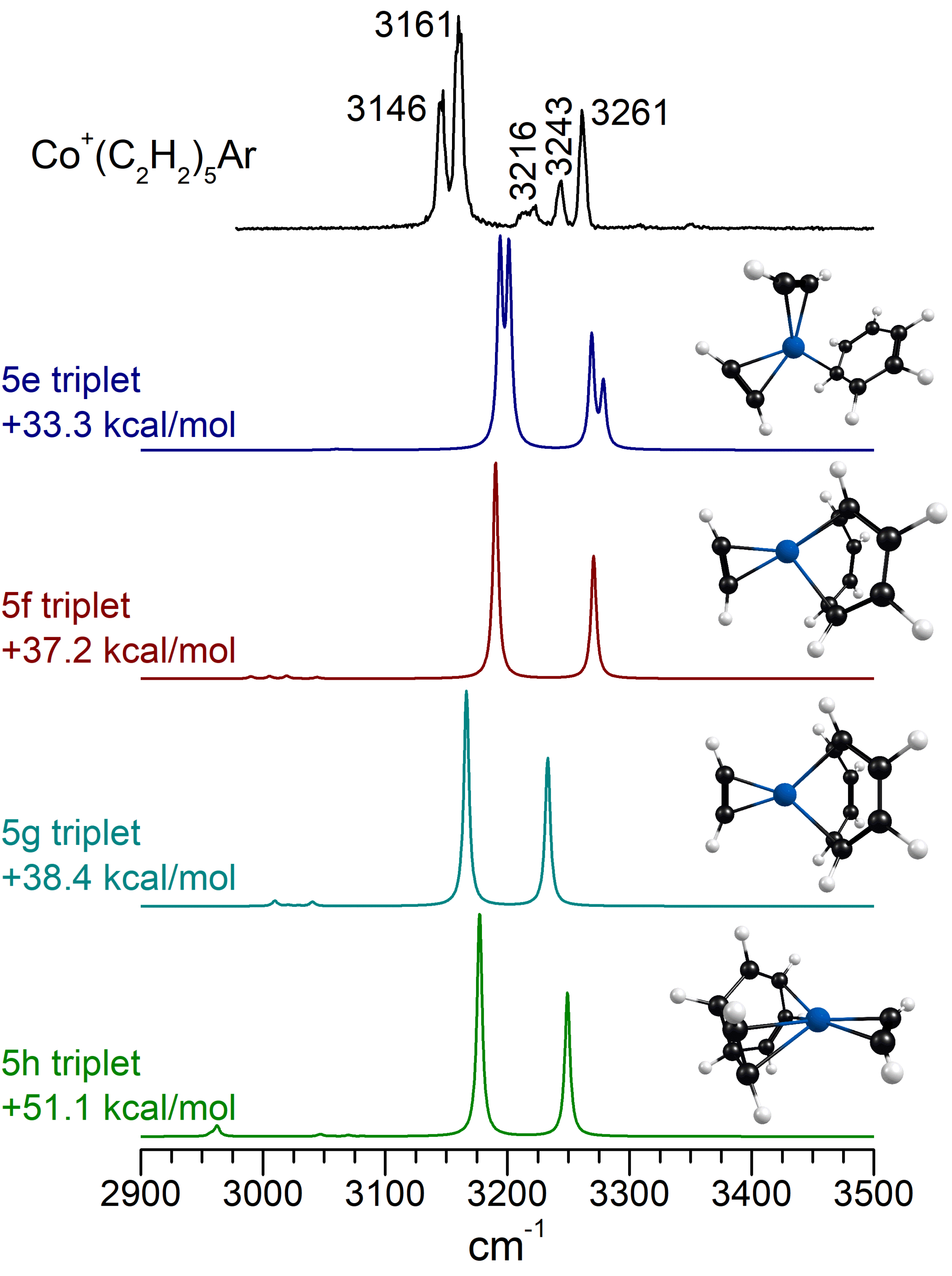

Figure S56. The experimental spectrum for $Co^{+}(C_2H_2)_5Ar$ with simulated spectra for $Co^{+}(C_2H_2)_5$ as a triplet and predicted isomers 5e-5h of triplet-$Co^{+}(C_2H_2)_5$.

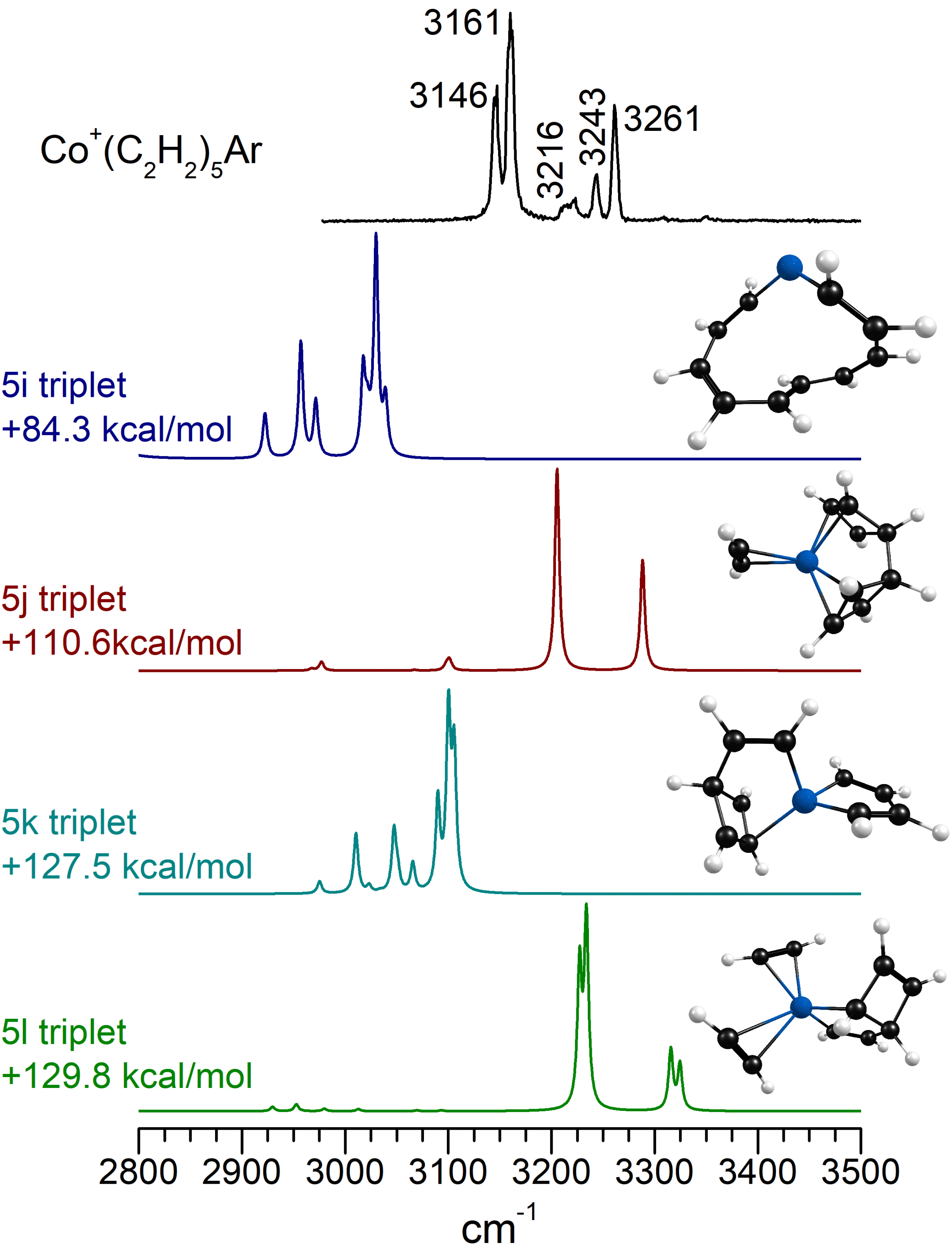

Figure S57. The experimental spectrum for $Co^+(C_2H_2)_5Ar$ with simulated spectra for $Co^+(C_2H_2)_5$ as a triplet and predicted isomers 5i-5l of triplet-$Co^+(C_2H_2)_5$.

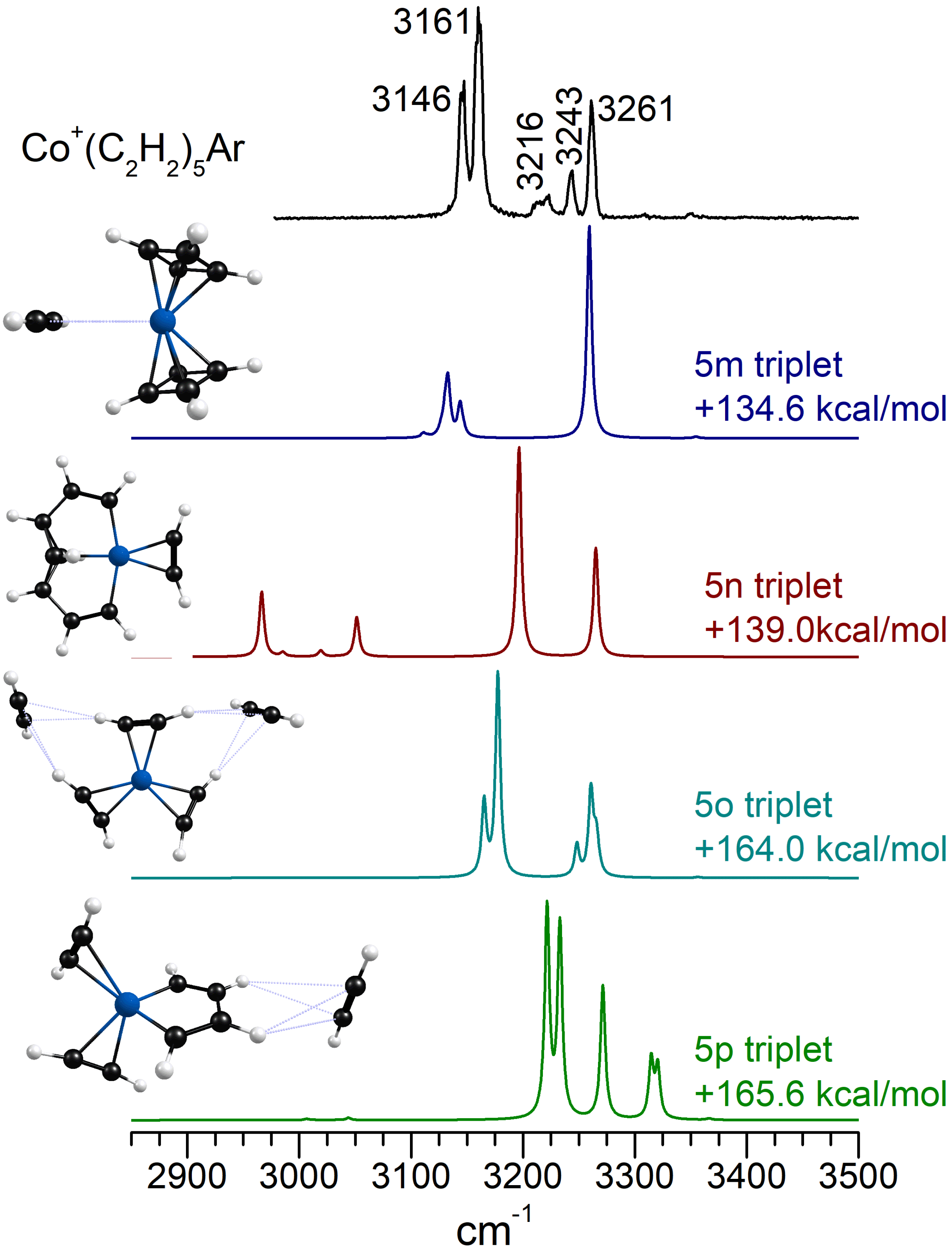

Figure S58. The experimental spectrum for $Co^{+}(C_2H_2)_5Ar$ with simulated spectra for $Co^{+}(C_2H_2)_5$ as a triplet and predicted isomers 5m-5p of triplet-$Co^{+}(C_2H_2)_5$.

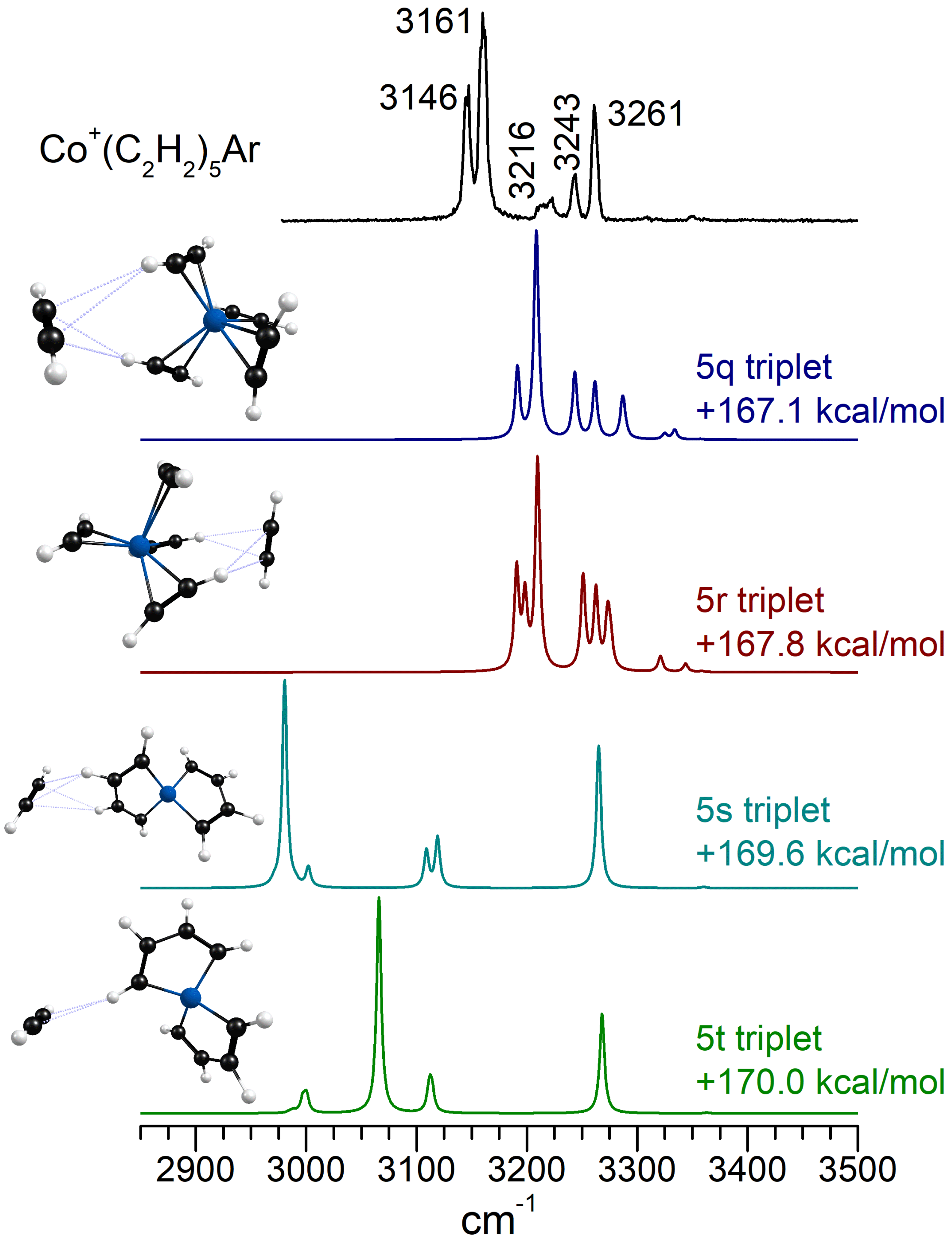

Figure S59. The experimental spectrum for $Co^+(C_2H_2)_5Ar$ with simulated spectra for $Co^+(C_2H_2)_5$ as a triplet and predicted isomers 5q-5t of triplet-$Co^+(C_2H_2)_5$.

Table S189. $Co^+(C_2H_2)_6$ calculated at the B3LYP/Def2TZVP level of theory using Gaussian16.

Isomer	2s + 1	E (hartree)	Relative E (kcal/mol)
6a	1	-1847.086164	+39.5
6a	3	-1847.149159	+0.0
6b	1	-1846.662429	+305.4
6b	3	-1846.688477	+289.1
6c	3	-1846.685426	+291.0

Table S190. Cartesian coordinates for the optimized geometry of isomer 6a-singlet of $Co^{+}(C_2H_2)_6$.

Z	x	y	z
6	-1.856348000	0.032835000	-1.402651000
6	-1.856432000	1.231215000	-0.672959000
6	-1.856512000	-1.198468000	-0.729873000
1	-1.851730000	2.179969000	-1.191640000
1	-1.851932000	-2.121952000	-1.292300000
6	-1.856493000	1.198469000	0.729874000
6	-1.856436000	-1.231213000	0.672959000
1	-1.851901000	2.121953000	1.292301000
1	-1.851737000	-2.179967000	1.191640000
6	-1.856333000	-0.032834000	1.402650000
1	-1.851461000	-0.058173000	2.483642000
1	-1.851477000	0.058173000	-2.483642000
27	0.000002000	0.000005000	0.000009000
6	1.856428000	-0.675649000	-1.229748000
6	1.856497000	0.727252000	-1.200064000
1	1.851908000	1.287661000	-2.124774000
1	1.851724000	-1.196400000	-2.177368000
6	1.856336000	-1.402724000	-0.029777000
1	1.851456000	-2.483768000	-0.052755000
6	1.856504000	-0.727259000	1.200053000
1	1.851920000	-1.287668000	2.124763000
6	1.856440000	0.675643000	1.229736000
1	1.851746000	1.196393000	2.177355000
6	1.856341000	1.402715000	0.029765000
1	1.851474000	2.483760000	0.052743000

Table S191. Predicted frequencies (cm^{-1}) and IR intensities (km/mol) for isomer 6a-singlet of $Co^+(C_2H_2)_6$.

Frequency (cm^{-1})	Intensity (km/mol)	Frequency (cm^{-1})	Intensity (km/mol)
-9.0857	0.0001	1045.9343	0.0459
82.5022	0	1045.9421	0.0501
82.5193	0	1047.7497	4.1493
107.7957	0.11	1047.7611	4.1448
107.7962	0.11	1183.5474	0.0002
169.4968	0	1183.578	0.0002
191.6078	4.5547	1189.7525	0
193.3935	2.5711	1191.6425	0
193.4428	2.5744	1192.127	0.0002
394.5152	0.0005	1193.0871	0.0001
405.9491	0	1329.2231	0.0015
407.0085	0	1329.2967	0.0015
411.66	0.0003	1383.9984	0
611.6303	0.0001	1384.1735	0
616.8545	0	1500.4731	0.1589
617.9219	0	1500.4789	0.1942
619.7635	0.0001	1501.3262	31.3639
682.6254	0	1501.3338	31.3294
682.6361	0	1583.0667	0
760.3639	151.5163	1587.3675	0.0005
774.7958	0	1588.6049	0.0004
903.0929	0.001	1589.0153	0
903.0976	0.0011	3194.075	0
914.5773	1.7878	3194.0794	0
914.5833	1.7876	3200.1346	0
993.6089	0	3200.3736	0
994.4848	0	3200.6134	0
999.601	0.1762	3200.8664	0
999.7323	0.0011	3211.0024	0.0052
1000.2566	0.0007	3211.0029	0.0054
1000.3371	0	3211.6121	0.9998
1019.1285	0	3211.6133	1.0001
1019.1425	0	3217.6081	0.0579
1036.3864	0	3217.9939	0
1036.4005	0		

Table S192. Cartesian coordinates for the optimized geometry of isomer 6b-singlet of $Co^{+}(C_2H_2)_6$.

Z	x	y	z
27	0.000023000	-0.001029000	-0.000066000
6	-2.034992000	0.456902000	-0.155688000
6	-1.453788000	1.493517000	0.154111000
1	-1.247322000	2.518273000	0.393406000
1	-2.799564000	-0.255652000	-0.395870000
6	-0.566599000	-2.005510000	0.156864000
6	0.621179000	-1.989364000	-0.156261000
1	1.619413000	-2.297758000	-0.398001000
1	-1.556051000	-2.340927000	0.398725000
6	1.412401000	1.532647000	-0.154813000
6	2.021765000	0.512370000	0.155126000
1	2.805589000	-0.178933000	0.395360000
1	1.177988000	2.551324000	-0.394300000
6	4.411056000	-2.222323000	-0.560602000
6	4.204237000	-2.591322000	0.560939000
1	4.059091000	-2.942985000	1.555477000
1	4.635268000	-1.914926000	-1.555127000
6	-4.131479000	-2.705751000	-0.560707000
6	-4.348603000	-2.342095000	0.560631000
1	-4.581396000	-2.040553000	1.554976000
1	-3.976505000	-3.053722000	-1.555062000
6	0.142837000	4.935472000	0.561479000
6	-0.278117000	4.930037000	-0.560825000
1	-0.654663000	4.970512000	-1.556026000
1	0.518142000	4.985654000	1.556707000

Table S193. Predicted frequencies (cm^{-1}) and IR intensities (km/mol) for isomer 6b-singlet of $Co^+(C_2H_2)_6$.

Frequency (cm^{-1})	Intensity (km/mol)	Frequency (cm^{-1})	Intensity (km/mol)
17.8981	0.3449	736.2281	0.2513
27.265	0.097	771.9898	4.3461
27.401	0.1357	777.6164	86.5357
32.6645	0.0863	777.7403	89.1892
32.8695	0.0963	778.2155	31.3322
41.9225	0.0001	783.974	28.1371
57.1742	0.0025	788.8948	132.1932
57.307	0.0059	790.5698	252.2223
69.062	0.0003	791.0025	150.489
70.552	0.0704	793.4822	0.0025
75.2756	3.8349	799.4695	14.1274
75.2958	4.1285	799.5578	13.7842
93.3213	2.3143	807.6647	139.2425
96.7052	0.4213	844.7132	14.3077
97.4984	0.2891	844.9174	10.2794
119.6828	0.2487	856.7881	0.0003
121.1906	0.0001	1894.6204	1.2069
138.3582	0.1236	1895.9756	0.5246
142.3473	0.019	1897.5829	3.9783
188.4346	9.0408	2062.1493	15.0318
190.1097	7.7763	2062.1524	14.5878
266.9763	0.2856	2062.5024	0
269.9985	0.9529	3281.901	6.2669
284.493	2.2903	3288.8185	516.1934
404.253	2.0575	3288.8872	515.1956
422.3556	9.8607	3366.3594	158.4655
428.7853	0.299	3366.6688	134.8221
640.8239	0.0001	3375.1808	0.0182
640.9709	0.0003	3396.4844	25.5995
640.9911	0.0031	3396.5169	21.8237
669.7019	0.2498	3396.6591	306.0828
669.71	0.1444	3496.2421	1.9702
670.0377	0.4888	3496.2702	2.1353
731.6947	0.0001	3496.3375	0.0788
735.8653	0.2502		

Table S194. Cartesian coordinates for the optimized geometry of isomer 6a-triplet of $Co^{+}(C_2H_2)_6$.

Z	x	y	z
6	-1.866226000	0.974573000	1.009518000
6	-1.866244000	-0.387004000	1.348679000
6	-1.866076000	1.361609000	-0.339212000
1	-1.862766000	-0.685229000	2.388021000
1	-1.862533000	2.410797000	-0.600645000
6	-1.866145000	-1.361604000	0.339154000
6	-1.866093000	0.387020000	-1.348748000
1	-1.862721000	-2.410804000	0.600548000
1	-1.862508000	0.685210000	-2.388102000
6	-1.866141000	-0.974548000	-1.009570000
1	-1.862490000	-1.725475000	-1.787545000
1	-1.862677000	1.725501000	1.787501000
27	-0.000021000	-0.000013000	0.000006000
6	1.866159000	1.349456000	0.384461000
6	1.866075000	0.341782000	1.360983000
1	1.862593000	0.605175000	2.409684000
1	1.862588000	2.389366000	0.680701000
6	1.866225000	1.007696000	-0.976456000
1	1.862686000	1.784227000	-1.728888000
6	1.866160000	-0.341775000	-1.360909000
1	1.862707000	-0.605204000	-2.409598000
6	1.866195000	-1.349459000	-0.384415000
1	1.862709000	-2.389378000	-0.680632000
6	1.866180000	-1.007683000	0.976496000
1	1.862556000	-1.784223000	1.728908000

Table S195. Predicted frequencies (cm^{-1}) and IR intensities (km/mol) for isomer 6a-triplet of $Co^+(C_2H_2)_6$.

Frequency (cm^{-1})	Intensity (km/mol)	Frequency (cm^{-1})	Intensity (km/mol)
11.715	0	1037.4587	0.0001
103.8219	0.0002	1045.9489	0.0043
103.836	0.0002	1047.8302	4.1817
108.0921	0.1085	1047.8434	4.183
108.1019	0.1085	1182.7246	0
170.0199	0	1182.7602	0
196.8242	5.1063	1191.5309	0
209.9432	3.0138	1191.6523	0
209.9458	3.0138	1193.0742	0
409.7439	0	1193.1786	0
410.0046	0	1323.8446	0
414.2034	0	1323.9554	0
414.3911	0	1383.9495	0
619.1737	0	1384.1451	0
619.2511	0	1500.2814	0.1405
620.7919	0	1500.2917	0.1201
620.8655	0	1501.2171	31.0375
688.2842	0	1501.2259	31.0446
688.2999	0	1588.2563	0
760.3193	155.3722	1588.4158	0
775.1121	0	1588.429	0
903.4445	0	1588.5974	0
903.4507	0	3194.2013	0
915.043	1.7343	3194.2115	0
915.0527	1.7342	3200.4646	0
994.1148	0	3200.589	0
994.3546	0	3200.8865	0
998.9439	0.0775	3201.004	0
999.5796	0	3211.1703	0.012
1000.1213	0.0001	3211.1718	0.0092
1000.372	0	3211.7722	1.0006
1019.0314	0	3211.7752	0.9976
1019.0537	0	3217.7085	0.1198
1037.4417	0.0001	3218.077	0

Table S196. Cartesian coordinates for the optimized geometry of isomer 6b-triplet of $Co^{+}(C_2H_2)_6$.

Z	x	y	z
27	-0.000045000	-0.000062000	0.000809000
6	1.765333000	1.139890000	-0.204345000
6	2.101174000	0.034618000	0.205101000
1	2.668107000	-0.818736000	0.520579000
1	1.761672000	2.164388000	-0.519873000
6	-1.020448000	-1.837344000	0.205202000
6	0.104406000	-2.098834000	-0.205124000
1	0.993323000	-2.607720000	-0.521362000
1	-2.042768000	-1.901852000	0.521174000
6	-1.080716000	1.802197000	0.206793000
6	-1.870086000	0.959096000	-0.203218000
1	-2.755532000	0.443859000	-0.518940000
1	-0.625067000	2.719656000	0.522689000
6	3.771452000	-3.168139000	-0.538845000
6	3.410951000	-3.553542000	0.537331000
1	3.121998000	-3.928261000	1.491168000
1	4.126060000	-2.854885000	-1.492701000
6	-4.783481000	-1.177262000	0.536886000
6	-4.629338000	-1.681031000	-0.539732000
1	-4.534792000	-2.143703000	-1.494042000
1	-4.964034000	-0.740721000	1.491112000
6	0.858129000	4.849596000	-0.539546000
6	1.372782000	4.731000000	0.536428000
1	1.842293000	4.668961000	1.490066000
1	0.409013000	4.999217000	-1.493285000

Table S197. Predicted frequencies (cm^{-1}) and IR intensities (km/mol) for isomer 6b-triplet of $Co^{+}(C_2H_2)_6$.

Frequency (cm^{-1})	Intensity (km/mol)	Frequency (cm^{-1})	Intensity (km/mol)
14.7324	0.4997	722.0258	0.0005
23.9421	0.1623	758.6232	0.7751
24.009	0.162	771.839	1.345
32.4324	0.1174	772.0522	1.1517
32.4622	0.118	777.742	56.217
50.3976	0	779.0116	119.6145
57.4697	0.0089	779.019	120.0027
57.4882	0.0087	787.852	253.6613
66.814	0.0003	787.8594	252.7128
69.3418	0.0832	791.6294	17.6987
72.4686	3.5715	791.7337	17.0721
72.5503	3.5741	792.3117	0.0277
93.8982	2.2853	796.2392	152.7966
97.7267	0.4507	826.6926	4.3245
97.7856	0.4451	826.7961	4.439
121.3412	0.0806	842.6855	0.0016
141.5022	0.2188	1910.5924	4.8638
141.6717	0.2241	1910.6541	4.9976
144.3542	0.0001	1910.7969	0.1113
177.8248	6.9489	2062.4059	13.6167
177.9331	6.9451	2062.4224	13.6191
269.5981	0.0002	2062.7355	0.0178
274.5729	2.9145	3291.2448	13.6049
274.6326	2.917	3296.8171	469.9777
408.8325	0.6216	3296.9334	469.4598
409.6811	10.853	3377.0059	125.8288
409.7405	10.8452	3377.1735	125.5239
641.4858	0.0001	3384.4795	0.0156
641.5701	0.0035	3397.1254	33.8235
641.6018	0.003	3397.1336	33.4371
669.0173	0.207	3397.2551	282.0779
669.0289	0.2144	3496.8227	1.9743
669.3112	0.3475	3496.833	1.9881
718.9462	0.2345	3496.896	0.0191
719.0429	0.2328		

Table S198. Cartesian coordinates for the optimized geometry of isomer 6c-triplet of $Co^{+}(C_2H_2)_6$.

Z	x	y	z
27	0.088159000	0.264497000	0.664295000
6	-1.394941000	1.128042000	1.891167000
6	-1.989489000	0.553175000	0.988633000
1	-2.733574000	0.119409000	0.350352000
1	-1.150942000	1.689706000	2.767584000
6	0.737439000	-1.305727000	-0.613273000
6	-0.372773000	-1.622798000	-0.207813000
1	-1.308786000	-2.114885000	-0.035989000
1	1.701557000	-1.294572000	-1.080058000
6	1.469058000	1.767968000	1.167213000
6	2.119962000	0.740021000	1.027849000
1	2.908112000	0.018805000	0.937853000
1	1.172334000	2.780432000	1.340702000
6	-4.168101000	-2.261776000	0.085730000
6	-3.958594000	-1.959257000	-1.054999000
1	-3.808096000	-1.712760000	-2.079822000
1	-4.393308000	-2.548415000	1.086168000
6	4.593432000	-1.067252000	-0.992113000
6	4.342001000	-2.046752000	-0.348699000
1	4.158349000	-2.934681000	0.209469000
1	4.859035000	-0.214158000	-1.571170000
6	-1.228847000	2.860163000	-1.906962000
6	-0.490151000	2.206225000	-2.586365000
1	0.154256000	1.653534000	-3.227162000
1	-1.893206000	3.463957000	-1.336109000

Table S197. Predicted frequencies (cm^{-1}) and IR intensities (km/mol) for isomer 6c-triplet of $Co^+(C_2H_2)_6$.

Frequency (cm^{-1})	Intensity (km/mol)	Frequency (cm^{-1})	Intensity (km/mol)
1.0881	0.3554	711.371	0.6725
14.5146	0.2705	725.5488	19.793
16.8596	0.0894	748.7209	1.2088
25.9723	0.8846	756.4638	6.4047
31.8958	0.7063	770.637	106.0091
38.0276	0.3425	777.5758	123.7583
43.1943	0.3709	778.1684	48.0421
54.6622	0.0107	778.6403	192.1946
57.4842	0.0865	781.2353	81.3006
60.3835	0.1803	784.545	100.6928
68.2109	1.8042	787.87	83.1651
70.7265	0.9434	789.2834	154.7537
72.1259	2.3306	791.3404	50.1781
92.4906	1.4139	800.0536	1.8631
95.7907	0.1076	820.2148	3.8632
111.399	0.47	835.0978	1.7704
131.4158	0.8434	1915.9759	5.8293
134.4841	0.3671	1918.4981	9.8729
142.5897	0.5925	1920.6256	1.2439
160.8185	2.9127	2062.3851	12.6426
171.4956	5.5582	2062.6212	4.5834
259.6938	2.3763	2066.1521	4.4028
267.5792	0.6285	3304.2243	129.824
271.8139	3.1454	3312.3467	296.6041
397.4094	6.3137	3313.4025	302.5058
399.1116	2.8503	3390.1356	79.3209
403.2834	8.2096	3397.1898	76.672
629.1906	0.0556	3397.3903	151.3759
641.6117	0.002	3404.9301	94.739
642.6945	0.0576	3407.0213	39.4408
643.632	0.265	3407.2014	70.2687
668.5566	0.0699	3496.8828	1.4037
668.8223	0.5674	3497.0312	1.083
690.4659	2.2993	3505.8725	0.8622
695.1971	10.4817		

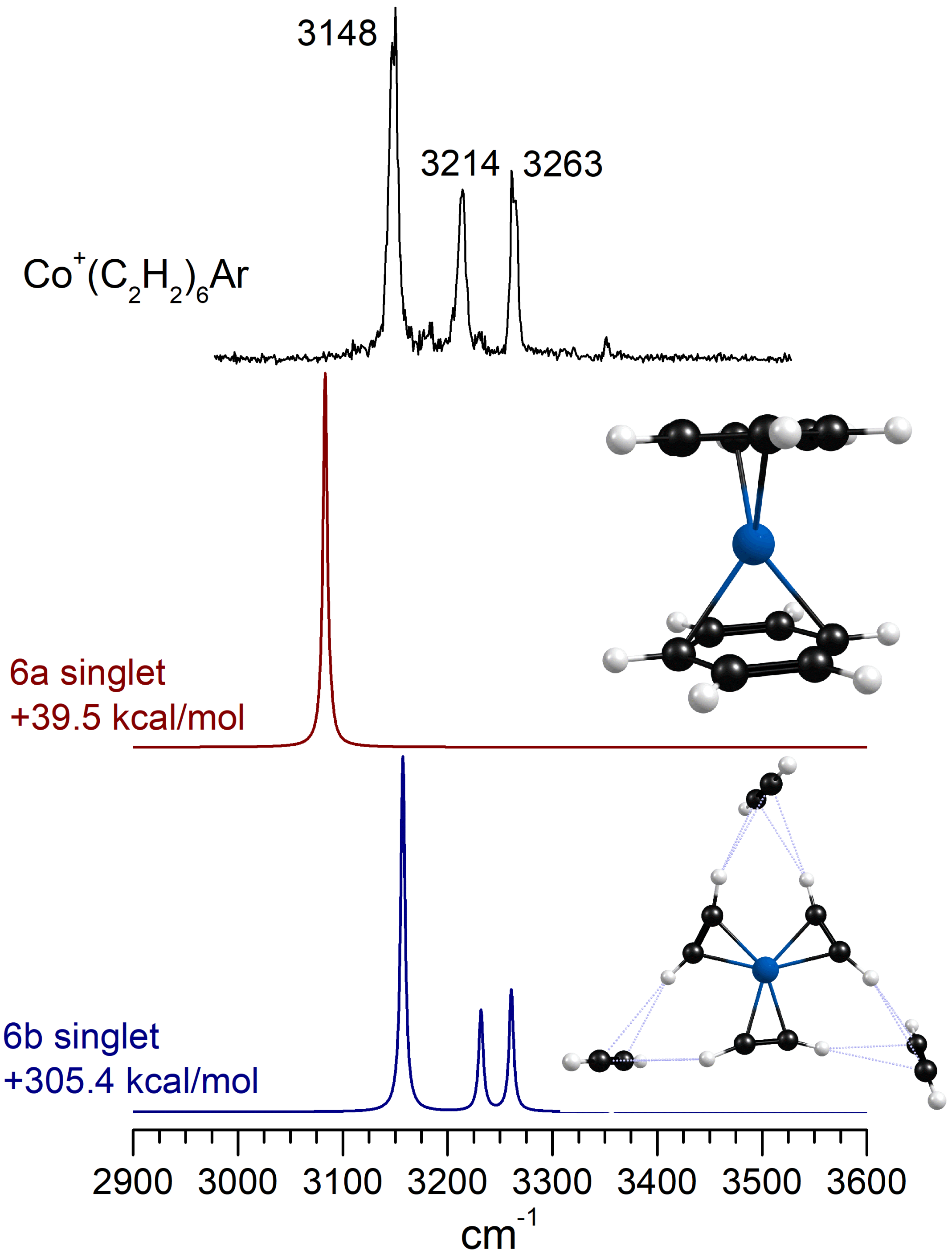

Figure S60. The experimental spectrum for $Co^+(C_2H_2)_6Ar$ with simulated spectra for $Co^+(C_2H_2)_6$ as a singlet and all predicted isomers of singlet-$Co^+(C_2H_2)_6$.

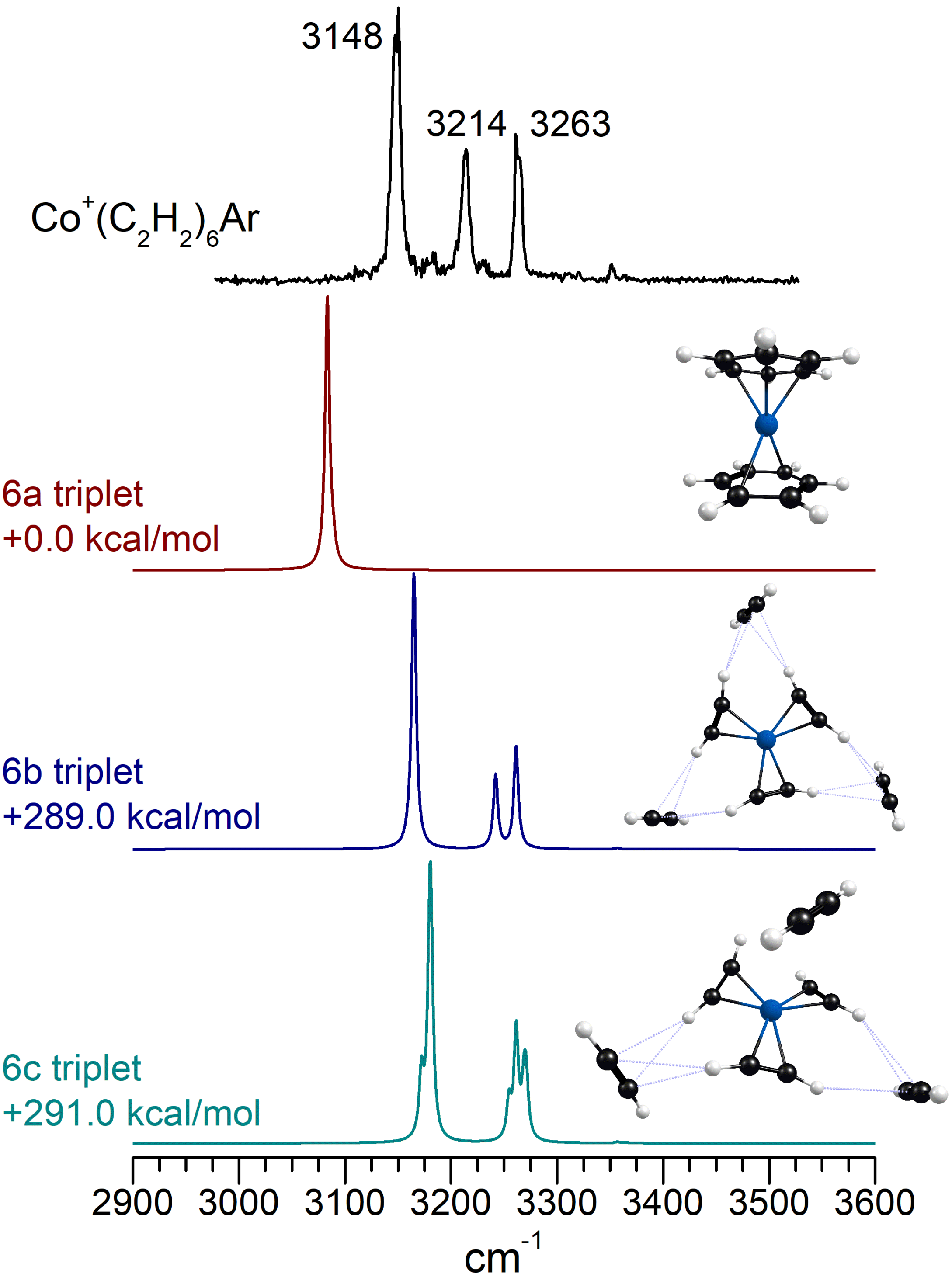

Figure S61. The experimental spectrum for $Co^{+}(C_2H_2)_6Ar$ with simulated spectra for $Co^{+}(C_2H_2)_6$ as a triplet and all predicted isomers of triplet-$Co^{+}(C_2H_2)_6$.
